# Supplementary material for: Enantioselective functionalization at the C4 position of pyridinium salts through NHC catalysis
Source: Nat Commun. 2022 Apr 1;13:1776. doi: 10.1038/s41467-022-29462-7 (PMC8975994; doi:10.1038/s41467-022-29462-7)
Supplement: Supplementary file 1 — Supplementary Information [file 41467_2022_29462_MOESM1_ESM.pdf]

Supplementary Information

Enantioselective Functionalization at the C4 Position of Pyridinium Salts through NHC Catalysis

Choi et al.

Supplementary Methods

General Information

Unless stated otherwise, reactions were performed in flame-dried glassware. Analytical thin layer chromatography (TLC) was performed on precoated silica gel 60 F254 plates, and visualization on TLC was achieved by UV light (254 and 365 nm). Flash column chromatography was performed on silica gel (400-630 mesh) or a CombiFlash® Rf + system with RediSep® Rf silica columns (230-400 mesh) using a proper eluent. ^{1}H NMR was recorded on Agilent Technologies DD2 600 MHz, Bruker 500MHz, and Bruker 400 MHz. Chemical shifts were quoted in parts per million (ppm) referenced to the appropriate solvent peak or 0.0 ppm for tetramethylsilane. The following abbreviations were used to describe peak splitting patterns when appropriate: br = broad, s = singlet, d = doublet, t = triplet, q = quartet, m = multiplet, dd = doublet of doublet, td = triplet of doublet, ddd = doublet of doublet of doublet. Coupling constants, *J*, were reported in hertz unit (Hz). ^{13}C NMR was recorded on Agilent Technologies DD2 150 MHz, Bruker 125MHz and Bruker 100 MHz, and was fully decoupled by broad band proton decoupling. Chemical shifts were reported in ppm referenced to the centerline of a pentet at 77.2 ppm of $CDCl_3$. ^{19}F NMR was recorded on Brucker Advance 376 MHz. High-resolution mass spectra were obtained by using EI or FAB method from Korea Basic Science Intitute (Daegu) or ESI method from Korea Basic Science Institute (Ochang) and KAIST Research Analysis Center (Daejeon). Commercial grade reagents and solvents were used without further purification except as indicated below. We have used commercially available blue LEDs as light sources from Kessil (PR160L-440). Reaction mixture is placed 5 cm far from the light source without any other filters. Data collections of parabar oil-coated single crystal were carried out on a Bruker D8 Quest diffractometer equipped with a monochromator in the Mo Kα radiation and PHOTON 2 area detector. The diffraction data was integrated, scaled, and reduced by using the Bruker APEX3 software. The structure was solved and refined using SHELX programs. High pressure liquid chromatography (HPLC) analysis was performed on Shimadzu Prominence HPLC system composed of LC-40D and SPD-M40 photodiode array detector, and on Agilent 1260 Infinity HPLC system at 25 ^{o}C, using CHIRALCEL OD-H and CHIRALPAK IB column (250 mmL x 4.6 mm ID). Optical rotations were recorded with Jasco P-2000 Polarimeter equipped with temperature controller. The Absorption spectra were measured by a spectrophotometer (V-530 UV/Vis Spectrophotometer, Jasco, Inc.).

Procedures for the Preparation of Starting Materials

1. General procedure for the preparation of pyridinium salts

(1) Preparation of *N*-amino pyridinium salts from pyridines

Amination of pyridine was conducted using the previously developed method with hydroxylamine-*O*-sulfonic acid[1] or *O*-mesitylsulfonylhydroxylamine (MSH)[2]. MSH was purchased from a commercial source or synthesized by using the previous developed method[3].

(2) General procedure for *N*-Protected 1-aminopyridinium ylides.

To a solution of 1-aminopyridinium (1 equiv) in dichloromethane (0.2 M) were added triethylamine (2.2 equiv) and 4-toluensulfonyl chloride (1.0 equiv) at 0 °C. The reaction mixture was stirred at room temperature for 24 h. The resulting mixture was diluted with 1N NaOH and extracted with dichloromethane three times. The combined organic layers were dried over sodium sulfate, filtered, and concentrated in a vacuum. The resulting mixture was purified by flash column chromatography on silica gel (CH_2Cl_2 : MeOH = 20 : 1) to obtain N-protected 1-aminopyridinium ylides.

(3) General procedure for *N*-Protected aminopyridinium salts.

To a solution of *N*-protected 1-aminopyridinium ylide (1 equiv) in dichloromethane (0.1 M) were added trimethyloxonium tetrafluoroborate (meerwein's reagent, 1.1 equiv) at room temperature. The reaction mixture was stirred at room temperature for 24 h. The resulting mixture was concentrated under reduced pressure. The product was recrystallized with diethyl ether from CH_2Cl_2 and MeOH (20:1) solution at -20 °C. A white solid product was obtained.

Supplementary Fig. 1. General procedure for *N*-protected aminopyridinium salts

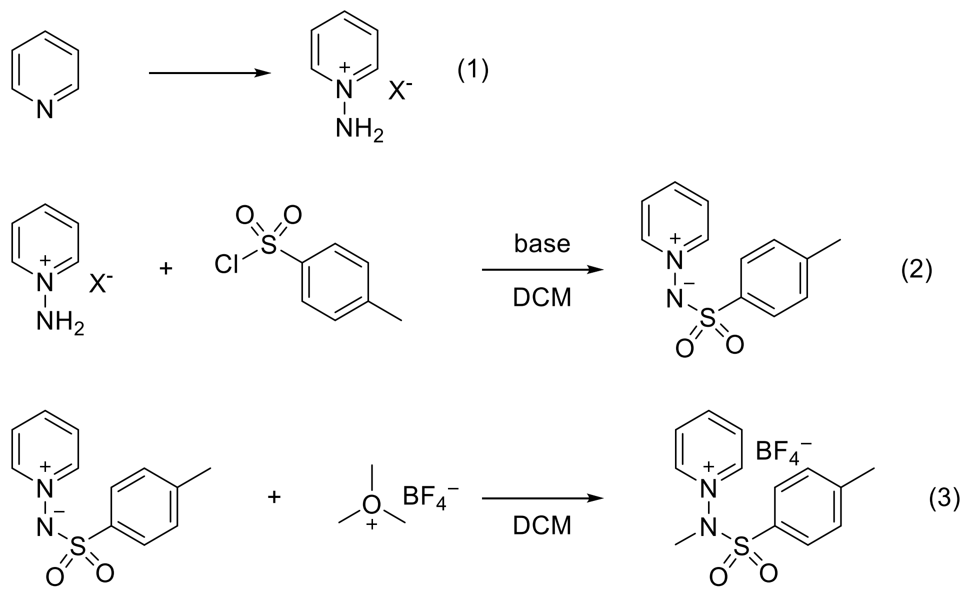

2. General procedure for the preparation of α,β-unsaturated aldehydes (enals)

Most of enal substrates used in this paper were purchased from commercial sources, and used without additional purification. Few enals including late-stage functionalization substrates were synthesized through the reference.[4] Aryl iodide containing estrone derivative (**7a**) was synthesized through the reference.[5] Boc-4-iodo-*L*-phenylalanine (CAS: 62129-44-6) was purchased from commercial source, and used after esterification to synthesize corresoponding enal substrates (**7b**). Oxaprozin substrate[6] (**7c**) and febuxostat analog substrate[7] (**7d**) was synthesized through the references. All ^{1}H and ^{13}C NMR data were matched with the references.

(*E*)-3-((8*R*,9*S*,13*S*,14*S*)-13-Methyl-17-oxo-7,8,9,11,12,13,14,15,16,17-decahydro-6*H*-cyclopenta[*a*] phenanthren-3-yl)acrylaldehyde (7a)

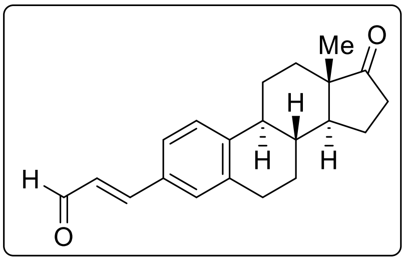

White solid (250 mg, 62% isolated yield from estrone). **^{1}H NMR** (400 MHz, $CDCl_3$) δ 9.68 (d, *J* = 7.8 Hz, 1H), 7.42 (d, *J* = 15.9 Hz, 1H), 7.38 – 7.33 (m, 2H), 7.32 – 7.27 (m, 1H), 6.68 (dd, *J* = 15.9, 7.7 Hz, 1H), 3.00 – 2.86 (m, 2H), 2.55 – 2.47 (m, 1H), 2.47 – 2.40 (m, 1H), 2.33 (td, *J* = 10.8, 4.2 Hz, 1H), 2.21 – 2.02 (m, 3H), 2.01 – 1.93 (m, 1H), 1.70 – 1.43 (m, 6H), 0.92 (s, 3H). **^{13}C NMR** (100 MHz, $CDCl_3$) δ 220.6, 193.9, 153.0, 143.9, 137.5, 131.7, 129.3, 128.1, 126.3, 126.1, 50.6, 48.0, 44.8, 38.0, 35.9, 31.6, 29.4, 26.4, 25.7, 21.7, 13.9. **HRMS** (ESI^+) m/z calcd. For $C_{21}H_{25}O_2^+$ $[M+H]^+$: 309.1855, found 309.1856.

Methyl (*S*,*E*)-2-((tert-butoxycarbonyl)amino)-3-(4-(3-oxoprop-1-en-1-yl)phenyl)propanoate (7b)

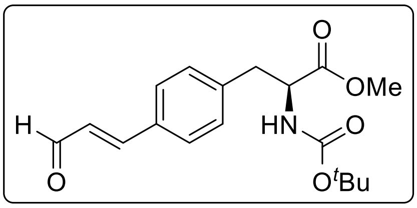

Light yellow solid (700 mg, 78% isolated yield from Boc-4-iodo-*L*-phenylalanine). **^{1}H NMR** (600 MHz, $CDCl_3$) δ 9.70 (d, *J* = 7.6 Hz, 1H), 7.50 (d, *J* = 7.9 Hz, 2H), 7.45 (d, *J* = 16.0 Hz, 1H), 7.21 (d, *J* = 7.8 Hz, 2H), 6.70 (dd, *J* = 15.9, 7.7 Hz, 1H), 5.05 – 4.95 (m, 1H), 4.65 – 4.58 (m, 1H), 3.73 (s, 3H), 3.18 (dd, J = 13.9, 5.8 Hz, 1H), 3.07 (dd, J = 14.0, 6.3 Hz, 1H), 1.41 (s, 9H). **^{13}C NMR** (100 MHz, $CDCl_3$) δ 193.8, 172.2, 155.1, 152.5, 140.0, 133.0, 130.3, 128.8, 128.6, 80.2, 54.4, 52.5, 38.5, 28.4. **HRMS** (EI^+) m/z calcd. For $C_{18}H_{23}NO_5^+$ $[M]^+$: 333.1576, found 333.1576. **HPLC Analysis**. CHIRALPAK IB, 25 °C; *n*-hexane:*i*-PrOH = 85:15, 1.0 mL/min, 254 nm, t_{R1} (major) = 11.57 min,

t_{R2} (minor) = 12.74 min, 81:19 er. Enantioenriched aryl iodide was partially racemized during Pd-catalyzed Heck reaction.[4] This enantiomeric mixture was used in the reaction.

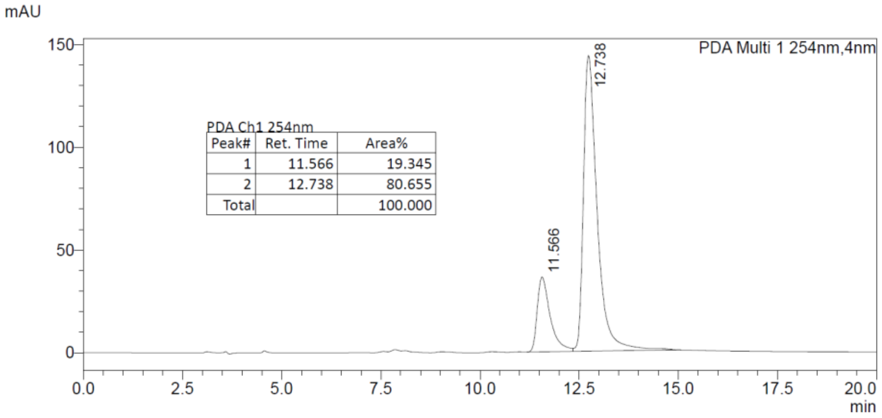

Supplementary Fig. 2. HPLC traces of enantiomeric mixture of **7b**.

Ethyl (*E*)-2-(4-isobutoxy-3-(3-oxoprop-1-en-1-yl)phenyl)-4-methylthiazole-5-carboxylate (7d)

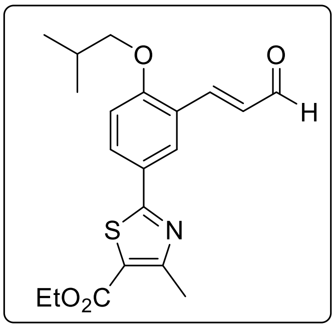

White solid (200 mg, total 14% isolated yield from aryl aldehyde). **^{1}H NMR** (400 MHz, $CDCl_3$) δ 9.72 (d, *J* = 7.7 Hz, 1H), 8.16 (d, *J* = 2.3 Hz, 1H), 7.97 (dd, *J* = 8.7, 2.2 Hz, 1H), 7.81 (d, *J* = 16.1 Hz, 1H), 6.99 (d, *J* = 8.8 Hz, 1H), 6.92 (dd, *J* = 16.1, 7.7 Hz, 1H), 4.35 (q, *J* = 7.1 Hz, 2H), 3.90 (d, *J* = 6.3 Hz, 2H), 2.77 (d, *J* = 0.9 Hz, 3H), 2.29 – 2.15 (m, 1H), 1.39 (t, *J* = 7.1 Hz, 3H), 1.09 (d, *J* = 6.5 Hz, 6H). **^{13}C NMR** (100 MHz, $CDCl_3$) δ 194.4, 168.8, 162.4, 161.2, 159.9, 147.1, 131.0, 130.3, 127.6, 126.1, 123.8, 121.6, 112.7, 75.5, 61.4, 28.4, 19.5, 17.7, 14.5. **HRMS** (ESI$^+$) m/z calcd. For $C_{20}H_{24}NO_4S^+$ [M+H]$^+$: 374.1426, found 374.1426.

400 MHz, ^{1}H NMR in CDCl₃.

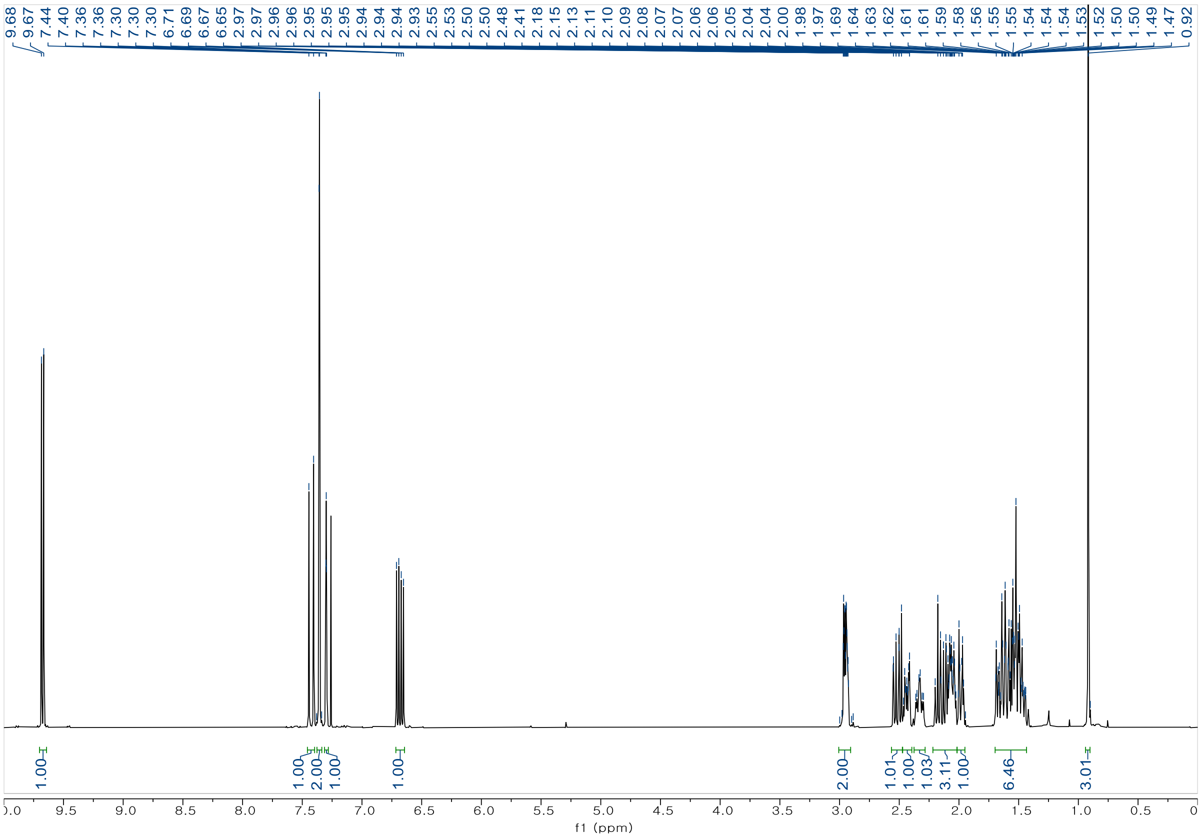

100 MHz, ^{13}C NMR in CDCl₃.

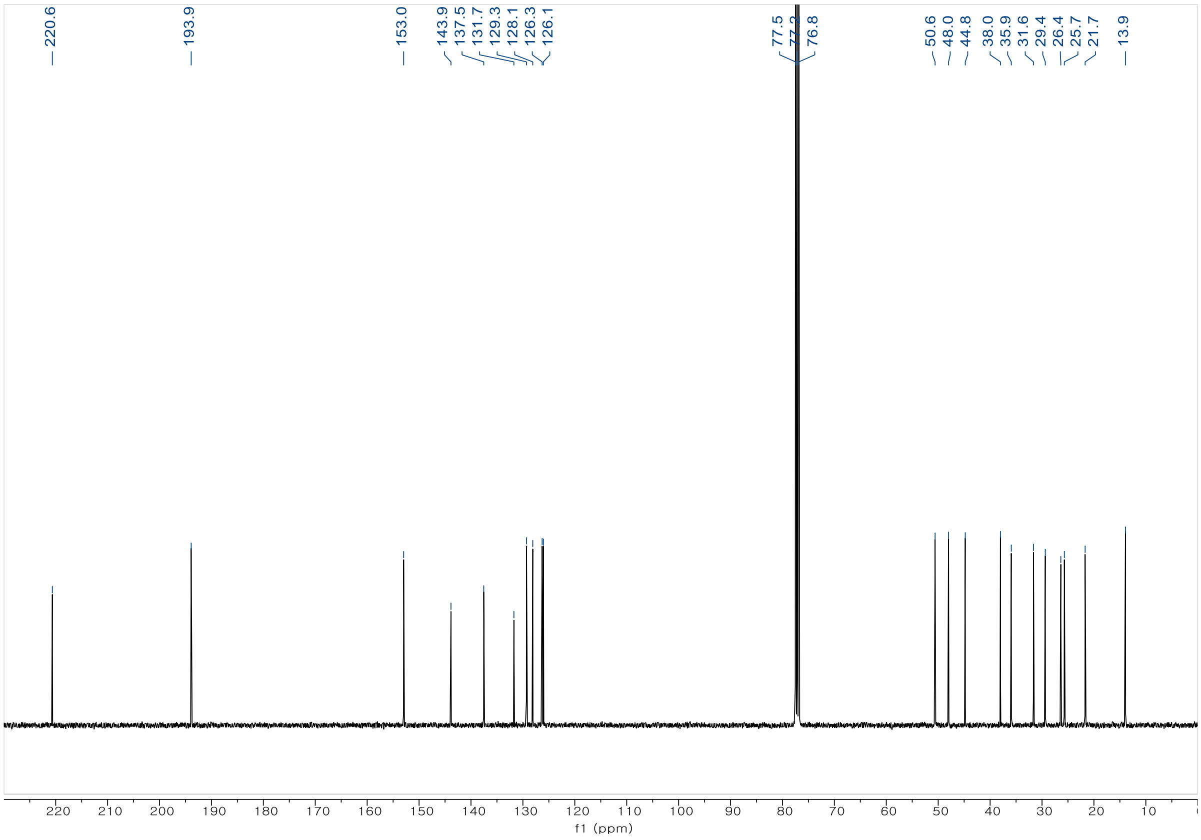

Supplementary Fig. 3. ^{1}H and ^{13}C NMR of **7a**.

600 MHz, ^{1}H NMR in $CDCl_3$.

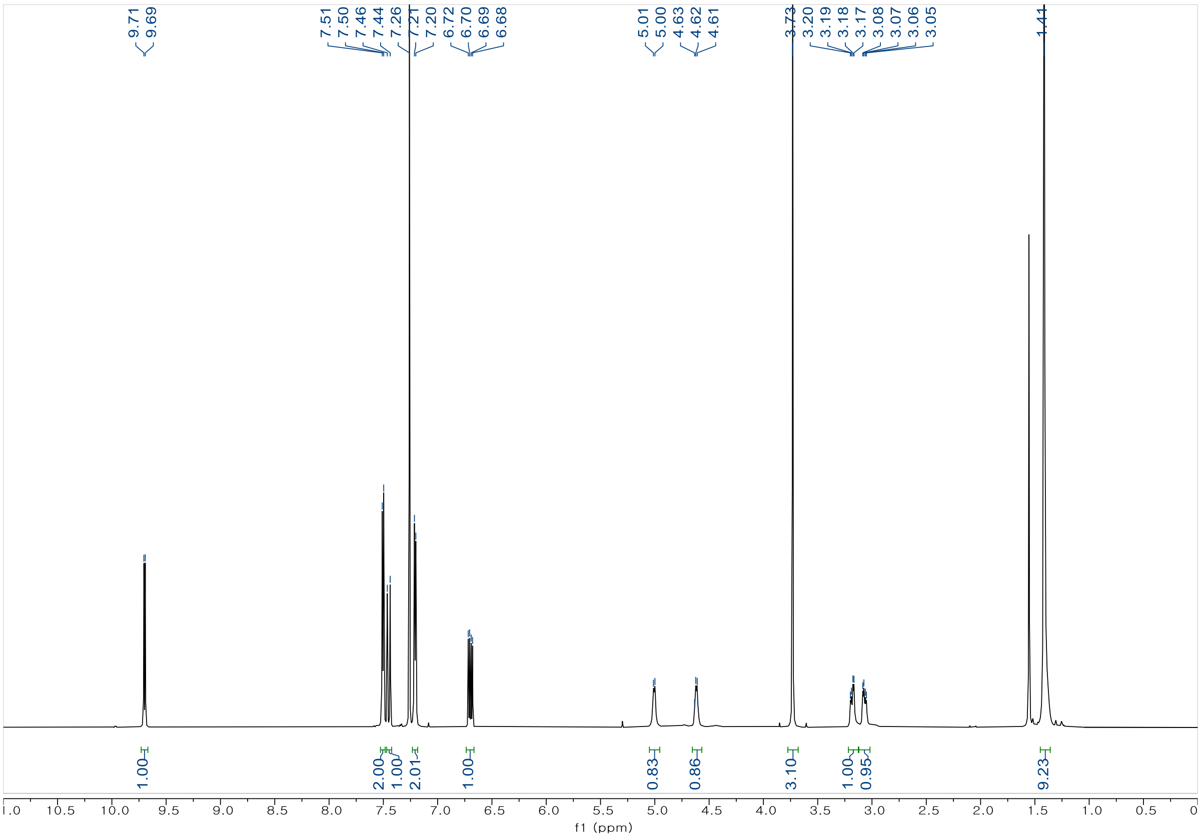

100 MHz, ^{13}C NMR in $CDCl_3$.

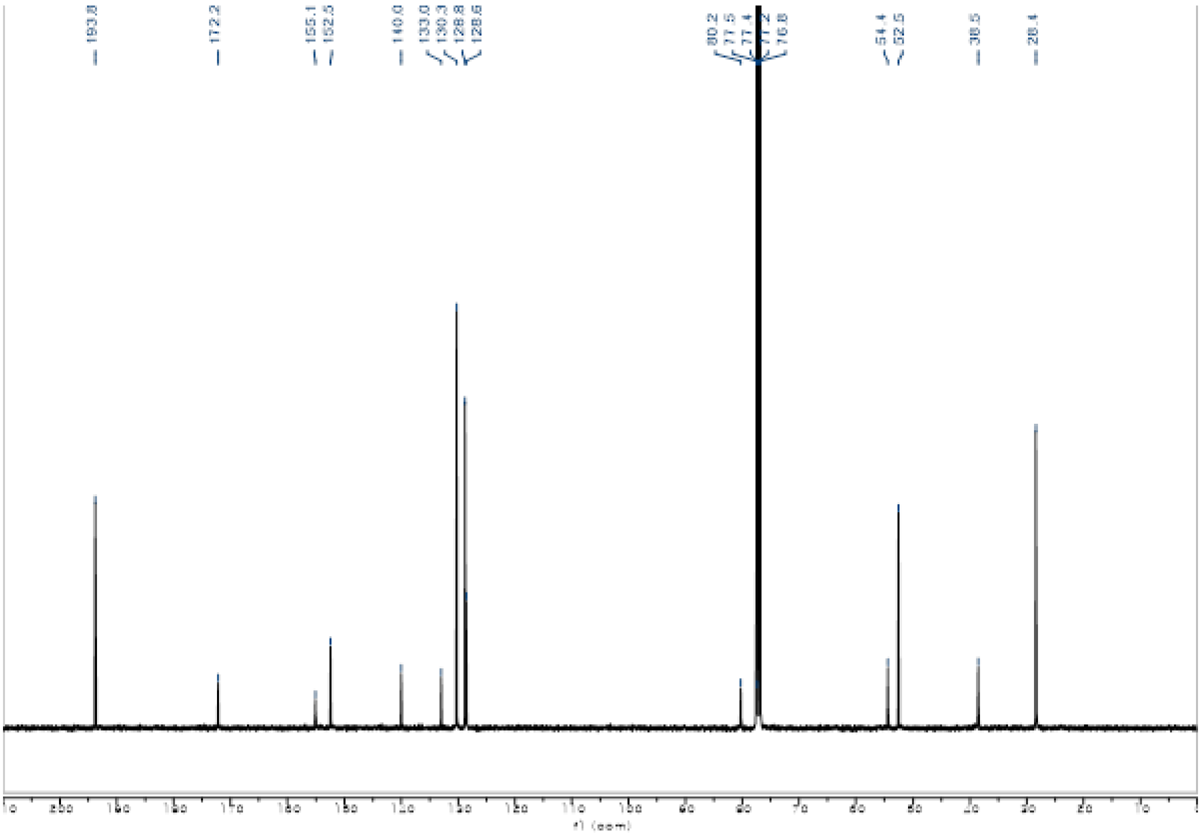

Supplementary Fig. 4. ^{1}H and ^{13}C NMR of **7b**.

400 MHz, ^{1}H NMR in $CDCl_3$.

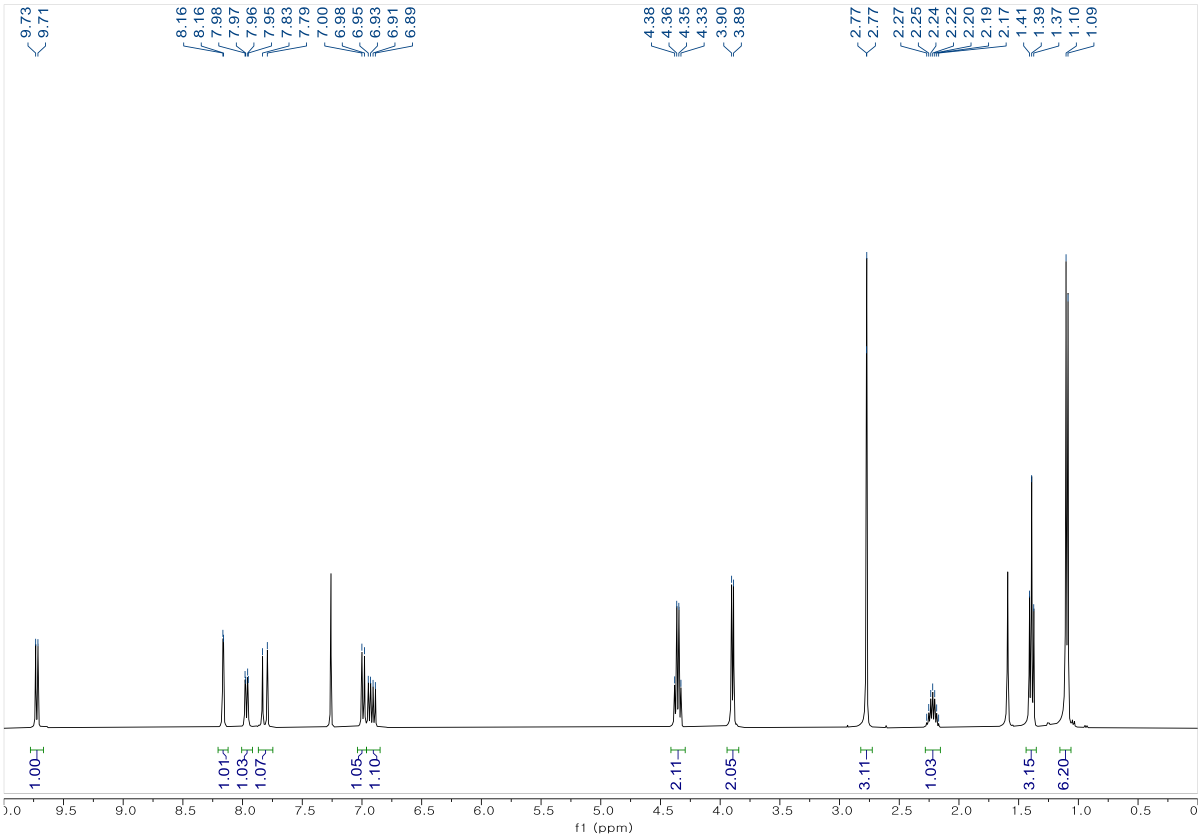

100 MHz, ^{13}C NMR in $CDCl_3$.

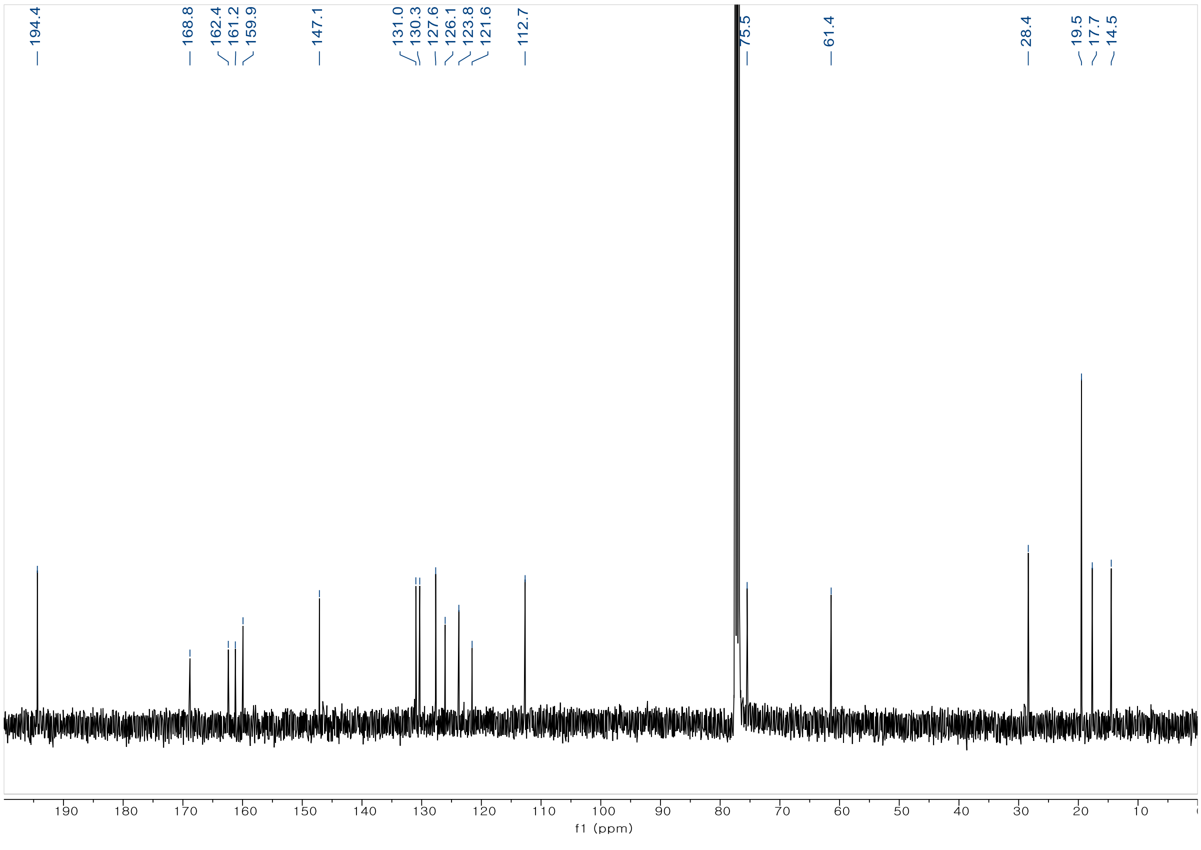

Supplementary Fig. 5. ^{1}H and ^{13}C NMR of **7d**.

3. General procedure for the preparation of N-heterocyclic carbene (NHC) catalyst

NHC catalysts used in this paper were purchased from commercial sources, or synthesized through the references.[8,9]

(*R*)-5-Benzhydryl-2-(4-(trifluoromethyl)phenyl)-6,7-dihydro-5*H*-pyrrolo[2,1-*c*][1,2,4]triazol-2-ium tetrafluoroborate (5c)

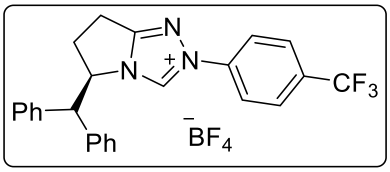

Light yellow solid (560mg, 63% isolated yield from pyrrolidinone). **^{1}H NMR** (400 MHz, DMSO-*d$_6$*) δ 9.66 (s, 1H), 8.11 – 8.04 (m, 2H), 8.03 – 7.96 (m, 2H), 7.51 – 7.45 (m, 4H), 7.45 – 7.26 (m, 6H), 5.87 (ddd, *J* = 10.2, 7.8, 4.3 Hz, 1H), 4.55 (d, *J* = 10.2 Hz, 1H), 3.22 (dd, *J* = 8.4, 6.7 Hz, 2H), 2.95 – 2.81 (m, 1H), 2.48 – 2.39 (m, 1H). **^{13}C NMR** (100 MHz, DMSO-*d$_6$*) δ 163.0, 140.2, 140.1, 138.3, 138.2, 130.5 (q, *J* = 32.6 Hz), 129.3, 129.0, 128.3, 128.1, 127.7, 127.4 (q, *J* = 3.2 Hz), 123.5 (q, *J* = 272.7 Hz), 122.3, 63.5, 53.7, 32.4, 20.7. **^{19}F NMR** (376 MHz, DMSO-*d$_6$*) -61.29, -148.33, -148.39. **HRMS** (FAB$^+$) m/z calcd. For $C_{25}H_{21}F_3N_3^+$ [M]$^+$: 420.1688, found 420.1691.

(5a*R*,10b*S*)-2-(4-(Trifluoromethyl)phenyl)-5a,10b-dihydro-4*H*,6*H*-indeno[2,1-*b*][1,2,4]triazolo[4,3-*d*][1,4]oxazin-2-ium tetrafluoroborate (5d)

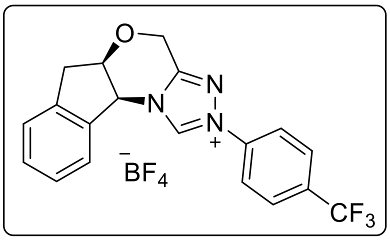

White solid (280 mg, 31% isolated yield from amide substrate). **^{1}H NMR** (400 MHz, CD_3OD) δ 8.22 (d, *J* = 8.2 Hz, 2H), 8.04 (d, *J* = 8.6 Hz, 2H), 7.70 – 7.62 (m, 1H), 7.45 – 7.32 (m, 3H), 6.03 (d, *J* = 4.1 Hz, 1H), 5.26 (d, *J* = 16.3 Hz, 1H), 5.09 (d, *J* = 16.4 Hz, 1H), 5.03 – 4.96 (m, 1H), 4.58 (s, 1H), 3.48 (dd, *J* = 17.1, 5.0 Hz, 1H), 3.28 (d, *J* = 16.4 Hz, 1H). **^{13}C NMR** (100 MHz, CD_3OD) δ 151.97, 141.91, 139.41, 136.44, 133.66 (q, *J* = 33.3 Hz), 130.84, 128.64, 128.58 (q, *J* = 3.8 Hz), 126.73, 125.18, 124.91 (q, *J* = 271.8 Hz), 125.2, 123.03, 78.67, 63.40, 61.12, 38.08. **^{19}F NMR** (376 MHz, CD_3OD) -64.33, -154.28, -154.33. **HRMS** (FAB$^+$) m/z calcd. For $C_{19}H_{15}F_3N_3O^+$ [M]$^+$: 358.1167, found 358.1165.

(5a*R*,10b*S*)-2-(4-Chlorophenyl)-5a,10b-dihydro-4*H*,6*H*-indeno[2,1-*b*][1,2,4]triazolo[4,3-*d*][1,4]oxazin-2-ium tetrafluoroborate (5f)

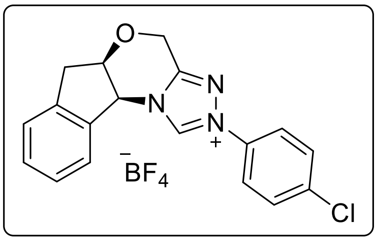

Light brown solid (1.05 g, 51% isolated yield from amide substrate). **^{1}H NMR** (400 MHz, $CDCl_3$) δ 10.70 (s, 1H), 7.91 – 7.82 (m, 2H), 7.59 – 7.52 (m, 1H), 7.44 – 7.29 (m, 5H), 5.93 (d, *J* = 4.0 Hz, 1H), 5.05 (s, 2H), 4.99 – 4.90 (m, 1H), 3.23 – 3.09 (m, 2H). **^{13}C NMR** (100 MHz, $CDCl_3$) δ 150.4, 140.0, 139.6, 137.2, 134.8, 133.3, 130.5, 130.0, 128.2, 125.8, 124.3, 121.9, 84.5, 62.4, 60.3, 37.5. **^{19}F NMR** (376 MHz, $CDCl_3$) -150.51, -150.56. **HRMS** (FAB$^+$) m/z calcd. For $C_{18}H_{15}ClN_3O^+$ [M]$^+$: 324.0904, found 324.0901.

(5a*S*,10b*R*)-2-(4-chlorophenyl)-5a,10b-dihydro-4*H*,6*H*-indeno[2,1-*b*][1,2,4]triazolo[4,3-d][1,4]oxazin-2-ium tetrafluoroborate (5f*)

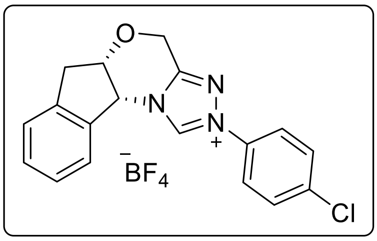

Synthesized according to the same procedure of **5f** starting from (1*R*,2*S*)-1-amino-2,3-dihydro-1*H*-inden-2-ol.

400 MHz, ^{1}H NMR in DMSO.

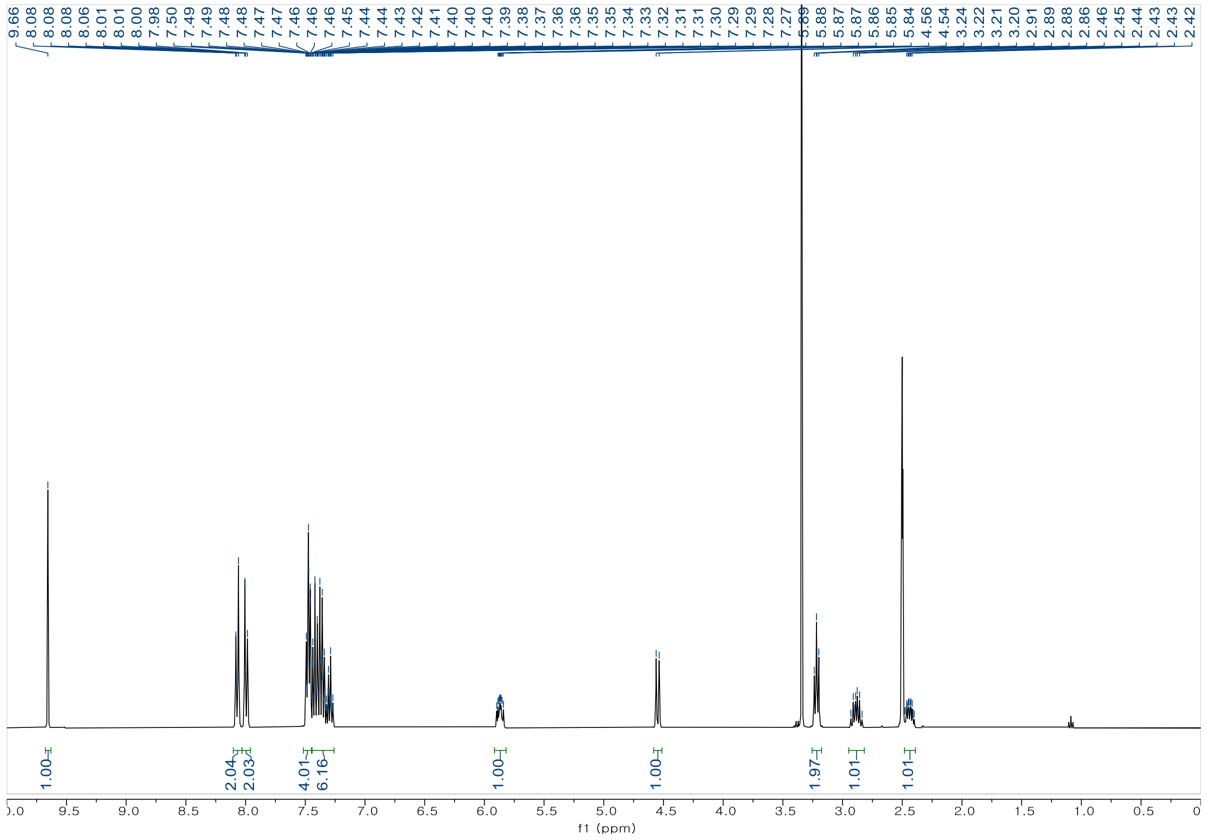

100 MHz, ^{13}C NMR in DMSO.

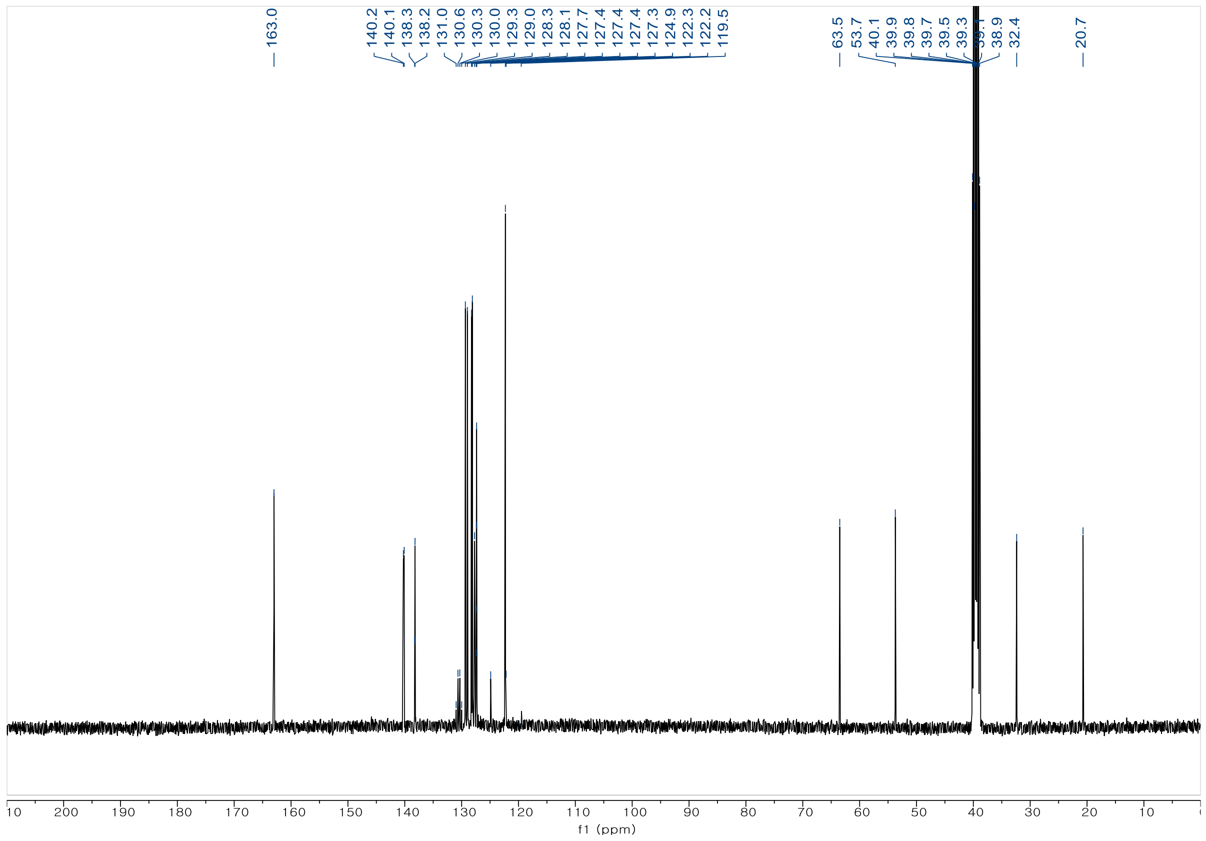

376 MHz, ^{19}F NMR in DMSO.

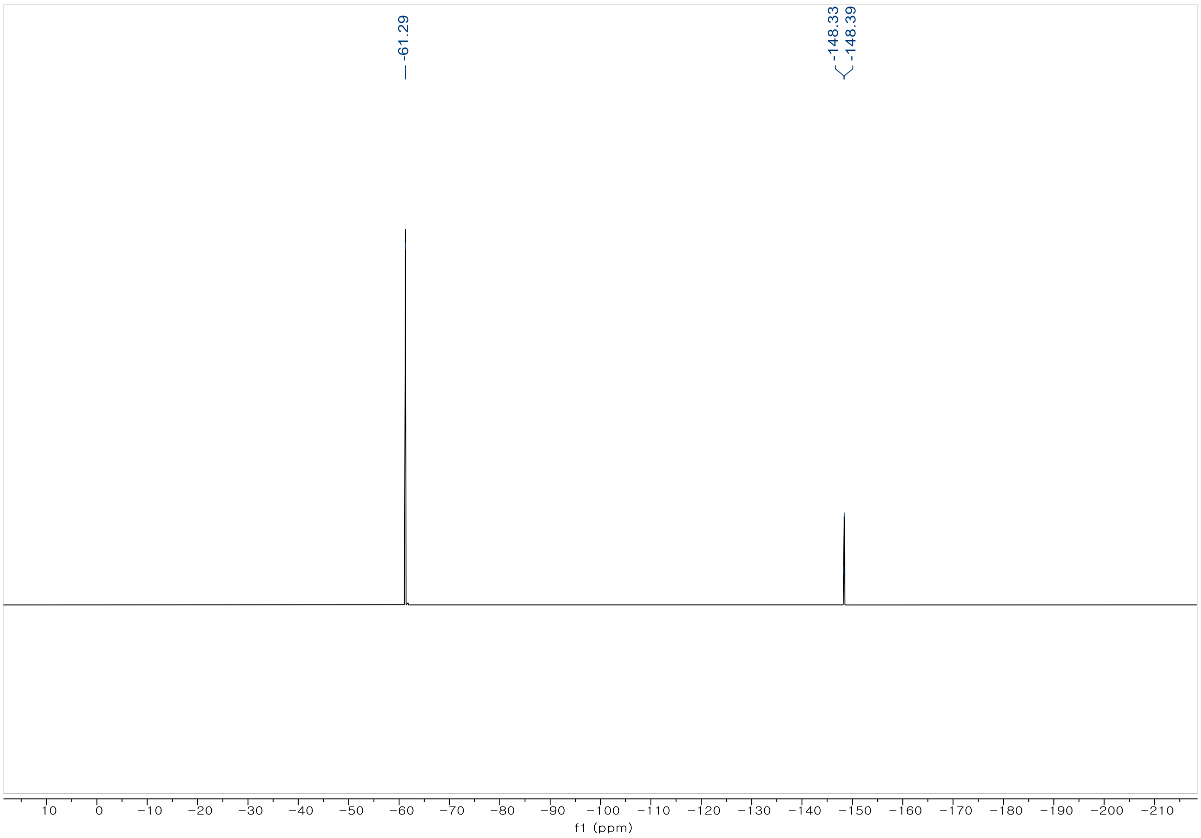

Supplementary Fig. 6. ^{1}H, ^{13}C and ^{19}F NMR of **5c**.

400 MHz, ^{1}H NMR in CD_3OD.

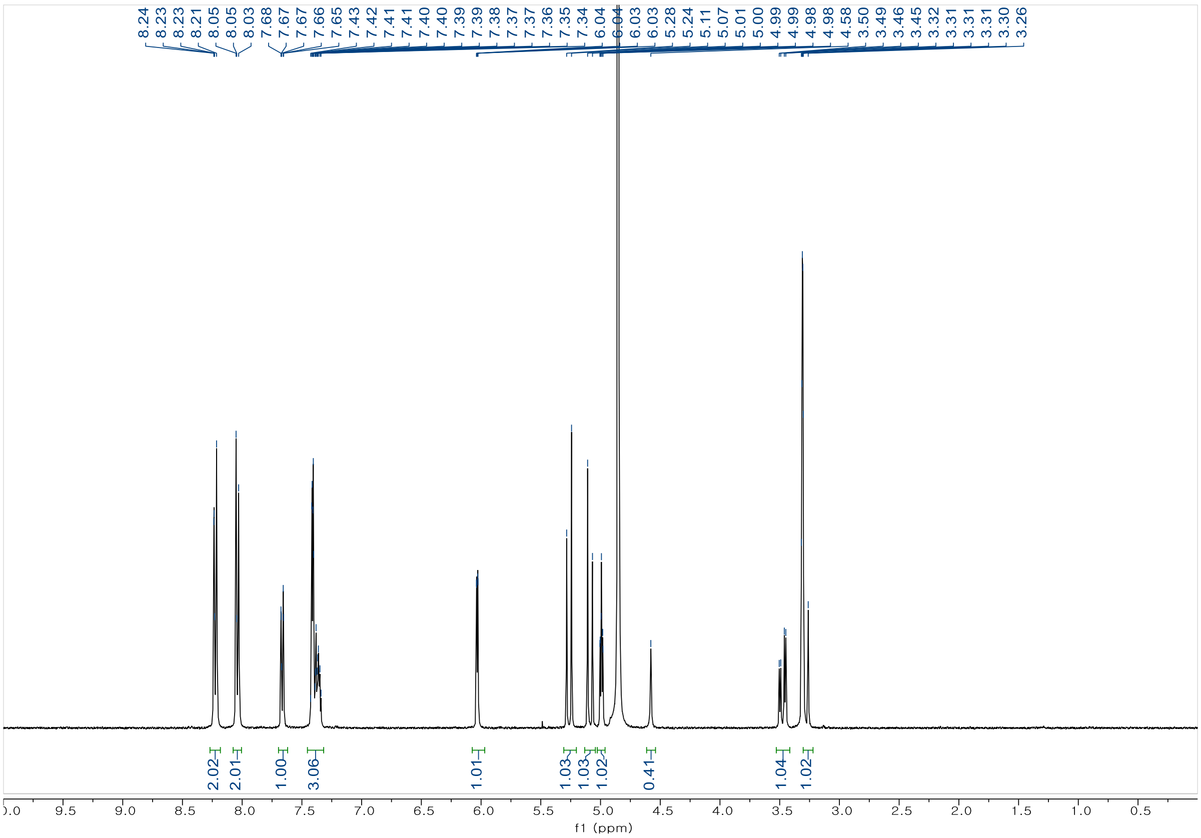

100 MHz, ^{13}C NMR in CD_3OD.

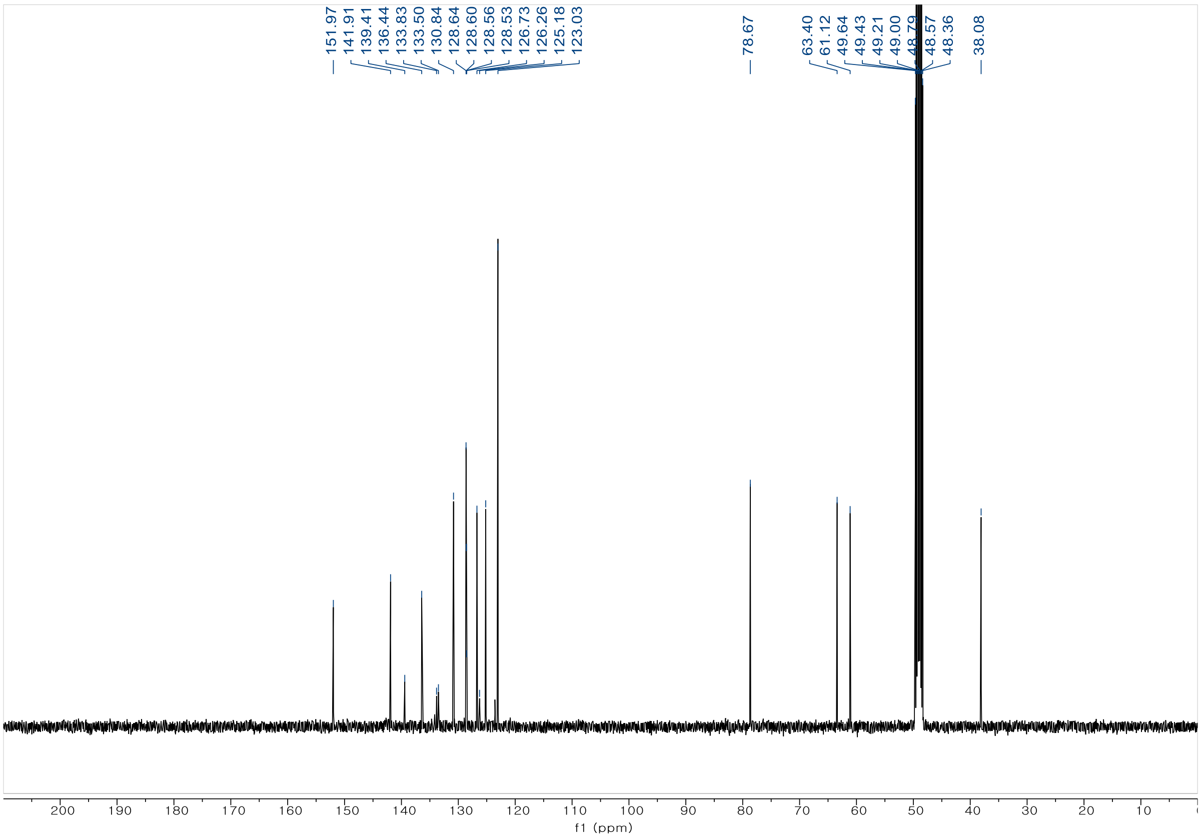

376 MHz, ^{19}F NMR in CD_3OD.

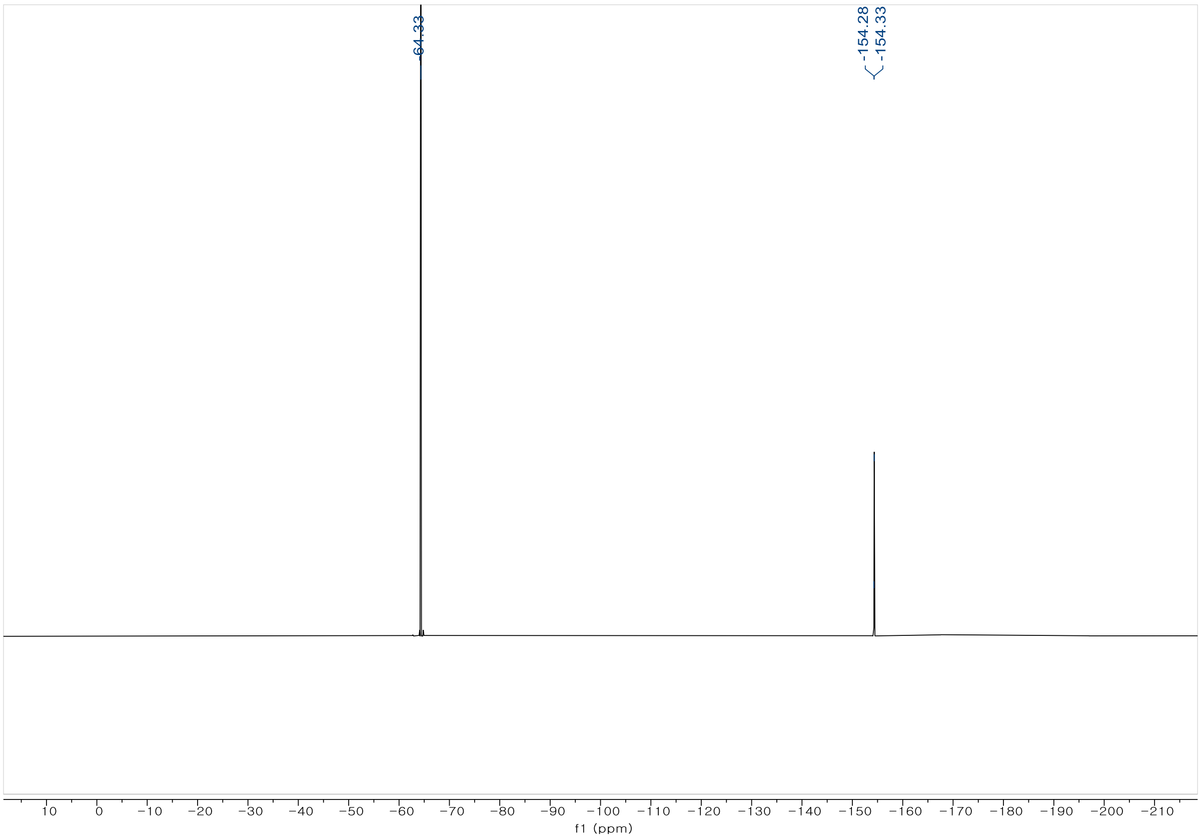

Supplementary Fig. 7. ^{1}H, ^{13}C and ^{19}F NMR of **5d**.

400 MHz, ^{1}H NMR in CDCl$_3$.

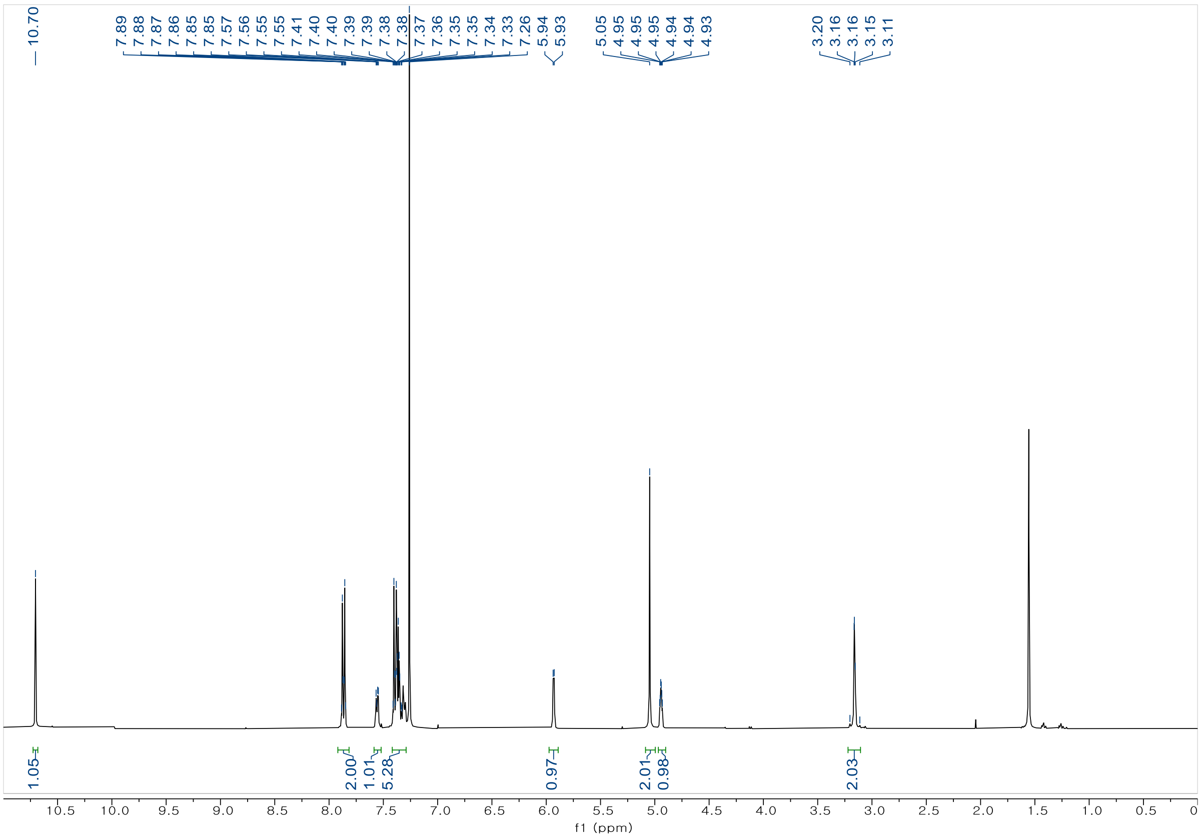

100 MHz, ^{13}C NMR in CDCl$_3$.

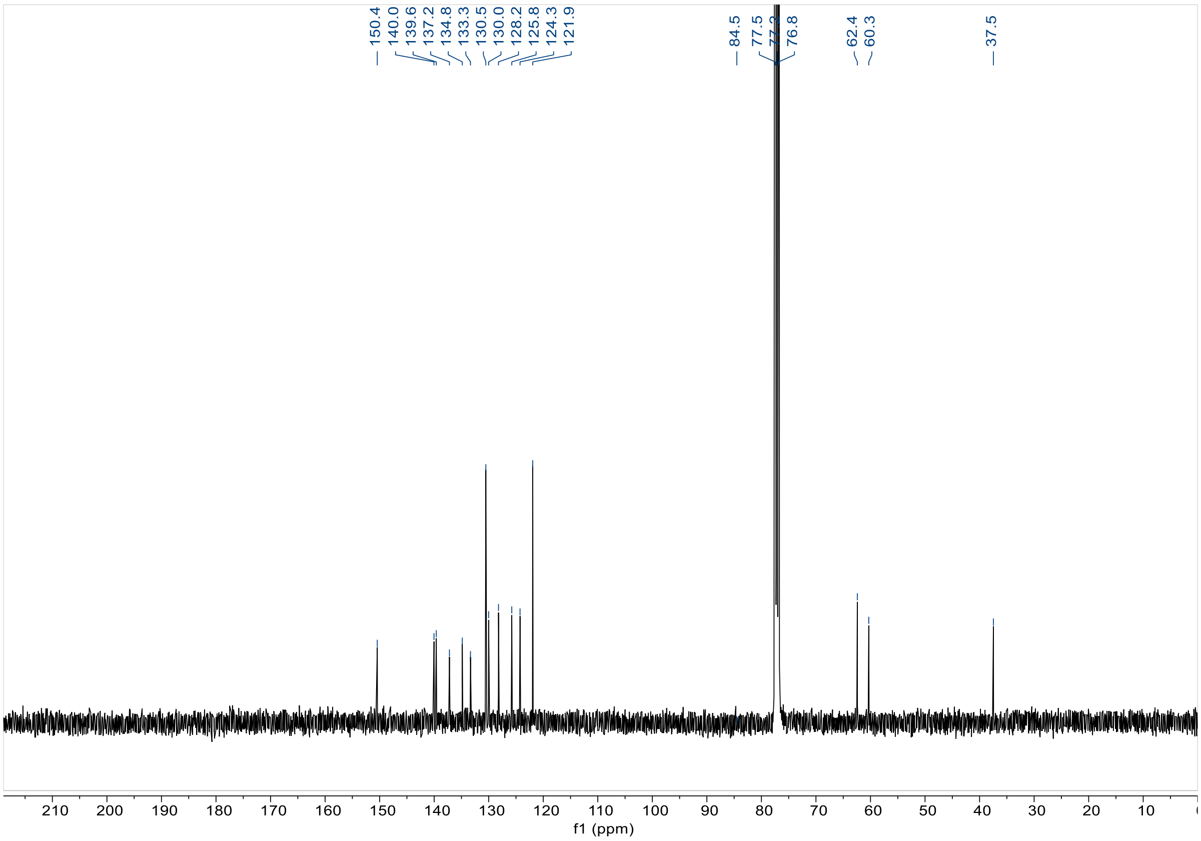

376 MHz, ^{19}F NMR in $CDCl_3$

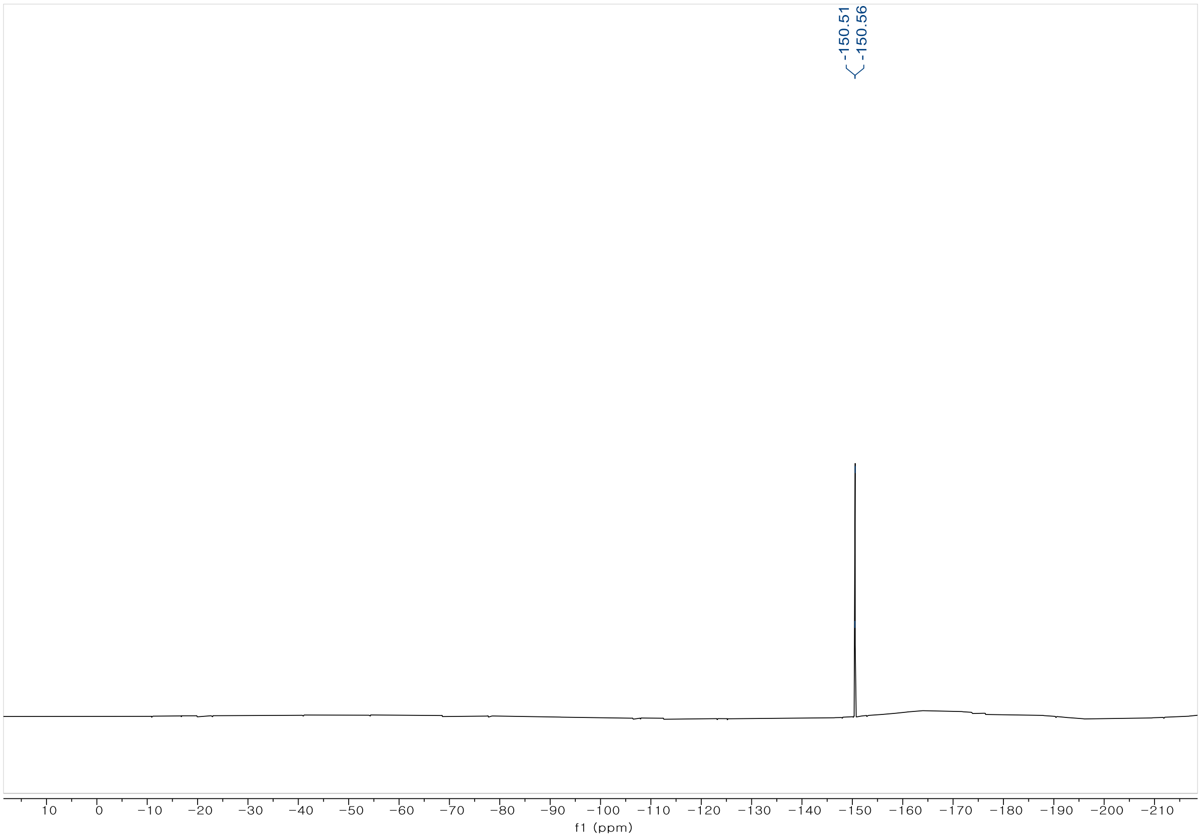

Supplementary Fig. 8. ^{1}H, ^{13}C and ^{19}F NMR of **5f**.

Procedures for the Preparation of NHC-Catalyzed Enantioselective Pyridylation

General procedure for enantioselective pyridylation (GP)

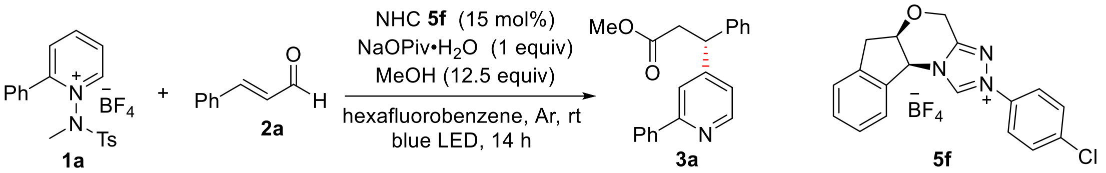

To a 16 mL test tube equipped with a Teflon-coated magnetic bar were added pyridinium salt (0.05 mmol), sodium pivalate hydrate (0.05 mmol), and NHC catalyst (0.0075 mmol). The test tube, anhydrous methanol, hexafluorobenzene degassed by freeze-pump-thaw, and cinnamaldehyde were placed into an argon-filled glovebox. In glovebox, stock solution of cinnamaldehyde (0.1 mmol) and MeOH (12.5 equiv) was prepared with 2.0 mL of hexafluorobenzene. The stock solution was added to the test tube. After adding solvent, the test tube was closed by cap and removed from the glovebox. The reaction mixture was stirred under 440 nm Kessil blue LEDs (10 W, 25% intensity) at room temperature for 14 h. After reaction completion, the mixture was diluted and extracted with dichloromethane three times. The organic layer was dried over sodium sulfate and filtered. The resulting mixture was concentrated under reduced pressure and purified by flash coloum chromatography on silica gel (ethyl acetate : *n*-hexane = 1 : 4 or CH_2Cl_2 : MeOH = 30 : 1) to obtain the desired product **3a** (67%, 10.6 mg).

Optimization Studies

Supplementary Table 1. Initial optimization with racemic NHC catalyst.[a]

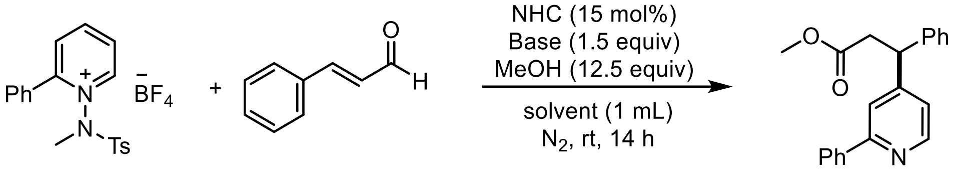

1a **2a** **3a**

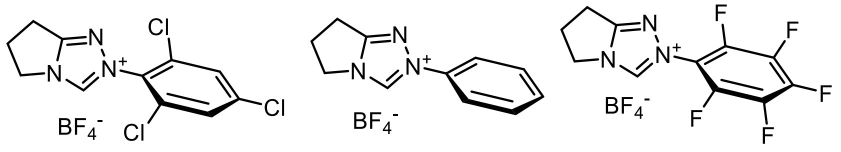

5a **5a'** **5a"**

entry	NHC catalyst	base	solvent	yield [%][b]
1	**5a**	K_2CO_3	Toluene	36
2	**5a′**	K_2CO_3	Toluene	20
3	**5a″**	K_2CO_3	Toluene	34
4	**5a**	K_2CO_3	$CHCl_3$	4
5	**5a**	K_2CO_3	MeOH	12
6	**5a**	K_2CO_3	DMSO	0
7	**5a**	K_2CO_3	DMF	0
8	**5a**	K_3PO_4	Toluene	40
9	**5a**	Na_2CO_3	Toluene	12
10	**5a**	NaOAc	Toluene	55
11	**5a**	KOAc	Toluene	52
12	**5a**	LiOAc	Toluene	5
13	**5a**	NaOMe	Toluene	47
14	**5a**	NaO*t*-Bu	Toluene	0
**15**	**5a**	**NaOPiv•H_2O**	**Toluene**	**64**
16	**5a**	DBU	Toluene	16
17[c]	**5a**	NaOPiv•H_2O	Toluene	51

[a]Reaction conditions: **1a** (0.05 mmol), **2a** (0.1 mmol), NHC catalyst (15 mol%) and base (1.5 equiv), MeOH (12.5 equiv) in solvent irradiated by blue LEDs (440 nm, 10 W) at rt under N_2 for 14 h. [b]Yields were determined by ^{1}H NMR with caffeine as an internal standard. [c]Under dark.

Supplementary Table 2. NHC catalyst optimization.[a]

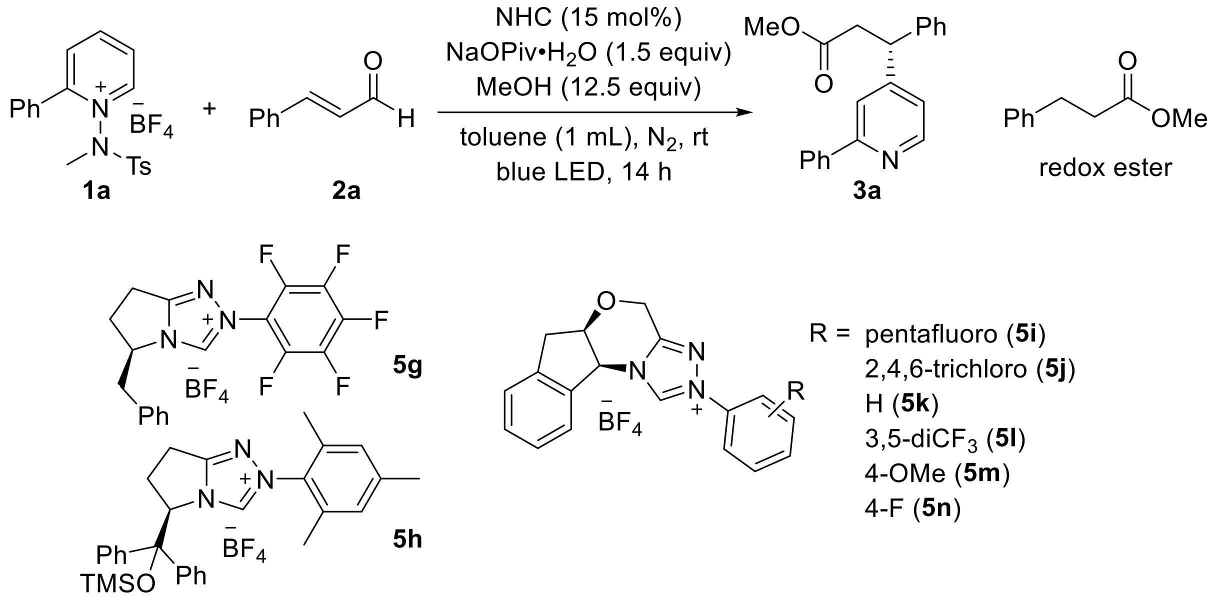

entry	NHC catalyst	yield [%]	er	redox ester [%]
1	**5a**	64	-	44
2	**5b**	47	78:22	37
3	**5c**	46	84:16	< 1
4[b]	**5d**	55	85:15	25
5	**5e**	57	80:20	48
6	**5f**	58	89:11	< 1
7	**5g**	18	43:57	< 1
8	**5h**	< 3	41:59	< 1
9	**5i**	61	55:45	23
10	**5j**	53	75:25	30
11	**5k**	52	89:11	14
12	**5l**	51	79:21	10
13	**5m**	55	85:15	3
14	**5n**	50	89:11	21

[a]Reaction conditions: **1a** (0.05 mmol), **2a** (0.1 mmol), NHC catalyst (15 mol%) and NaOPiv•H_2O (1.5 equiv), MeOH (12.5 equiv) in toluene irradiated by blue LEDs (440 nm, 10 W) at rt under N_2 for 14 h. Yields were determined by ^1H NMR with caffeine as an internal standard. [b]NaOAc (1.5 equiv) was used as base.

NHC catalysts used in the optimization studies were either commercially available or synthesized through the references.[10] All ^1H and ^{13}C NMR data were matched with the references.

Supplementary Table 3. Solvent optimization.[a]

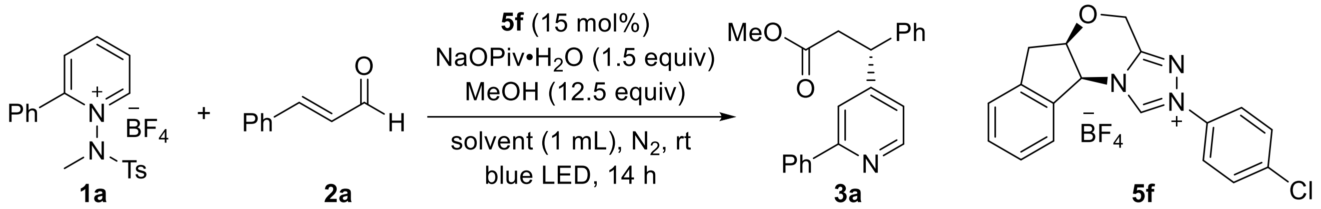

entry	solvent	yield [%][b]	er
1	*p*-xylene	54	87:13
2	m-xylene	47	86:14
3	o-xylene	49	85:15
4	benzene	56	83:17
5	ethylbenzene	55	86:14
6	PhCl	39	80:20
7	$PhCF_3$	46	81:19
8	Hex	39	75:25
9	dioxane	trace	-
10	$CHCl_3$	10	77:23
11[c]	THF	trace	-
12	1,2-DCE	7	72:28
13	MeCN	trace	-
14	DMF	n.d.	-
15	DMSO	n.d.	-
16	DMAc	n.d.	-
17	fluorobenzene	28	80:20
18	1,4-difluorobenzene	35	85:15
19	1,2,4,5-tetrafluorobenzene	39	87:13
**20**	**hexafluorobenzene**	**40**	**94:6**
21	hexafluorobenzene:$CHCl_3$ = 5:1	52	90:10

[a]Reaction conditions: **1a** (0.05 mmol), **2a** (0.1 mmol), NHC catalyst (15 mol%) and NaOPiv•H_2O (1.5 equiv), MeOH (12.5 equiv) in toluene irradiated by blue LEDs (440 nm, 10 W) at rt under N_2 for 14 h. [b]Yields were determined by ^1H NMR with caffeine as an internal standard. [c]Side product, which THF radical is inserted to C4 position of pyridinium salt, was formed in 40% yield.

Supplementary Table 4. Additional optimization.[a]

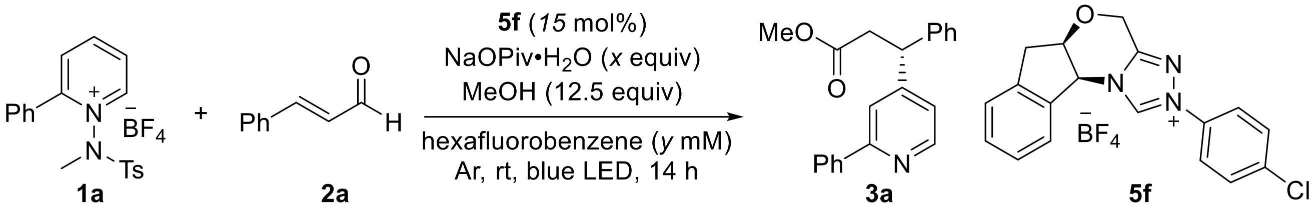

entry	x	y	yield [%][b]	er
1	2.0	50	46	94:6
2	1.5	50	40	94:6
3	1.0	50	69	94:6
4	0.5	50	31	94:6
**5**	**1.0**	**25**	**67**	**96:4**
6	1.0	12	40	97:3
7	1.0	6	11	95:5
8	1.0	100	70	92:8
9[c,e]	1.0	25	63	95:5
10[d,e]	1.0	25	52	95:5

[a]Reaction conditions: **1a** (0.05 mmol), **2a** (0.1 mmol), NHC catalyst (15 mol%) and NaOPiv•H_2O (*x* equiv), MeOH (12.5 equiv) in hexafluorobenzene (*y* mM) irradiated by blue LEDs (440 nm, 10 W) at rt under Ar for 14 h. [b]Yields were determined by ^1H NMR with caffeine as an internal standard. [c]At 15 °C. [d]At 5 °C. [e]Irradiated by blue LEDs (440 nm, 20 W)

Mechanistic Investigation

1. Control experiments

Supplementary Table 5. Control experiments.[a]

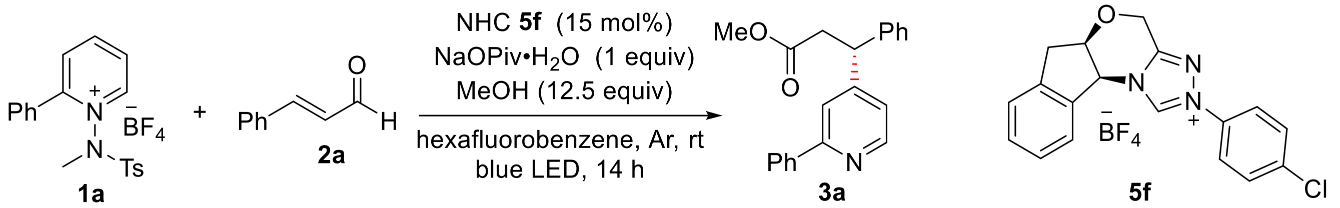

entry	deviation from standard condition	yield [%][b]	er
1	without NHC **5f**	n.d.	-
2	without base	n.d.	-
3[c]	under O_2	n.d.	-
4	with H_2O 0.1 mL	35%	93:7
5[d]	with TEMPO (3 equiv.)	n.d.	-
6	with Galvinoxyl (5 equiv.)	n.d.	-

[a]Reaction conditions: **1a** (0.05 mmol), **2a** (0.1 mmol), **5f** (15 mol%) and NaOPiv•H_2O (1 equiv), MeOH (12.5 equiv) in hexafluorobenzene irradiated by blue LEDs (440 nm, 10 W) at rt under Ar for 14 h. [b]Yields were determined by ^1H NMR with caffeine as an internal standard. [c]Solvent bubbled with O_2 during 10 min was used. [d]1.5 equiv of TEMPO ester[11] was observed, and TEMPO−amidyl radical adduct was observed by HRMS analysis.

2. Kinetic isotope experiment

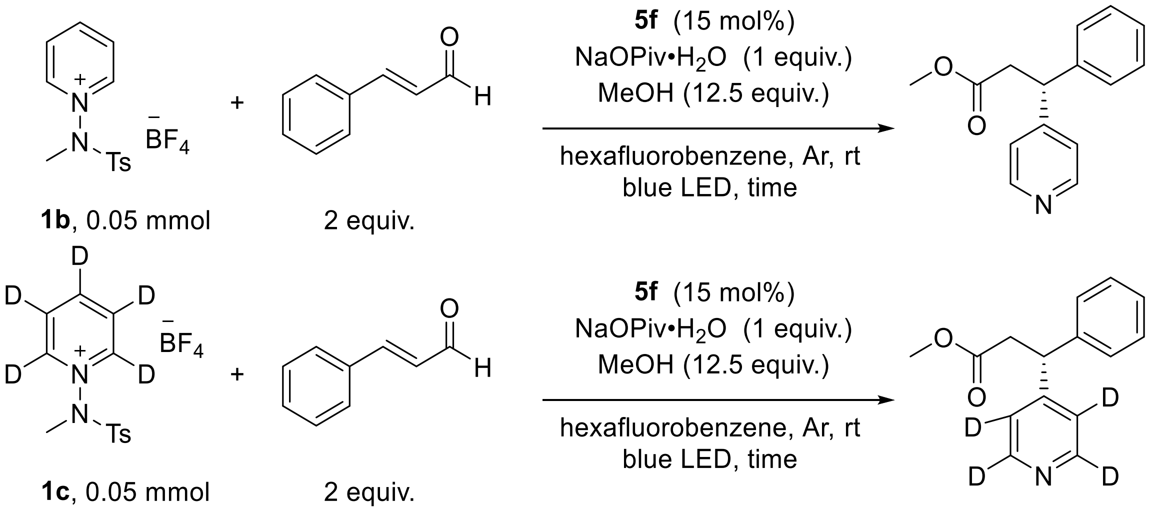

Supplementary Fig. 9. Measurement of primary kinetic isotope effect (two parallel reactions).

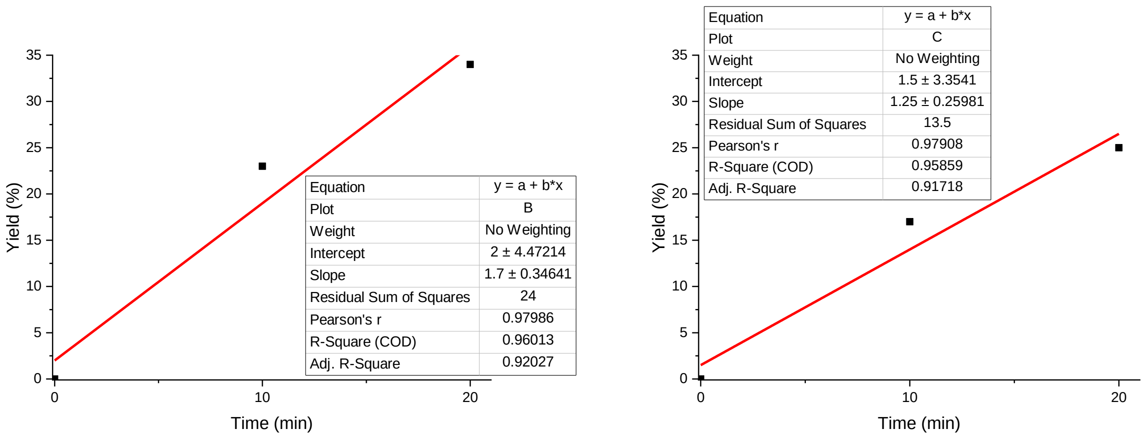

$k_H/k_D = 1.70/1.25 = 1.36$

Supplementary Fig. 10. Estimation of rate constant. Reaction with **1b** (top) and **1c** (bottom).

3. Radical clock experiment

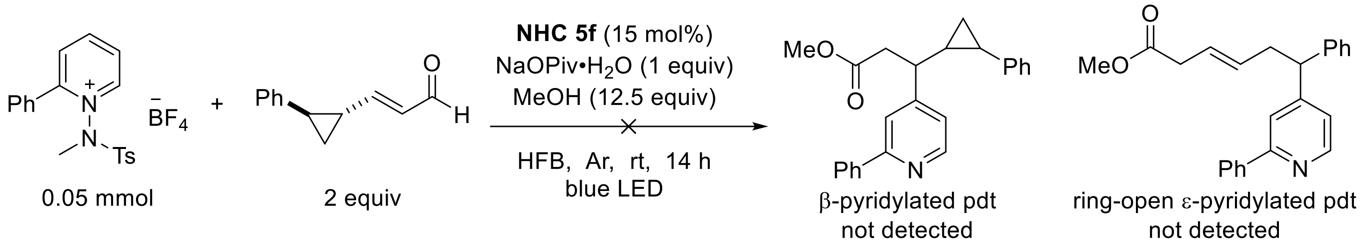

We have investigated radical-clock experiments using β-cyclopropyl enal. However, we were not able to obtain any meaningful yields of β-pyridylation products under the standard reaction conditions. Previous radical-clock experiments[12] using cyclopropyl-substituted enals showed that isomerization or ring-opening of the cyclopropyl unit was not observed probably because the radical spin is more likely delocalized between the enal formal carbonyl carbon and the triazolium NHC unit.

4. Absorption spectra

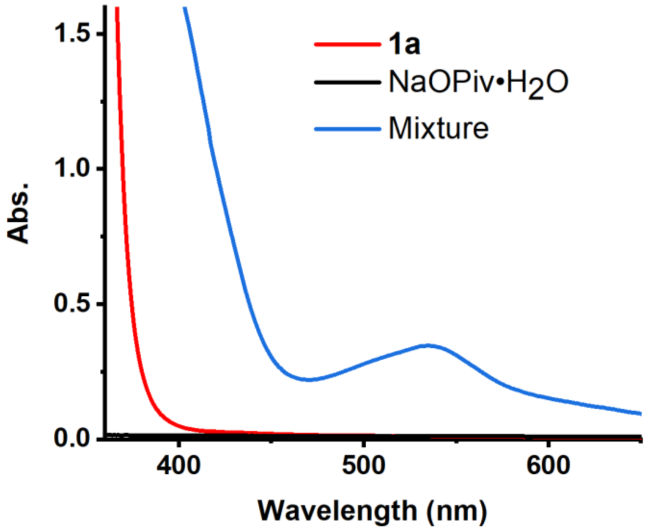

Supplementary Fig. 11. Apsorption data of **1a**, NaOPiv•H_2O, and 1:1 mixture of **1a** and NaOPiv•H_2O (0.025 M in $CHCl_3$).

Due to the low solubility of base in hexafluorobenzene and other nonpolar aromatic solvents, the absorption spectra experiments were conducted in $CHCl_3$.

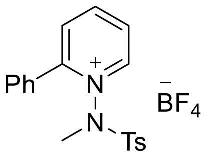

1a

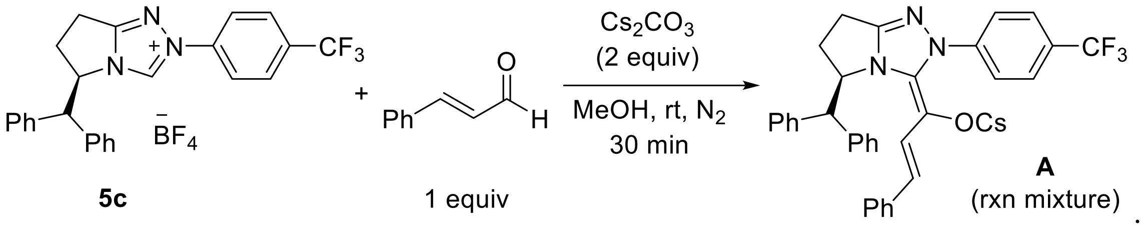

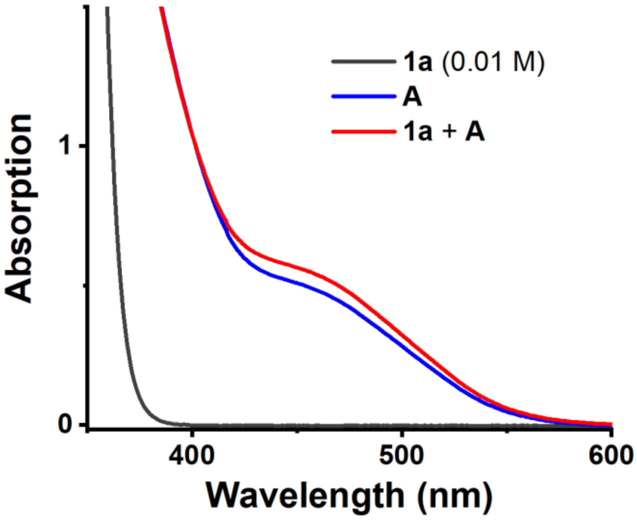

Supplementary Fig. 12. Apsorption data of **1a**, homoenolate form of Breslow intermediate **A** (reaction mixture), and 1:1 mixture of **1a** and **A** (0.01 M in MeOH).

Due to the low solubility of base and NHC catalyst in hexafluorobenzene and other nonpolar aromatic solvents, the absorption spectra experiments were conducted in MeOH. Also, we used carbonate base (Cs_2CO_3), instead of carboxylate base (NaOPiv), to avoid the formation of EDA complex between salt and base.

Compound Characterization

Methyl (*R*)-3-phenyl-3-(2-phenylpyridin-4-yl)propanoate (3a)

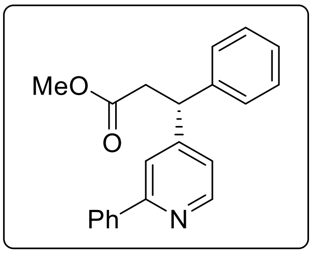

Prepared according to **GP**. Desired product **3a** was obtained as colorless oil (10.6 mg, 67% isolated yield). **^{1}H NMR** (400 MHz, $CDCl_3$) δ 8.59 (dd, *J* = 5.1, 0.8 Hz, 1H), 7.97 – 7.89 (m, 2H), 7.59 (d, *J* = 1.8 Hz, 1H), 7.48 – 7.29 (m, 6H), 7.26 – 7.21 (m, 2H), 7.11 (dd, *J* = 5.1, 1.7 Hz, 1H), 4.61 (t, *J* = 7.9 Hz, 1H), 3.61 (s, 3H), 3.19 – 3.05 (m, 2H). **^{13}C NMR** (100 MHz, $CDCl_3$) δ 171.9, 158.0, 153.1, 150.0, 141.9, 139.5, 129.1, 129.0, 128.9, 127.8, 127.3, 127.1, 121.5, 120.1, 52.0, 46.7, 39.9. **Specific Rotation** $[\alpha]_D^{20}$ +4.4(*c* 1.0, $CHCl_3$). **HRMS** (ESI$^+$) m/z calcd. For $C_{21}H_{20}NO_2^+$ $[M+H]^+$: 318.1489, found 318.1493. **HPLC Analysis**. CHIRALCEL OD-H, 25 °C; *n*-hexane:*i*-PrOH = 85:15, 1.0 mL/min, 254 nm, t_{R1} (major) = 10.22 min, t_{R2} (minor) = 22.70 min, 96:4 er.

The absolute stereochemistry was assigned by analogy to compound **3x** and **4k**.

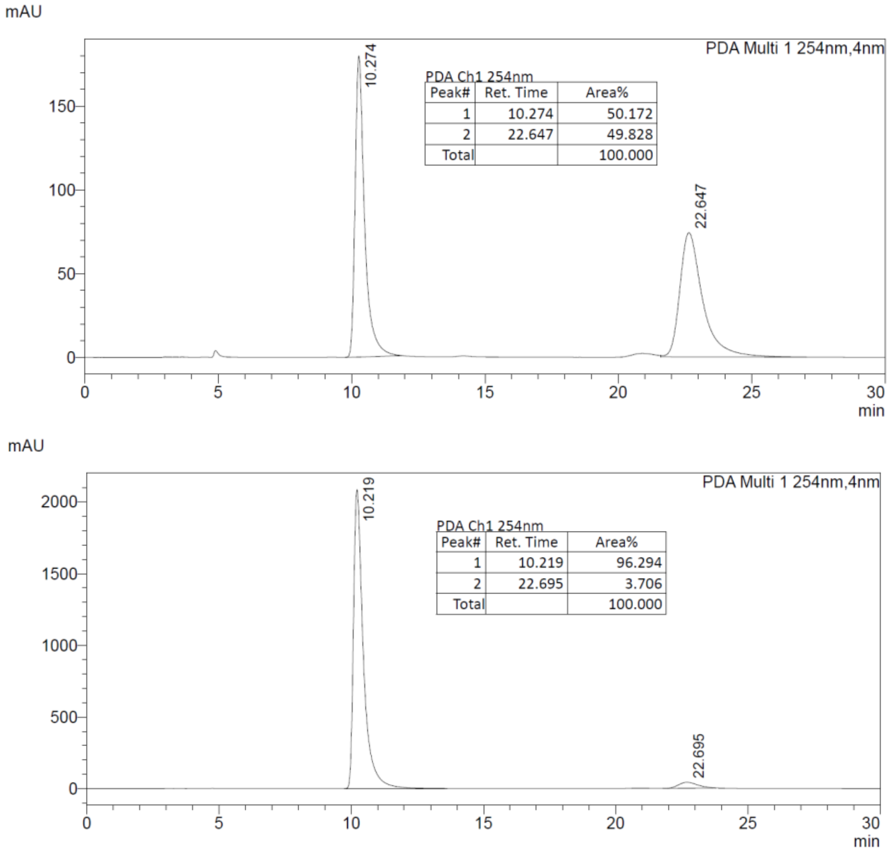

Supplementary Fig. 13. HPLC traces of *rac*-**3a** (top) and enantioenriched-**3a** (bottom).

400 MHz, ^{1}H NMR in $CDCl_3$

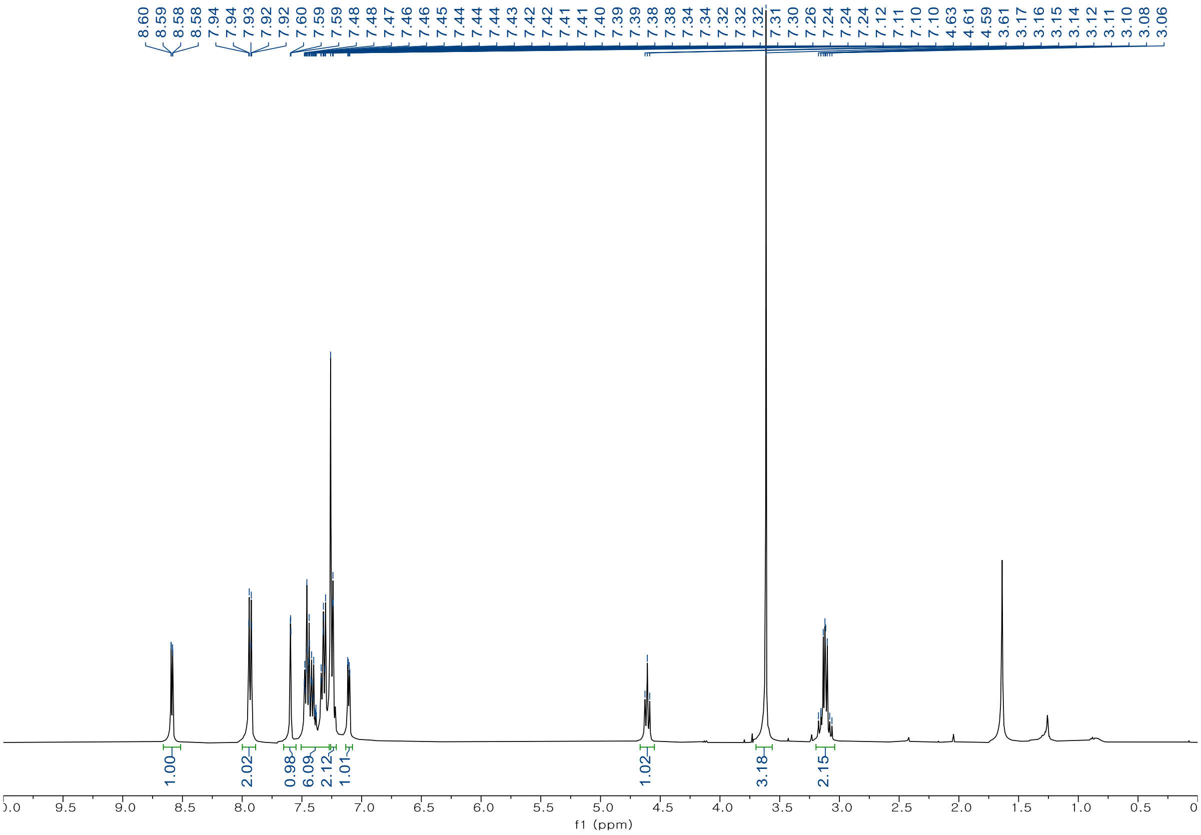

100 MHz, ^{13}C NMR in $CDCl_3$

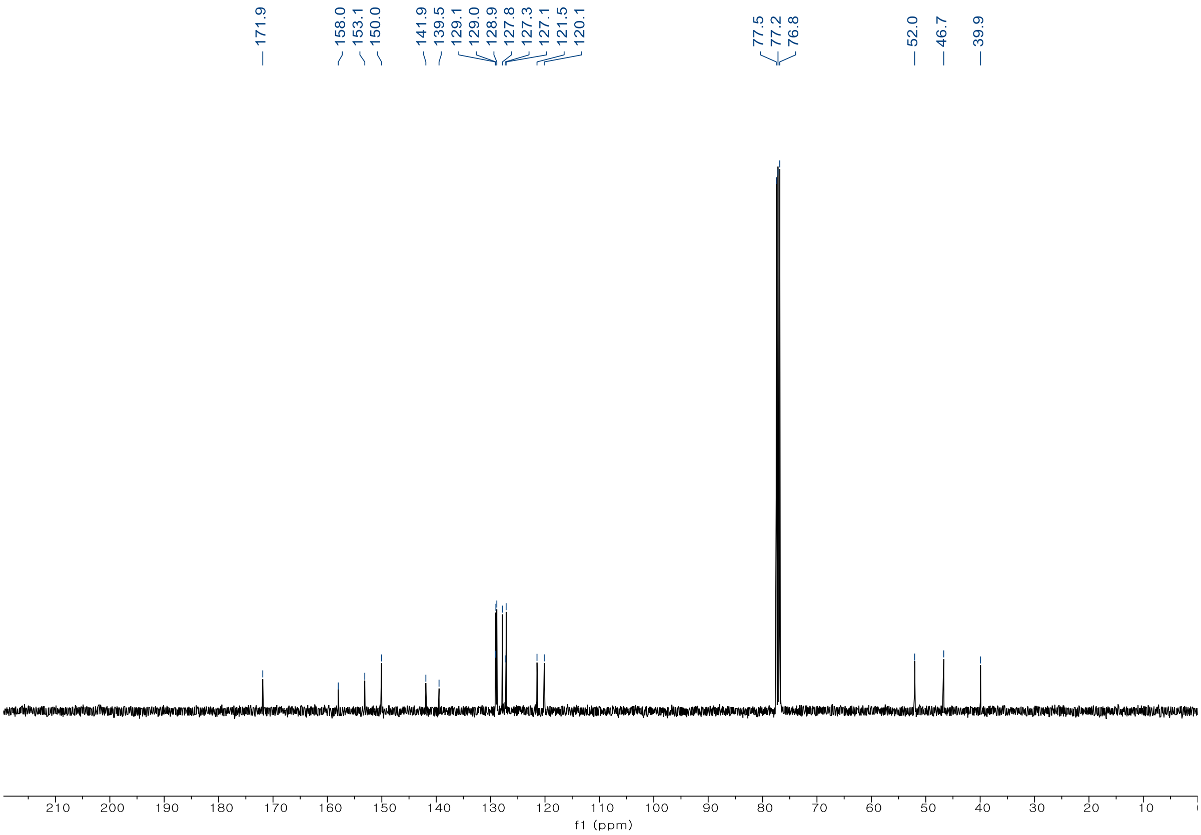

Supplementary Fig. 14. ^{1}H and ^{13}C NMR of **3a**.

Methyl (*R*)-3-(2-phenylpyridin-4-yl)-3-(*p*-tolyl)propanoate (3b)

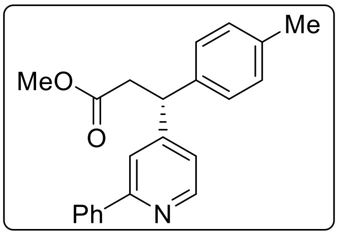

Prepared according to **GP**. Desired product **3b** was obtained as colorless oil (9.6 mg, 58% isolated yield). ^{1}**H NMR** (400 MHz, $CDCl_3$) δ 8.58 (d, *J* = 5.1 Hz, 1H), 7.97 – 7.89 (m, 2H), 7.61 – 7.56 (m, 1H), 7.53 – 7.35 (m, 3H), 7.19 – 7.07 (m, 5H), 4.56 (t, *J* = 7.9 Hz, 1H), 3.61 (s, 3H), 3.17 – 3.02 (m, 2H), 2.31 (s, 3H). ^{13}**C NMR** (100 MHz, $CDCl_3$) δ 172.0, 158.0, 153.4, 150.0, 139.5, 138.9, 137.0, 129.7, 129.1, 128.8, 127.7, 127.2, 121.4, 120.1, 52.0, 46.3, 40.0, 21.1. **HRMS** (ESI^+) m/z calcd. For $C_{22}H_{22}NO_2^+$ $[M+H]^+$: 332.1651, found 332.1650. **Specific Rotation** $[\alpha]_D^{26}$ –2.5 (*c* 0.95, $CHCl_3$). **HPLC Analysis**. CHIRALCEL OD-H, 25 °C; *n*-hexane:*i*-PrOH = 75:25, 1.0 mL/min, 254 nm, t_{R1} (major) = 6.89 min, t_{R2} (minor) = 11.66 min, 96:4 er.

The absolute stereochemistry was assigned by analogy to compound **3x** and **4k**.

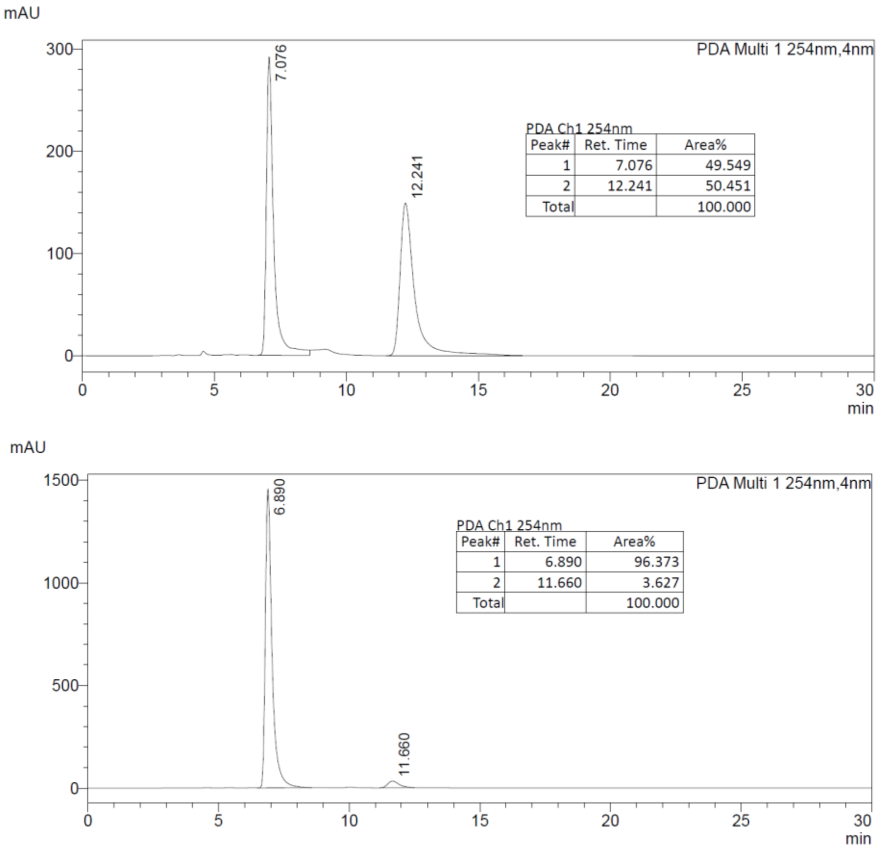

Supplementary Fig. 15. HPLC traces of *rac*-**3b** (top) and enantioenriched-**3b** (bottom).

400 MHz, ^{1}H NMR in $CDCl_3$.

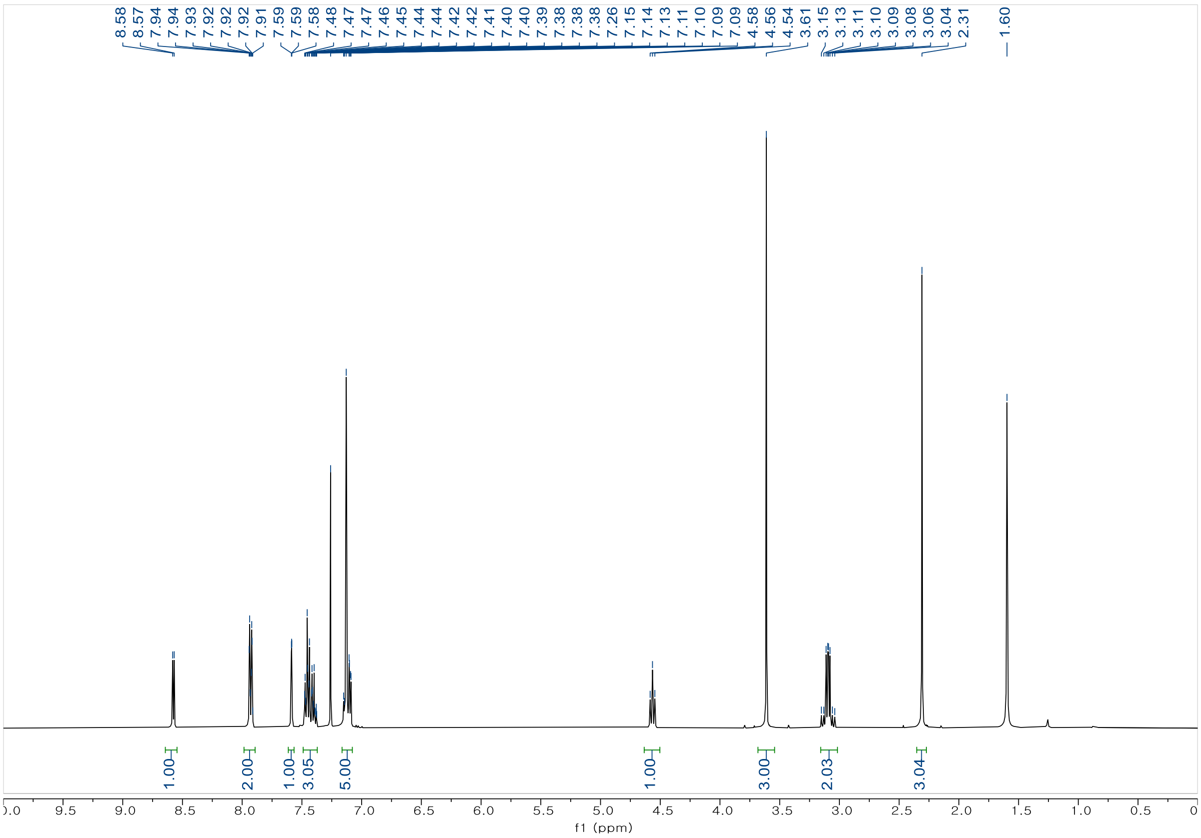

100 MHz, ^{13}C NMR in $CDCl_3$.

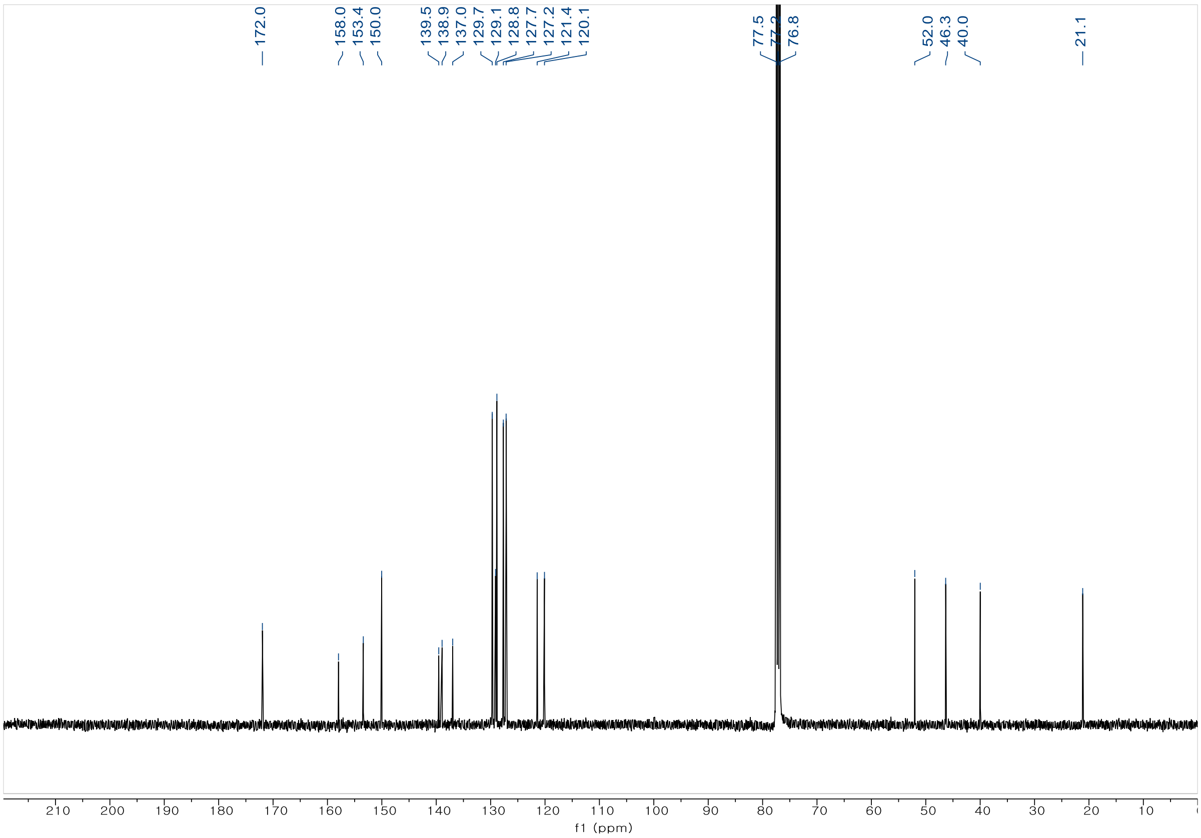

Supplementary Fig. 16. ^{1}H and ^{13}C NMR of **3b**.

Methyl (*R*)-3-(4-methoxyphenyl)-3-(2-phenylpyridin-4-yl)propanoate (3c)

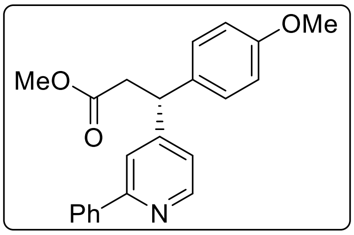

Prepared according to **GP**. Desired product **3c** was obtained as colorless oil (11.2 mg, 65% isolated yield). **^{1}H NMR** (400 MHz, $CDCl_3$) δ 8.58 (dd, *J* = 5.1, 0.8 Hz, 1H), 7.97 – 7.89 (m, 2H), 7.60 – 7.55 (m, 1H), 7.48 – 7.43 (m, 2H), 7.42 – 7.37 (m, 1H), 7.21 – 7.12 (m, 2H), 7.12 – 7.06 (m, 1H), 6.89 – 6.81 (m, 2H), 4.56 (t, *J* = 7.9 Hz, 1H), 3.78 (s, 3H), 3.61 (s, 3H), 3.16 – 3.00 (m, 2H). **^{13}C NMR** (100 MHz, $CDCl_3$) δ171.9, 158.7, 158.0, 153.5, 150.0, 139.5, 134.0, 129.1, 128.8, 128.8, 127.1, 121.4, 120.0, 114.4, 55.4, 52.0, 45.9, 40.1. **HRMS** (ESI^+) m/z calcd. For $C_{22}H_{22}NO_3^+$ $[M+H]^+$: 348.1600, found 348.1599. **Specific Rotation** $[\alpha]_D^{26}$ +1.4 (*c* 1.0, $CHCl_3$). **HPLC Analysis**. CHIRALCEL OD-H, 25 ºC; *n*-hexane:*i*-PrOH = 75:25, 1.0 mL/min, 254 nm, t_{R1} (major) = 9.30 min, t_{R2} (minor) = 15.85 min, 96:4 er.

The absolute stereochemistry was assigned by analogy to compound **3x** and **4k**.

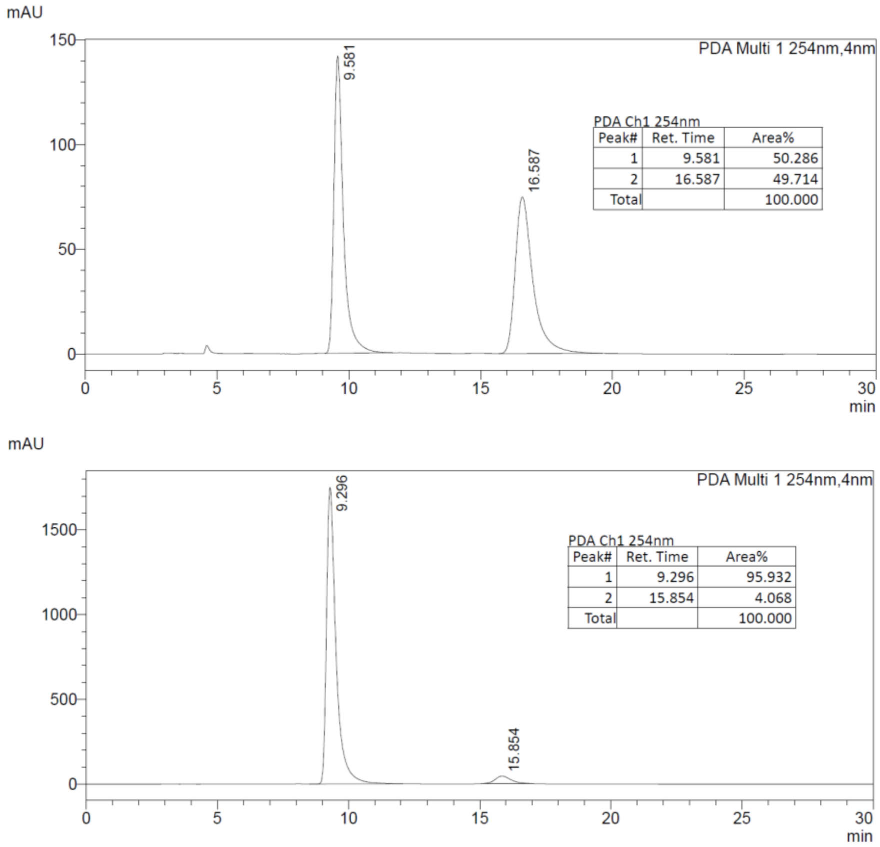

Supplementary Fig. 17. HPLC traces of *rac*-**3c** (top) and enantioenriched-**3c** (bottom).

400 MHz, ^{1}H NMR in $CDCl_3$.

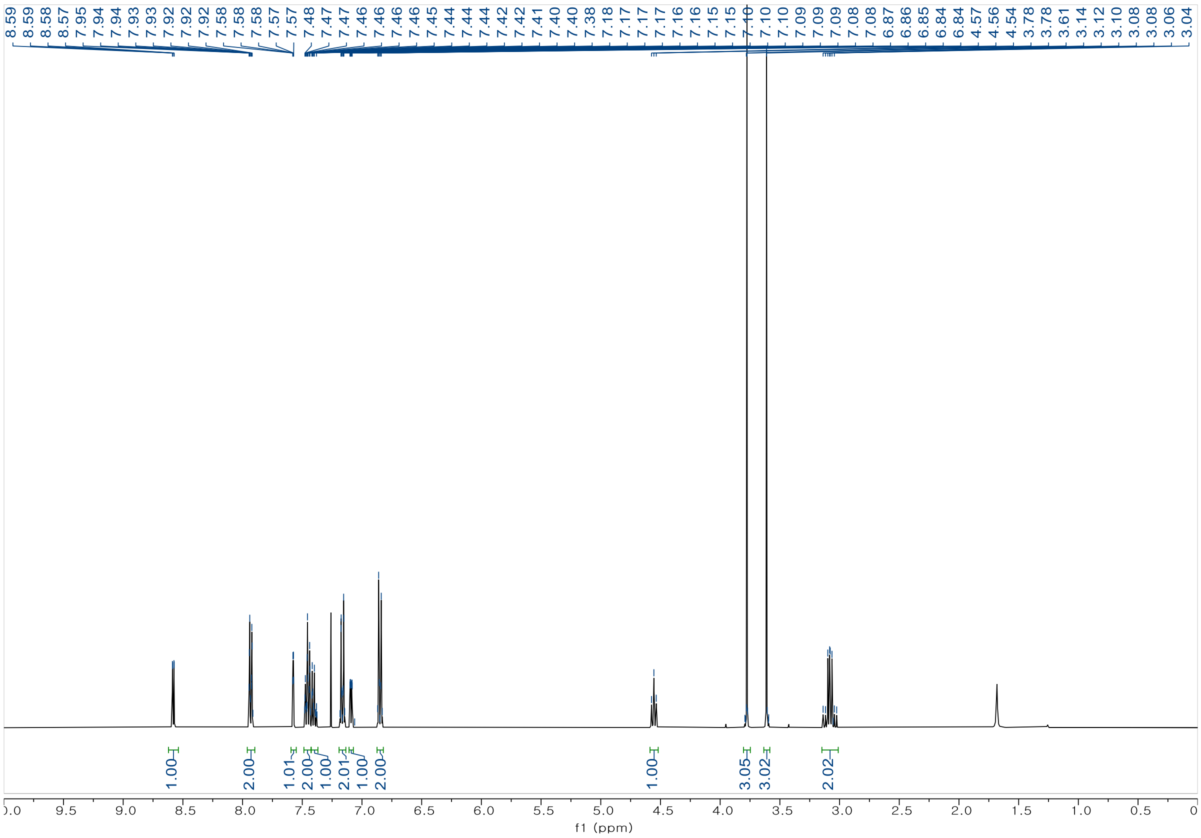

100 MHz, ^{13}C NMR in $CDCl_3$.

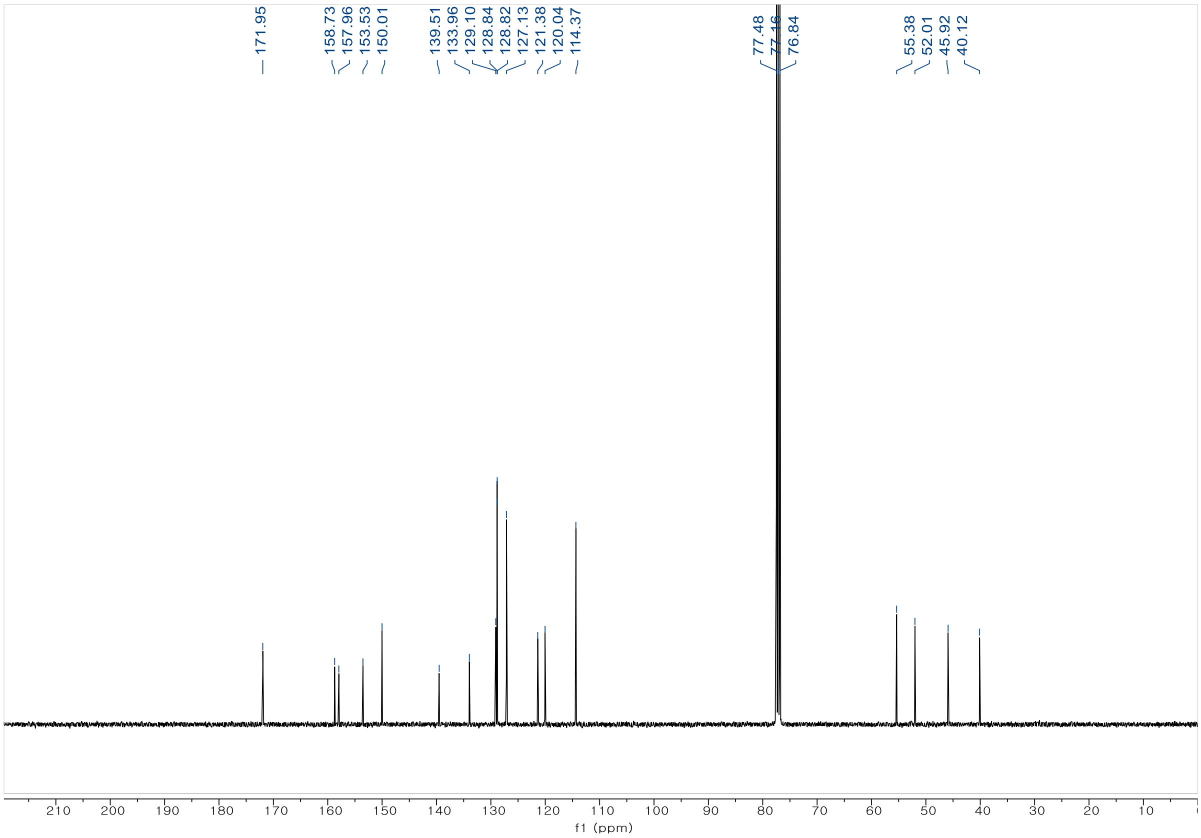

Supplementary Fig. 18. ^{1}H and ^{13}C NMR of **3c**.

Methyl (*R*)-3-([1,1'-biphenyl]-4-yl)-3-(2-phenylpyridin-4-yl)propanoate (3d)

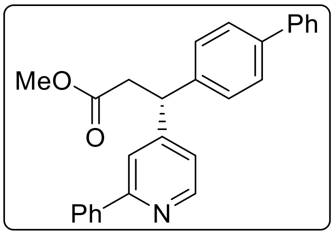

Prepared according to **GP**. Desired product **3d** was obtained as white solid (10.1 mg, 51% isolated yield). **^{1}H NMR** (400 MHz, $CDCl_3$) δ 8.62 (dd, *J* = 5.2, 0.8 Hz, 1H), 7.98 – 7.91 (m, 2H), 7.66 – 7.61 (m, 1H), 7.59 – 7.51 (m, 4H), 7.49 – 7.38 (m, 5H), 7.36 – 7.30 (m, 3H), 7.15 (dd, *J* = 5.2, 1.7 Hz, 1H), 4.66 (t, *J* = 7.9 Hz, 1H), 3.64 (s, 3H), 3.22 – 3.10 (m, 2H). **^{13}C NMR** (100 MHz, $CDCl_3$) δ 171.9, 158.0, 153.1, 150.1, 140.9, 140.6, 140.2, 139.5, 129.2, 128.9, 128.9, 128.2, 127.7, 127.5, 127.1, 127.1, 121.5, 120.1, 52.1, 46.4, 39.9. **HRMS** (ESI^+) m/z calcd. For $C_{27}H_{24}NO_2^+$ $[M+H]^+$: 394.1807, found 394.1809. **Specific Rotation** $[\alpha]_D^{26}$ +10.9 (*c* 1.0, $CHCl_3$). **HPLC Analysis**. CHIRALCEL OD-H, 25 °C; *n*-hexane:*i*-PrOH = 75:25, 1.0 mL/min, 254 nm, t_{R1} (major) = 13.84 min, t_{R2} (minor) = 20.58 min, 90:10 er.

The absolute stereochemistry was assigned by analogy to compound **3x** and **4k**.

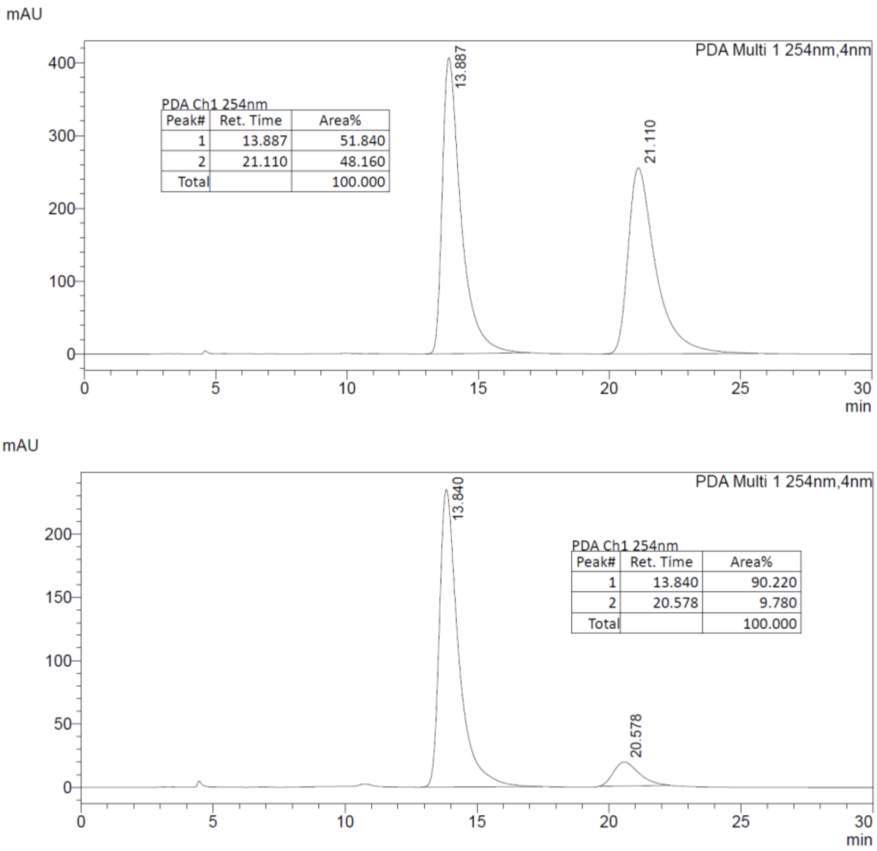

Supplementary Fig. 19. HPLC traces of *rac*-**3d** (top) and enantioenriched-**3d** (bottom).

400 MHz, ^{1}H NMR in $CDCl_3$.

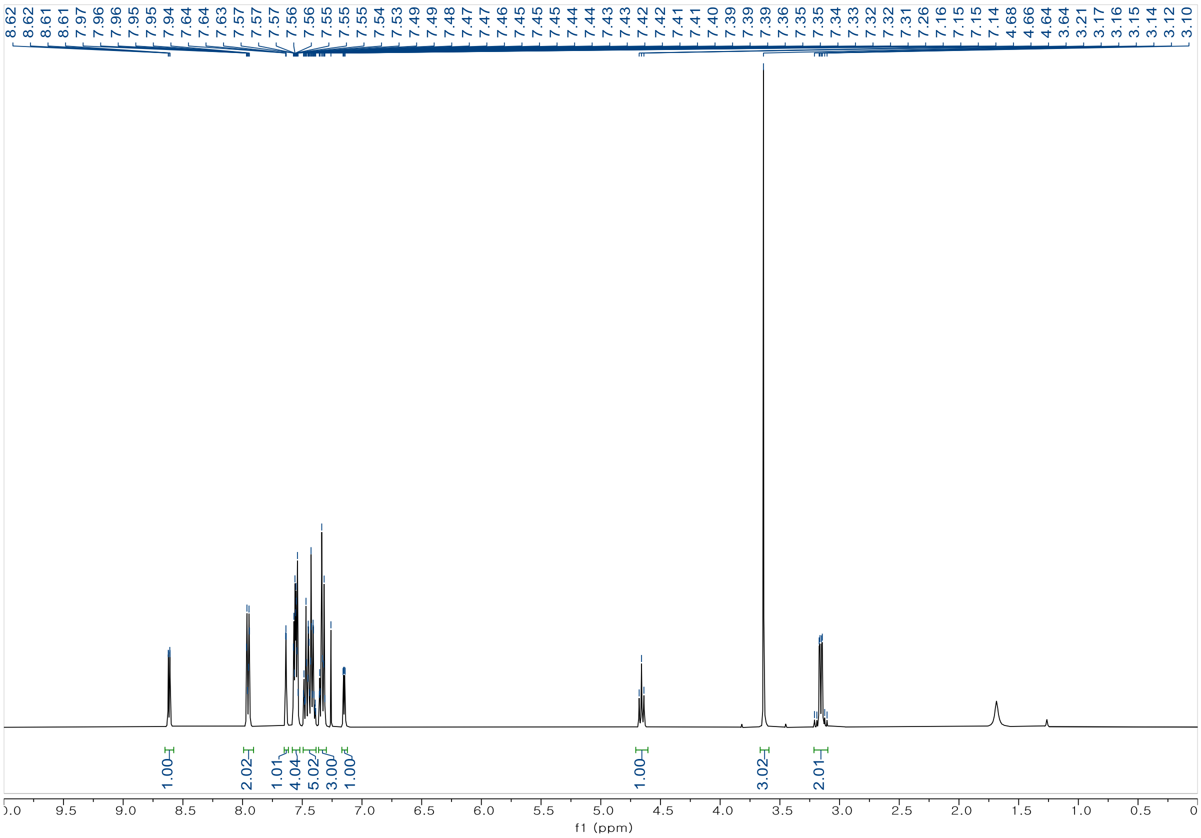

100 MHz, ^{13}C NMR in $CDCl_3$.

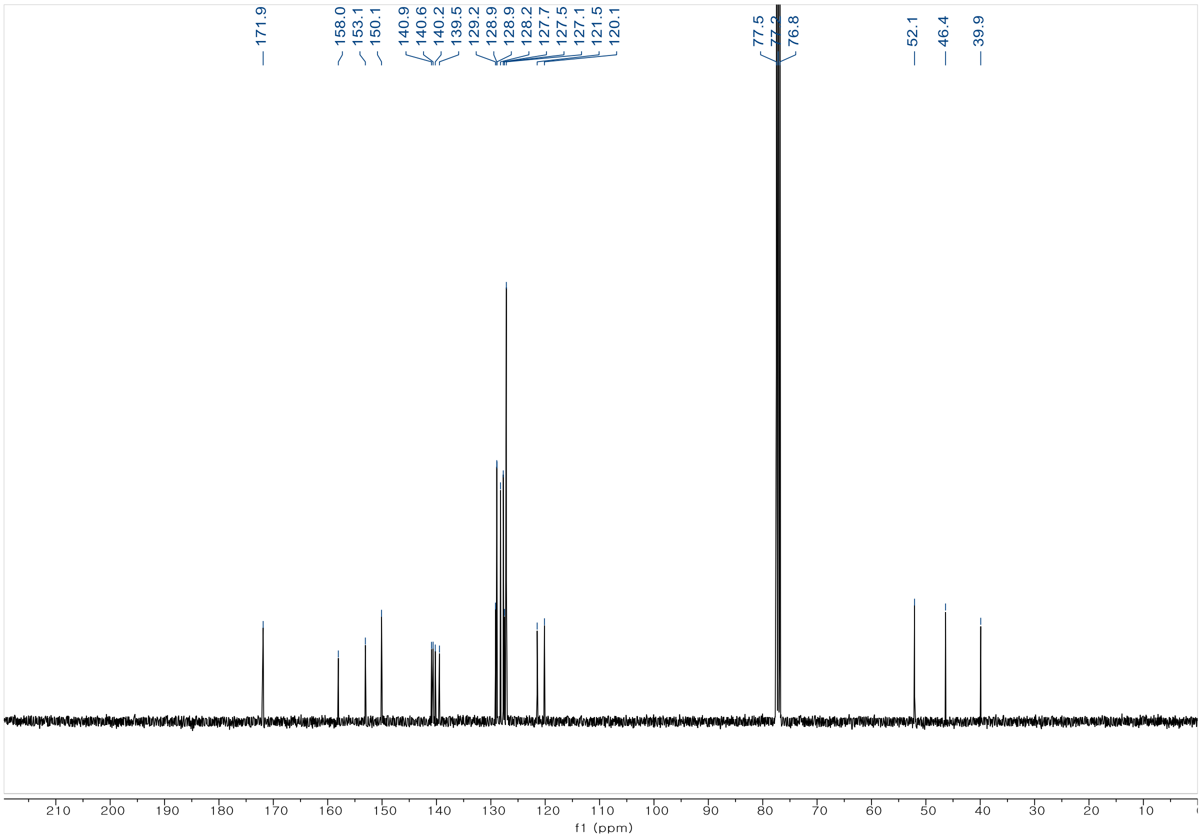

Supplementary Fig. 20. ^{1}H and ^{13}C NMR of **3d**.

Methyl (*R*)-3-(2-phenylpyridin-4-yl)-3-(4-(trifluoromethyl)phenyl)propanoate (3e)

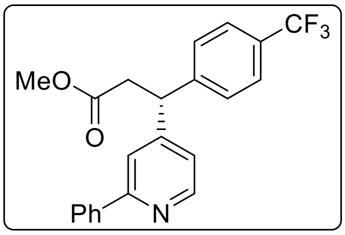

Prepared according to **GP**. Desired product **3e** was obtained as white solid (11.5 mg, 60% isolated yield). **^{1}H NMR** (400 MHz, $CDCl_3$) δ 8.61 (dd, *J* = 5.1, 0.8 Hz, 1H), 7.97 – 7.88 (m, 2H), 7.61 – 7.55 (m, 3H), 7.50 – 7.40 (m, 3H), 7.40 – 7.35 (m, 2H), 7.10 – 7.06 (m, 1H), 4.67 (t, *J* = 7.9 Hz, 1H), 3.63 (s, 3H), 3.21 – 3.05 (m, 2H). **^{13}C NMR** (100 MHz, $CDCl_3$) δ 171.4, 158.2, 152.1, 150.2, 145.9, 139.2, 129.7 (q, J = 32.4 Hz), 129.3, 128.9, 128.3, 127.1, 126.0 (q, *J* = 3.8 Hz), 124.1 (q, *J* = 272.2 Hz), 121.3, 120.0, 52.2, 46.4, 39.6. **^{19}F NMR** (376 MHz, $CDCl_3$) δ -62.58. **HRMS** (ESI^+) m/z calcd. For $C_{22}H_{19}F_3NO_2^+$ $[M+H]^+$: 386.1368, found 386.1368. **Specific Rotation** $[\alpha]_D^{26}$ –2.3 (*c* 1.0, $CHCl_3$). **HPLC Analysis**. CHIRALCEL OD-H, 25 °C; *n*-hexane:*i*-PrOH = 75:25, 1.0 mL/min, 254 nm, t_{R1} (major) = 7.42 min, t_{R2} (minor) = 15.25 min, 90:10 er.

The absolute stereochemistry was assigned by analogy to compound **3x** and **4k**.

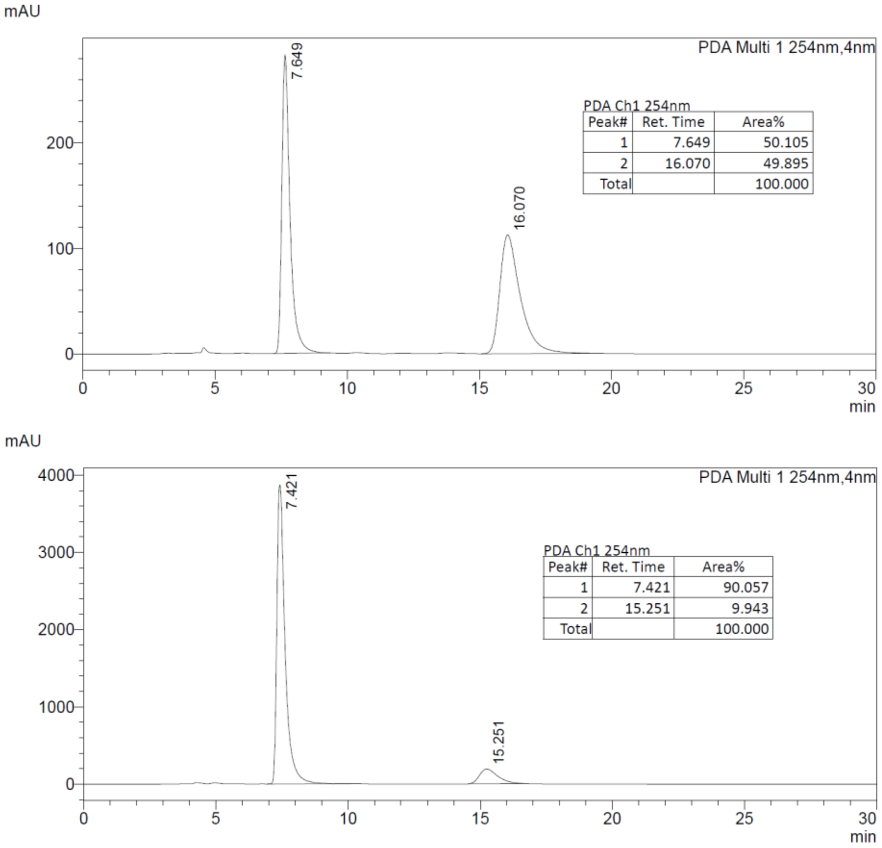

Supplementary Fig. 21. HPLC traces of *rac*-**3e** (top) and enantioenriched-**3e** (bottom).

400 MHz, ^{1}H NMR in CDCl$_3$.

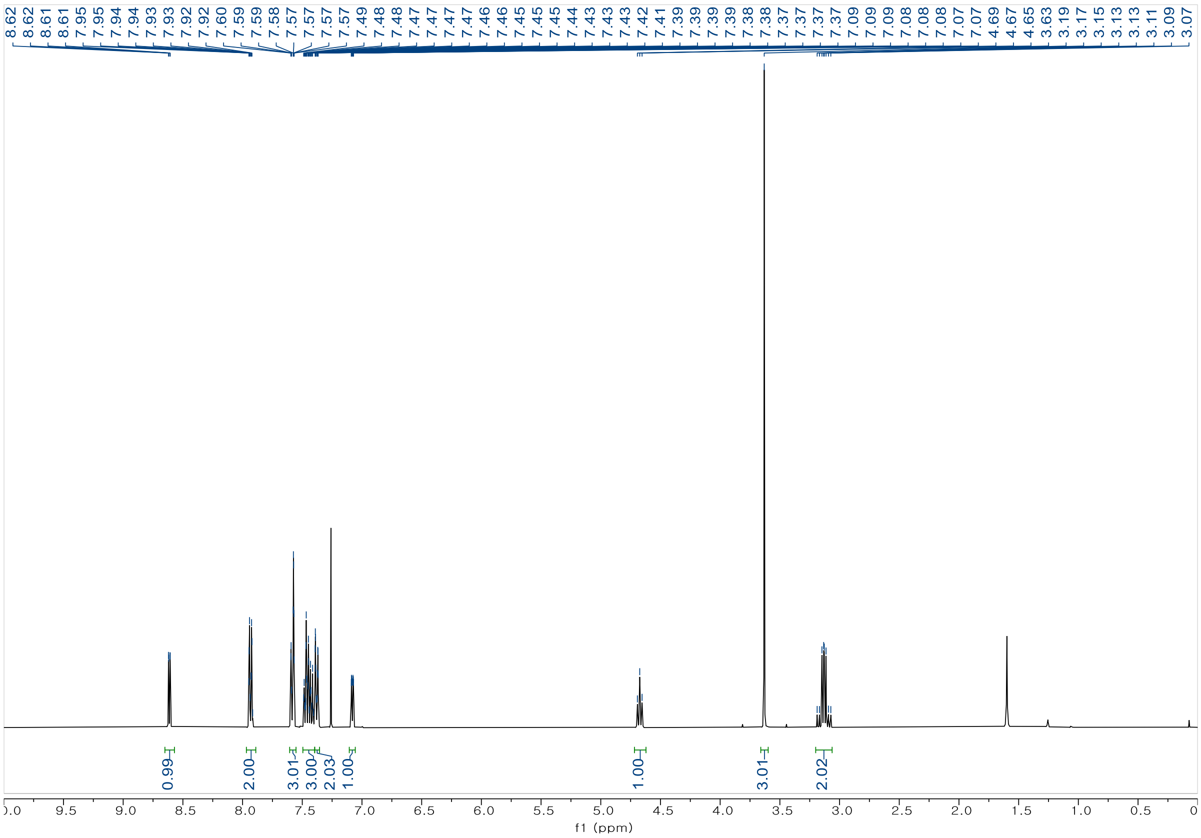

100 MHz, ^{13}C NMR in CDCl$_3$.

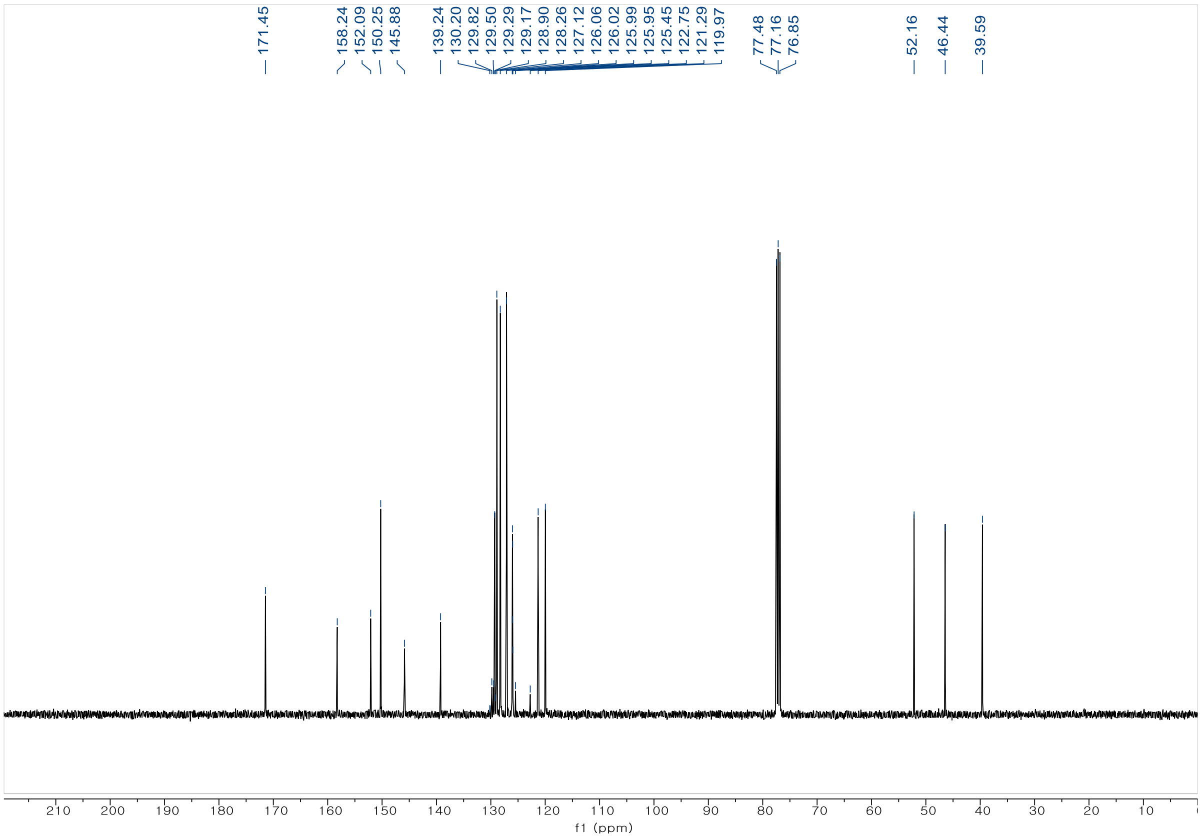

376 MHz, ^{19}F NMR in CDCl$_3$.

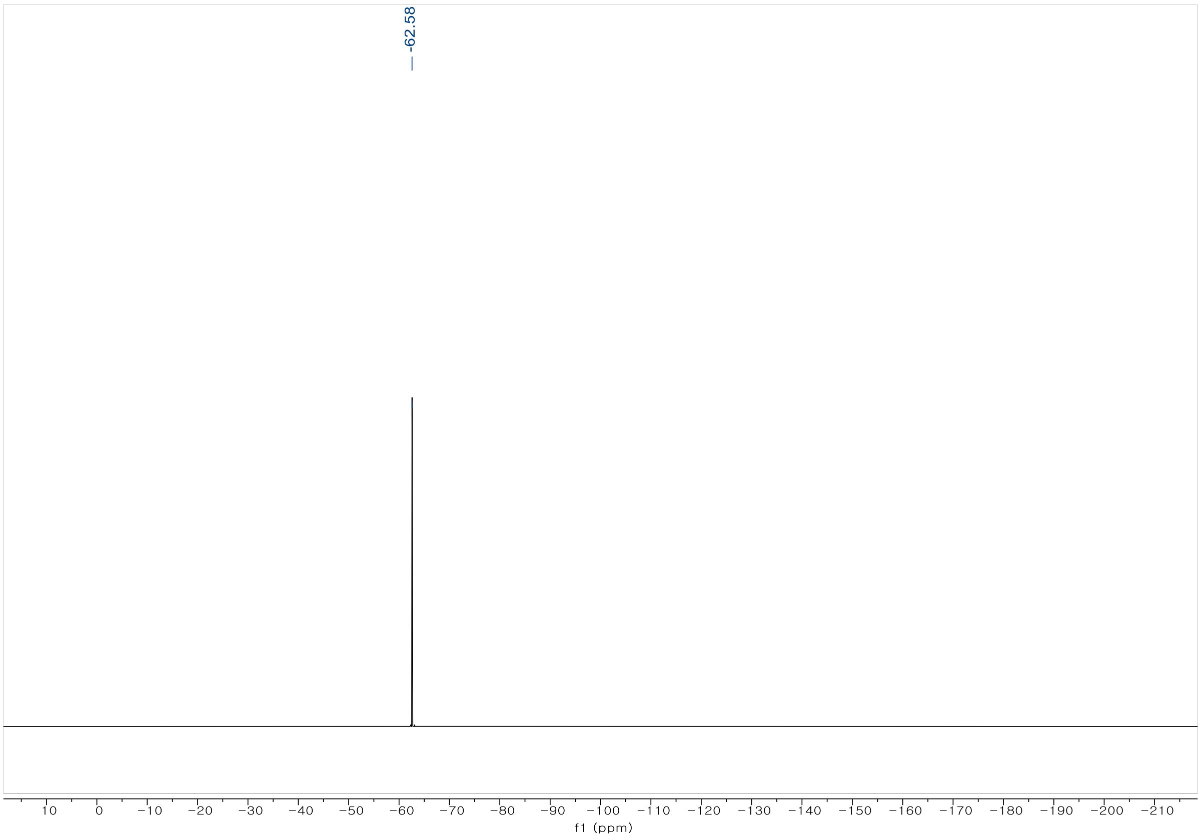

Supplementary Fig. 22. ^{1}H, ^{13}C, and ^{19}F NMR of **3e**.

Methyl (*R*)-3-(4-fluorophenyl)-3-(2-phenylpyridin-4-yl)propanoate (3f)

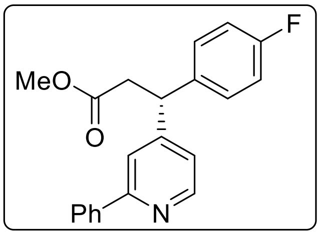

Prepared according to **GP**. Desired product **3f** was obtained as colorless oil (9.2 mg, 55% isolated yield). **^{1}H NMR** (400 MHz, $CDCl_3$) δ 8.60 (dd, *J* = 5.1, 0.8 Hz, 1H), 7.96 – 7.89 (m, 2H), 7.59 – 7.53 (m, 1H), 7.49 – 7.43 (m, 2H), 7.43 – 7.38 (m, 1H), 7.25 – 7.18 (m, 2H), 7.08 (ddd, *J* = 5.2, 1.7, 0.6 Hz, 1H), 7.04 – 6.97 (m, 2H), 4.59 (t, *J* = 7.9 Hz, 1H), 3.62 (s, 3H), 3.17 – 3.00 (m, 2H). **^{13}C NMR** (100 MHz, $CDCl_3$) δ 171.7, 162.0 (d, *J* = 246.0 Hz), 158.1, 152.9, 150.1, 139.4, 137.6 (d, *J* = 3.3 Hz), 129.4 (d, *J* = 8.0 Hz), 129.2, 128.9, 127.1, 121.3, 120.0, 115.9 (d, *J* = 21.4 Hz), 52.1, 45.9, 40.0. **HRMS** (ESI^+) m/z calcd. For $C_{21}H_{19}FNO_2^+$ $[M+H]^+$: 336.1400, found 336.1400. **Specific Rotation** $[\alpha]_D^{26}$ –2.6 (*c* 0.92, $CHCl_3$). **HPLC Analysis**. CHIRALCEL OD-H, 25 ºC; *n*-hexane:*i*-PrOH = 75:25, 1.0 mL/min, 254 nm, t_{R1} (major) = 7.58 min, t_{R2} (minor) = 14.17 min, 95:5 er.

The absolute stereochemistry was assigned by analogy to compound **3x** and **4k**.

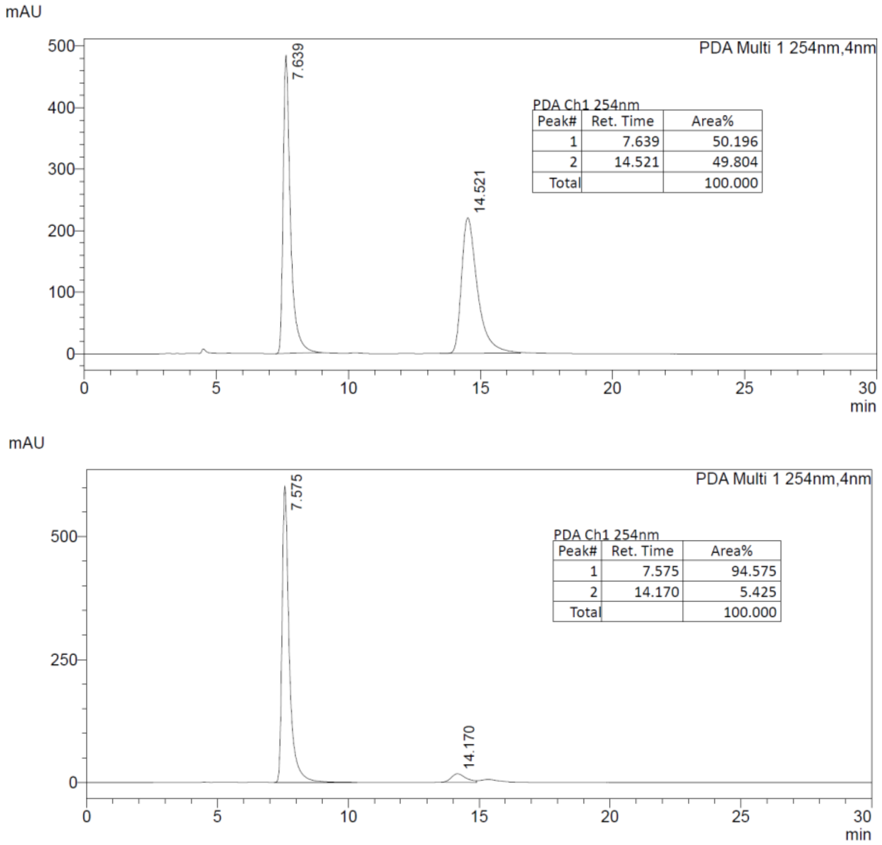

Supplementary Fig. 23. HPLC traces of *rac*-**3f** (top) and enantioenriched-**3f** (bottom).

400 MHz, ^{1}H NMR in $CDCl_3$.

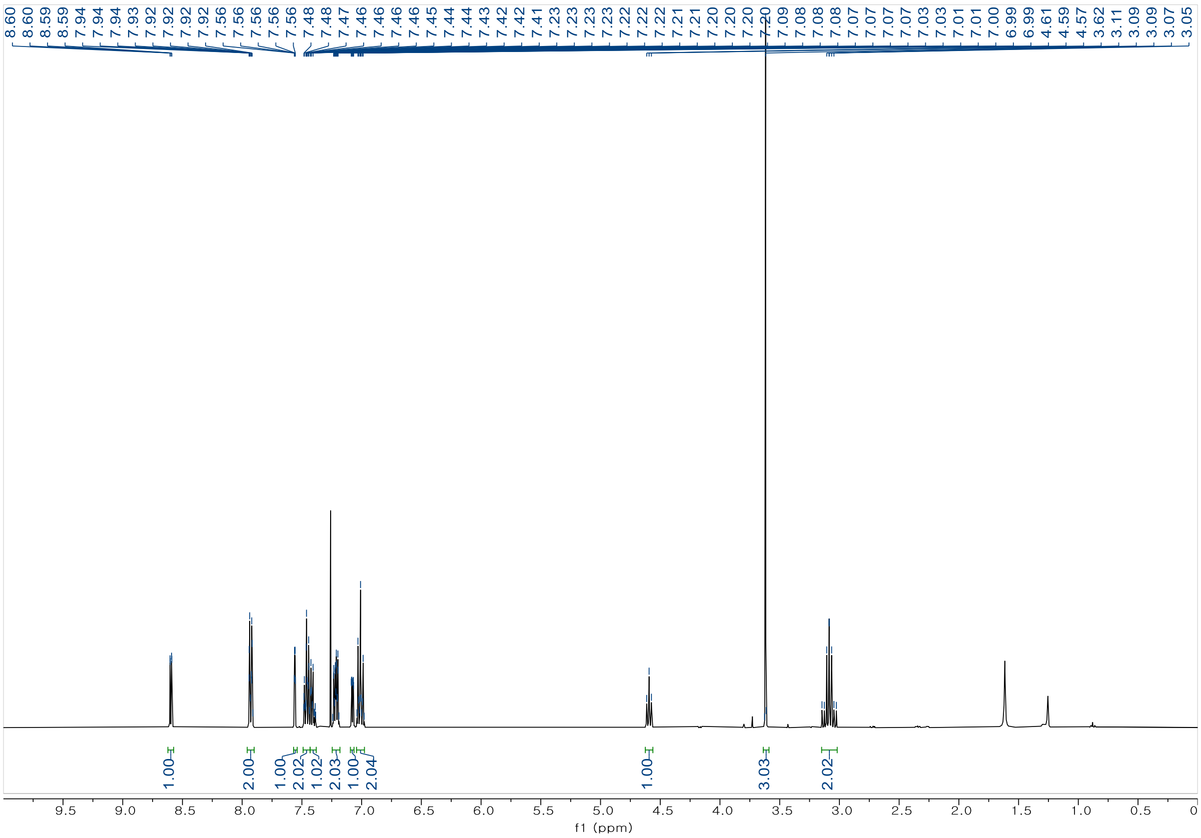

100 MHz, ^{13}C NMR in $CDCl_3$.

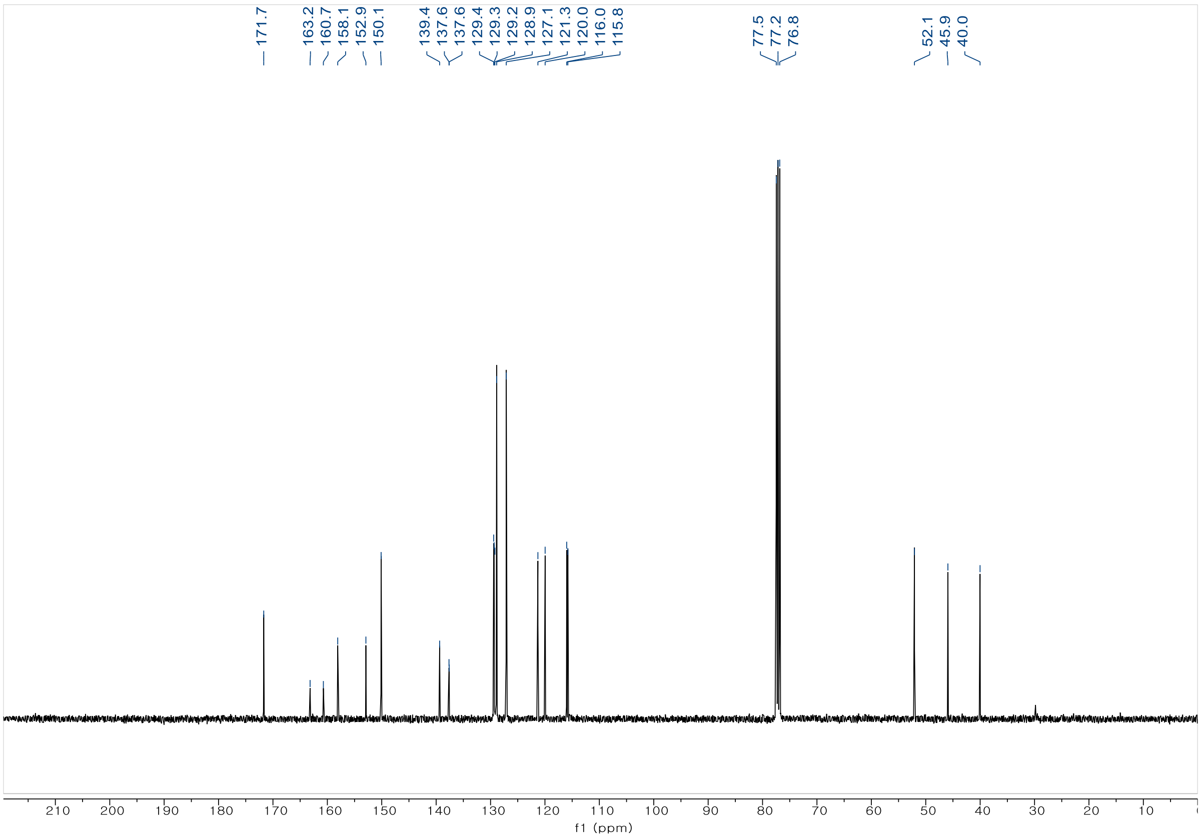

376 MHz, ^{19}F NMR in $CDCl_3$.

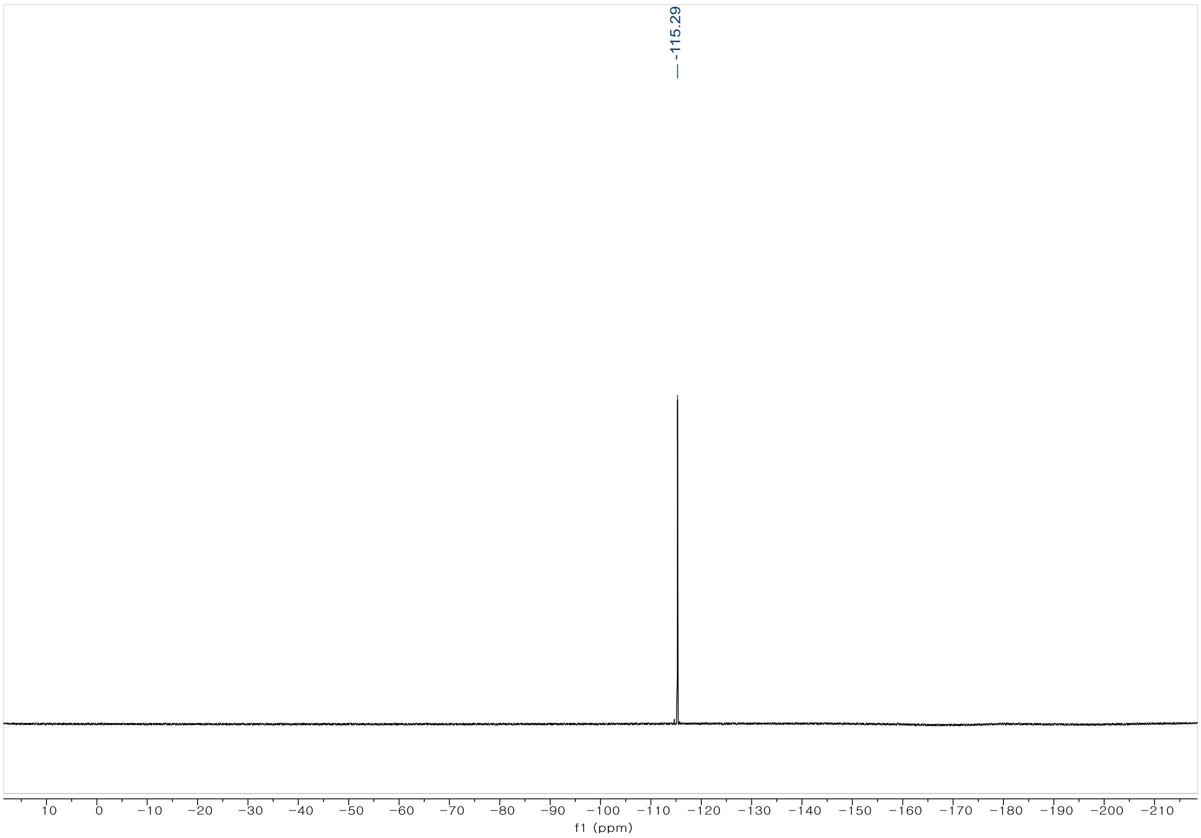

Supplementary Fig. 24. ^{1}H, ^{13}C, and ^{19}F NMR of **3f**.

Methyl (*R*)-3-(4-chlorophenyl)-3-(2-phenylpyridin-4-yl)propanoate (3g)

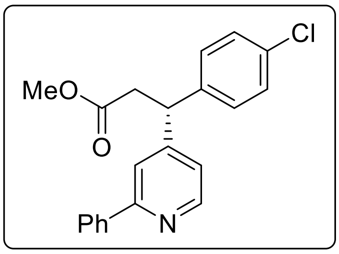

Prepared according to **GP**. Desired product **3g** was obtained as white solid (11.7 mg, 67% isolated yield). **^{1}H NMR** (400 MHz, $CDCl_3$) δ 8.60 (dd, *J* = 5.1, 0.8 Hz, 1H), 7.96 – 7.88 (m, 2H), 7.58 – 7.53 (m, 1H), 7.49 – 7.43 (m, 2H), 7.43 – 7.36 (m, 1H), 7.33 – 7.25 (m, 2H), 7.22 – 7.14 (m, 2H), 7.07 (dd, *J* = 5.2, 1.7 Hz, 1H), 4.58 (t, *J* = 7.9 Hz, 1H), 3.62 (s, 3H), 3.07 (h, *J* = 8.0 Hz, 2H). **^{13}C NMR** (100 MHz, $CDCl_3$) δ 171.6, 158.2, 152.6, 150.2, 140.4, 139.3, 133.2, 129.2, 129.2, 129.2, 128.9, 127.1, 121.3, 120.0, 52.1, 46.1, 39.8. **HRMS** (ESI^+) m/z calcd. For $C_{21}H_{19}NO_2Cl^+$ $[M+H]^+$: 352.1104, found 352.1103. **Specific Rotation** $[\alpha]_D^{25}$ +3.2 (*c* 0.4, $CHCl_3$). **HPLC Analysis**. CHIRALCEL OD-H, 25 °C; *n*-hexane:*i*-PrOH = 75:25, 1.0 mL/min, 254 nm, t_{R1} (major) = 8.32 min, t_{R2} (minor) = 16.02 min, 94:6 er.

The absolute stereochemistry was assigned by analogy to compound **3x** and **4k**.

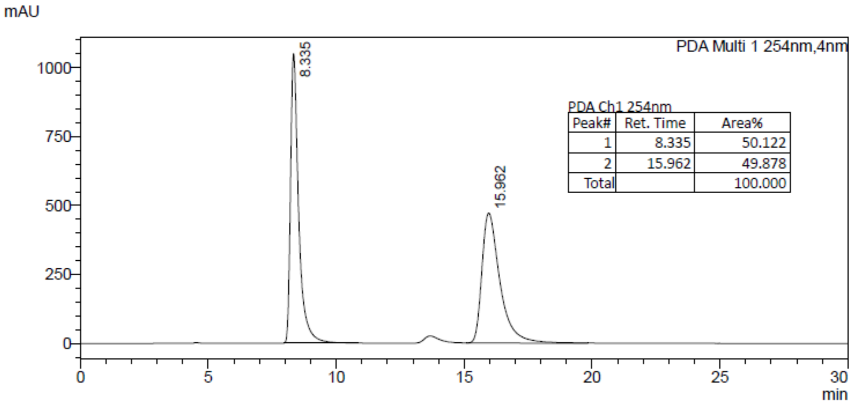

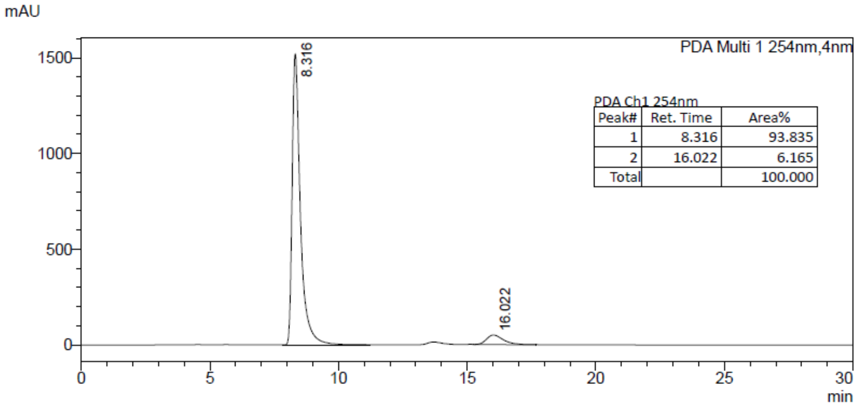

Supplementary Fig. 25. HPLC traces of *rac*-**3g** (top) and enantioenriched-**3g** (bottom).

400 MHz, ^{1}H NMR in $CDCl_3$

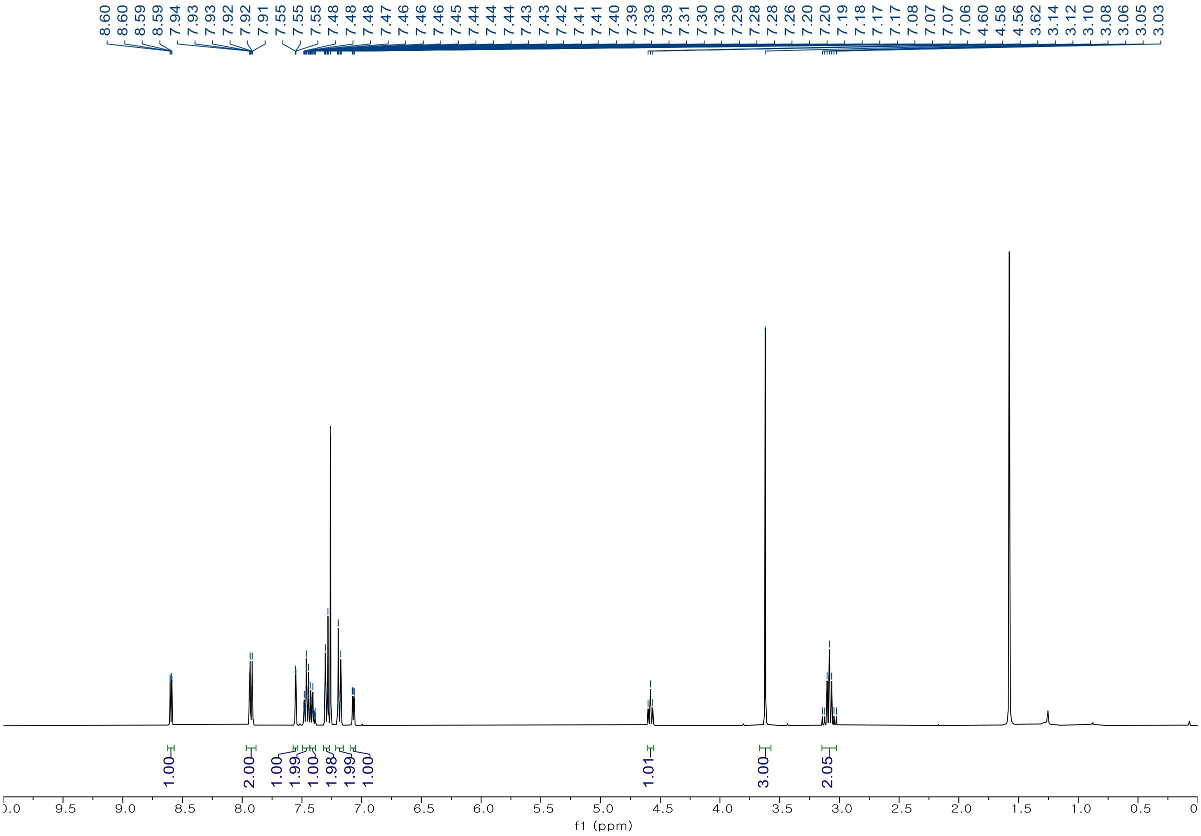

100 MHz, ^{13}C NMR in $CDCl_3$

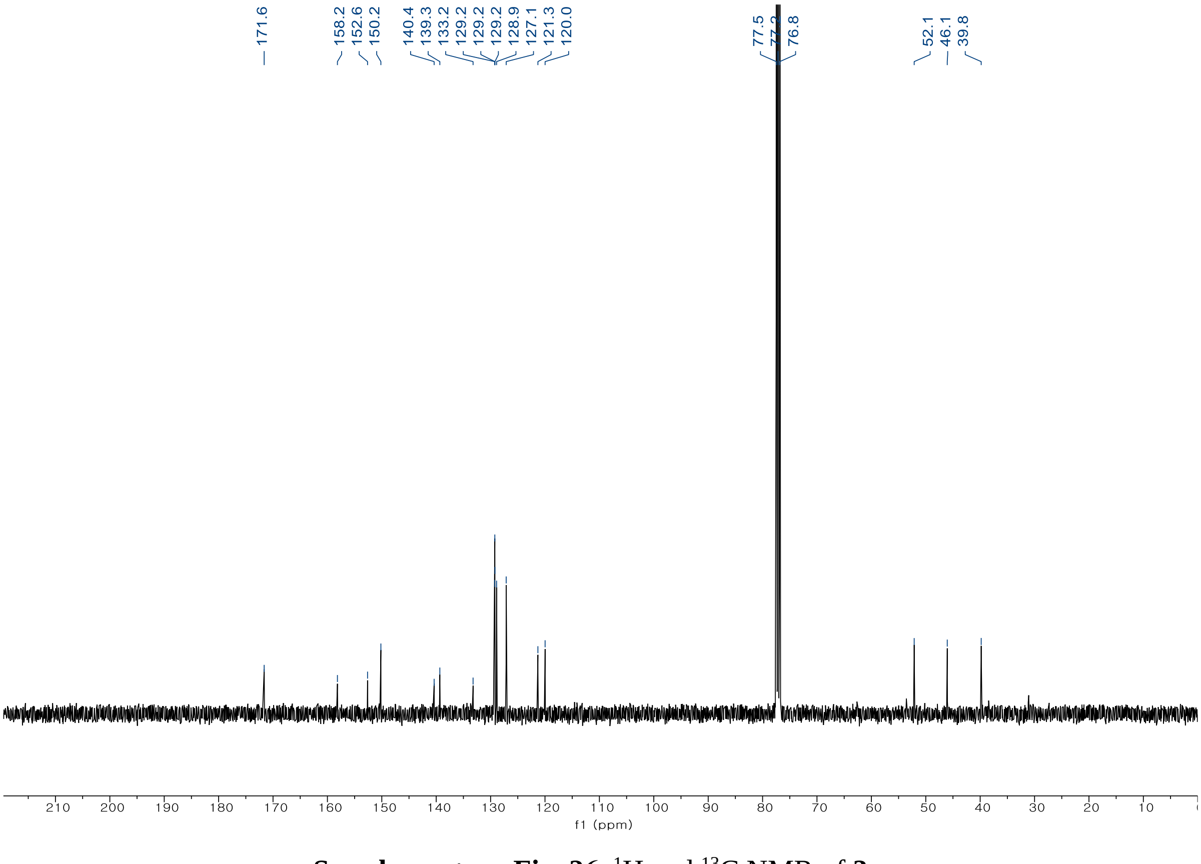

Supplementary Fig. 26. ^{1}H and ^{13}C NMR of **3g**.

Methyl (*R*)-3-(4-bromophenyl)-3-(2-phenylpyridin-4-yl)propanoate (3h)

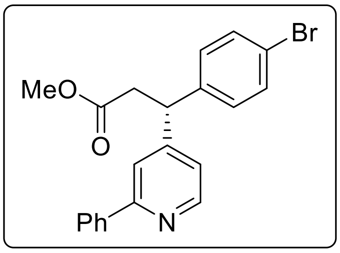

Prepared according to **GP**. Desired product **3h** was obtained as colorless oil (14.4 mg, 73% isolated yield). **^{1}H NMR** (600 MHz, $CDCl_3$) δ 8.60 (d, *J* = 5.1 Hz, 1H), 7.95 – 7.90 (m, 2H), 7.57 – 7.53 (m, 1H), 7.48 – 7.39 (m, 5H), 7.13 (d, *J* = 8.5 Hz, 2H), 7.07 (dd, *J* = 5.1, 1.7 Hz, 1H), 4.57 (t, *J* = 7.9 Hz, 1H), 3.62 (s, 3H), 3.14 – 3.02 (m, 2H). **^{13}C NMR** (125 MHz, $CDCl_3$) δ 171.6, 158.1, 152.5, 150.2, 140.9, 139.3, 132.1, 129.6, 129.2, 128.9, 127.1, 121.3, 121.3, 119.9, 52.1, 46.1, 39.7. **HRMS** (ESI^+) m/z calcd. For $C_{21}H_{19}NO_2Br^+$ $[M+H]^+$ 396.0599, found 396.0599. **Specific Rotation** $[\alpha]_D^{25}$ +5.6 (*c* 1.0, $CHCl_3$). **HPLC Analysis**. CHIRALCEL OD-H, 25 ºC; *n*-hexane:*i*-PrOH = 75:25, 1.0 mL/min, 254 nm, t_{R1} (major) = 9.02 min, t_{R2} (minor) = 16.37 min, 92:8 er.

The absolute stereochemistry was assigned by analogy to compound **3x** and **4k**.

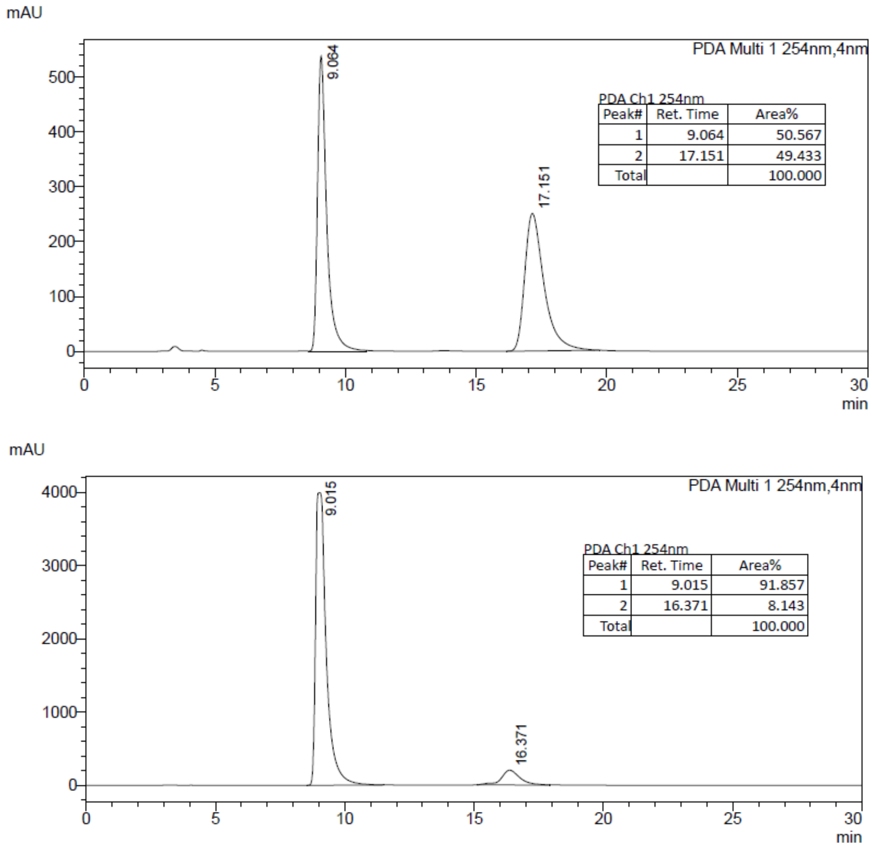

Supplementary Fig. 27. HPLC traces of *rac*-**3h** (top) and enantioenriched-**3h** (bottom).

600 MHz, ^{1}H NMR in $CDCl_3$

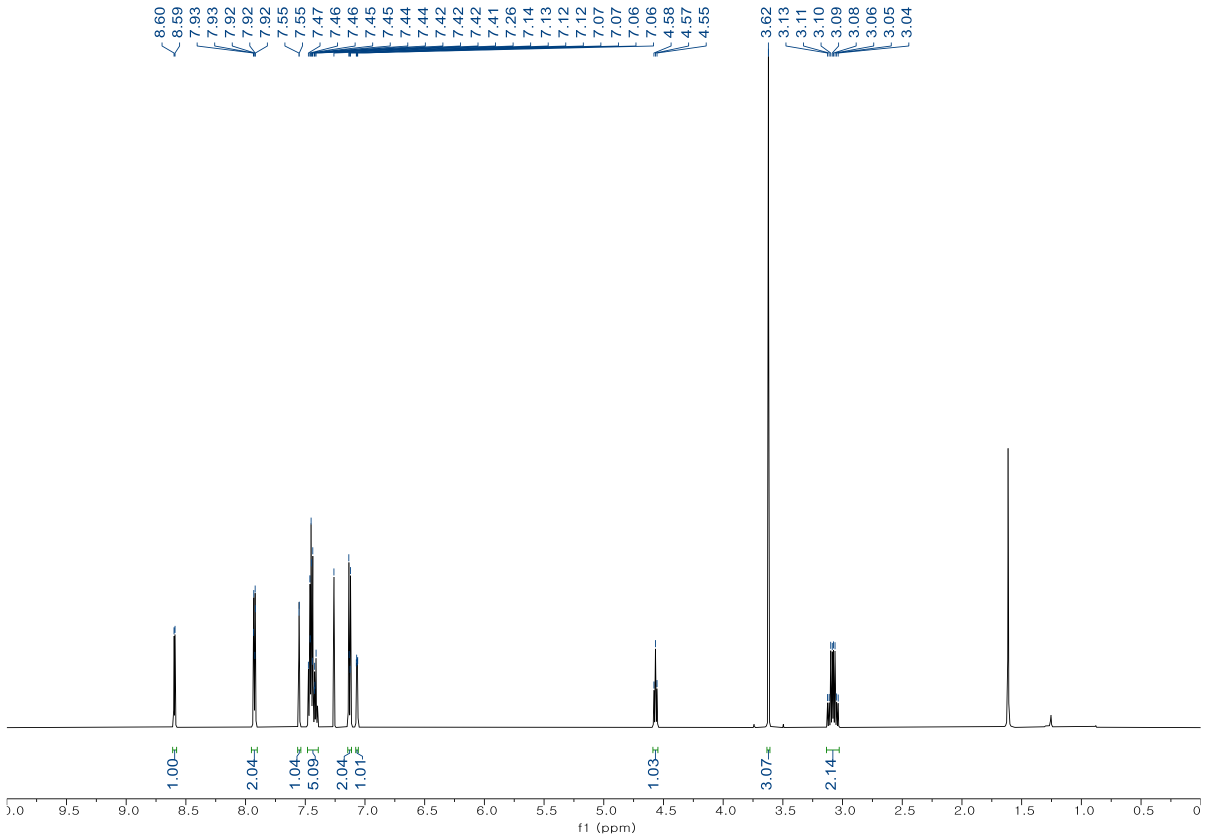

125 MHz, ^{13}C NMR in $CDCl_3$

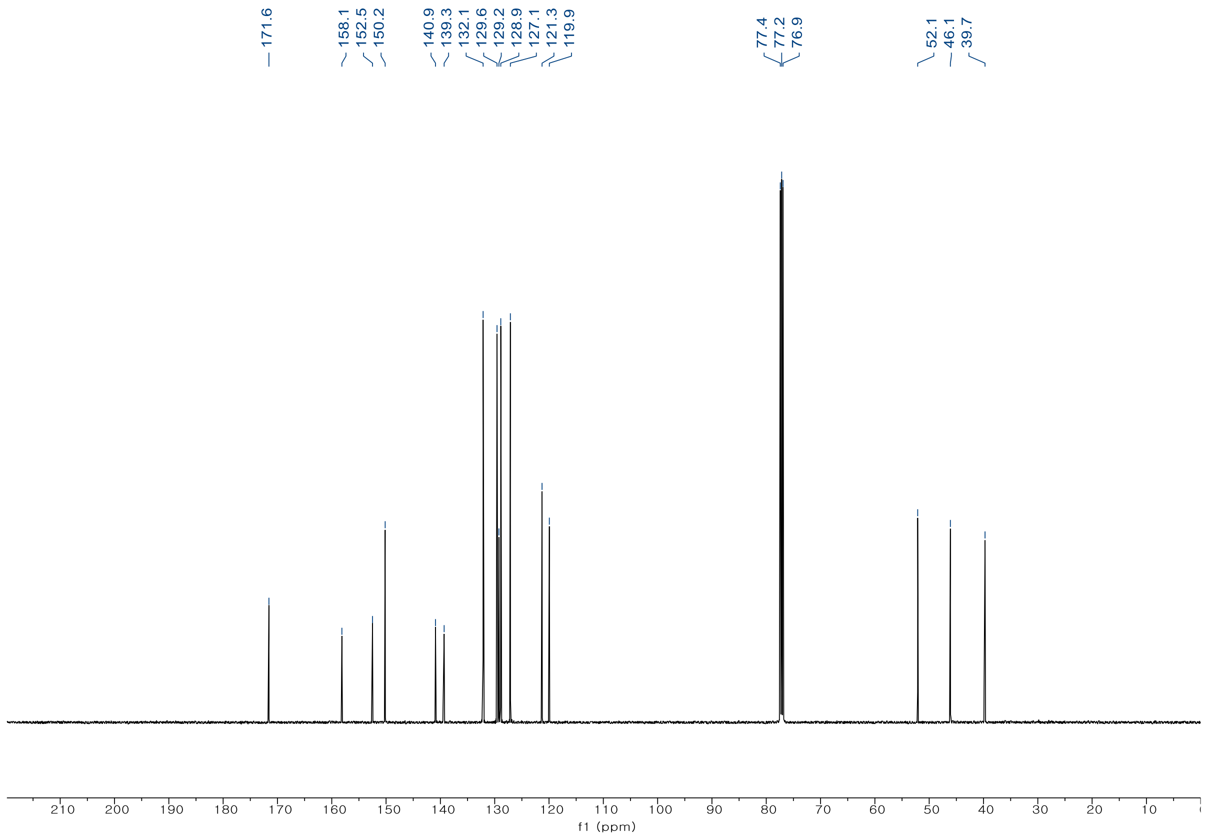

Supplementary Fig. 28. ^{1}H and ^{13}C NMR of **3h**.

Methyl (*R*)-3-(4-cyanophenyl)-3-(2-phenylpyridin-4-yl)propanoate (3i)

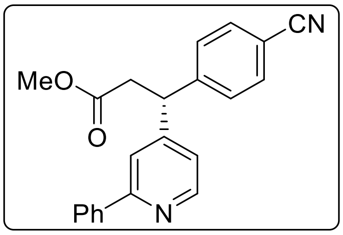

Prepared according to **GP**. Desired product **3i** was obtained as colorless oil (11.4 mg, 67% isolated yield). **^{1}H NMR** (400 MHz, $CDCl_3$) δ 8.62 (dd, *J* = 5.1, 0.8 Hz, 1H), 7.95 – 7.89 (m, 2H), 7.66 – 7.59 (m, 2H), 7.57 – 7.51 (m, 1H), 7.49 – 7.39 (m, 3H), 7.39 – 7.35 (m, 2H), 7.08 – 7.04 (m, 1H), 4.66 (t, *J* = 7.8 Hz, 1H), 3.63 (s, 3H), 3.20 – 3.04 (m, 2H). **^{13}C NMR** (100 MHz, $CDCl_3$) δ 171.3, 158.4, 151.6, 150.4, 147.2, 139.1, 132.9, 129.4, 129.0, 128.8, 127.1, 121.2, 119.9, 118.6, 111.5, 52.3, 46.6, 39.4. **HRMS** (ESI^+) m/z calcd. For $C_{22}H_{19}N_2O_2^+$ $[M+H]^+$: 343.1447, found 343.1447. **Specific Rotation** $[\alpha]_D^{26}$ +3.7 (*c* 0.6, $CHCl_3$). **HPLC Analysis**. CHIRALCEL OD-H, 25 °C; *n*-hexane:*i*-PrOH = 75:25, 1.0 mL/min, 254 nm, t_{R1} (major) = 16.01 min, t_{R2} (minor) = 27.84 min, 85:15 er.

The absolute stereochemistry was assigned by analogy to compound **3x** and **4k**.

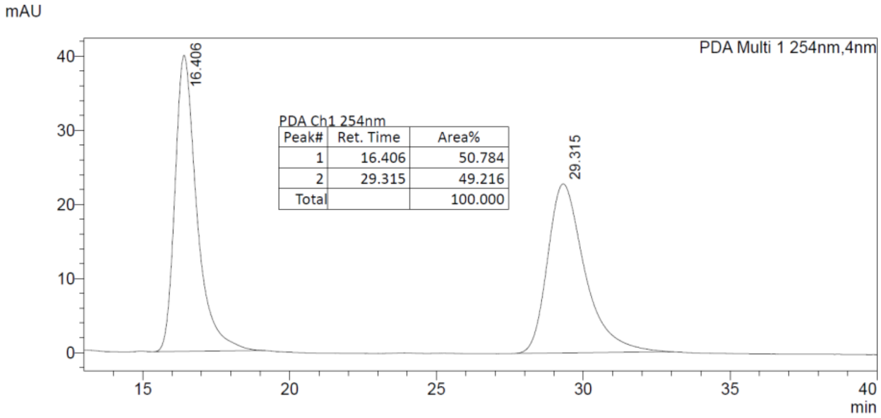

Peak#	Ret. Time	Area%
1	16.406	50.784
2	29.315	49.216
Total		100.000

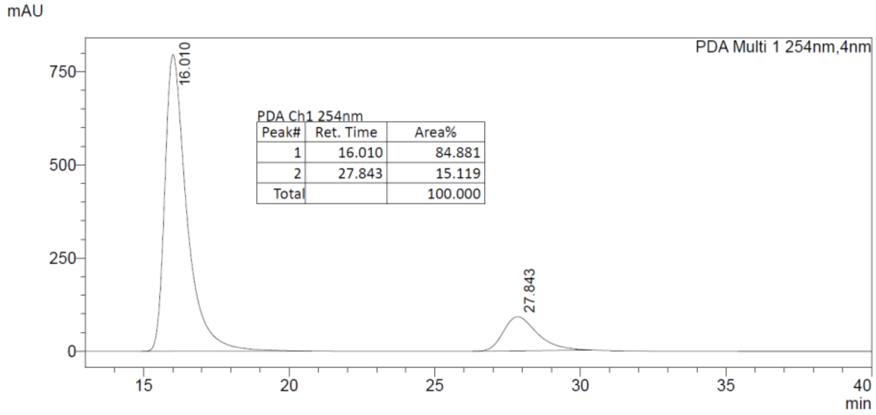

Peak#	Ret. Time	Area%
1	16.010	84.881
2	27.843	15.119
Total		100.000

Supplementary Fig. 29. HPLC traces of *rac*-**3i** (top) and enantioenriched-**3i** (bottom).

400 MHz, ^{1}H NMR in $CDCl_3$.

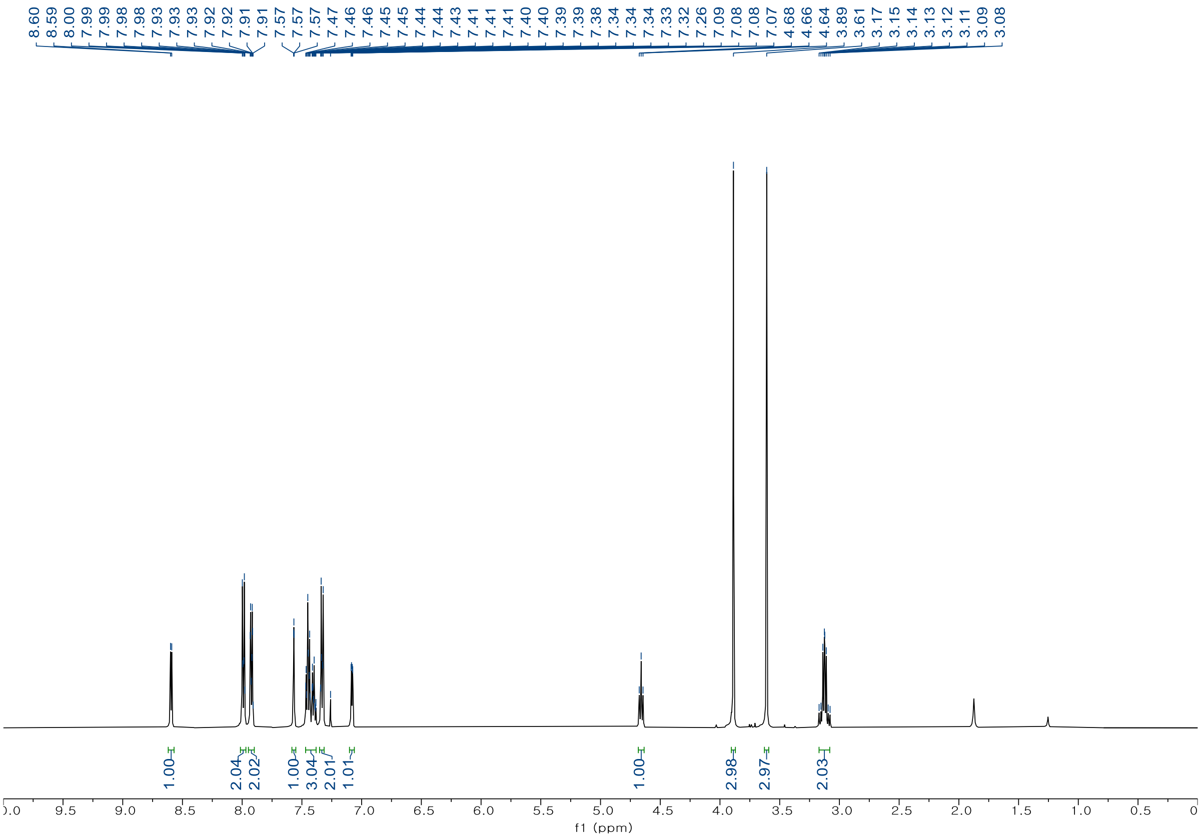

100 MHz, ^{13}C NMR in $CDCl_3$.

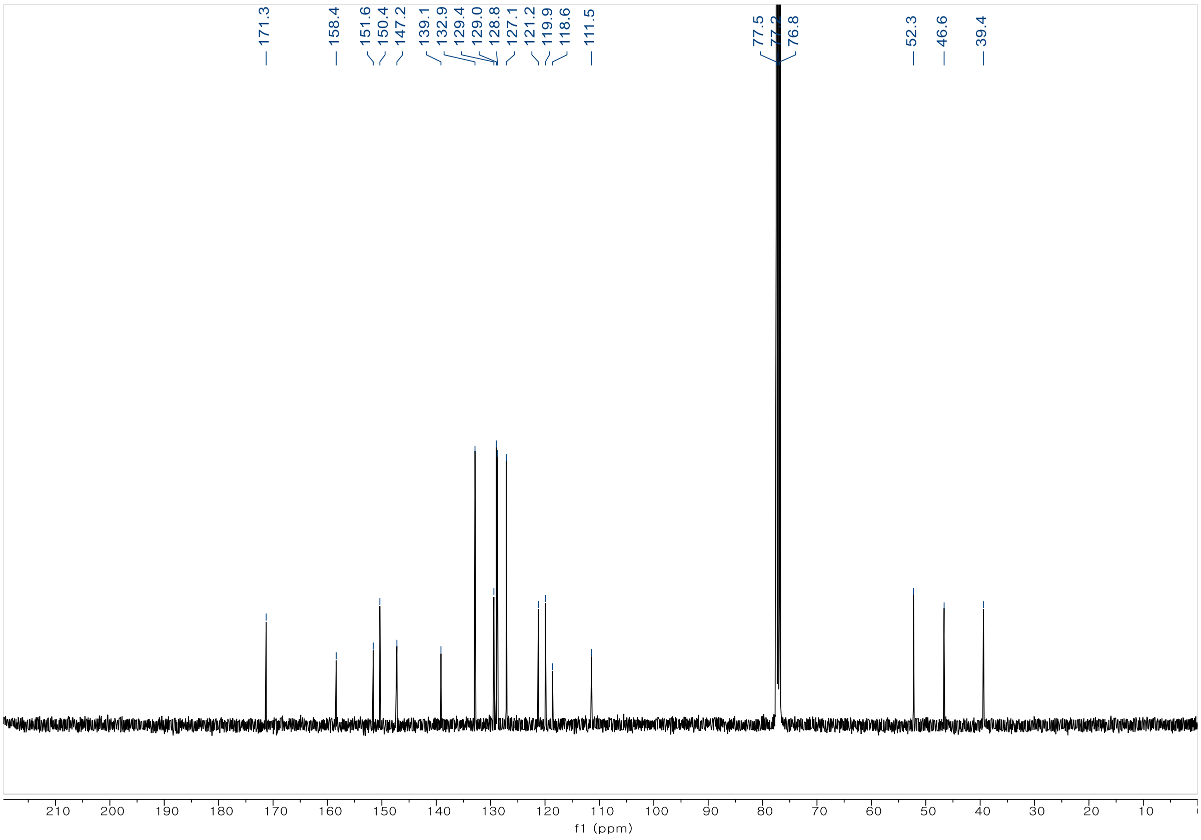

Supplementary Fig. 30. ^{1}H and ^{13}C NMR of **3i**.

Methyl (*R*)-4-(3-methoxy-3-oxo-1-(2-phenylpyridin-4-yl)propyl)benzoate (3j)

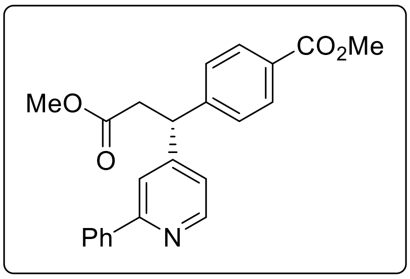

Prepared according to **GP**. Desired product **3j** was obtained as colorless oil (13.1 mg, 70% isolated yield). **^{1}H NMR** (500 MHz, $CDCl_3$) δ 8.59 (d, *J* = 5.1 Hz, 1H), 8.03 – 7.97 (m, 2H), 7.94 – 7.90 (m, 2H), 7.59 – 7.55 (m, 1H), 7.48 – 7.37 (m, 3H), 7.36 – 7.31 (m, 2H), 7.08 (dd, *J* = 5.2, 1.7 Hz, 1H), 4.66 (t, *J* = 7.9 Hz, 1H), 3.89 (s, 3H), 3.61 (s, 3H), 3.19 – 3.06 (m, 2H). **^{13}C NMR** (125 MHz, $CDCl_3$) δ 171.5, 166.8, 158.1, 152.2, 150.2, 146.9, 139.2, 130.3, 129.3, 129.2, 128.8, 127.9, 127.1, 121.3, 120.0, 52.2, 52.1, 46.6, 39.6. **HRMS** (ESI^+) m/z calcd. For $C_{23}H_{22}NO_4^+$ $[M+H]^+$: 374.1549, found 376.1548. **Specific Rotation** $[\alpha]_D^{25}$ +0.4 (*c* 1.0, $CHCl_3$). **HPLC Analysis**. CHIRALCEL OD-H, 25 °C; *n*-hexane:*i*-PrOH = 75:25, 1.0 mL/min, 254 nm, t_{R1} (major) = 13.39 min, t_{R2} (minor) = 23.47 min, 87:13 er. The absolute stereochemistry was assigned by analogy to compound **3x** and **4k**.

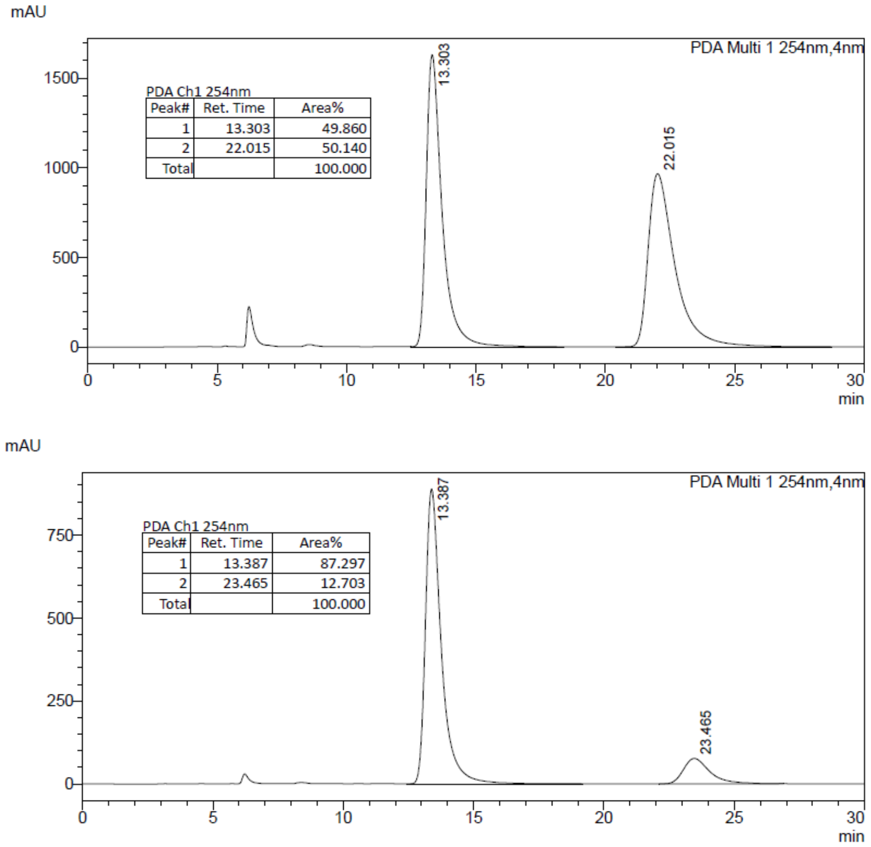

Supplementary Fig. 31. HPLC traces of *rac*-**3j** (top) and enantioenriched-**3j** (bottom).

500 MHz, ^{1}H NMR in CDCl$_3$

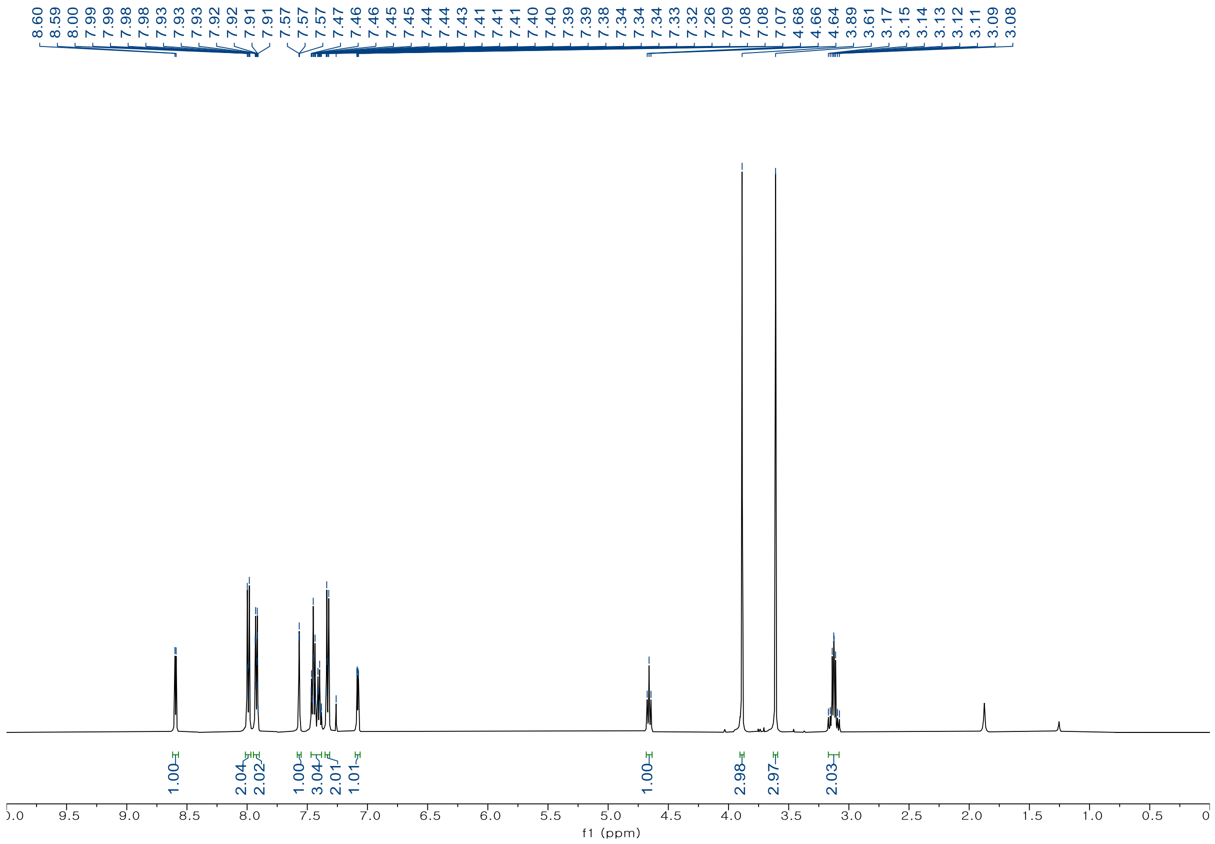

125 MHz, ^{13}C NMR in CDCl$_3$

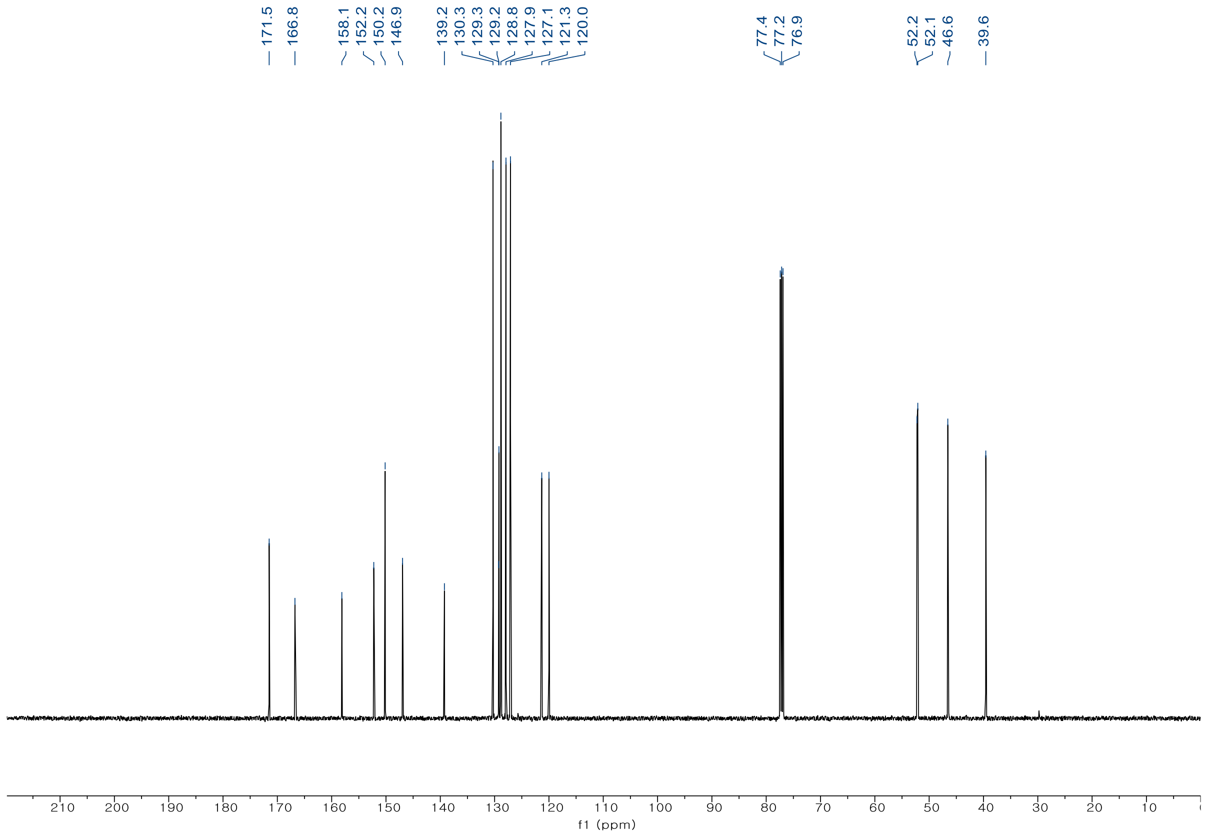

Supplementary Fig. 32. ^{1}H and ^{13}C NMR of **3j**.

Methyl (*R*)-3-(2-phenylpyridin-4-yl)-3-(*m*-tolyl)propanoate (3k)

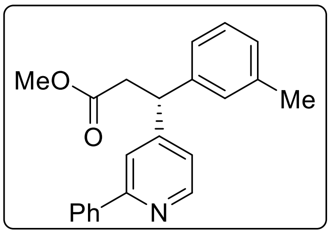

Prepared according to **GP**. Desired product **3k** was obtained as colorless oil (10.4 mg, 63% isolated yield). **^{1}H NMR** (400 MHz, $CDCl_3$) δ 8.59 (dd, *J* = 5.1, 0.8 Hz, 1H), 7.97 – 7.90 (m, 2H), 7.62 – 7.57 (m, 1H), 7.49 – 7.43 (m, 2H), 7.43 – 7.37 (m, 1H), 7.24 – 7.17 (m, 1H), 7.13 – 7.09 (m, 1H), 7.08 – 7.02 (m, 3H), 4.56 (t, *J* = 7.9 Hz, 1H), 3.62 (s, 3H), 3.18 – 3.02 (m, 2H), 2.32 (s, 3H). **^{13}C NMR** (100 MHz, $CDCl_3$) δ 171.9, 158.0, 153.3, 150.0, 141.9, 139.5, 138.7, 129.1, 128.9, 128.9, 128.7, 128.1, 127.2, 124.7, 121.5, 120.2, 52.0, 46.7, 39.9, 21.6. **HRMS** (ESI^+) m/z calcd. For $C_{22}H_{22}NO_2^+$ $[M+H]^+$: 332.1651, found 332.1652. **Specific Rotation** $[\alpha]_D^{26}$ +1.0 (*c* 0.79, $CHCl_3$). **HPLC Analysis**. CHIRALCEL OD-H, 25 °C; *n*-hexane:*i*-PrOH = 75:25, 1.0 mL/min, 254 nm, t_{R1} (major) = 6.87 min, t_{R2} (minor) = 14.33 min, 96:4 er.

The absolute stereochemistry was assigned by analogy to compound **3x** and **4k**.

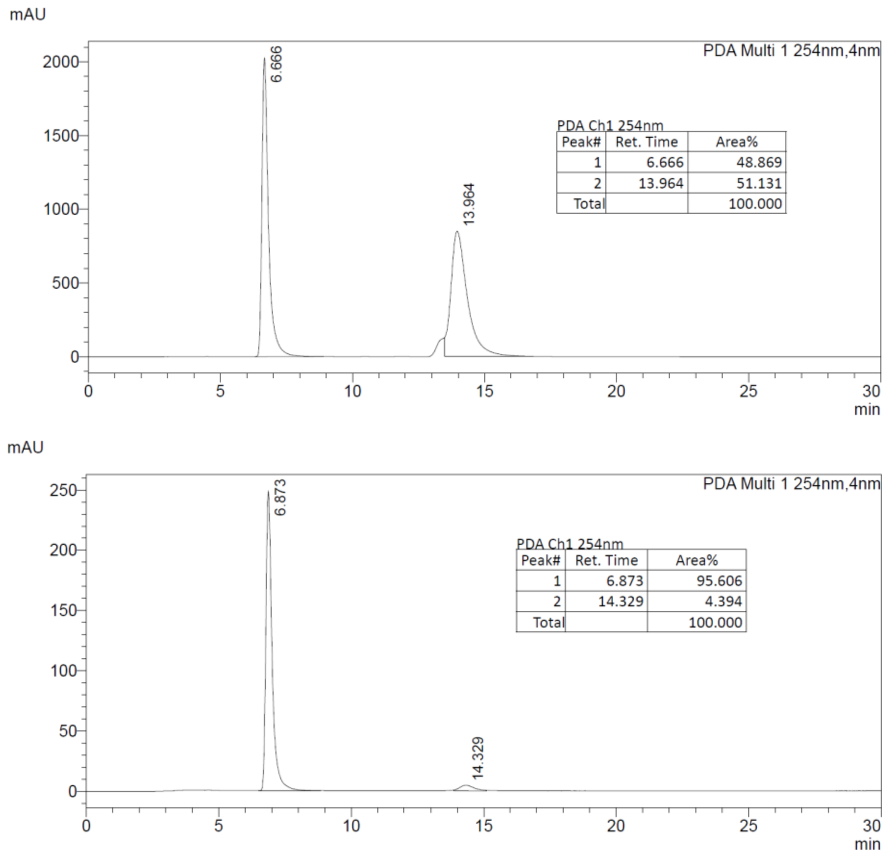

Supplementary Fig. 33. HPLC traces of *rac*-**3k** (top) and enantioenriched-**3k** (bottom).

400 MHz, ^{1}H NMR in $CDCl_3$.

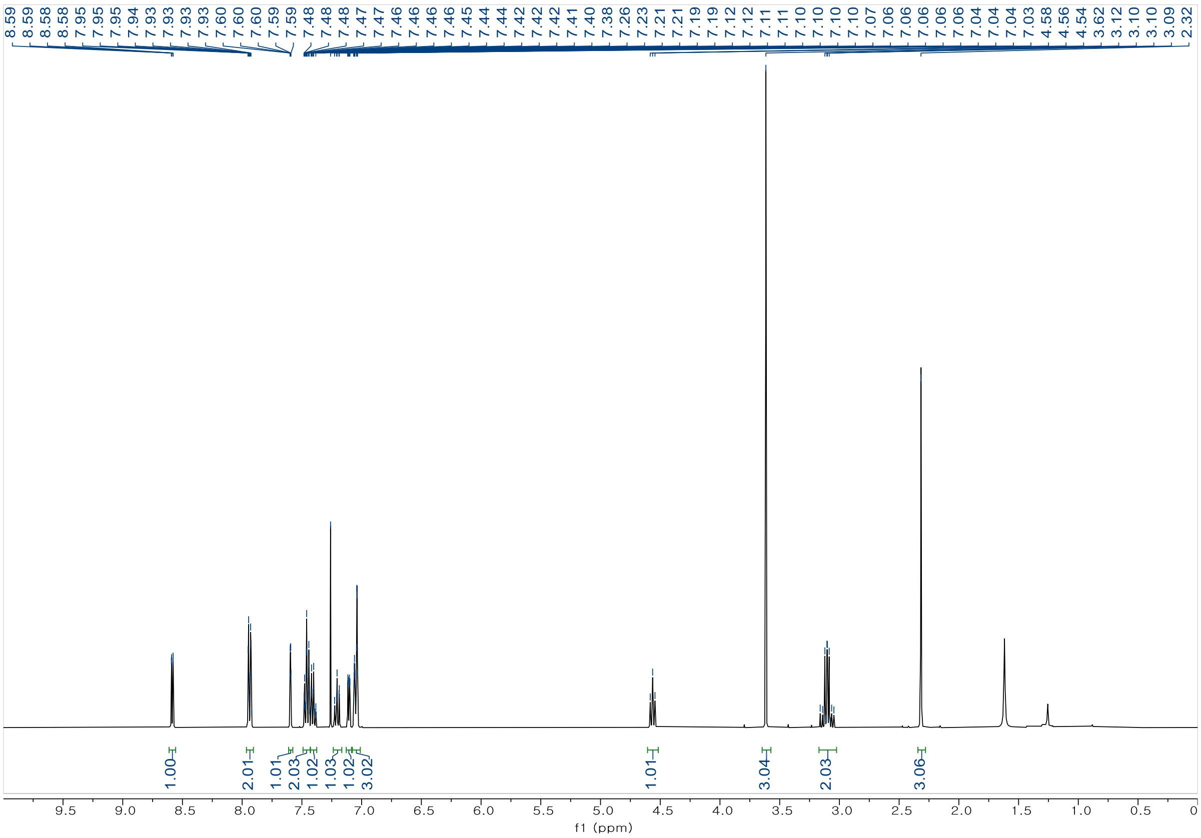

100 MHz, ^{13}C NMR in $CDCl_3$.

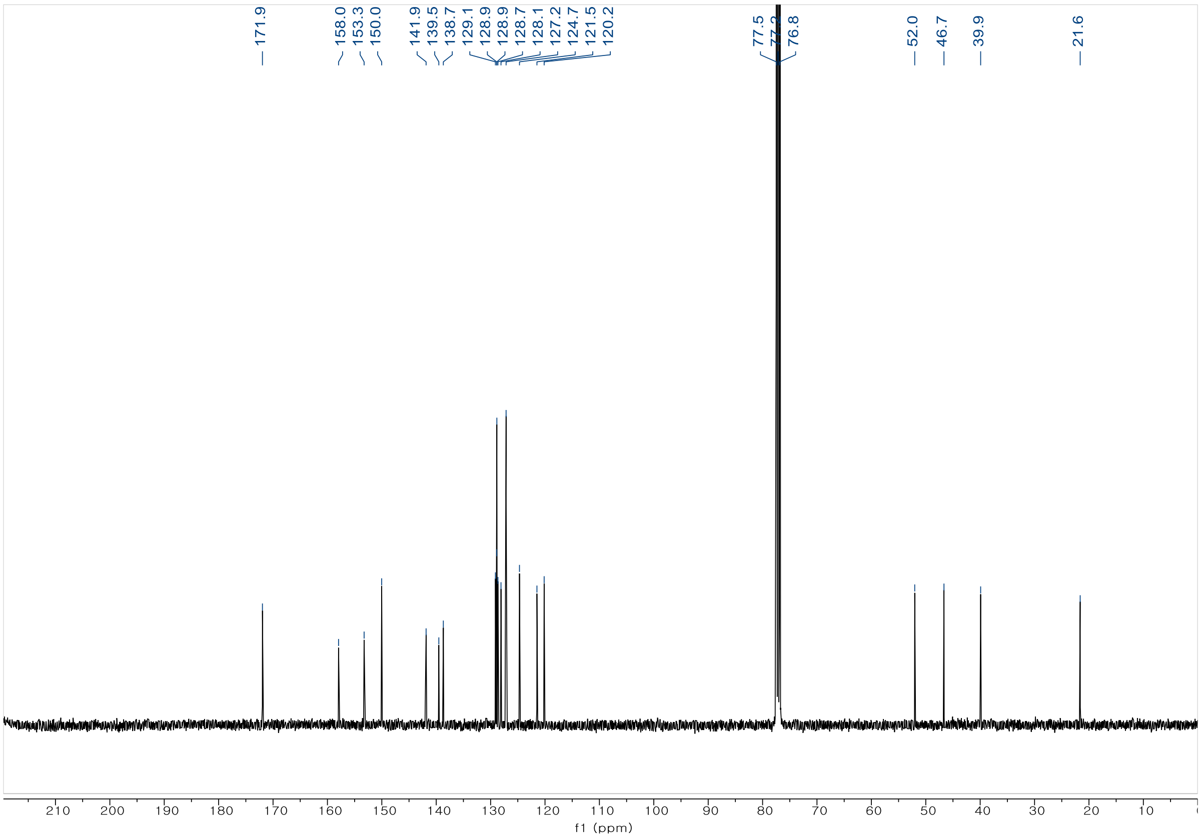

Supplementary Fig. 34. ^{1}H and ^{13}C NMR of **3k**.

Methyl (*S*)-3-(3-methoxyphenyl)-3-(2-phenylpyridin-4-yl)propanoate (3l)

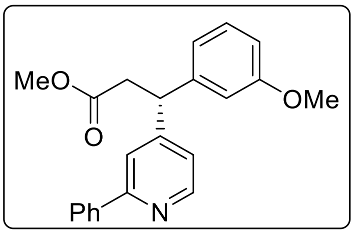

Prepared according to **GP**. Desired product **3l** was obtained as colorless oil (11.6 mg, 67% isolated yield). **^{1}H NMR** (400 MHz, $CDCl_3$) δ 8.59 (dd, *J* = 5.1, 0.8 Hz, 1H), 7.97 – 7.89 (m, 2H), 7.62 – 7.57 (m, 1H), 7.49 – 7.43 (m, 2H), 7.43 – 7.36 (m, 1H), 7.27 – 7.21 (m, 1H), 7.11 (dd, *J* = 5.1, 1.7 Hz, 1H), 6.88 – 6.81 (m, 1H), 6.81 – 6.74 (m, 2H), 4.57 (t, *J* = 7.9 Hz, 1H), 3.77 (s, 3H), 3.62 (s, 3H), 3.18 – 3.03 (m, 2H). **^{13}C NMR** (100 MHz, $CDCl_3$) δ 171.9, 160.0, 158.0, 153.0, 150.0, 143.5, 139.5, 130.0, 129.1, 128.8, 127.1, 121.4, 120.1, 120.1, 114.1, 112.2, 55.3, 52.1, 46.7, 39.8. **HRMS** (ESI$^+$) m/z calcd. For $C_{22}H_{22}NO_3^+$ $[M+H]^+$: 348.1600, found 348.1600. **Specific Rotation** $[\alpha]_D^{26}$ +1.2 (*c* 1.0, $CHCl_3$). **HPLC Analysis**. CHIRALCEL OD-H, 25 °C; *n*-hexane:*i*-PrOH = 75:25, 1.0 mL/min, 254 nm, t_{R1} (major) = 12.28 min, t_{R2} (minor) = 20.75 min, 95:5 er.

The absolute stereochemistry was assigned by analogy to compound **3x** and **4k**.

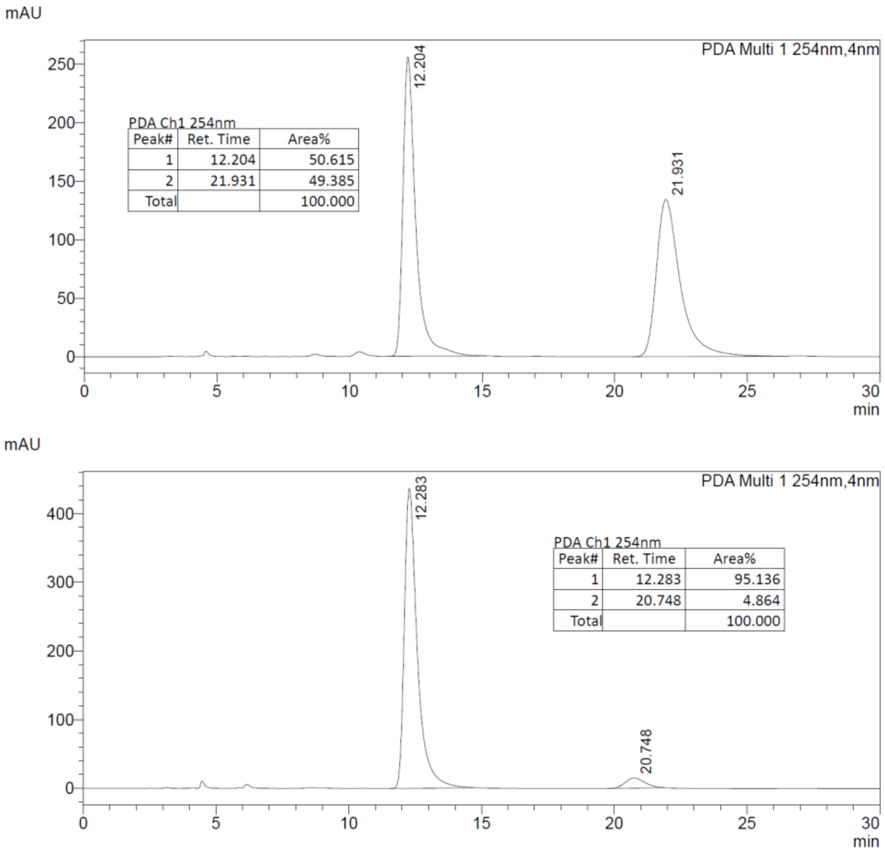

Supplementary Fig. 35. HPLC traces of *rac*-**3l** (top) and enantioenriched-**3l** (bottom).

400 MHz, ^{1}H NMR in $CDCl_3$.

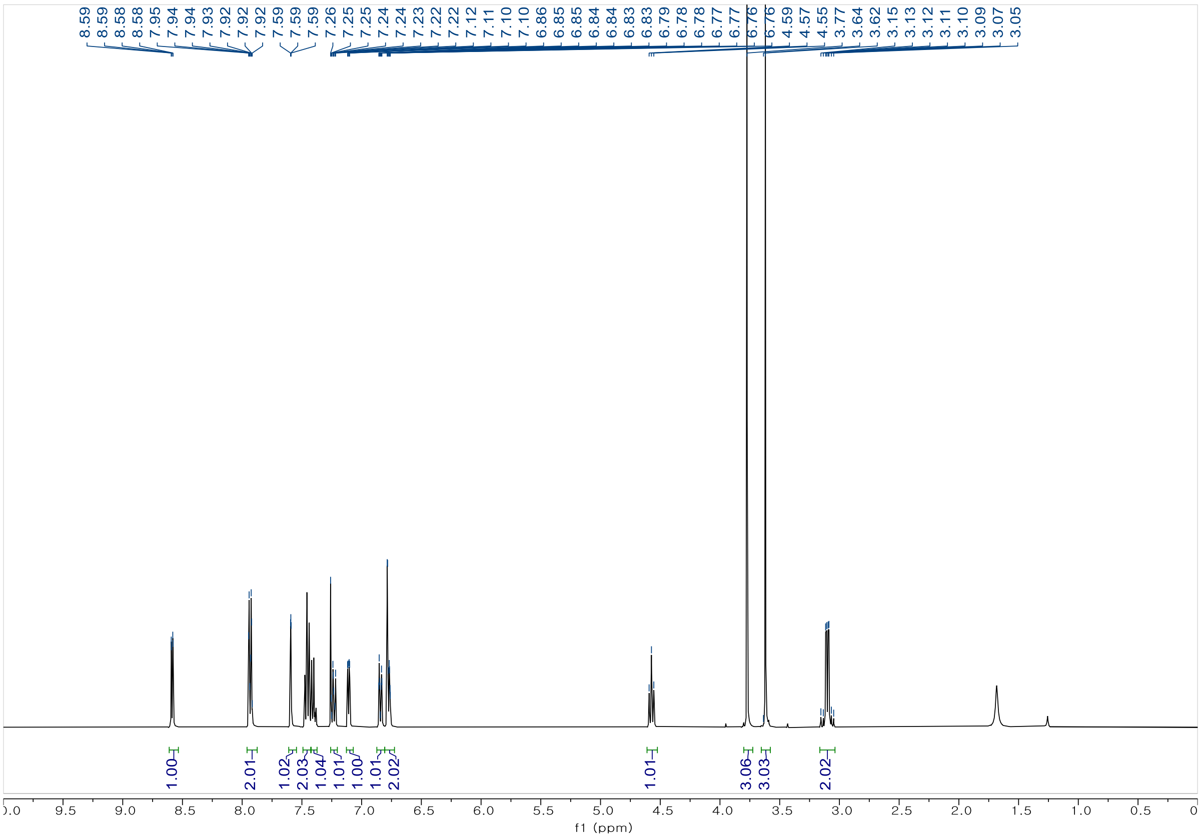

100 MHz, ^{13}C NMR in $CDCl_3$.

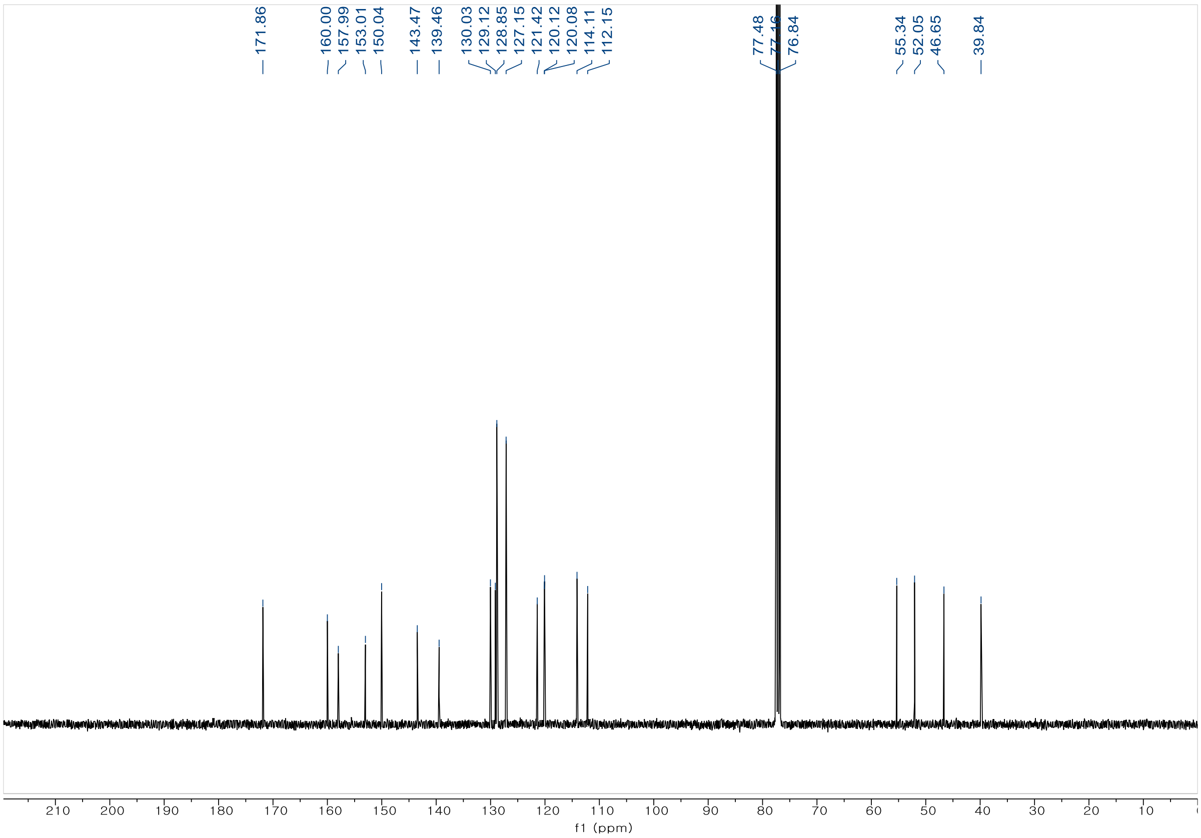

Supplementary Fig. 36. ^{1}H and ^{13}C NMR of **3l**.

Methyl (*S*)-3-(3-chlorophenyl)-3-(2-phenylpyridin-4-yl)propanoate (3m)

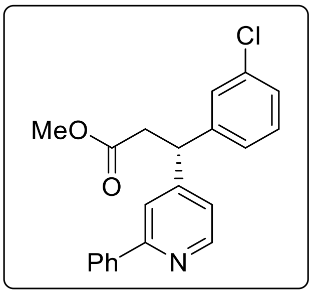

Prepared according to **GP**. Desired product **3m** was obtained as white solid (12.6 mg, 72% isolated yield). **^{1}H NMR** (400 MHz, $CDCl_3$) δ 8.61 (dd, *J* = 5.2, 0.8 Hz, 1H), 7.96 – 7.91 (m, 2H), 7.59 – 7.55 (m, 1H), 7.49 – 7.39 (m, 3H), 7.26 – 7.19 (m, 3H), 7.14 (dt, *J* = 7.1, 1.8 Hz, 1H), 7.08 (dd, *J* = 5.1, 1.7 Hz, 1H), 4.58 (t, *J* = 7.9 Hz, 1H), 3.63 (s, 3H), 3.18 – 3.02 (m, 2H). **^{13}C NMR** (125 MHz, $CDCl_3$) δ 171.5, 158.2, 152.3, 150.2, 143.9, 139.3, 134.9, 130.3, 129.2, 128.9, 128.1, 127.6, 127.2, 126.0, 121.3, 120.0, 52.2, 46.3, 39.7. **HRMS** (ESI^+) m/z calcd. For $C_{21}H_{19}NO_2Cl^+$ $[M+H]^+$: 352.1104, found 352.1104. **Specific Rotation** $[\alpha]_D^{20}$ +5.3 (*c* 0.5, $CHCl_3$). **HPLC Analysis**. CHIRALCEL OD-H, 25 °C; *n*-hexane:*i*-PrOH = 75:25, 1.0 mL/min, 254 nm, t_{R1} (major) =8.73 min, t_{R2} (minor) = 19.33 min, 94:6 er.

The absolute stereochemistry was assigned by analogy to compound **3x** and **4k**.

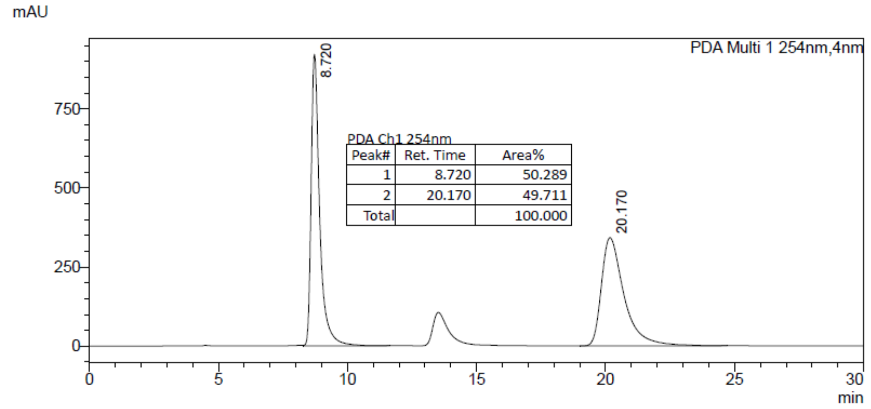

PDA Ch1 254nm

Peak#	Ret. Time	Area%
1	8.720	50.289
2	20.170	49.711
Total		100.000

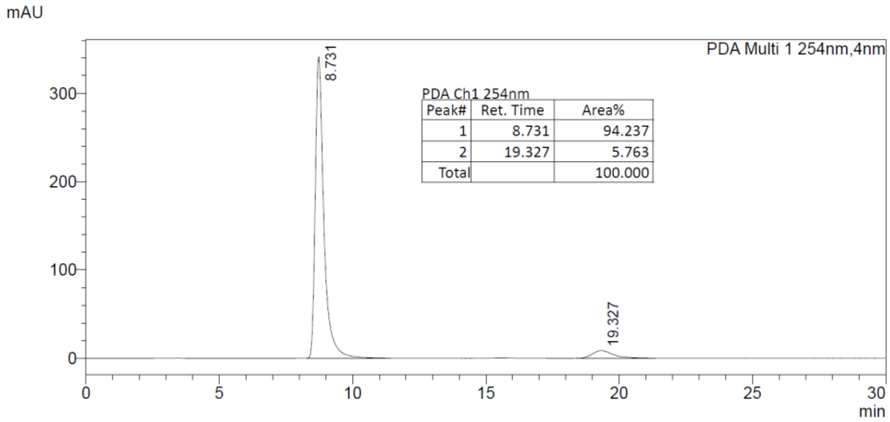

PDA Ch1 254nm

Peak#	Ret. Time	Area%
1	8.731	94.237
2	19.327	5.763
Total		100.000

Supplementary Fig. 37. HPLC traces of *rac*-**3m** (top) and enantioenriched-**3m** (bottom).

400 MHz, ^{1}H NMR in $CDCl_3$

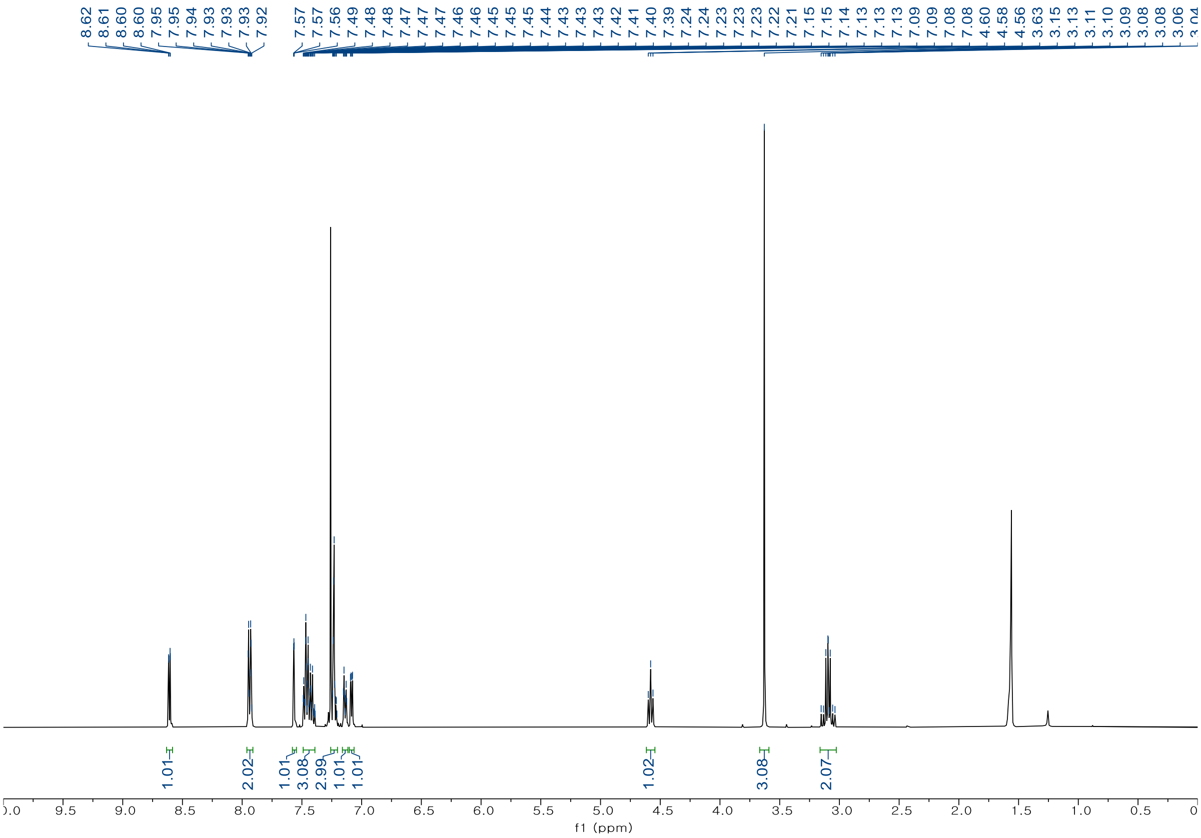

125 MHz, ^{13}C NMR in $CDCl_3$

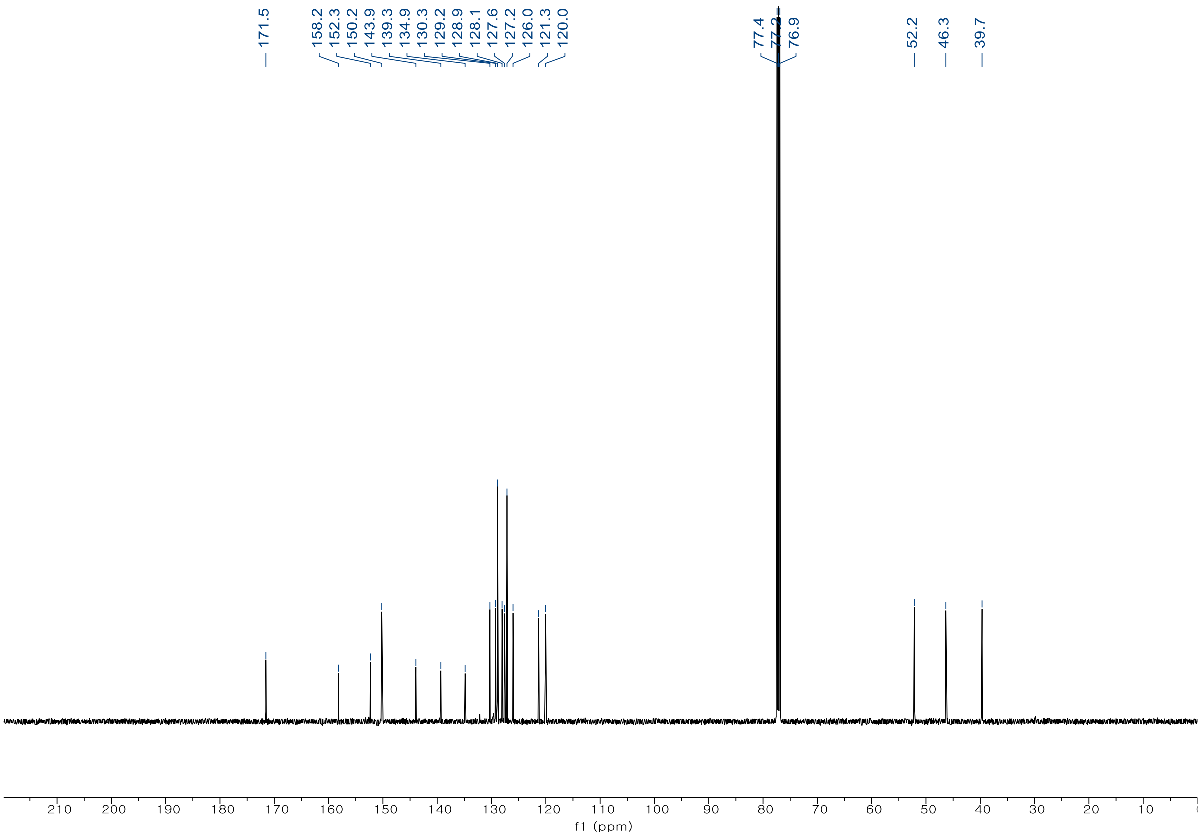

Supplementary Fig. 38. ^{1}H and ^{13}C NMR of **3m**.

Methyl (*R*)-3-(3-nitrophenyl)-3-(2-phenylpyridin-4-yl)propanoate (3n)

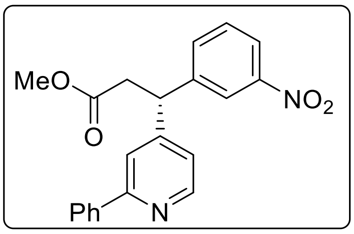

Prepared according to **GP**. Desired product **3n** was obtained as colorless oil (10.5 mg, 58% isolated yield). **^{1}H NMR** (600 MHz, $CDCl_3$) δ 8.63 (d, *J* = 5.1 Hz, 1H), 8.18 – 8.14 (m, 1H), 8.14 – 8.10 (m, 1H), 7.96 – 7.90 (m, 2H), 7.62 – 7.55 (m, 2H), 7.53 – 7.49 (m, 1H), 7.49 – 7.44 (m, 2H), 7.45 – 7.39 (m, 1H), 7.10 (dd, *J* = 5.1, 1.7 Hz, 1H), 4.72 (t, J = 7.8 Hz, 1H), 3.64 (s, 3H), 3.22 – 3.11 (m, 2H). **^{13}C NMR** (100 MHz, $CDCl_3$) δ 171.2, 158.4, 151.6, 150.4, 148.7, 144.0, 139.1, 134.2, 130.1, 129.4, 128.9, 127.1, 122.7, 122.5, 121.2, 119.9, 52.3, 46.3, 39.5. **HRMS** (ESI^+) m/z calcd. For $C_{21}H_{19}N_2O_4^+$ $[M+H]^+$: 363.1345, found 363.1343. **Specific Rotation** $[\alpha]_D^{26}$ +5.5 (*c* 1.0, $CHCl_3$). **HPLC Analysis**. CHIRALCEL OD-H, 25 °C; *n*-hexane:*i*-PrOH = 75:25, 1.0 mL/min, 254 nm, t_{R1} (major) = 15.10 min, t_{R2} (minor) = 41.58 min, 91:9 er.

The absolute stereochemistry was assigned by analogy to compound **3x** and **4k**.

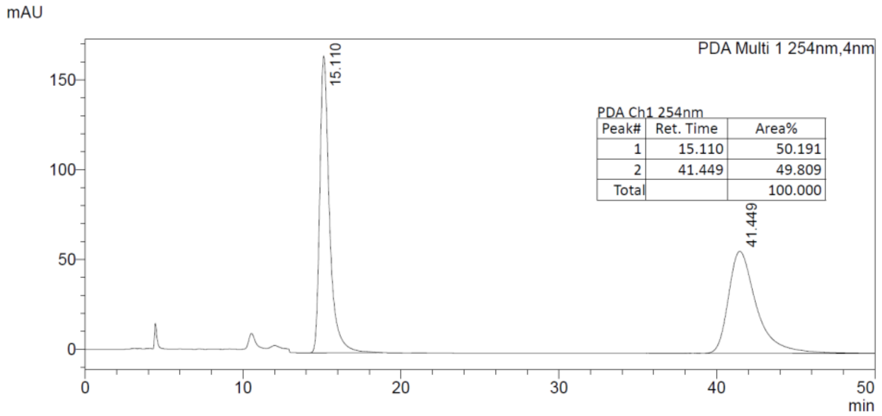

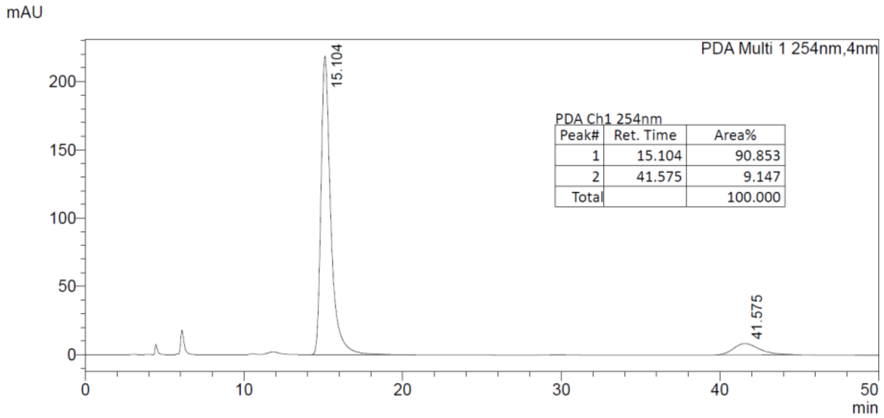

Supplementary Fig. 39. HPLC traces of *rac*-**3n** (top) and enantioenriched-**3n** (bottom).

600 MHz, ^{1}H NMR in CDCl$_3$.

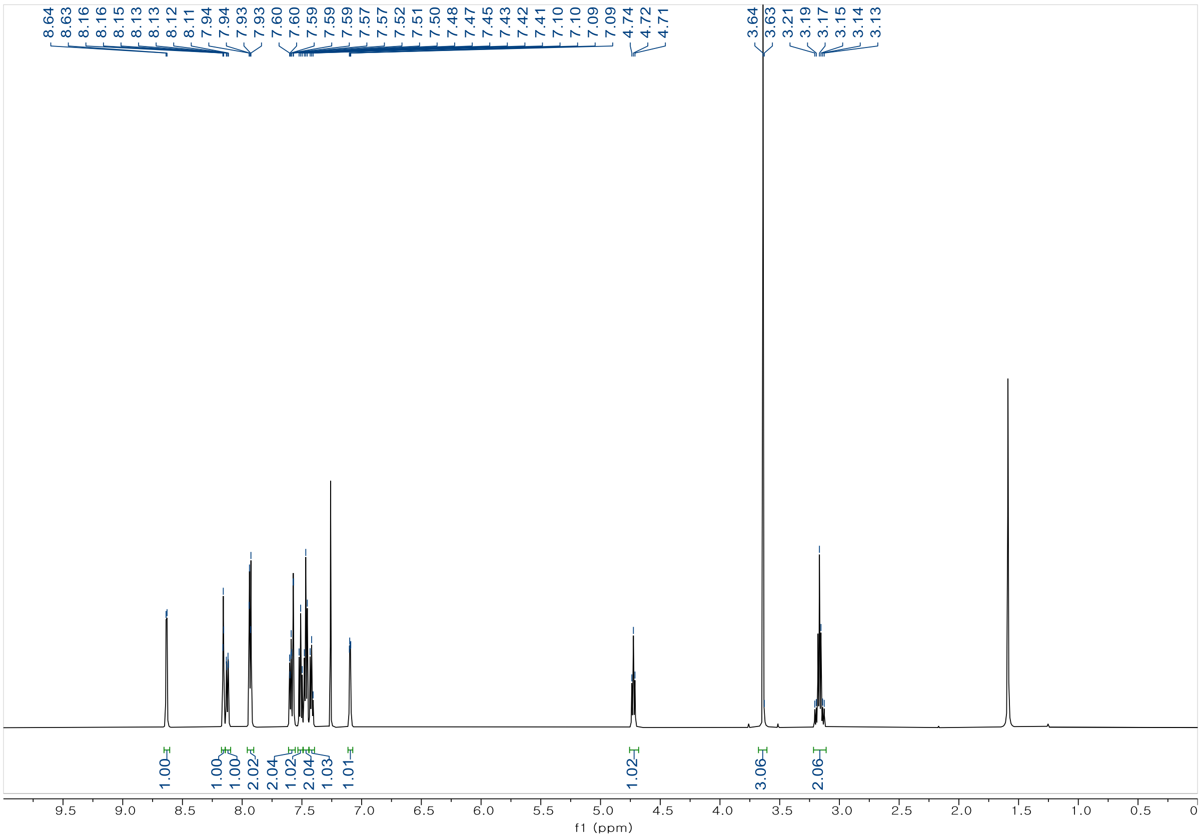

100 MHz, ^{13}C NMR in CDCl$_3$.

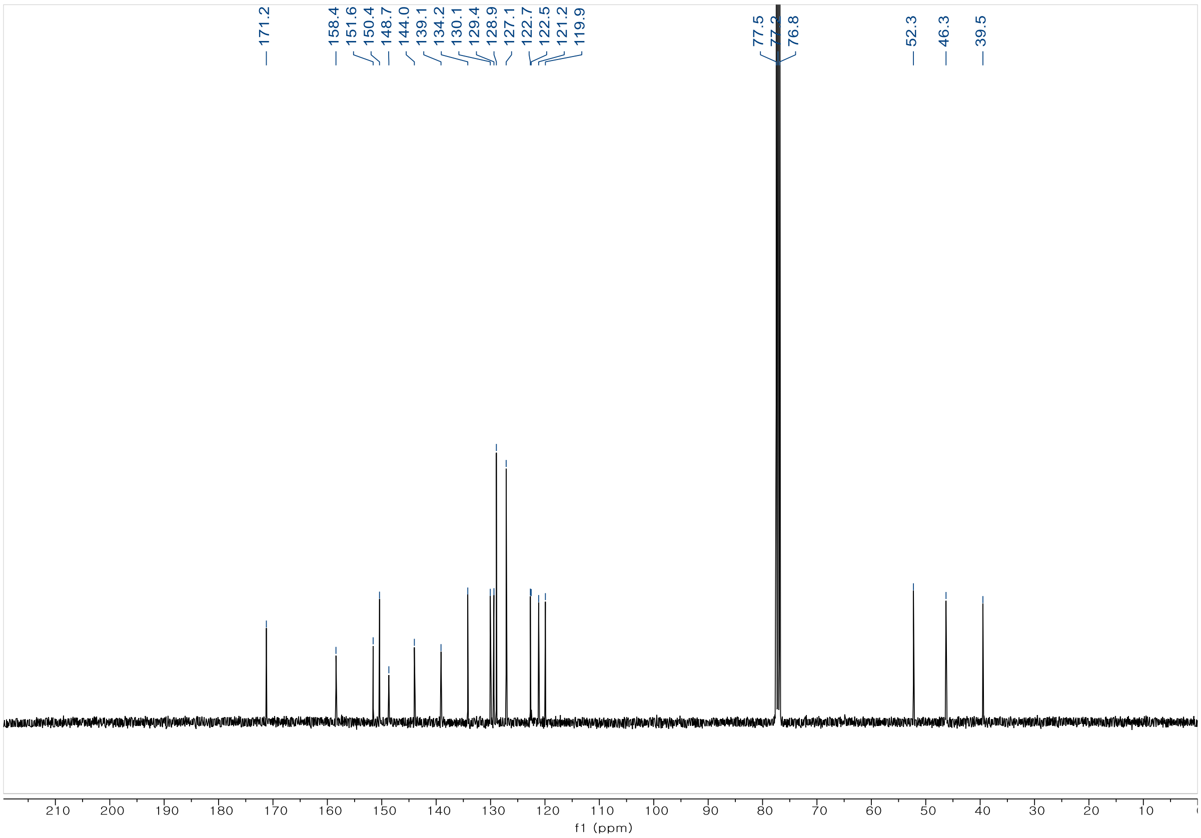

Supplementary Fig. 40. ^{1}H and ^{13}C NMR of **3n**.

Methyl (*S*)-3-(2-phenylpyridin-4-yl)-3-(*o*-tolyl)propanoate (3o)

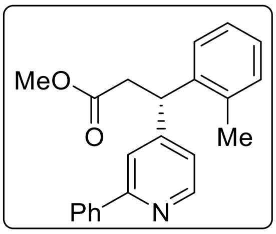

Prepared according to **GP**. Desired product **3o** was obtained as colorless oil (6.7 mg, 40% isolated yield). **^{1}H NMR** (400 MHz, $CDCl_3$) δ 8.56 (dd, *J* = 5.1, 0.8 Hz, 1H), 7.95 – 7.88 (m, 2H), 7.58 – 7.53 (m, 1H), 7.50 – 7.33 (m, 3H), 7.27 – 7.15 (m, 4H), 7.05 (dd, *J* = 5.1, 1.7 Hz, 1H), 4.81 (t, *J* = 7.8 Hz, 1H), 3.62 (s, 3H), 3.11 – 3.05 (m, 2H), 2.33 (s, 3H). **^{13}C NMR** (100 MHz, $CDCl_3$) δ 172.01, 157.88, 153.02, 149.99, 139.72, 139.47, 136.43, 131.14, 129.12, 128.85, 127.28, 127.12, 126.57, 126.51, 121.75, 120.30, 52.05, 42.68, 40.19, 19.97. **HRMS** (ESI$^+$) m/z calcd. For $C_{22}H_{22}NO_2^+$ [M+H]$^+$: 332.1651, found 332.1651. **Specific Rotation** $[\alpha]_D^{26}$ +44.0 (*c* 0.72, $CHCl_3$). **HPLC Analysis**. CHIRALCEL OD-H, 25 °C; *n*-hexane:*i*-PrOH = 75:25, 1.0 mL/min, 254 nm, t_{R1} (major) = 7.35 min, t_{R2} (minor) = 14.06 min, 96:4 er.

The absolute stereochemistry was assigned by analogy to compound **3x** and **4k**.

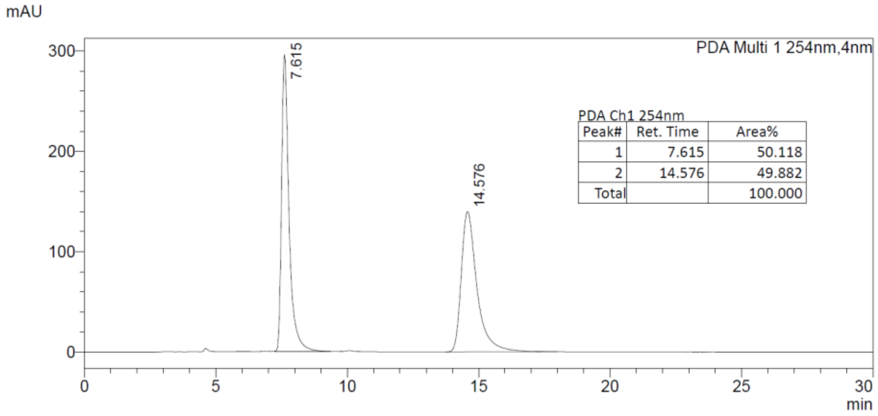

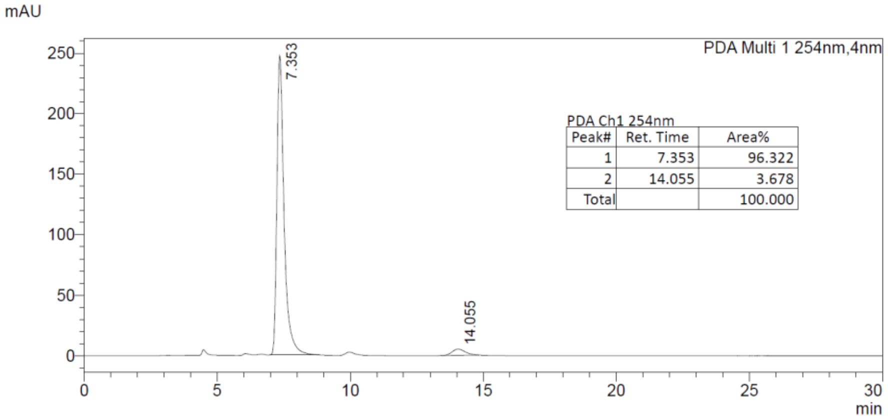

Supplementary Fig. 41. HPLC traces of *rac*-**3o** (top) and enantioenriched-**3o** (bottom).

400 MHz, ^{1}H NMR in $CDCl_3$.

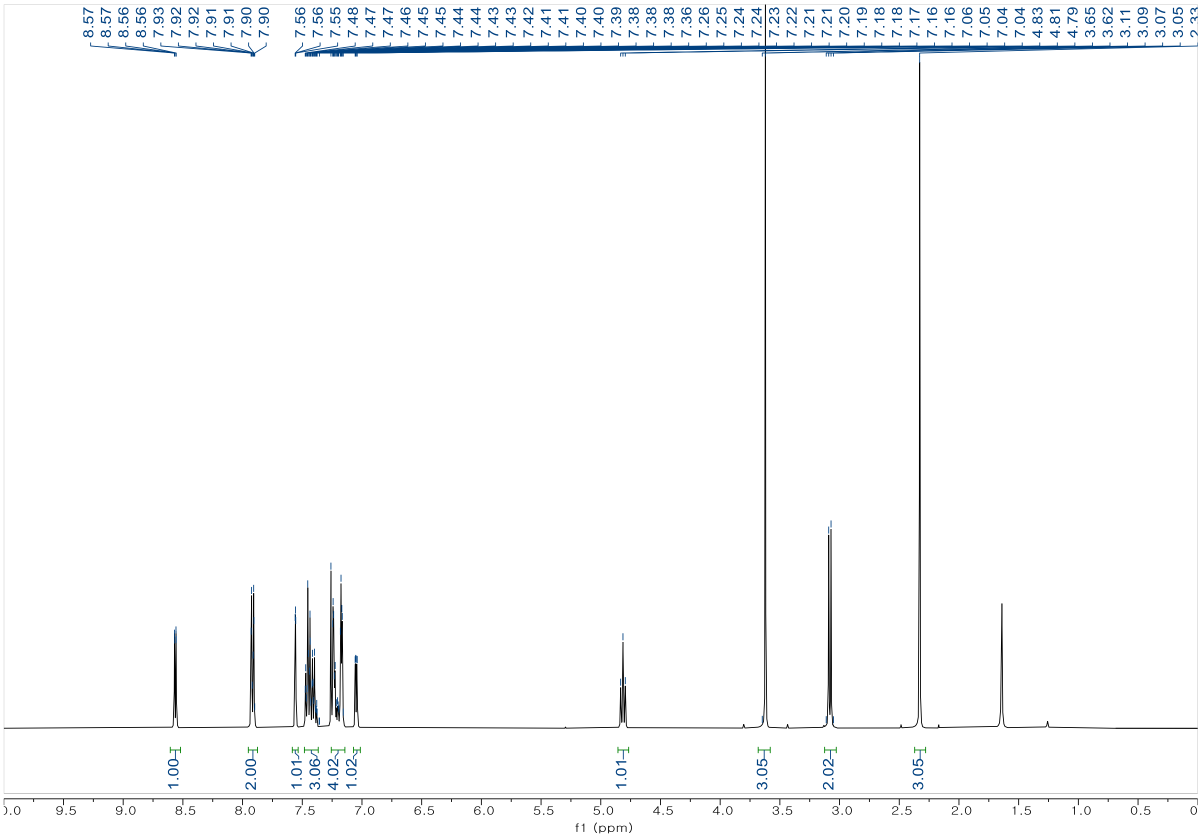

100 MHz, ^{13}C NMR in $CDCl_3$.

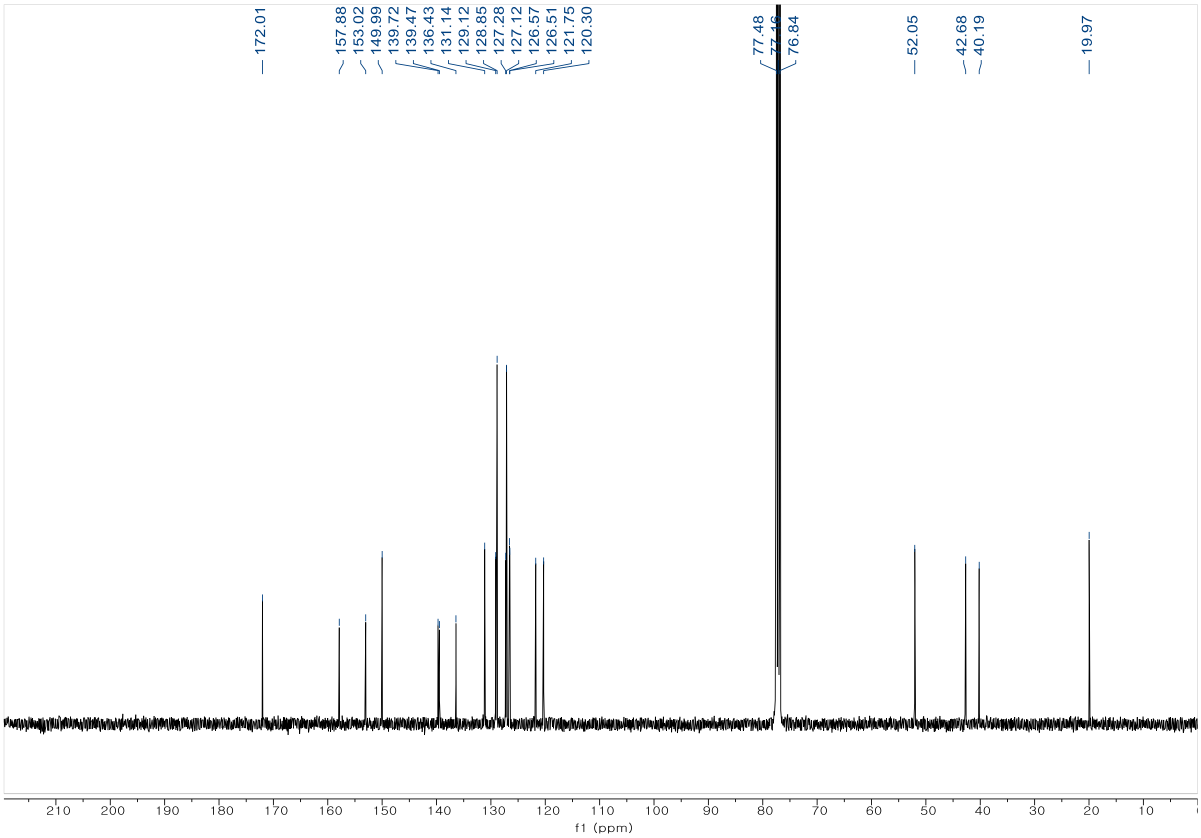

Supplementary Fig. 42. ^{1}H and ^{13}C NMR of **3o**.

Methyl (*S*)-3-(2-methoxyphenyl)-3-(2-phenylpyridin-4-yl)propanoate (3p)

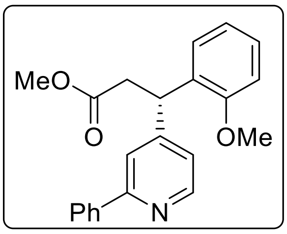

Prepared according to **GP**. Desired product **3p** was obtained as colorless oil (10.6 mg, 61% isolated yield). **^{1}H NMR** (400 MHz, $CDCl_3$) δ 8.56 (dd, *J* = 5.1, 0.8 Hz, 1H), 7.96 – 7.90 (m, 2H), 7.63 – 7.60 (m, 1H), 7.48 – 7.42 (m, 2H), 7.42 – 7.36 (m, 1H), 7.23 (ddd, *J* = 8.2, 7.4, 1.7 Hz, 1H), 7.17 (dd, *J* = 7.7, 1.7 Hz, 1H), 7.12 (ddd, *J* = 5.1, 1.7, 0.6 Hz, 1H), 6.93 (td, *J* = 7.5, 1.1 Hz, 1H), 6.86 (dd, *J* = 8.2, 1.1 Hz, 1H), 4.96 (t, *J* = 7.9 Hz, 1H), 3.79 (s, 3H), 3.61 (s, 3H), 3.14 – 3.05 (m, 2H). **^{13}C NMR** (100 MHz, $CDCl_3$) δ 172.2, 157.6, 157.0, 153.2, 149.7, 139.7, 130.3, 129.0, 128.8, 128.5, 127.9, 127.1, 121.8, 120.8, 120.4, 111.1, 55.5, 51.9, 40.5, 38.6. **HRMS** (ESI$^+$) m/z calcd. For $C_{22}H_{22}NO_3^+$ $[M+H]^+$: 348.1600, found 348.1601. **Specific Rotation** $[\alpha]_D^{26}$ –22.9 (*c* 1.0, $CHCl_3$). **HPLC Analysis**. CHIRALCEL OD-H, 25 °C; *n*-hexane:*i*-PrOH = 75:25, 1.0 mL/min, 254 nm, t_{R1} (major) = 6.90 min, t_{R2} (minor) = 19.77 min, 96:4 er.

The absolute stereochemistry was assigned by analogy to compound **3x** and **4k**.

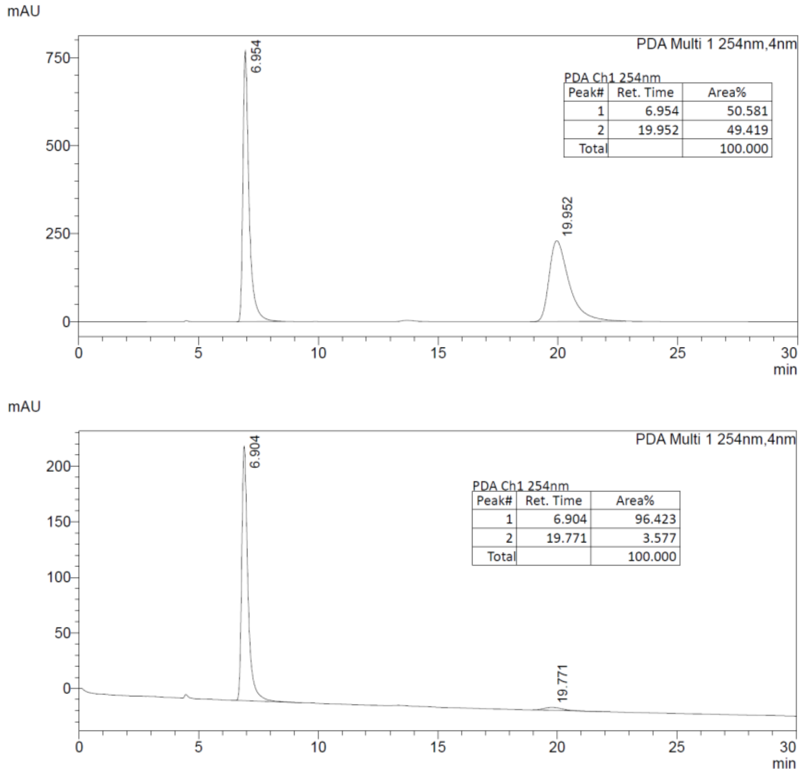

Supplementary Fig. 43. HPLC traces of *rac*-**3p** (top) and enantioenriched-**3p** (bottom).

400 MHz, ^{1}H NMR in $CDCl_3$.

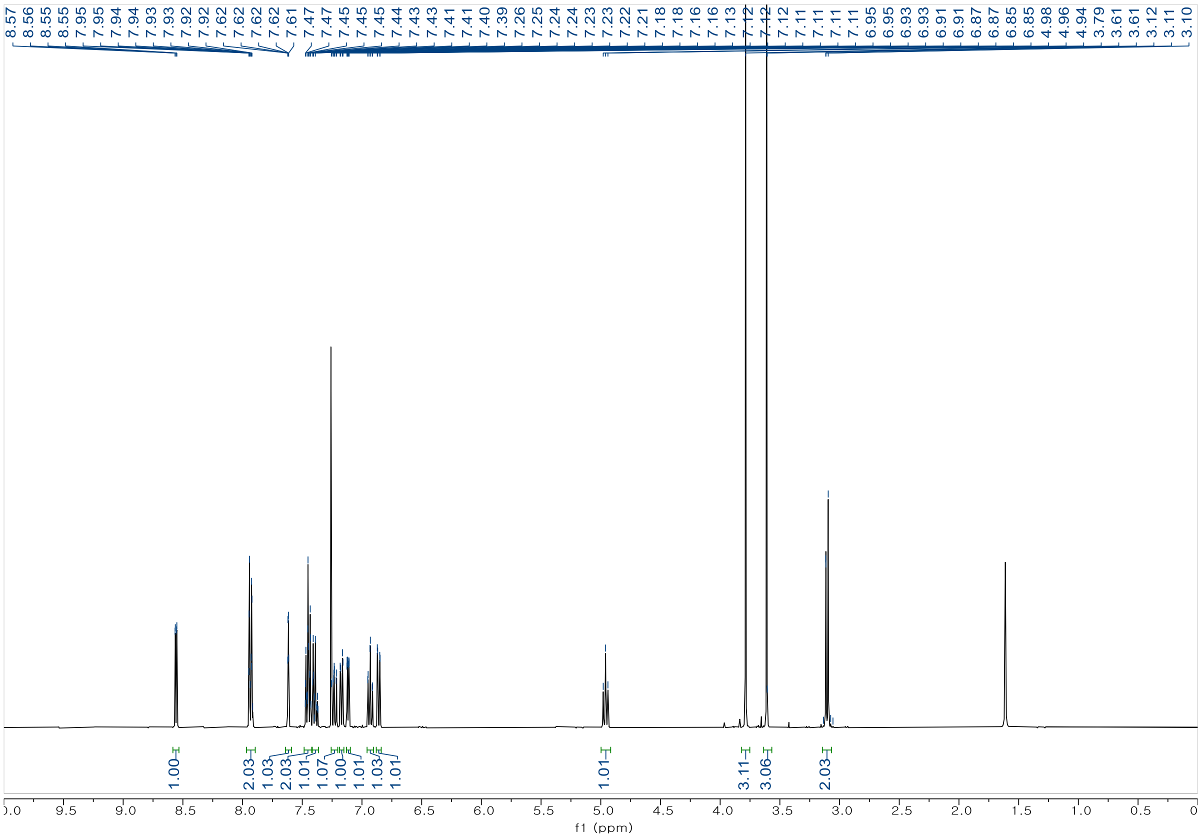

100 MHz, ^{13}C NMR in $CDCl_3$.

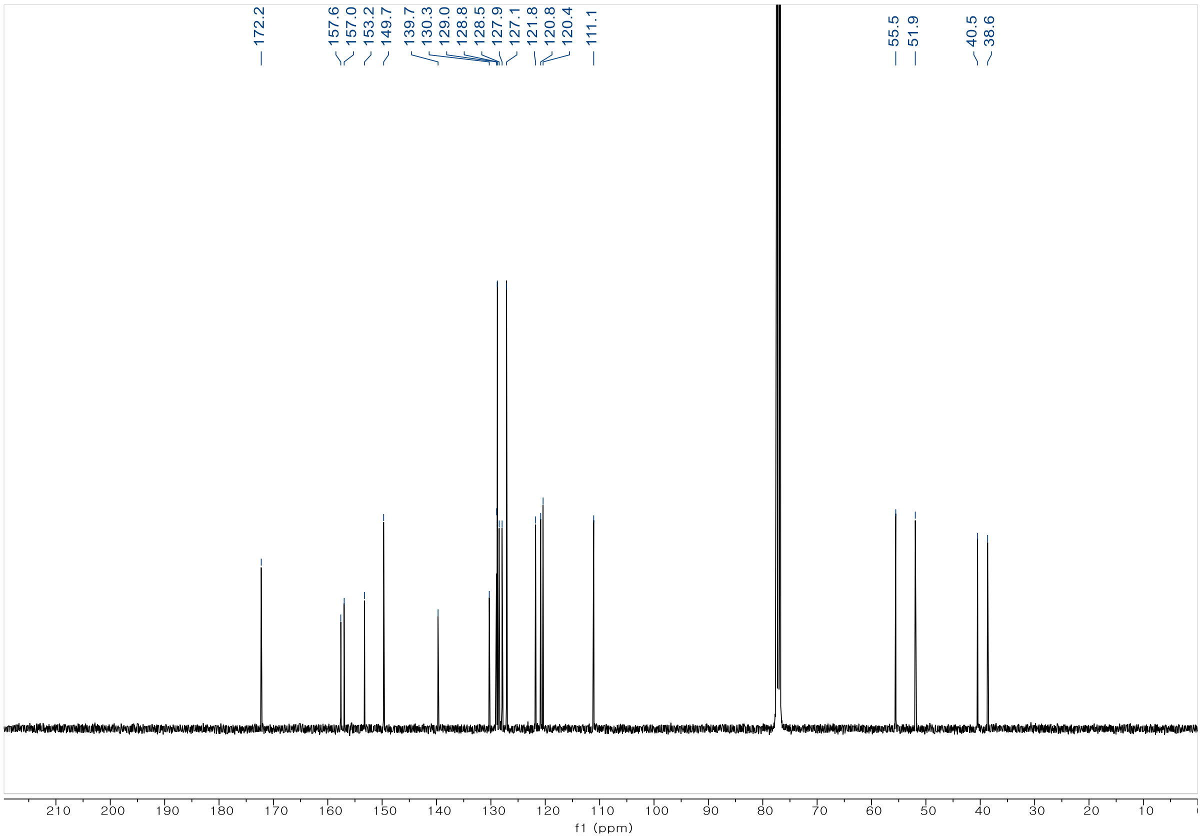

Supplementary Fig. 44. ^{1}H and ^{13}C NMR of **3p**.

Methyl (*S*)-3-(2-chlorophenyl)-3-(2-phenylpyridin-4-yl)propanoate (3q)

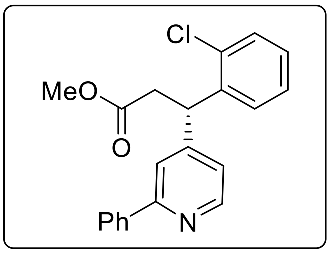

Prepared according to **GP**. Desired product **3q** was obtained as colorless oil (11.3 mg, 57% isolated yield). **^{1}H NMR** (400 MHz, $CDCl_3$) δ 8.59 (dd, *J* = 5.1, 0.8 Hz, 1H), 7.97 – 7.90 (m, 2H), 7.61 (s, 1H), 7.50 – 7.36 (m, 4H), 7.27 – 7.26 (m, 1H), 7.25 – 7.17 (m, 2H), 7.10 (dd, *J* = 5.2, 1.7 Hz, 1H), 5.13 (t, *J* = 7.9 Hz, 1H), 3.63 (s, 3H), 3.11 (d, *J* = 7.9 Hz, 2H). **^{13}C NMR** (125 MHz, $CDCl_3$) δ 171.5, 158.0, 151.8, 150.0, 139.4, 139.3, 134.2, 130.3, 129.1, 128.9, 128.6, 128.4, 127.4, 127.1, 121.6, 120.4, 52.1, 42.9, 39.2. **HRMS** (ESI^+) m/z calcd. For $C_{21}H_{19}NO_2Cl^+$ $[M+H]^+$: 352.1099, found 352.1103. **Specific Rotation** $[\alpha]_D^{26}$ -11.5 (*c* 1.0, $CHCl_3$). **HPLC Analysis**. CHIRALCEL OD-H, 25 °C; *n*-hexane:*i*-PrOH = 75:25, 1.0 mL/min, 254 nm, t_{R1} (major) =8.12 min, t_{R2} (minor) = 15.66 min, 96:4 er.

The absolute stereochemistry was assigned by analogy to compound **3x** and **4k**.

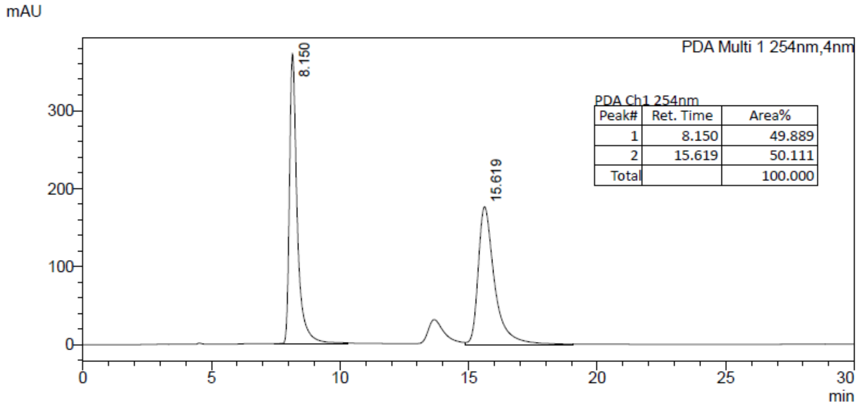

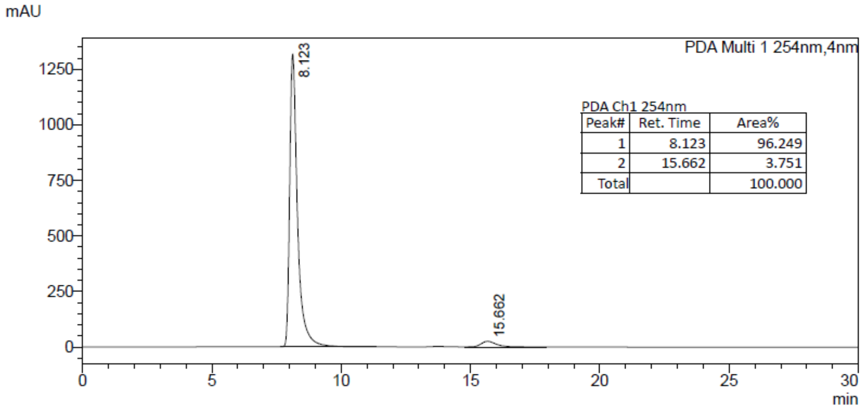

Supplementary Fig. 45. HPLC traces of *rac*-**3q** (top) and enantioenriched-**3q** (bottom).

400 MHz, ^{1}H NMR in $CDCl_3$.

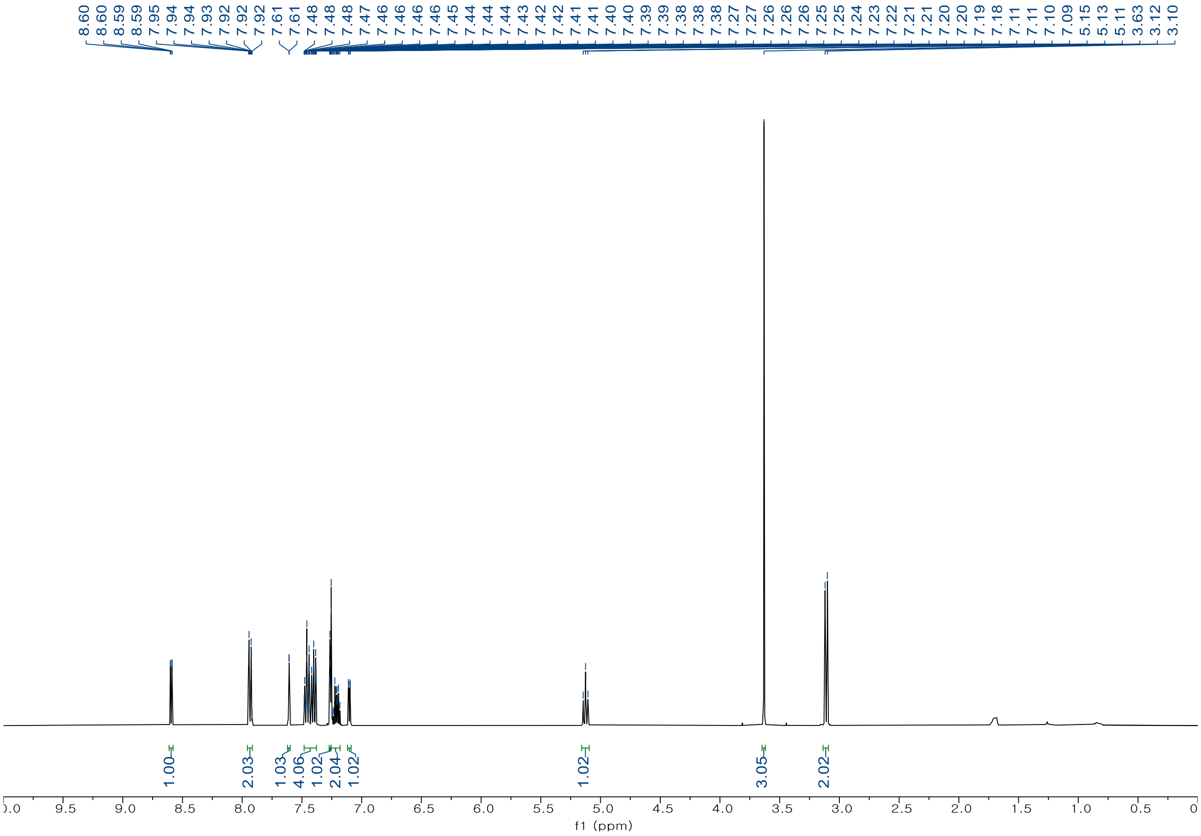

125 MHz, ^{13}C NMR in $CDCl_3$.

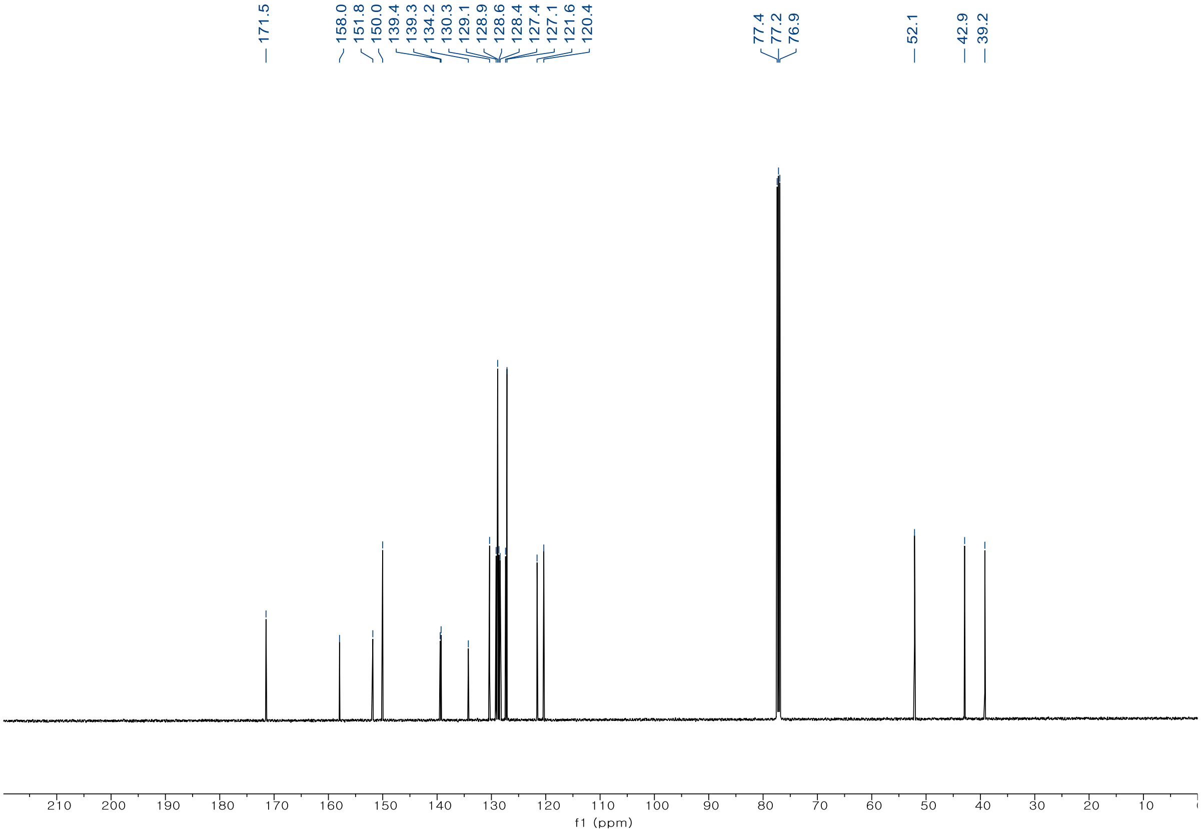

Supplementary Fig. 46. ^{1}H and ^{13}C NMR of **3q**.

Methyl (*S*)-3-(2-bromophenyl)-3-(2-phenylpyridin-4-yl)propanoate (3r)

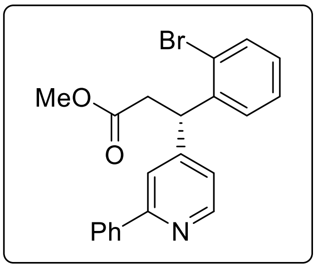

Prepared according to **GP**. Desired product **3r** was obtained as colorless oil (10.5 mg, 60% isolated yield). **^{1}H NMR** (600 MHz, $CDCl_3$) δ 8.60 (d, *J* = 5.1 Hz, 1H), 7.93 (d, *J* = 7.5 Hz, 2H), 7.62 (s, 1H), 7.59 (d, *J* = 8.0 Hz, 1H), 7.46 (t, *J* = 7.5 Hz, 2H), 7.40 (t, *J* = 7.3 Hz, 1H), 7.30 (t, *J* = 7.6 Hz, 1H), 7.25 – 7.21 (m, 1H), 7.15 – 7.09 (m, 2H), 5.12 (t, *J* = 7.9 Hz, 1H), 3.63 (s, 3H), 3.10 (d, *J* = 7.9 Hz, 2H). **^{13}C NMR** (125 MHz, $CDCl_3$) δ 171.5, 158.0, 151.8, 150.0, 140.9, 139.4, 133.7, 129.2, 128.9, 128.9, 128.6, 128.1, 127.2, 125.1, 121.6, 120.4, 52.2, 45.4, 39.5. **HRMS** (ESI$^+$) m/z calcd. For $C_{21}H_{19}NO_2Br^+$ $[M+H]^+$: 396.0599, found 396.0599. **Specific Rotation** $[\alpha]_D^{25}$ -16.2 (*c* 0.5, $CHCl_3$). **HPLC Analysis**. CHIRALCEL OD-H, 25 °C; *n*-hexane:*i*-PrOH = 75:25, 1.0 mL/min, 254 nm, t_{R1} (major) =8.48 min, t_{R2} (minor) = 16.31 min, 96:4 er.

The absolute stereochemistry was assigned by analogy to compound **3x** and **4k**.

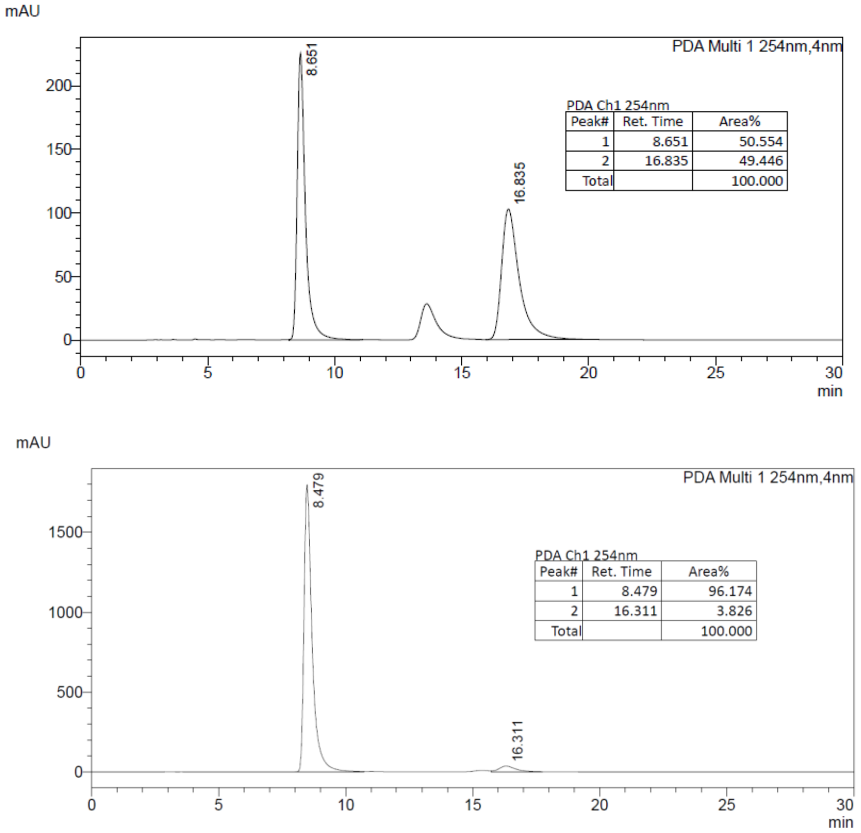

Supplementary Fig. 47. HPLC traces of *rac*-**3r** (top) and enantioenriched-**3r** (bottom).

600 MHz, ^{1}H NMR in $CDCl_3$

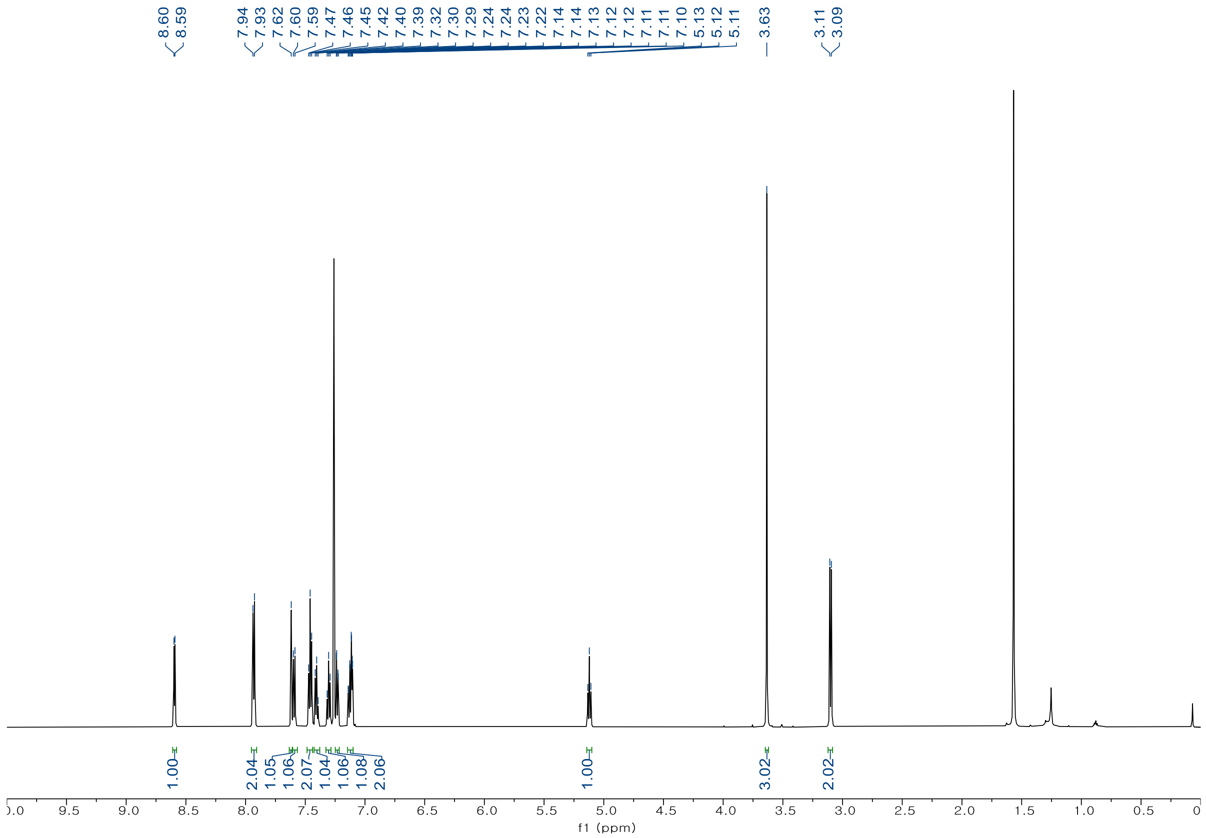

125 MHz, ^{13}C NMR in $CDCl_3$

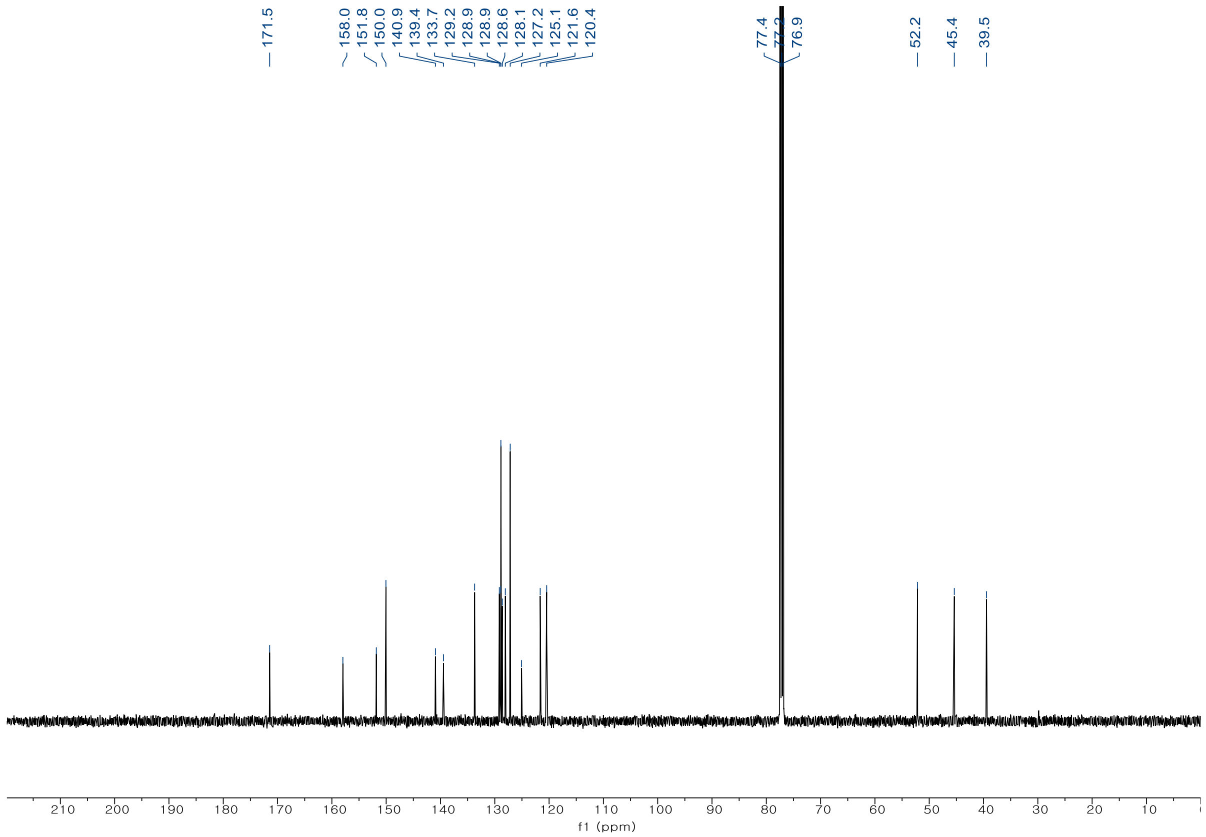

Supplementary Fig. 48. ^{1}H and ^{13}C NMR of **3r**.

Methyl (*S*)-3-(4-acetoxy-3-methoxyphenyl)-3-(2-phenylpyridin-4-yl)propanoate (3s)

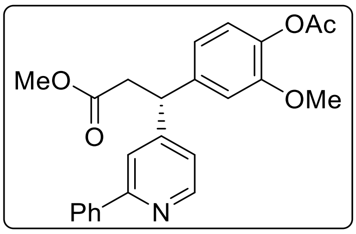

Prepared according to **GP**. Desired product **3s** was obtained as colorless oil (13.4 mg, 66% isolated yield). **^{1}H NMR** (400 MHz, $CDCl_3$) δ NMR 8.63 – 8.57 (m, 1H), 7.98 – 7.90 (m, 2H), 7.62 – 7.55 (m, 1H), 7.51 – 7.42 (m, 2H), 7.45 – 7.36 (m, 1H), 7.10 (dd, *J* = 5.2, 1.7 Hz, 1H), 7.02 – 6.95 (m, 1H), 6.85 – 6.79 (m, 2H), 4.60 (d, *J* = 7.8 Hz, 1H), 3.78 (s, 3H), 3.63 (s, 3H), 3.17 – 3.01 (m, 2H), 2.29 (s, 3H). **^{13}C NMR** (100 MHz, $CDCl_3$) δ 171.8, 169.1, 158.1, 152.7, 151.4, 150.1, 140.8, 139.4, 138.9, 129.2, 128.9, 127.1, 123.2, 121.4, 120.1, 119.7, 112.3, 56.0, 52.1, 46.5, 40.1, 20.8. **HRMS** (ESI$^+$) m/z calcd. For $C_{24}H_{24}NO_5^+$ [M+H]$^+$: 406.1654, found 406.1653. **Specific Rotation** $[\alpha]_D^{26}$ –7.0 (*c* 0.89, $CHCl_3$). **HPLC Analysis**. CHIRALCEL OD-H, 25 °C; *n*-hexane:*i*-PrOH = 75:25, 1.0 mL/min, 254 nm, t_{R1} (major) = 12.95 min, t_{R2} (minor) = 29.53 min, 91:9 er.

The absolute stereochemistry was assigned by analogy to compound **3x** and **4k**.

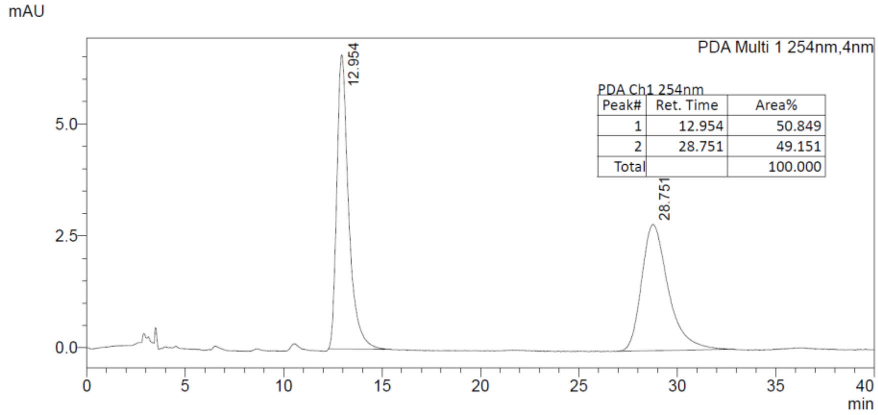

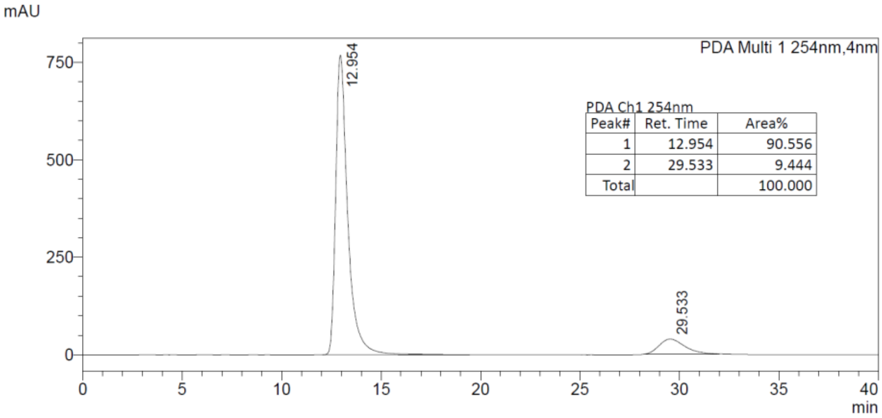

Supplementary Fig. 49. HPLC traces of *rac*-**3s** (top) and enantioenriched-**3s** (bottom).

400 MHz, ^{1}H NMR in CDCl$_3$.

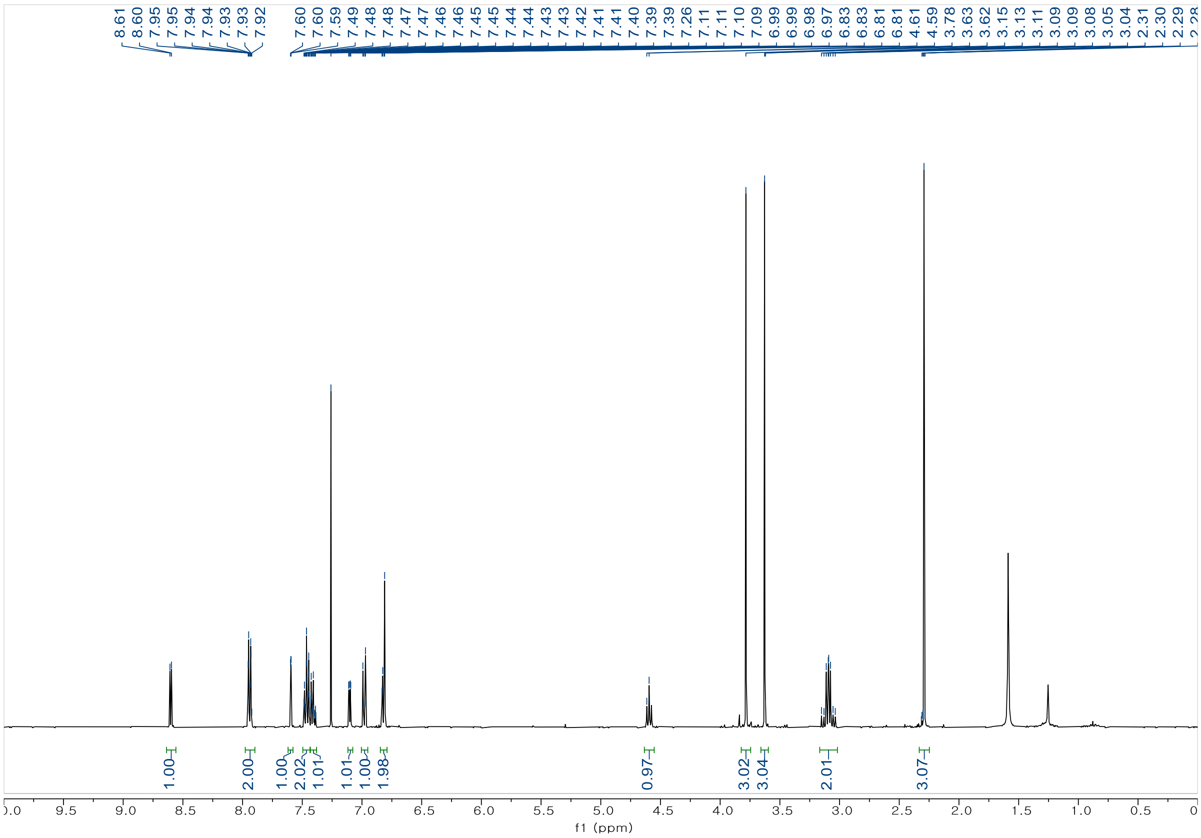

100 MHz, ^{13}C NMR in CDCl$_3$.

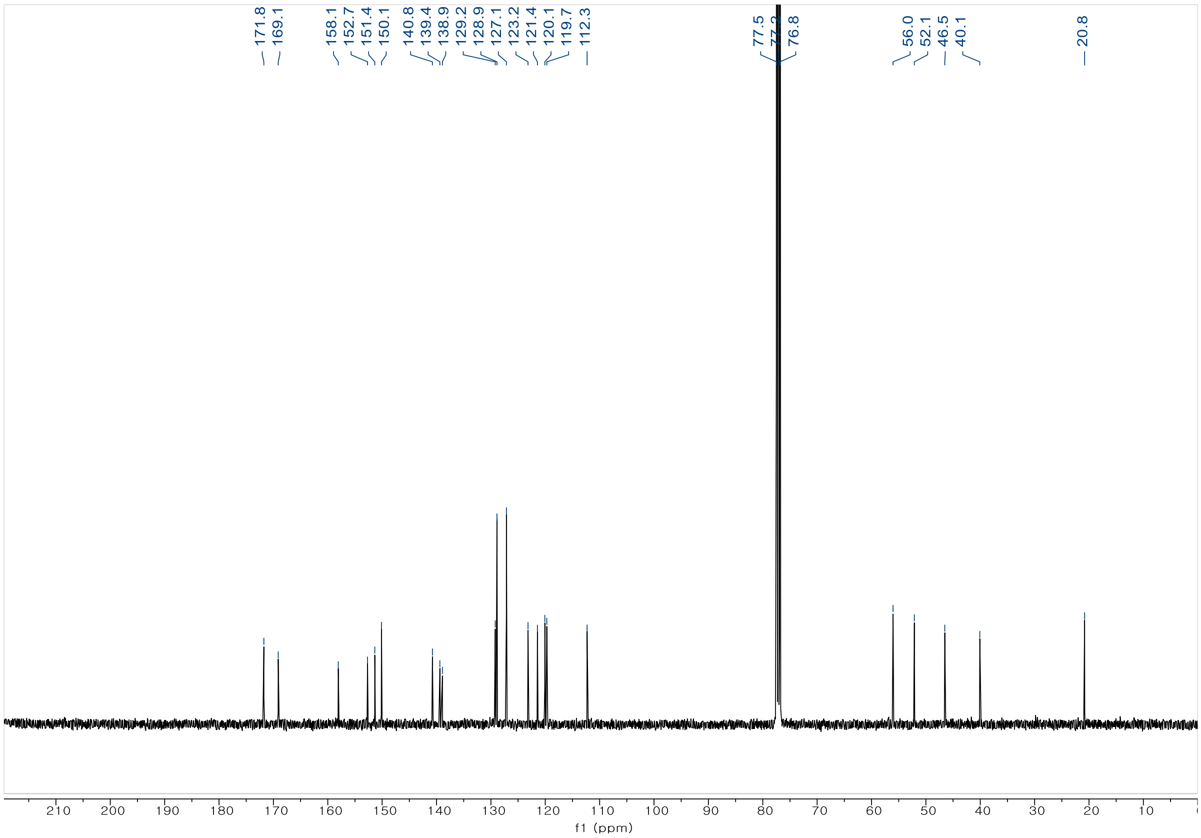

Supplementary Fig. 50. ^{1}H and ^{13}C NMR of **3s**.

Methyl (*S*)-3-(furan-2-yl)-3-(2-phenylpyridin-4-yl)propanoate (3t)

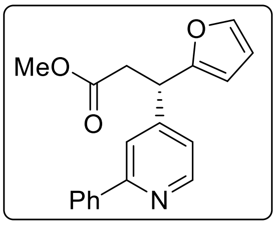

Prepared according to **GP**. Desired product **3t** was obtained as colorless oil (8.7 mg, 57% isolated yield). **^{1}H NMR** (400 MHz, $CDCl_3$) δ 8.65 – 8.59 (m, 1H), 7.99 – 7.91 (m, 2H), 7.64 – 7.59 (m, 1H), 7.50 – 7.44 (m, 2H), 7.44 – 7.38 (m, 1H), 7.37 – 7.33 (m, 1H), 7.13 (dd, J = 5.1, 1.7 Hz, 1H), 6.32 (dd, J = 3.3, 1.9 Hz, 1H), 6.18 – 6.10 (m, 1H), 4.66 – 4.58 (m, 1H), 3.64 (s, 3H), 3.15 (dd, *J* = 16.0, 7.5 Hz, 1H), 2.98 (dd, *J* = 16.0, 8.1 Hz, 1H). **^{13}C NMR** (100 MHz, $CDCl_3$) δ 171.4, 158.1, 154.4, 150.9, 150.1, 142.4, 139.4, 129.2, 128.9, 127.1, 121.5, 120.1, 110.5, 106.6, 52.1, 41.0, 38.8. **HRMS** (ESI$^+$) m/z calcd. For $C_{19}H_{18}NO_3^+$ [M+H]$^+$: 308.1287, found 308.1286. **Specific Rotation** $[\alpha]_D^{20}$ +60.8 (*c* 1.0, $CHCl_3$). **HPLC Analysis**. CHIRALCEL OD-H, 25 ºC; *n*-hexane:*i*-PrOH = 75:25, 1.0 mL/min, 254 nm, t_{R1} (major) = 7.12 min, t_{R2} (minor) = 9.41 min, 96:4 er.

The absolute stereochemistry was assigned by analogy to compound **3x** and **4k**.

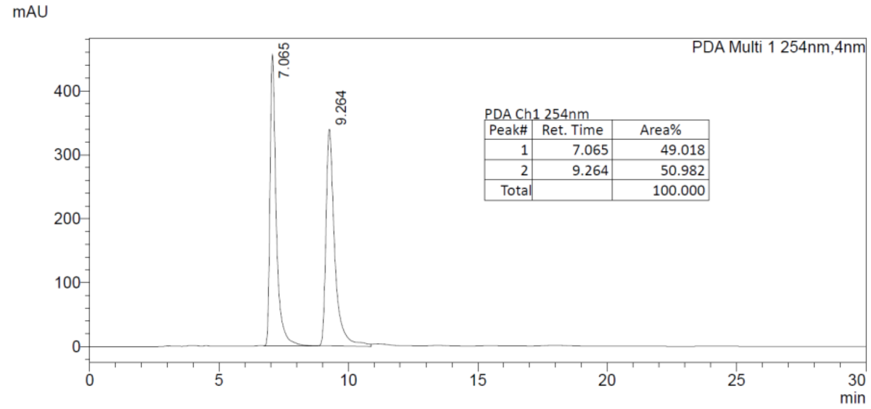

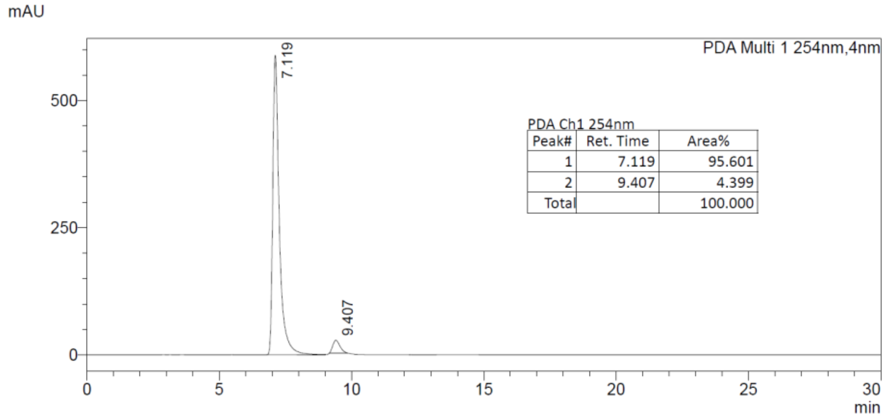

Supplementary Fig. 51. HPLC traces of *rac*-**3t** (top) and enantioenriched-**3t** (bottom).

400 MHz, ¹H NMR in $CDCl_3$.

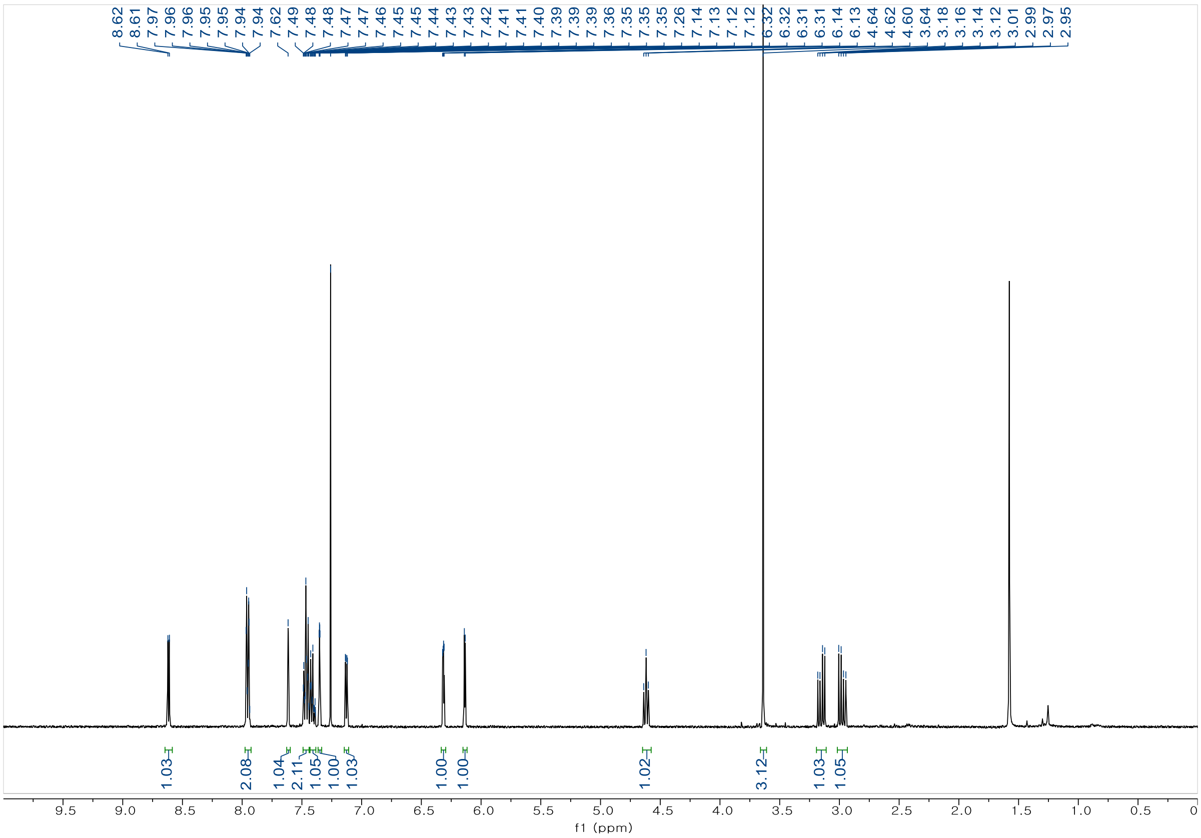

100 MHz, ¹³C NMR in $CDCl_3$.

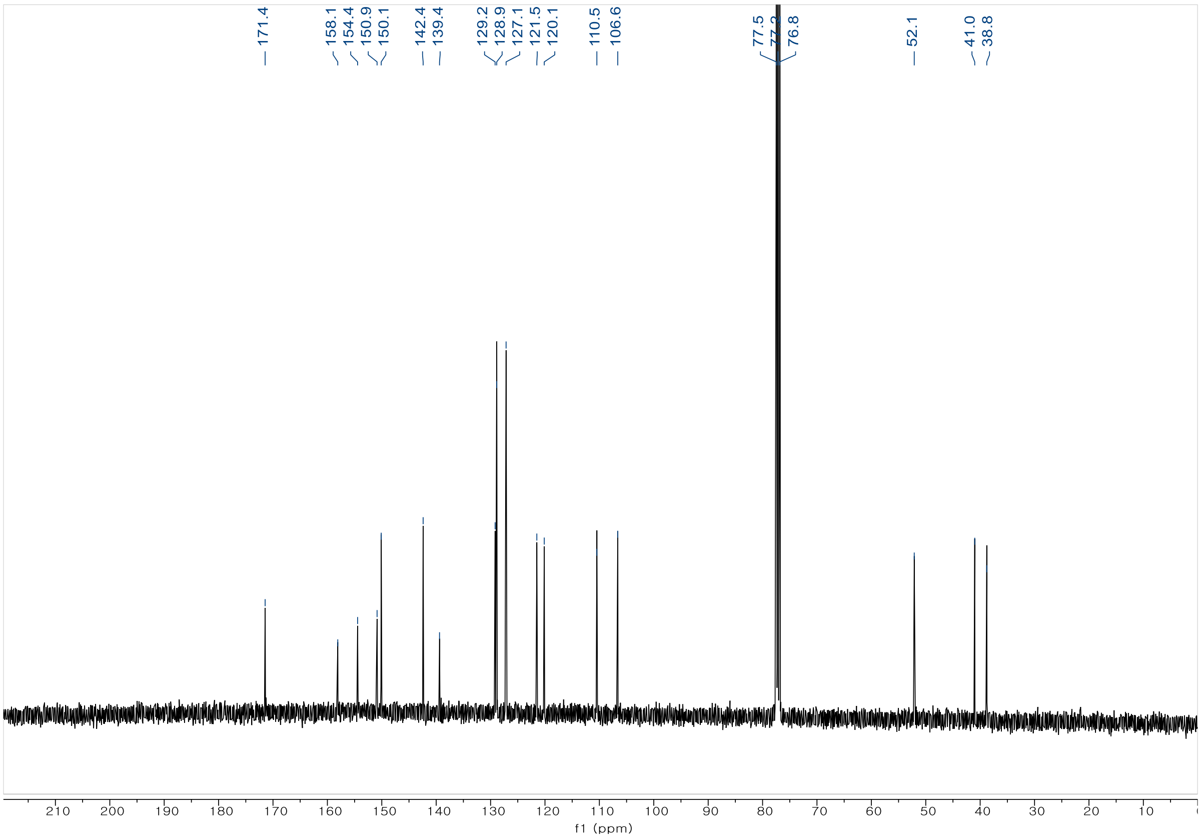

Supplementary Fig. 52. ¹H and ¹³C NMR of **3t**.

Methyl (*S*)-3-(2-phenylpyridin-4-yl)-3-(thiophen-2-yl)propanoate (3u)

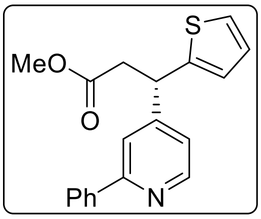

Prepared according to **GP**. Desired product **3u** was obtained as colorless oil (9.7 mg, 60% isolated yield). **^{1}H NMR** (400 MHz, $CDCl_3$) δ 8.65 – 8.59 (m, 1H), 7.99 – 7.92 (m, 2H), 7.67 – 7.62 (m, 1H), 7.49 – 7.44 (m, 2H), 7.43 – 7.38 (m, 1H), 7.20 (dd, *J* = 5.1, 1.2 Hz, 1H), 7.16 (dd, *J* = 5.2, 1.7 Hz, 1H), 6.94 (dd, *J* = 5.1, 3.5 Hz, 1H), 6.90 (dt, *J* = 3.5, 1.1 Hz, 1H), 4.88 – 4.80 (m, 1H), 3.63 (s, 3H), 3.25 – 3.05 (m, 2H). **^{13}C NMR** (150 MHz, $CDCl_3$) δ 171.3, 158.1, 152.6, 150.2, 145.3, 139.4, 129.2, 128.9, 127.1, 127.1, 124.9, 124.8, 121.2, 119.9, 52.1, 42.3, 41.3. **HRMS** (ESI^+) m/z calcd. For $C_{19}H_{18}NO_2S^+$ $[M+H]^+$: 324.1058, found 324.1059. **Specific Rotation** $[\alpha]_D^{26}$ +14.82 (*c* 1.0, $CHCl_3$). **HPLC Analysis**. CHIRALCEL OD-H, 25 ºC; *n*-hexane:*i*-PrOH = 75:25, 1.0 mL/min, 254 nm, t_{R1} (major) = 7.78 min, t_{R2} (minor) = 15.00 min, 95:5 er.

The absolute stereochemistry was assigned by analogy to compound **3x** and **4k**.

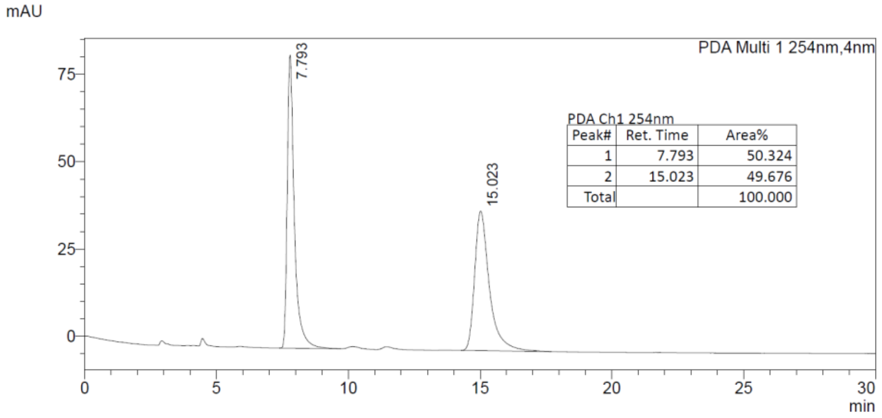

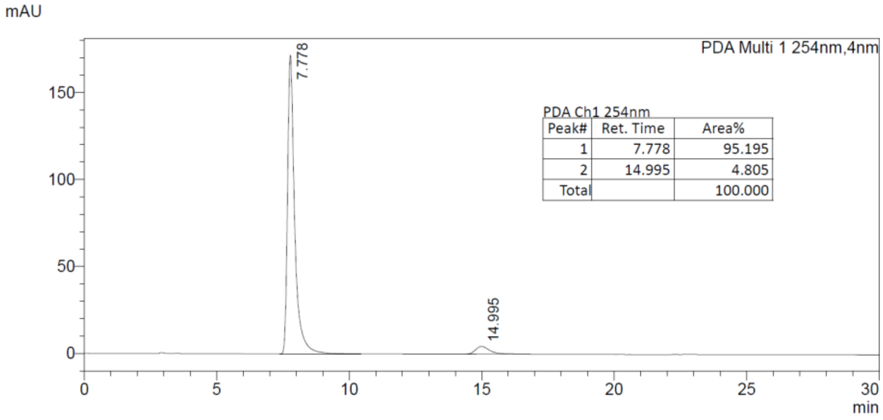

Supplementary Fig. 53. HPLC traces of *rac*-**3u** (top) and enantioenriched-**3u** (bottom).

400 MHz, ^{1}H NMR in CDCl$_3$.

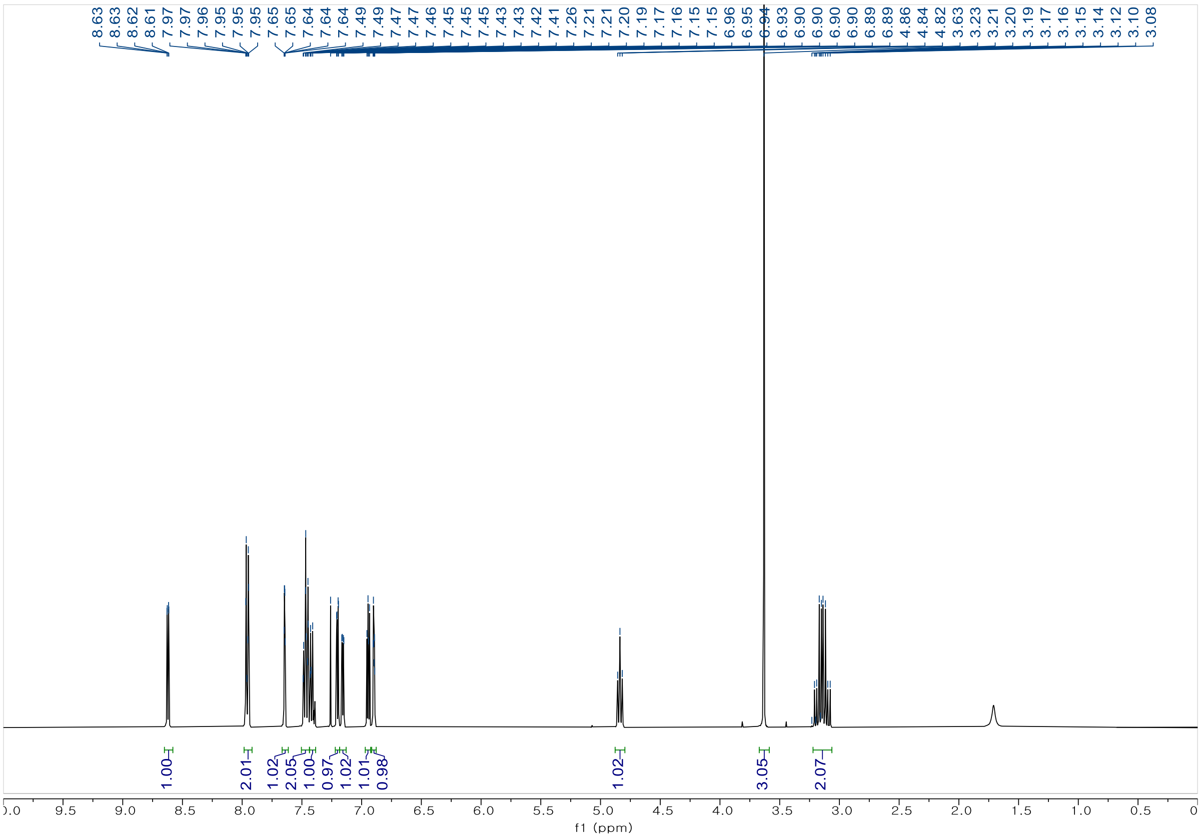

150 MHz, ^{13}C NMR in CDCl$_3$.

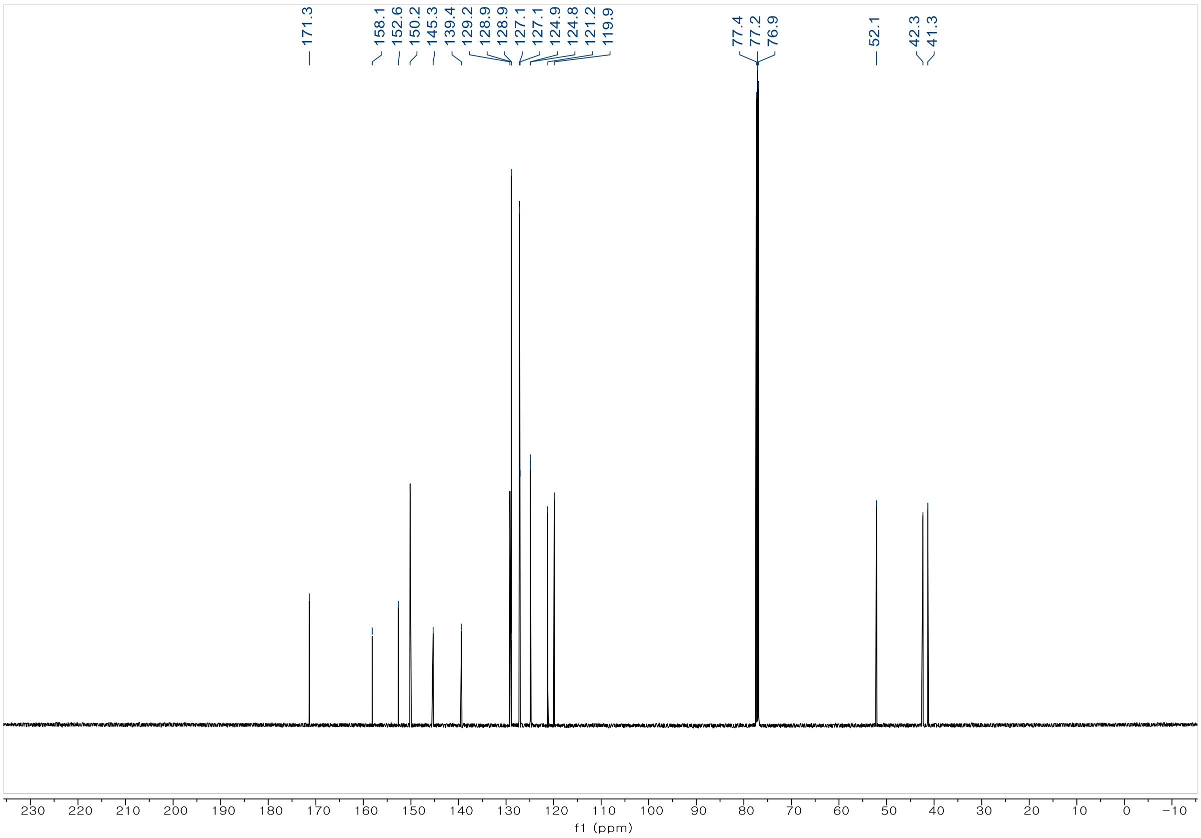

Supplementary Fig. 54. ^{1}H and ^{13}C NMR of **3u**.

Methyl (*S*)-3-(naphthalen-1-yl)-3-(2-phenylpyridin-4-yl)propanoate (3v)

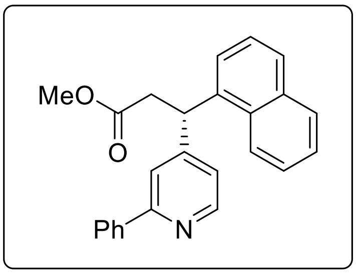

Prepared according to **GP**. Desired product **3v** was obtained as colorless oil (9.8 mg, 53% isolated yield). **^{1}H NMR** (400 MHz, $CDCl_3$) δ 8.57 (dd, *J* = 5.1, 0.8 Hz, 1H), 8.16 – 8.06 (m, 1H), 7.93 – 7.90 (m, 2H), 7.87 (dd, *J* = 6.8, 2.7 Hz, 1H), 7.80 (d, *J* = 8.3 Hz, 1H), 7.68 – 7.66 (m, 1H), 7.55 – 7.36 (m, 7H), 7.14 (dd, *J* = 5.2, 1.7 Hz, 1H), 5.45 (t, *J* = 7.7 Hz, 1H), 3.64 (s, 3H), 3.29 – 3.16 (m, 2H). **^{13}C NMR** (100 MHz, $CDCl_3$) δ 172.0, 158.0, 153.2, 150.1, 139.4, 137.5, 134.3, 131.4, 129.2, 129.1, 128.8, 128.2, 127.1, 126.7, 126.0, 125.5, 124.6, 123.4, 121.7, 120.2, 52.1, 42.3, 40.4. **HRMS** (EI^+) m/z calcd. For $C_{25}H_{21}NO_2^+$ $[M]^+$: 367.1572, found 367.1574. **Specific Rotation** $[\alpha]_D^{25}$ -4.36 (*c* 1.0, $CHCl_3$). **HPLC Analysis**. CHIRALCEL OD-H, 25 °C; *n*-hexane:*i*-PrOH = 75:25, 1.0 mL/min, 254 nm, t_{R1} (major) =12.29 min, t_{R2} (minor) = 22.71 min, 93:7 er.

The absolute stereochemistry was assigned by analogy to compound **3x** and **4k**.

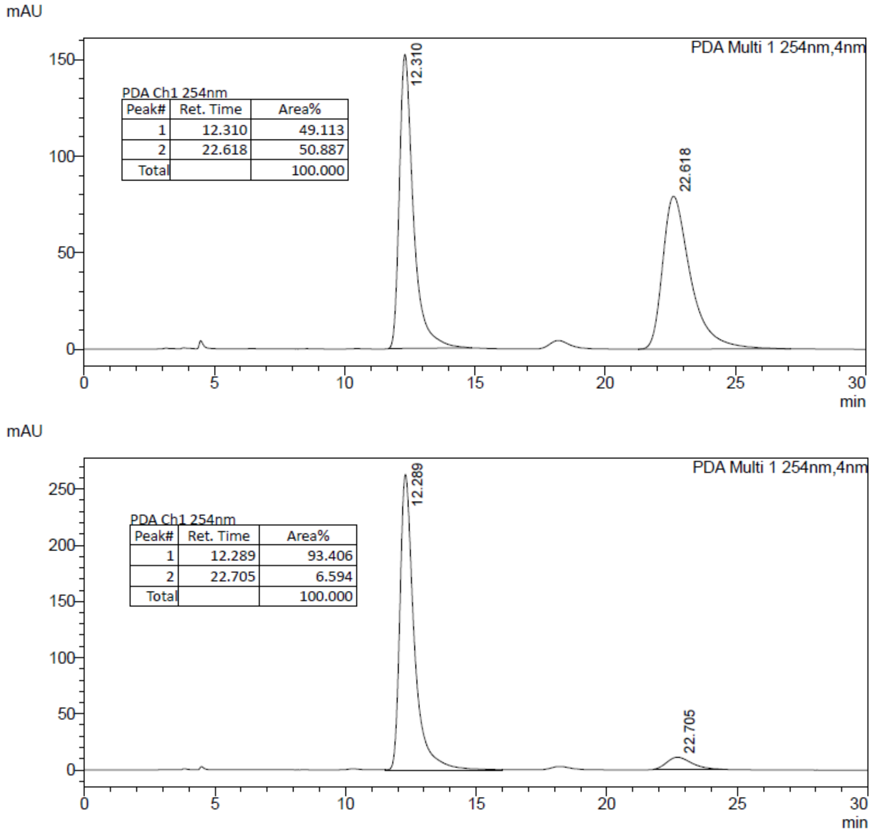

Supplementary Fig. 55. HPLC traces of *rac*-**3v** (top) and enantioenriched-**3v** (bottom).

400 MHz, ^{1}H NMR in $CDCl_3$

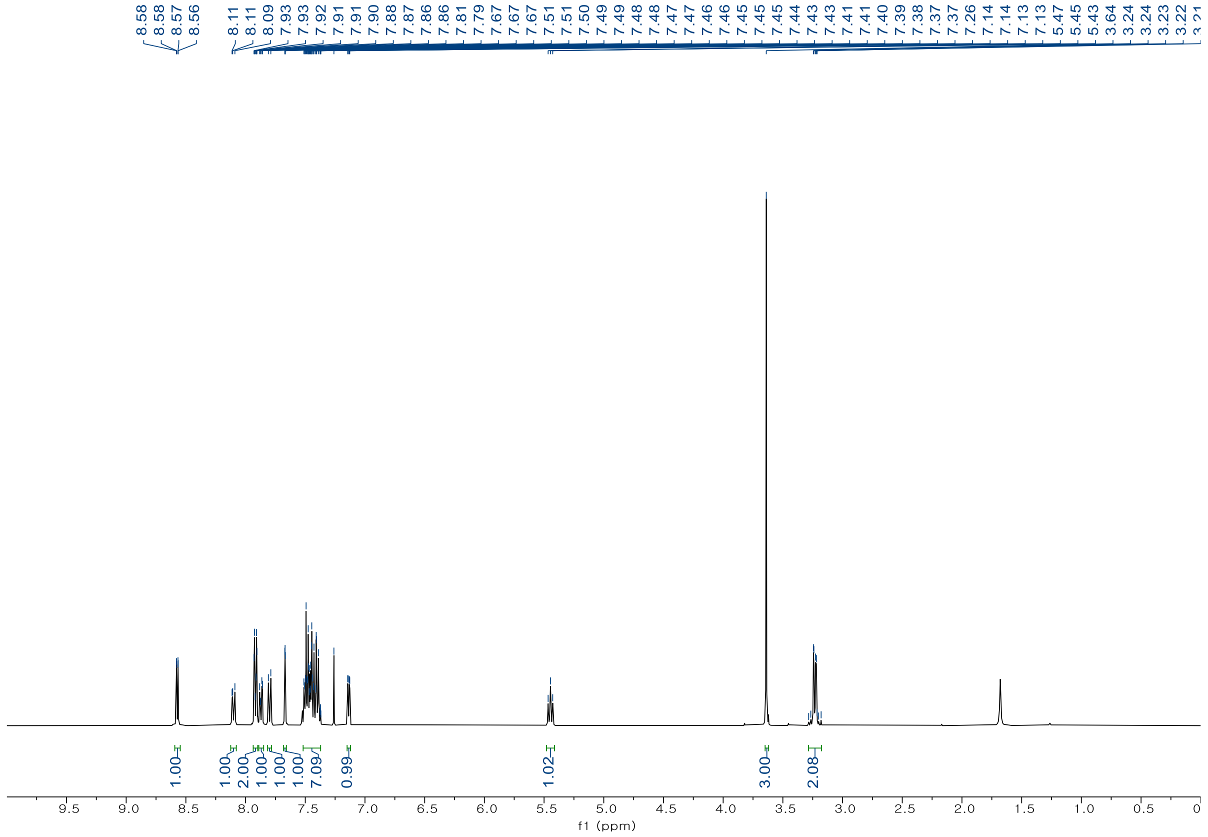

100 MHz, ^{13}C NMR in $CDCl_3$

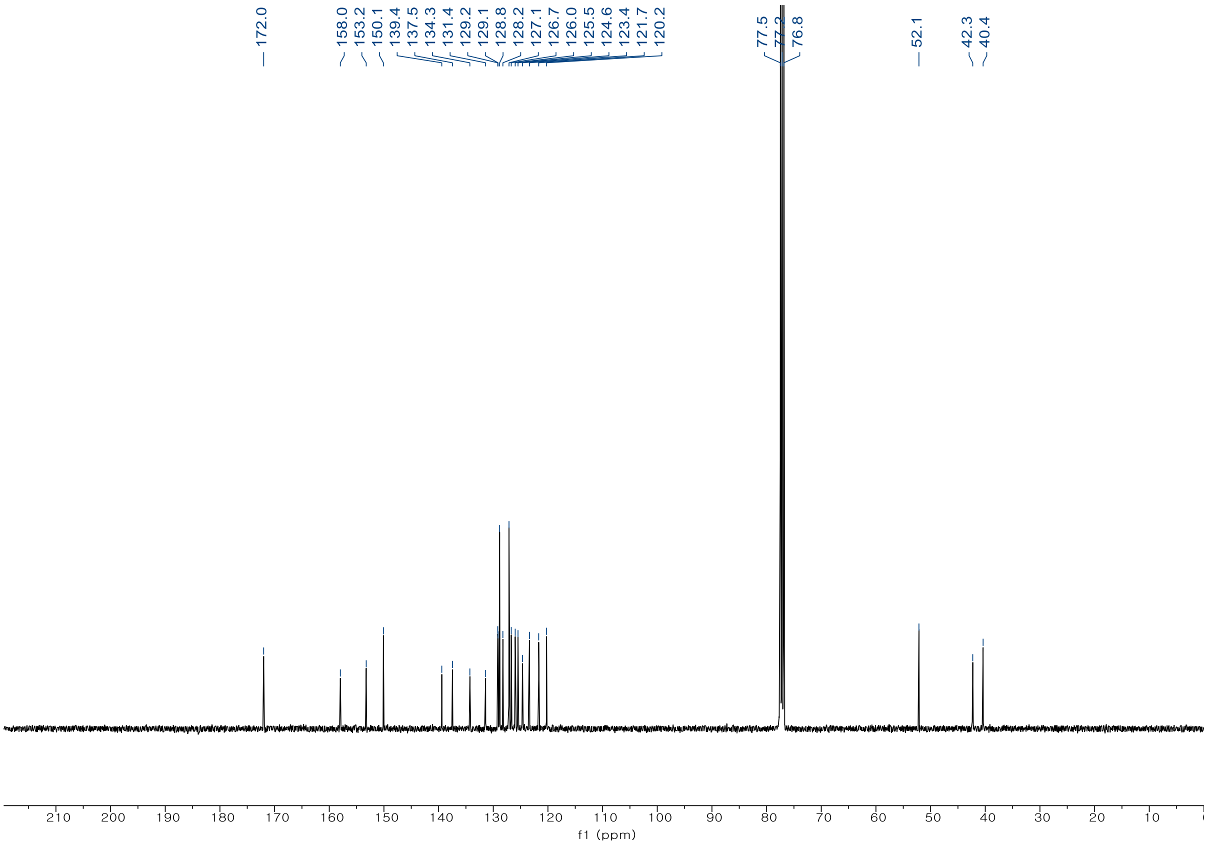

Supplementary Fig. 56. ^{1}H and ^{13}C NMR of **3v**.

Methyl (*R*)-3-(naphthalen-2-yl)-3-(2-phenylpyridin-4-yl)propanoate (3w)

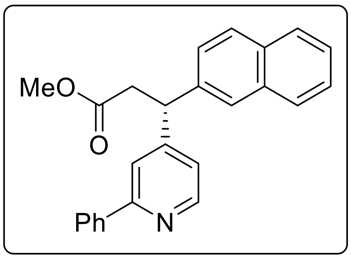

Prepared according to **GP**. Desired product **3w** was obtained as white solid (12.4 mg, 67% isolated yield). **^{1}H NMR** (500 MHz, $CDCl_3$) δ 8.60 (d, *J* = 5.1 Hz, 1H), 7.93 (d, *J* = 7.0 Hz, 2H), 7.83 – 7.76 (m, 3H), 7.73 (s, 1H), 7.65 (s, 1H), 7.50 – 7.43 (m, 4H), 7.42 – 7.37 (m, 1H), 7.33 (dd, *J* = 8.6, 1.9 Hz, 1H), 7.15 (dd, *J* = 5.2, 1.7 Hz, 1H), 4.78 (t, *J* = 7.9 Hz, 1H), 3.62 (s, 3H), 3.22 (d, *J* = 7.8 Hz, 2H). **^{13}C NMR** (125 MHz, $CDCl_3$) δ 171.9, 158.1, 153.0, 150.1, 139.4, 139.3, 133.6, 132.6, 129.1, 128.9, 128.0, 127.8, 127.2, 126.5, 126.2, 126.2, 126.2, 121.6, 120.2, 52.1, 46.8, 39.9. **HRMS** (ESI^+) m/z calcd. For $C_{25}H_{22}NO_2^+$ $[M+H]^+$: 368.1651, found 368.1652. **Specific Rotation** $[\alpha]_D^{25}$ +9.08 (*c* 0.37, $CHCl_3$). **HPLC Analysis**. CHIRALCEL OD-H, 25 ºC; *n*-hexane:*i*-PrOH = 75:25, 1.0 mL/min, 254 nm, t_{R1} (major) = 18.07 min, t_{R2} (minor) = 23.17 min, 92:8 er.

The absolute stereochemistry was assigned by analogy to compound **3x** and **4k**.

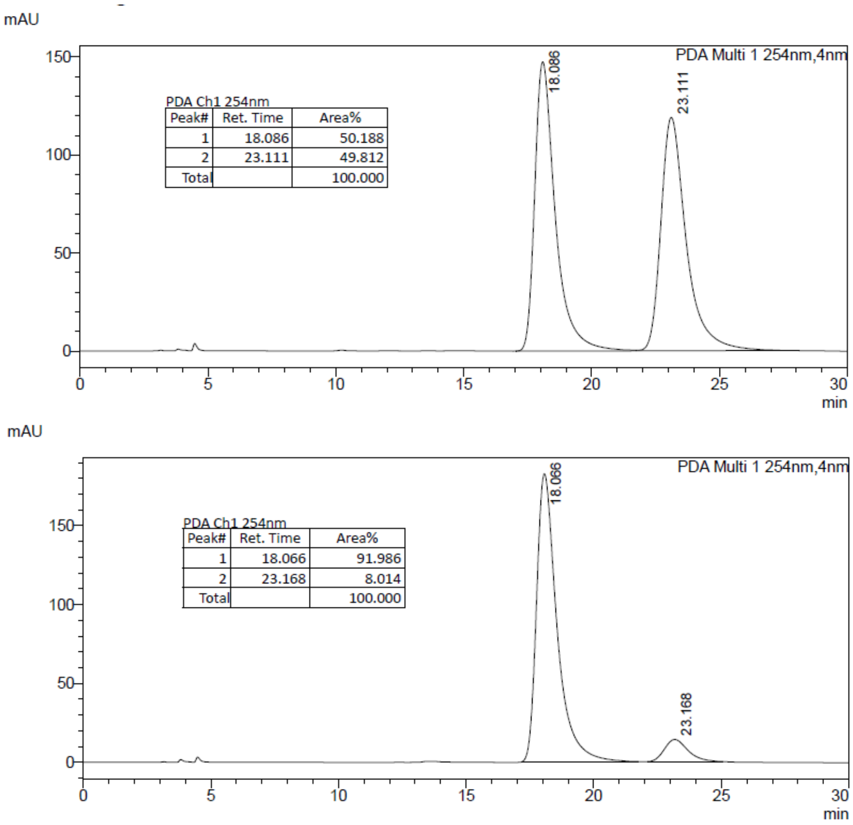

Supplementary Fig. 57. HPLC traces of *rac*-**3w** (top) and enantioenriched-**3w** (bottom).

500 MHz, ^{1}H NMR in $CDCl_3$

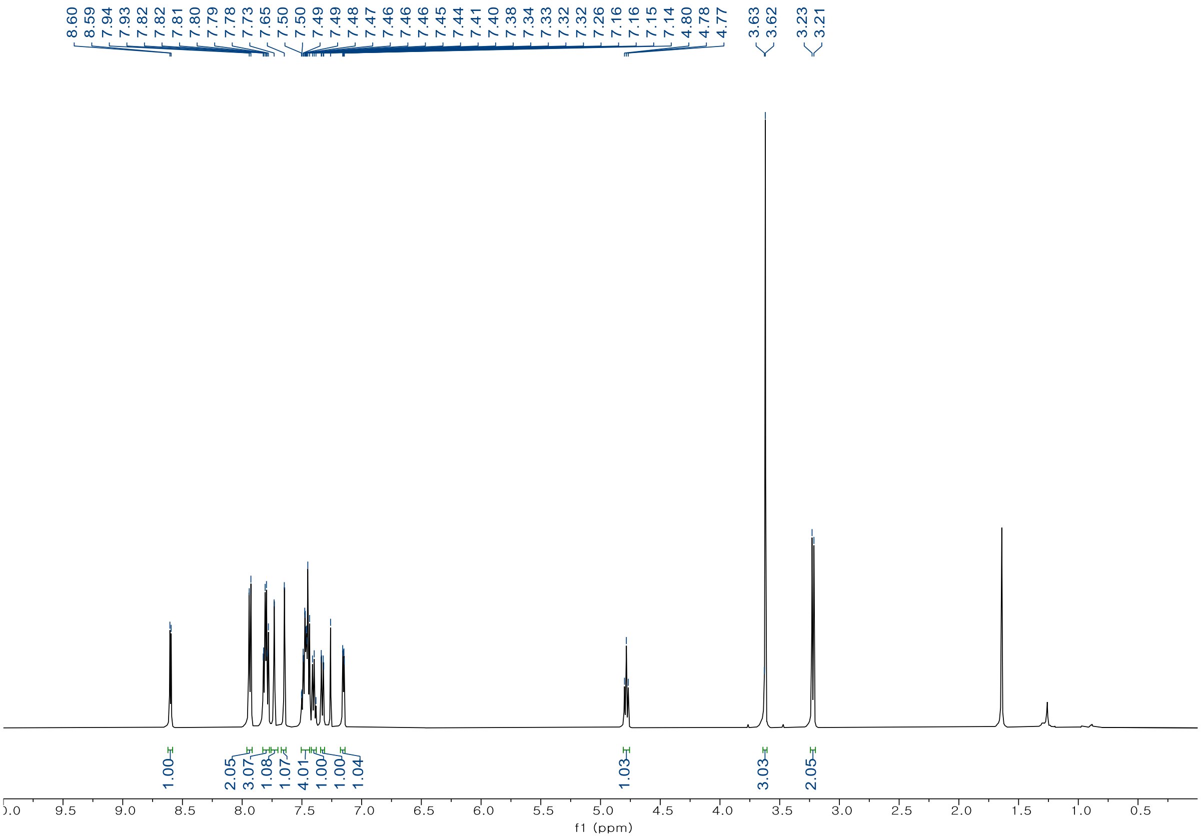

125 MHz, ^{13}C NMR in $CDCl_3$

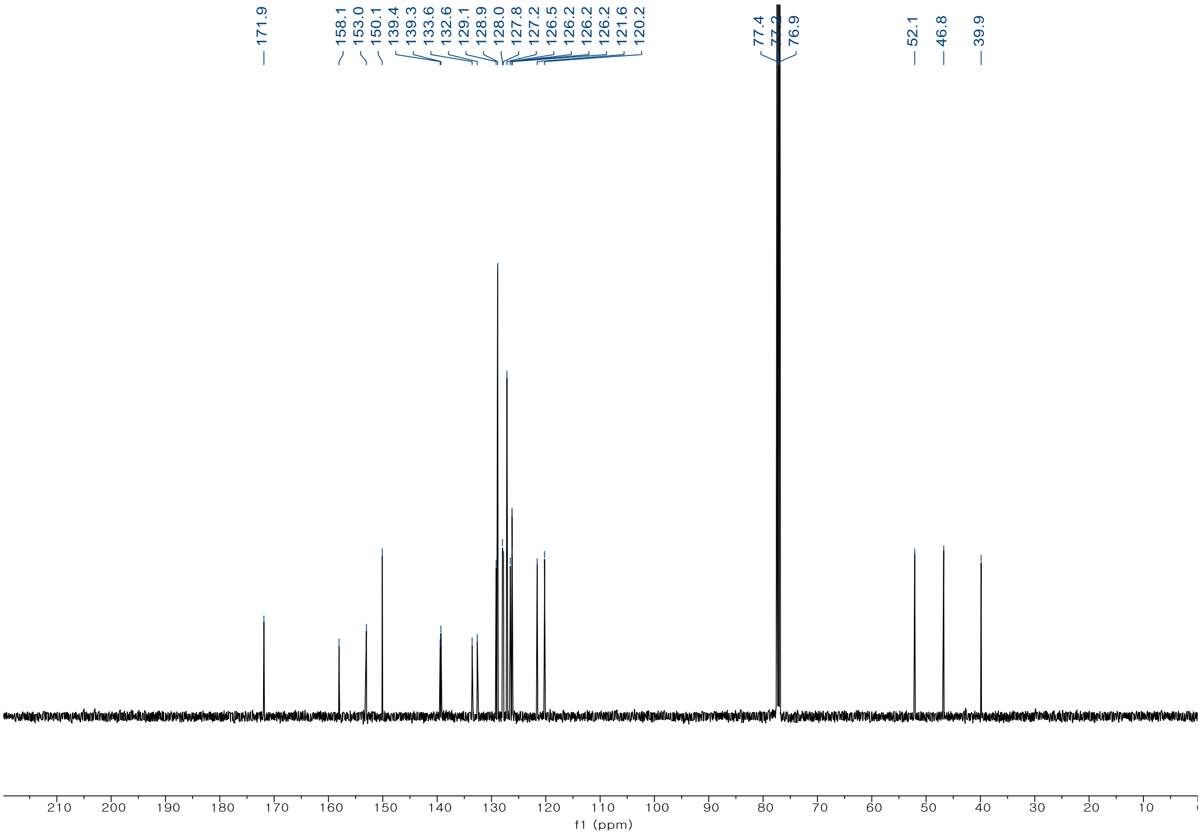

Supplementary Fig. 58. ^{1}H and ^{13}C NMR of **3w**.

Methyl (*R*)-3-(2-(4-bromophenyl)pyridin-4-yl)-3-(naphthalen-2-yl)propanoate (3x)

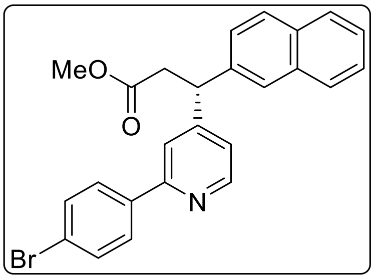

Prepared according to **GP**. Desired product **3x** was obtained as white solid (12.0 mg, 54% isolated yield). **^{1}H NMR** (400 MHz, $CDCl_3$) δ 8.59 (d, *J* = 5.1 Hz, 1H), 7.86 – 7.76 (m, 5H), 7.74 – 7.69 (m, 1H), 7.64 – 7.59 (m, 1H), 7.59 – 7.54 (m, 2H), 7.52 – 7.43 (m, 2H), 7.32 (dd, *J* = 8.5, 1.9 Hz, 1H), 7.17 (dd, *J* = 5.1, 1.7 Hz, 1H), 4.78 (t, *J* = 7.8 Hz, 1H), 3.62 (s, 3H), 3.21 (d, *J* = 7.9 Hz, 2H). **^{13}C NMR** (100 MHz, $CDCl_3$) δ 171.8, 156.8, 153.2, 150.2, 139.2, 138.3, 133.5, 132.6, 132.0, 128.9, 128.7, 128.0, 127.8, 126.6, 126.2, 126.2, 126.1, 123.6, 121.8, 120.0, 52.1, 46.7, 39.8. **HRMS** (ESI$^+$) m/z calcd. For $C_{25}H_{21}BrNO_2^+$ [M+H]$^+$: 446.0756, found 446.0755. **Specific Rotation** $[\alpha]_D^{26}$ +24.0 (*c* 1.0, $CHCl_3$). **HPLC Analysis**. CHIRALPAK IB, 25 ºC; *n*-hexane:*i*-PrOH = 90:10, 1.0 mL/min, 254 nm, t_{R1} (major) = 21.82 min, t_{R2} (minor) = 24.95 min, 94:6 er.

The absolute stereochemistry was assigned by X-ray crystallography analysis.

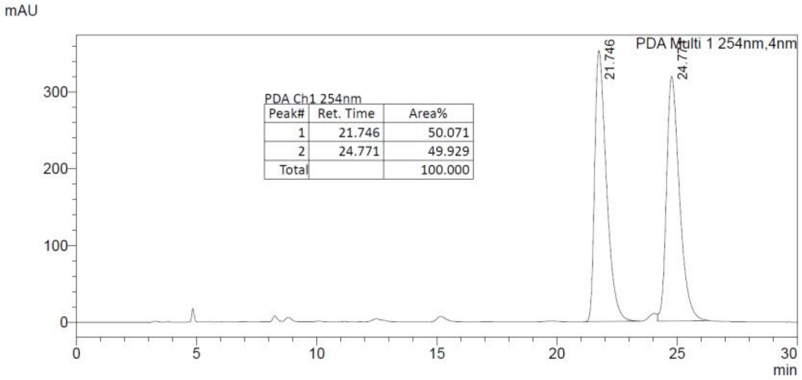

Peak#	Ret. Time	Area%
1	21.746	50.071
2	24.771	49.929
Total		100.000

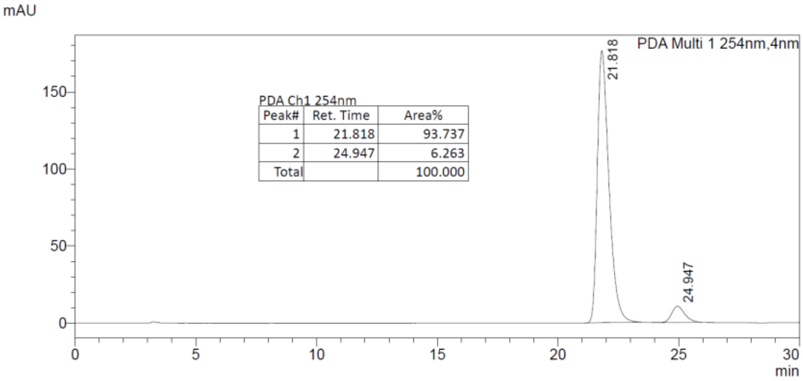

Peak#	Ret. Time	Area%
1	21.818	93.737
2	24.947	6.263
Total		100.000

Supplementary Fig. 59. HPLC traces of *rac*-**3x** (top) and enantioenriched-**3x** (bottom).

400 MHz, ^{1}H NMR in $CDCl_3$.

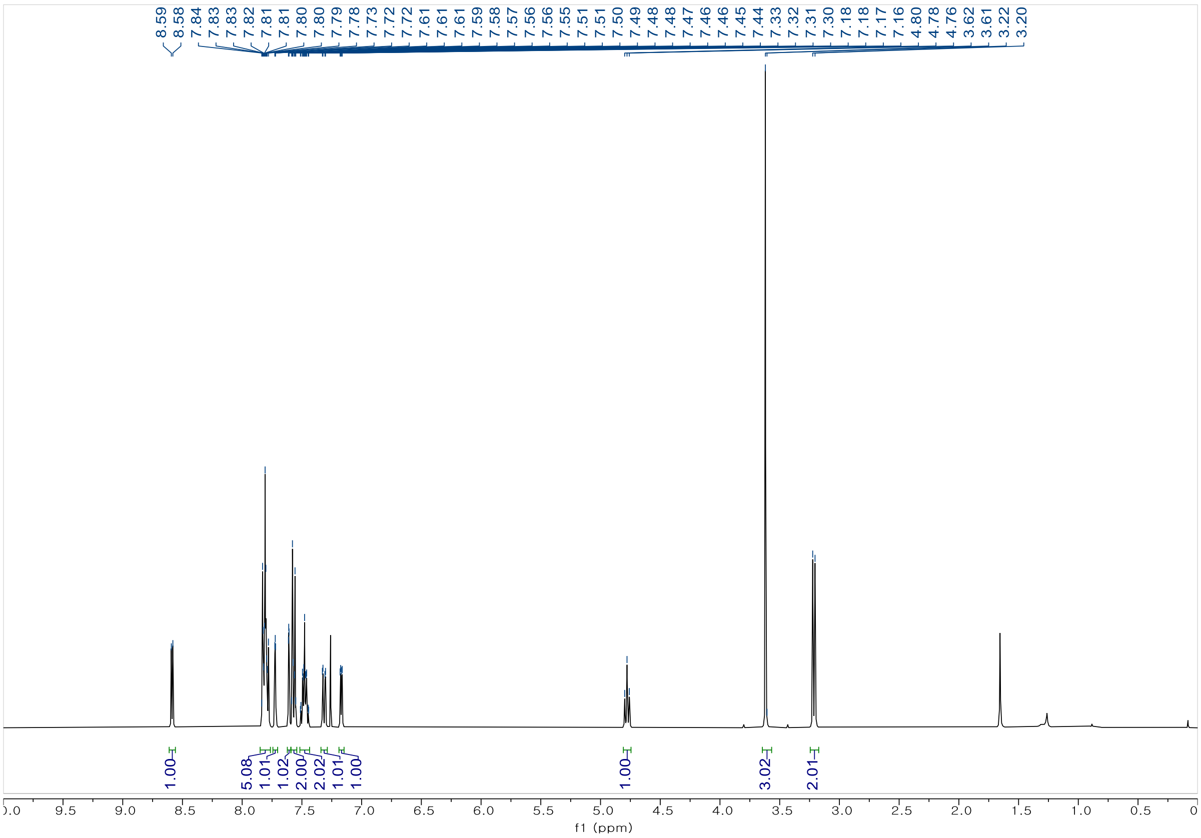

100 MHz, ^{13}C NMR in $CDCl_3$.

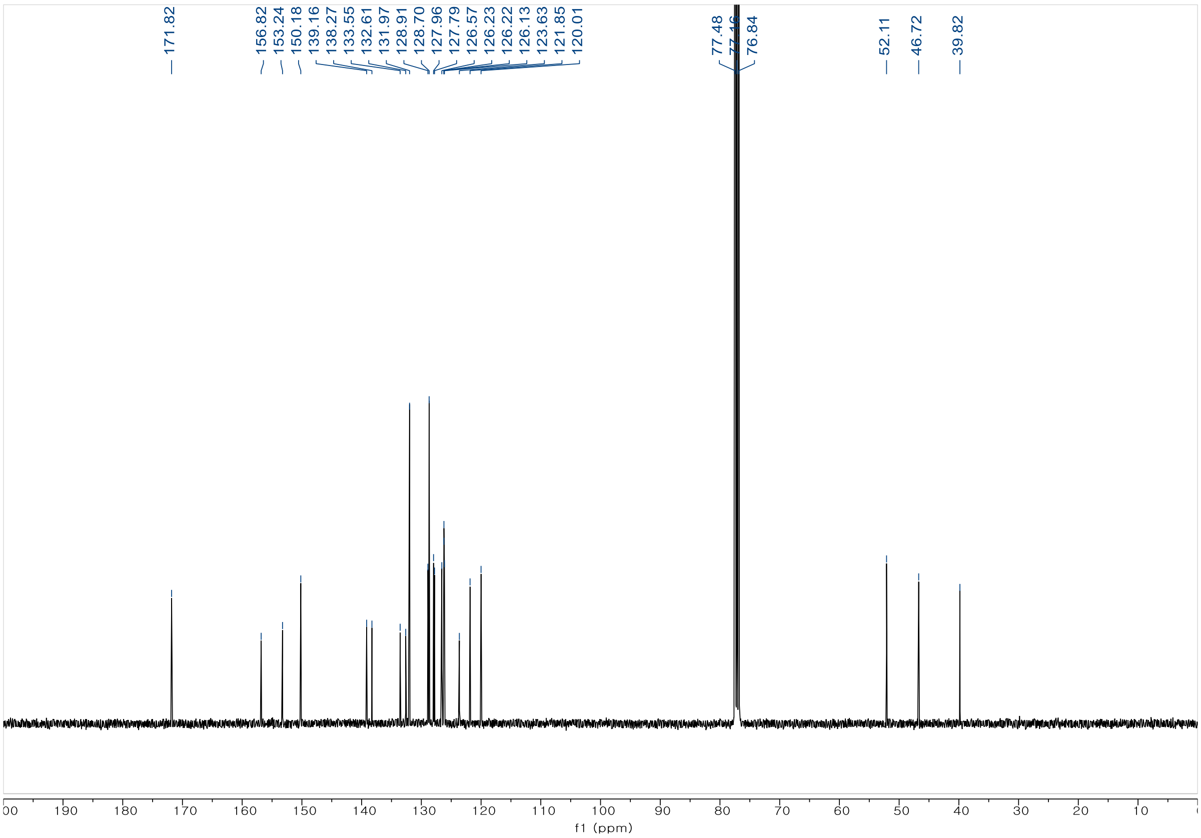

Supplementary Fig. 60. ^{1}H and ^{13}C NMR of **3x**.

Methyl (*R*)-3-phenyl-3-(pyridin-4-yl)propanoate (4a)

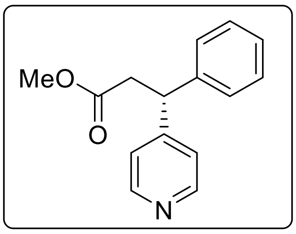

Prepared according to **GP**. Desired product **4a** was obtained as colorless gum (7.9 mg, 66% isolated yield). **^{1}H NMR** (500 MHz, $CDCl_3$) δ 8.51 (d, *J* = 5.3 Hz, 2H), 7.34 – 7.29 (m, 2H), 7.25 – 7.19 (m, 3H), 7.17 – 7.14 (m, 2H), 4.53 (t, *J* = 7.9 Hz, 1H), 3.60 (s, 3H), 3.14 – 2.98 (m, 2H). **^{13}C NMR** (100 MHz, $CDCl_3$) δ 171.8, 152.4, 150.2, 141.8, 129.0, 127.8, 127.3, 123.1, 52.0, 46.4, 39.8. **HRMS** (EI^+) m/z calcd. for $C_{15}H_{15}NO_2^+$ $[M]^+$: 241.1103, found: 241.1101. **Specific Rotation** $[\alpha]_D^{25}$ -5.9 (*c* 0.45, $CHCl_3$). **HPLC Analysis**. CHIRALCEL OD-H, 25 ºC; *n*-hexane:*i*-PrOH = 90:10, 0.7 mL/min, 254 nm, t_{R1} (major) = 24.06 min, t_{R2} (minor) = 26.27 min, 94:6 er.

The absolute stereochemistry was assigned by analogy to compound **3x** and **4k**.

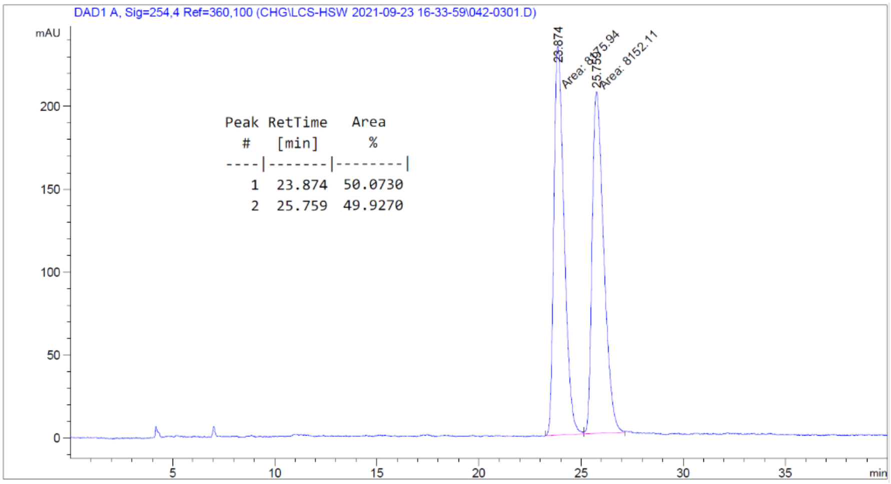

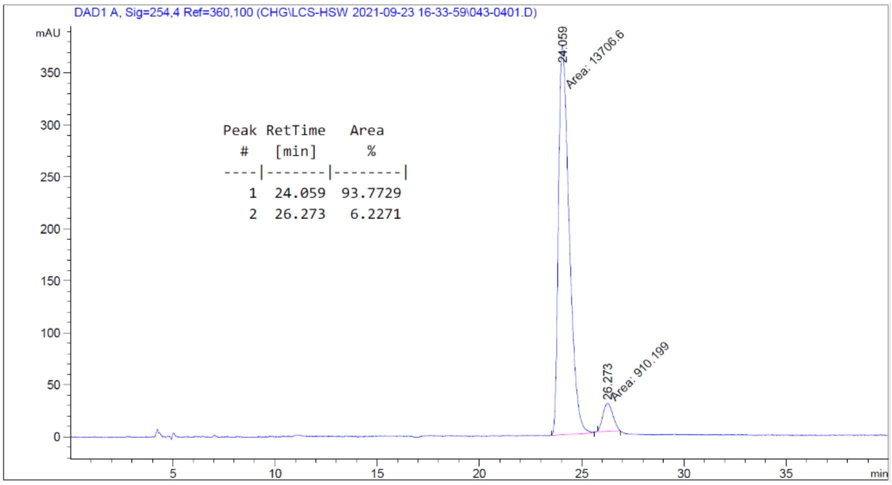

Supplementary Fig. 61. HPLC traces of *rac*-**4a** (top) and enantioenriched-**4a** (bottom).

500 MHz, ^{1}H NMR in $CDCl_3$

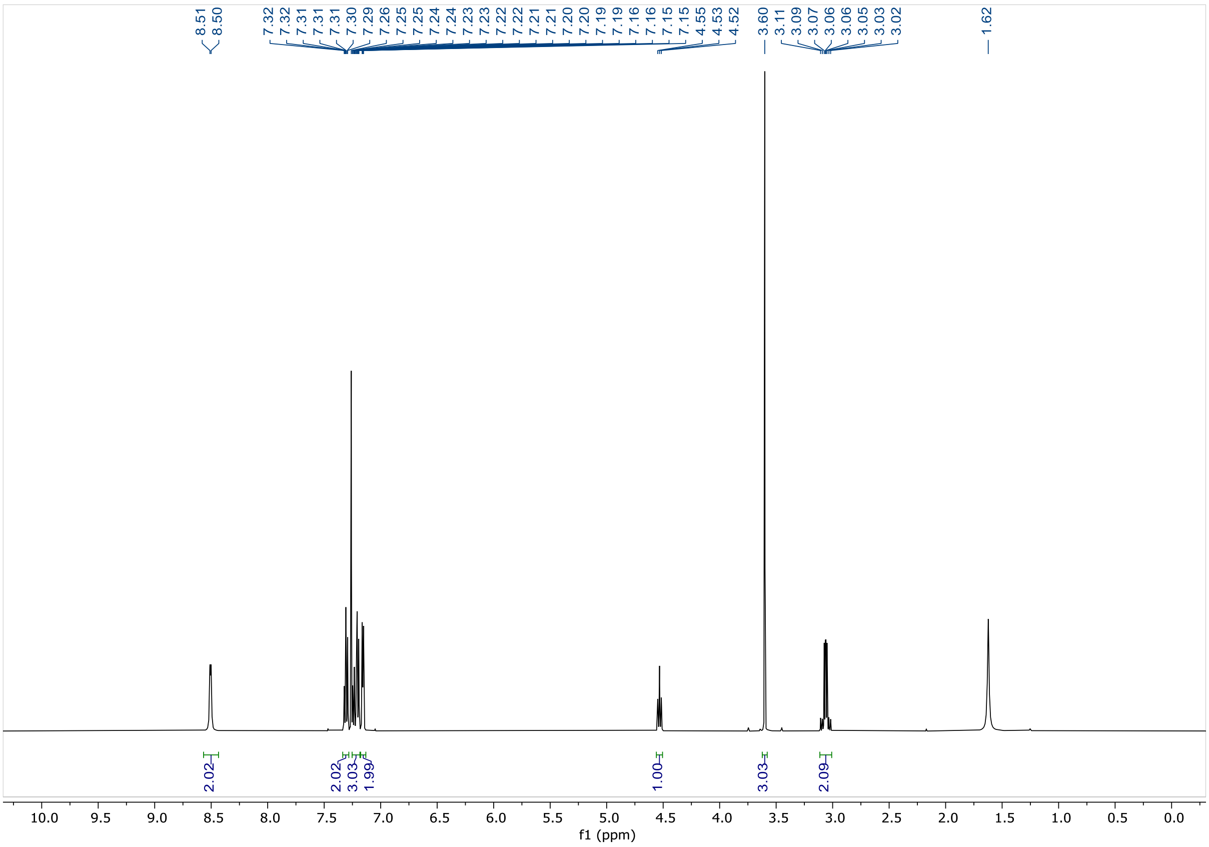

100 MHz, ^{13}C NMR in $CDCl_3$

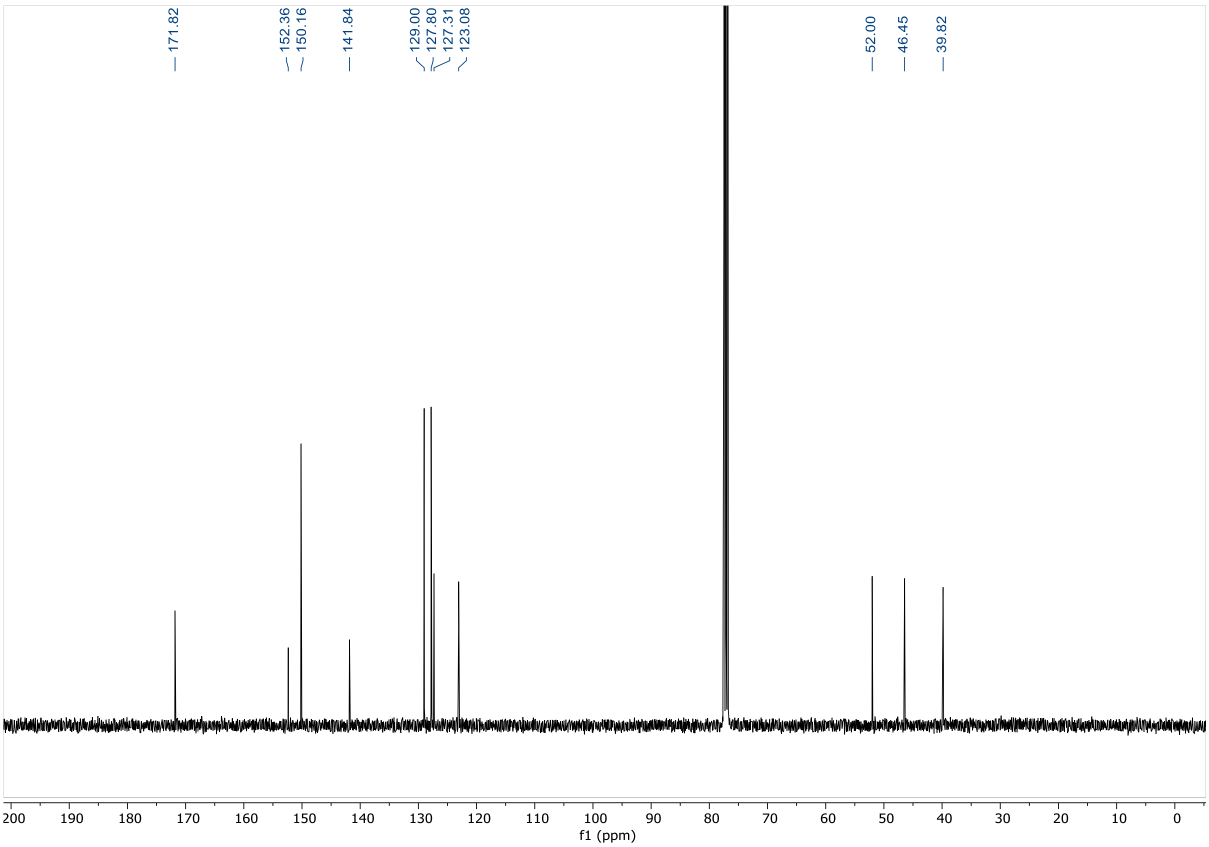

Supplementary Fig. 62. ^{1}H and ^{13}C NMR of **4a**.

Methyl (*R*)-3-(2-methylpyridin-4-yl)-3-phenylpropanoate (4b)

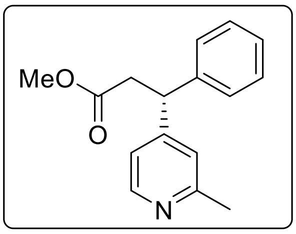

Prepared according to **GP**. Desired product **4b** was obtained as colorless gum (7.4 mg, 58% isolated yield). **^{1}H NMR** (500 MHz, $CDCl_3$) δ 8.38 (d, *J* = 5.2 Hz, 1H), 7.30 (dd, *J* = 8.1, 6.8 Hz, 2H), 7.25 – 7.17 (m, 3H), 7.01 (d, *J* = 1.8 Hz, 1H), 6.97 (dd, *J* = 5.1, 1.7 Hz, 1H), 4.48 (t, *J* = 7.9 Hz, 1H), 3.60 (s, 3H), 3.19 – 2.94 (m, 2H), 2.50 (s, 3H). **^{13}C NMR** (100 MHz, $CDCl_3$) δ 171.9, 158.8, 152.6, 149.5, 142.0, 129.0, 127.8, 127.2, 122.7, 120.1, 52.0, 46.4, 39.8, 24.6. **HRMS** (EI^+) m/z calcd. for $C_{16}H_{17}NO_2^+$ $[M]^+$: 255.1259, found: 255.1260. **Specific Rotation** $[\alpha]_D^{25}$ -9.9 (*c* 0.48, $CHCl_3$). **HPLC Analysis**. CHIRALCEL OD-H, 25 °C; *n*-hexane:*i*-PrOH = 80:20, 0.7 mL/min, 254 nm, t_{R1} (major) =10.72 min, t_{R2} (minor) = 12.36 min, 88:12 er.

The absolute stereochemistry was assigned by analogy to compound **3x** and **4k**.

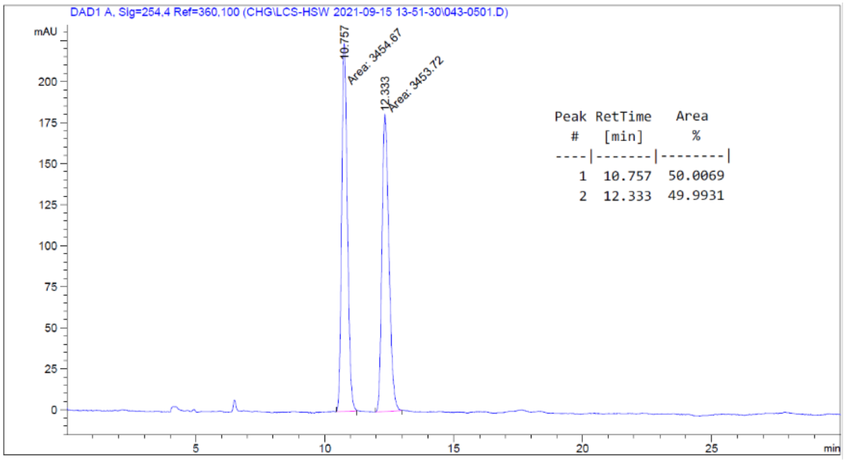

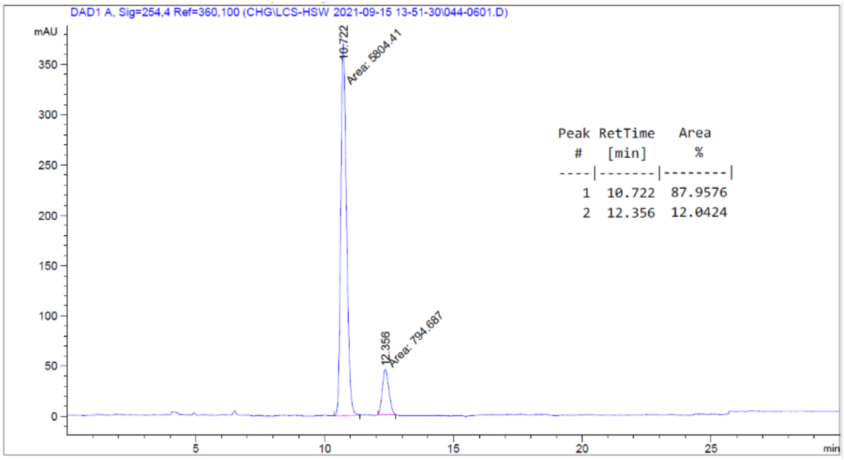

Supplementary Fig. 63. HPLC traces of *rac*-**4b** (top) and enantioenriched-**4b** (bottom).

500 MHz, ^{1}H NMR in $CDCl_3$

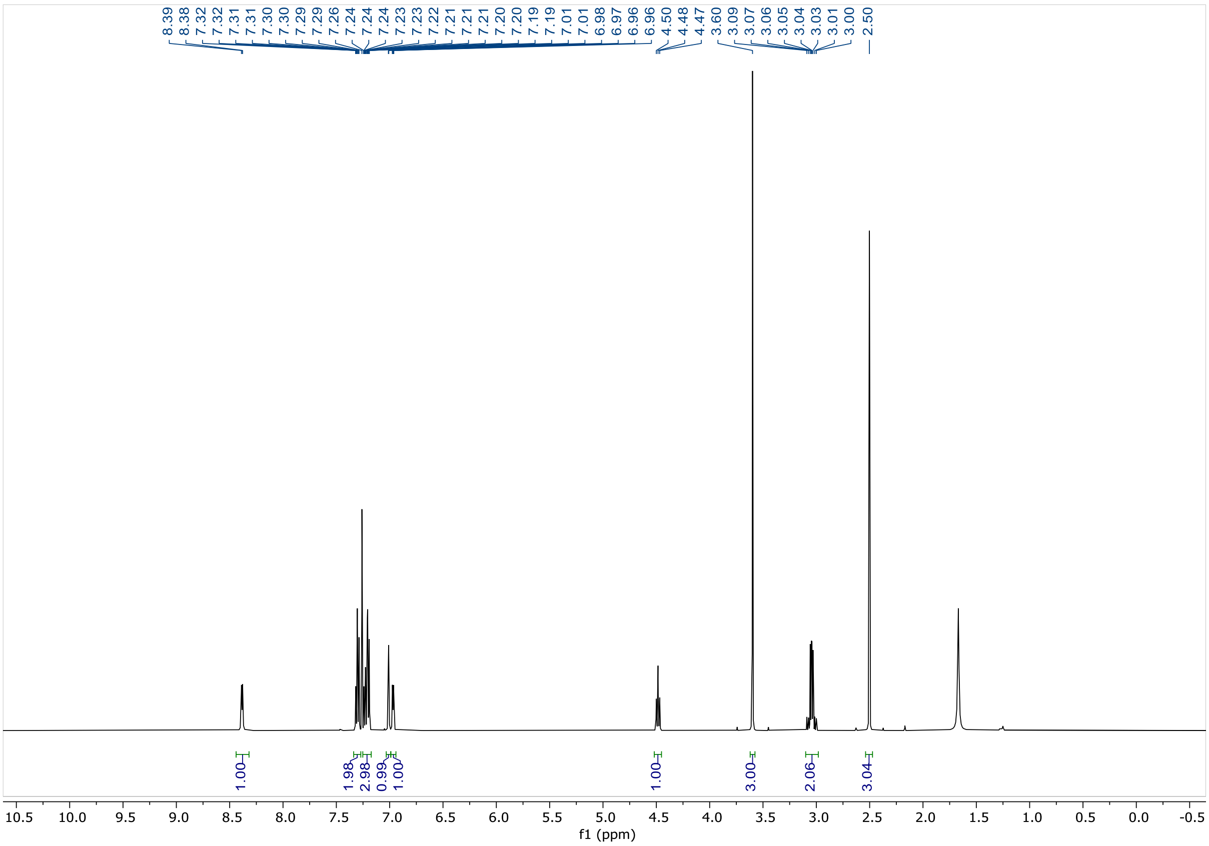

100 MHz, ^{13}C NMR in $CDCl_3$

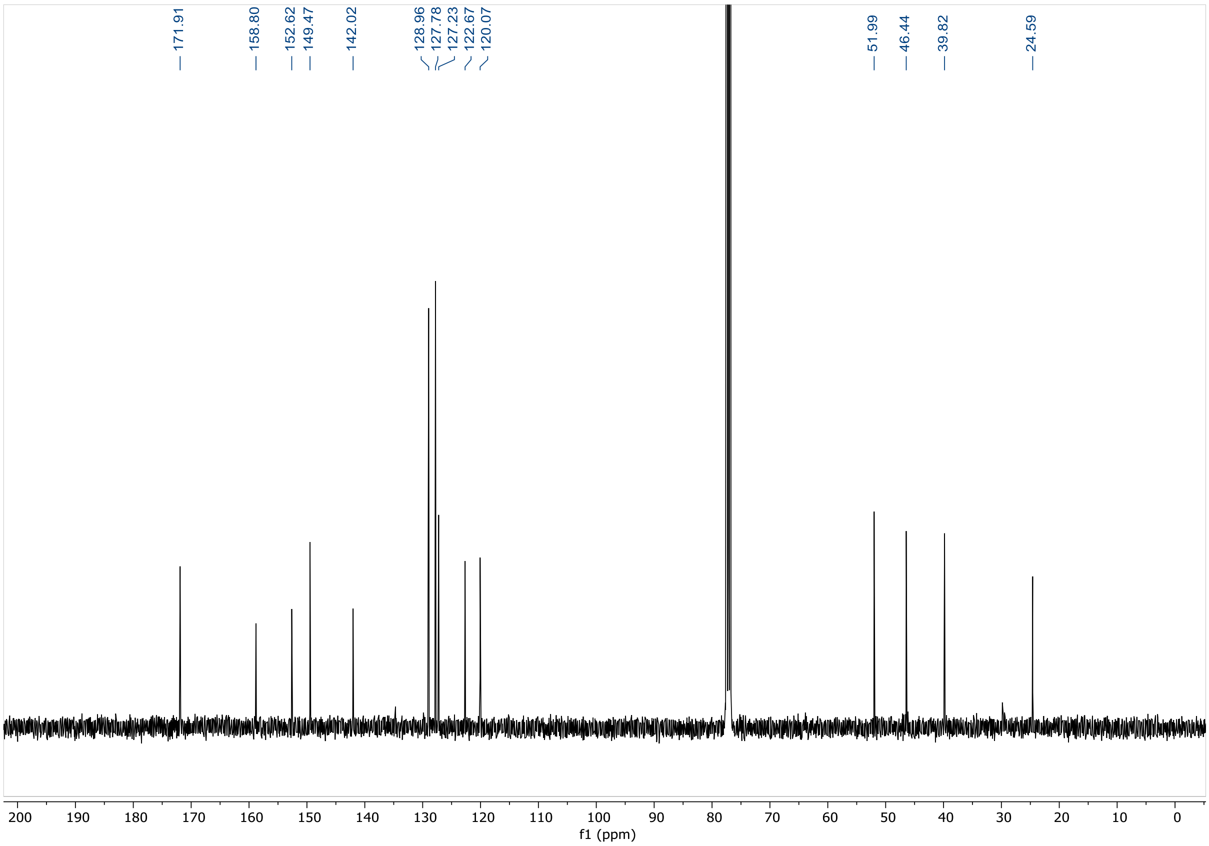

Supplementary Fig. 64. ^{1}H and ^{13}C NMR of **4b**.

Methyl (*R*)-3-(2,6-dimethylpyridin-4-yl)-3-phenylpropanoate (4c)

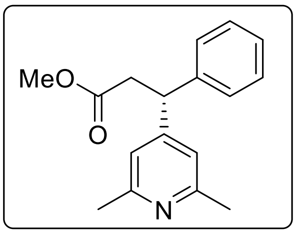

Prepared according to **GP**. Desired product **4c** was obtained as colorless gum (6.5 mg, 48% isolated yield). **^{1}H NMR** (500 MHz, $CDCl_3$) δ 7.30 (dd, *J* = 8.2, 6.9 Hz, 2H), 7.25 – 7.18 (m, 3H), 6.83 (s, 2H), 4.44 (t, *J* = 7.9 Hz, 1H), 3.60 (s, 3H), 3.11 – 2.97 (m, 2H), 2.48 (s, 6H). **^{13}C NMR** (100 MHz, $CDCl_3$) δ 172.0, 158.1, 152.9, 142.2, 128.9, 127.8, 127.2, 119.6, 52.0, 46.4, 39.8, 24.6. **HRMS** (EI^+) m/z calcd. for $C_{17}H_{19}NO_2^+$ $[M]^+$: 269.1416, found: 269.1417. **Specific Rotation** $[\alpha]_D^{25}$ -10.1 (*c* 0.31, $CHCl_3$). **HPLC Analysis**. CHIRALCEL OD-H, 25 ºC; *n*-hexane:*i*-PrOH = 75:25, 1.0 mL/min, 254 nm, t_{R1} (major) =5.38 min, t_{R2} (minor) = 6.88 min, 80:20 er.

The absolute stereochemistry was assigned by analogy to compound **3x** and **4k**.

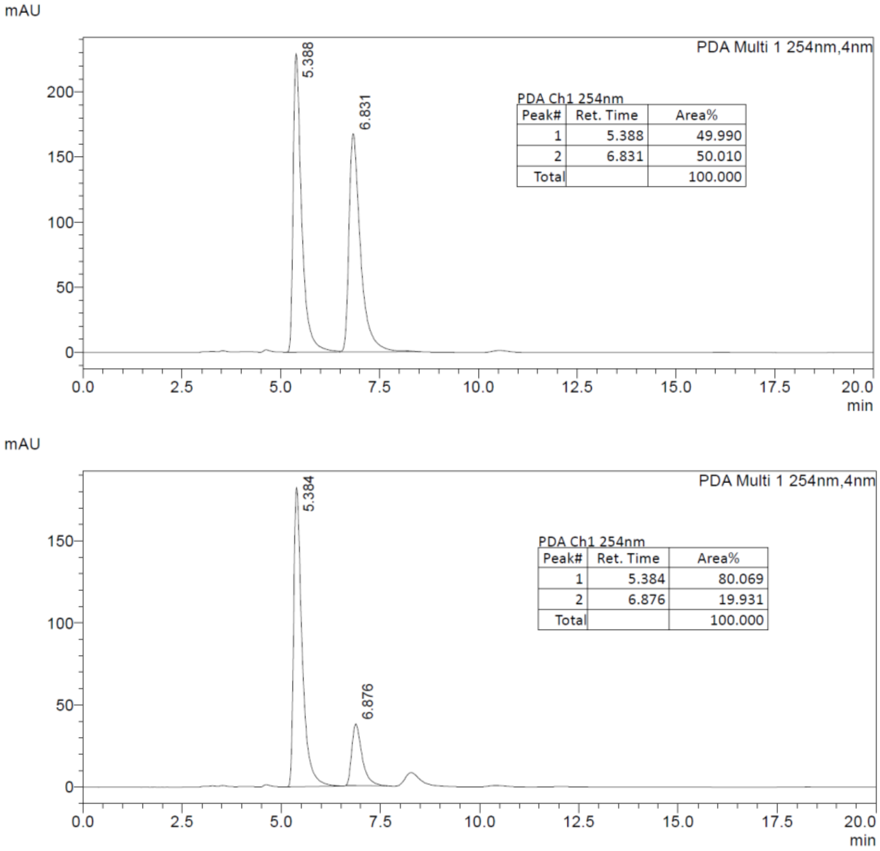

Supplementary Fig. 65. HPLC traces of *rac*-**4c** (top) and enantioenriched-**4c** (bottom).

500 MHz, ^{1}H NMR in $CDCl_3$

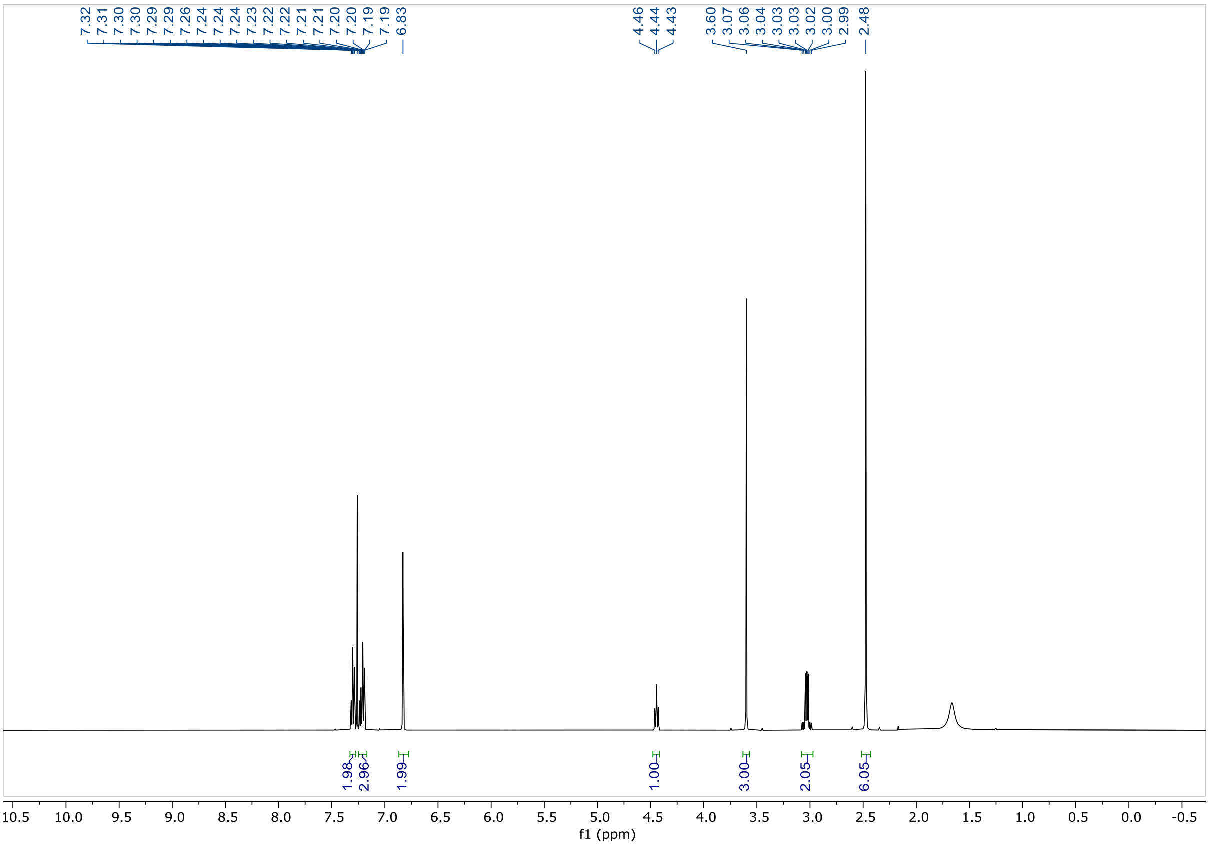

100 MHz, ^{13}C NMR in $CDCl_3$

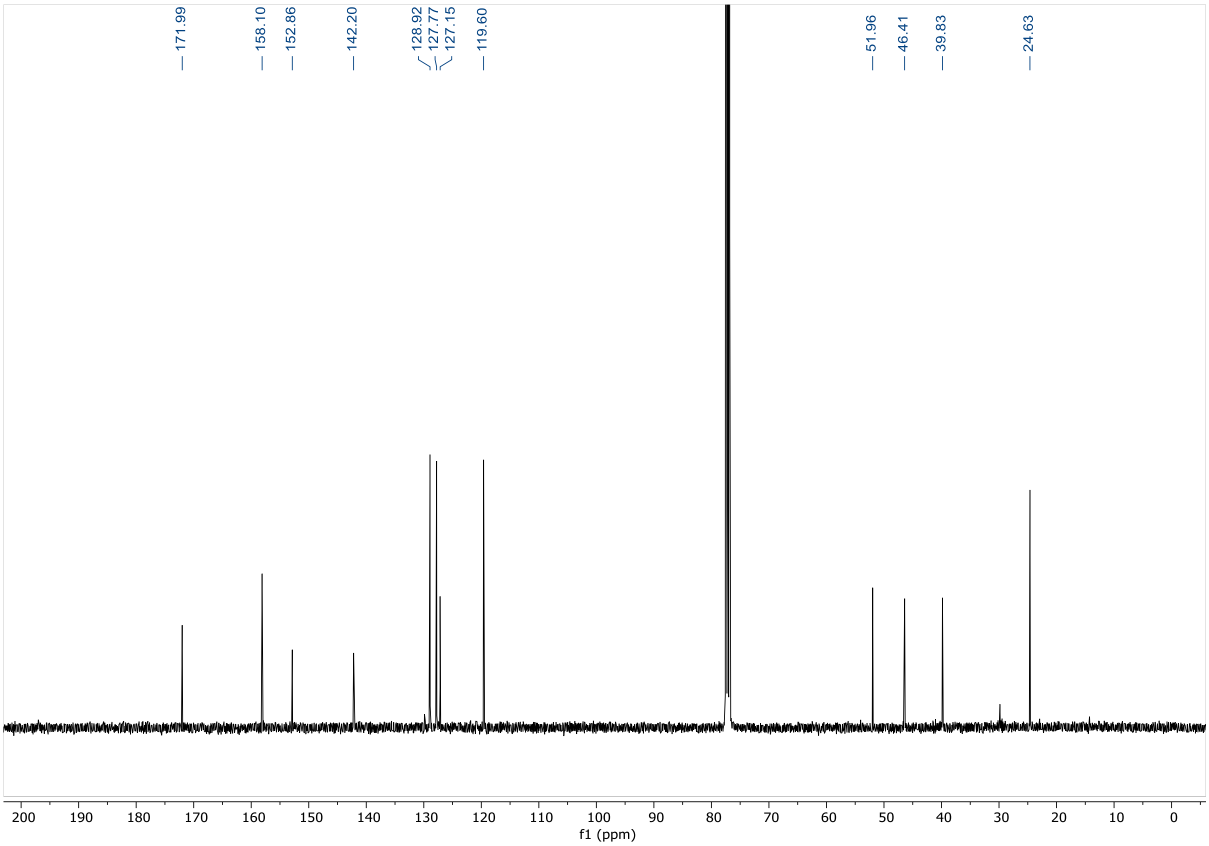

Supplementary Fig. 66. ^{1}H and ^{13}C NMR of **4c**.

Methyl (*R*)-3-phenyl-3-(2-(*p*-tolyl)pyridin-4-yl)propanoate (4d)

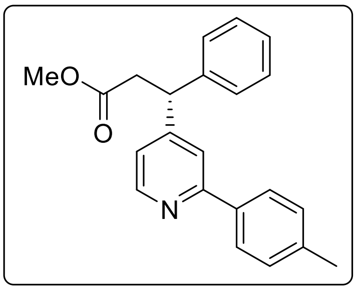

Prepared according to **GP**. Desired product **4d** was obtained as white solid (11.0 mg, 66% isolated yield). **^{1}H NMR** (500 MHz, $CDCl_3$) δ 8.56 (d, *J* = 5.1 Hz, 1H), 7.83 (d, *J* = 8.2 Hz, 2H), 7.59 – 7.52 (m, 1H), 7.31 (dd, *J* = 8.3, 6.8 Hz, 2H), 7.27 – 7.26 (m, 1H), 7.26 – 7.21 (m, 4H), 7.07 (dd, *J* = 5.2, 1.7 Hz, 1H), 4.59 (t, *J* = 7.9 Hz, 1H), 3.61 (s, 3H), 3.20 – 3.01 (m, 2H), 2.40 (s, 3H). **^{13}C NMR** (100 MHz, $CDCl_3$) δ 171.9, 158.0, 153.0, 150.0, 141.9, 139.1, 136.7, 129.6, 129.0, 127.8, 127.3, 127.0, 121.2, 119.8, 52.0, 46.7, 39.9, 21.4. **HRMS** (EI^+) m/z calcd. for $C_{22}H_{21}NO_2^+$ $[M]^+$: 331.1572, found: 331.1573. **Specific Rotation** $[\alpha]_D^{24}$ -0.5 (*c* 0.66, $CHCl_3$). **HPLC Analysis**. CHIRALCEL OD-H, 25 ºC; *n*-hexane:*i*-PrOH = 75:25, 1.0 mL/min, 254 nm, t_{R1} (major) = 7.42 min, t_{R2} (minor) = 13.02 min, 94:6 er.

The absolute stereochemistry was assigned by analogy to compound **3x** and **4k**.

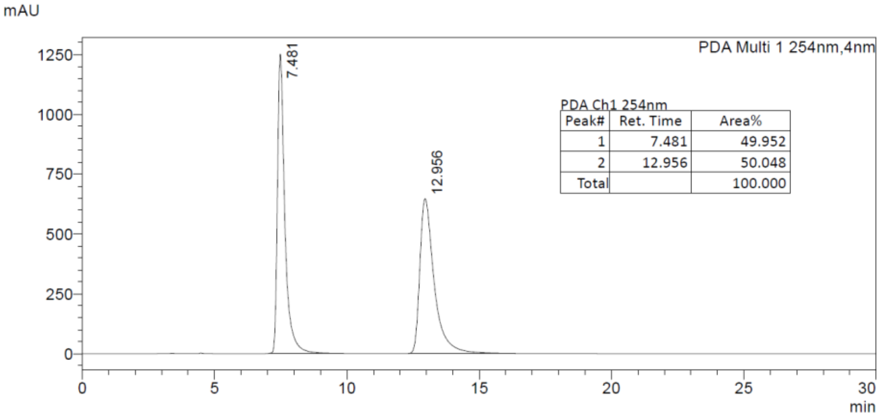

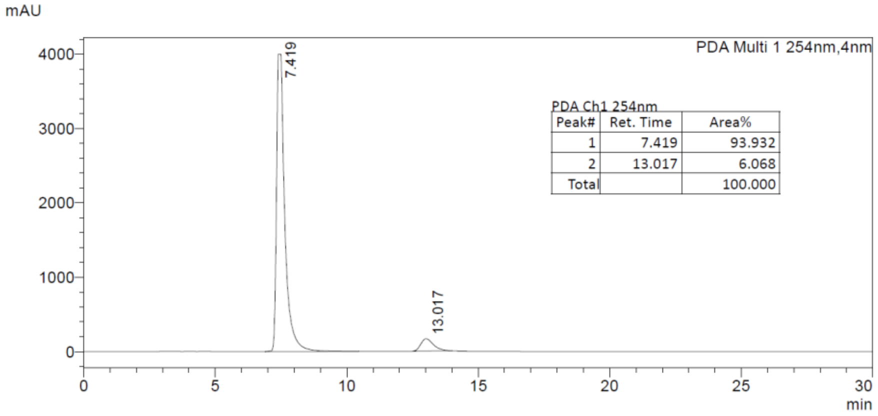

Supplementary Fig. 67. HPLC traces of *rac*-**4d** (top) and enantioenriched-**4d** (bottom).

500 MHz, ^{1}H NMR in $CDCl_3$

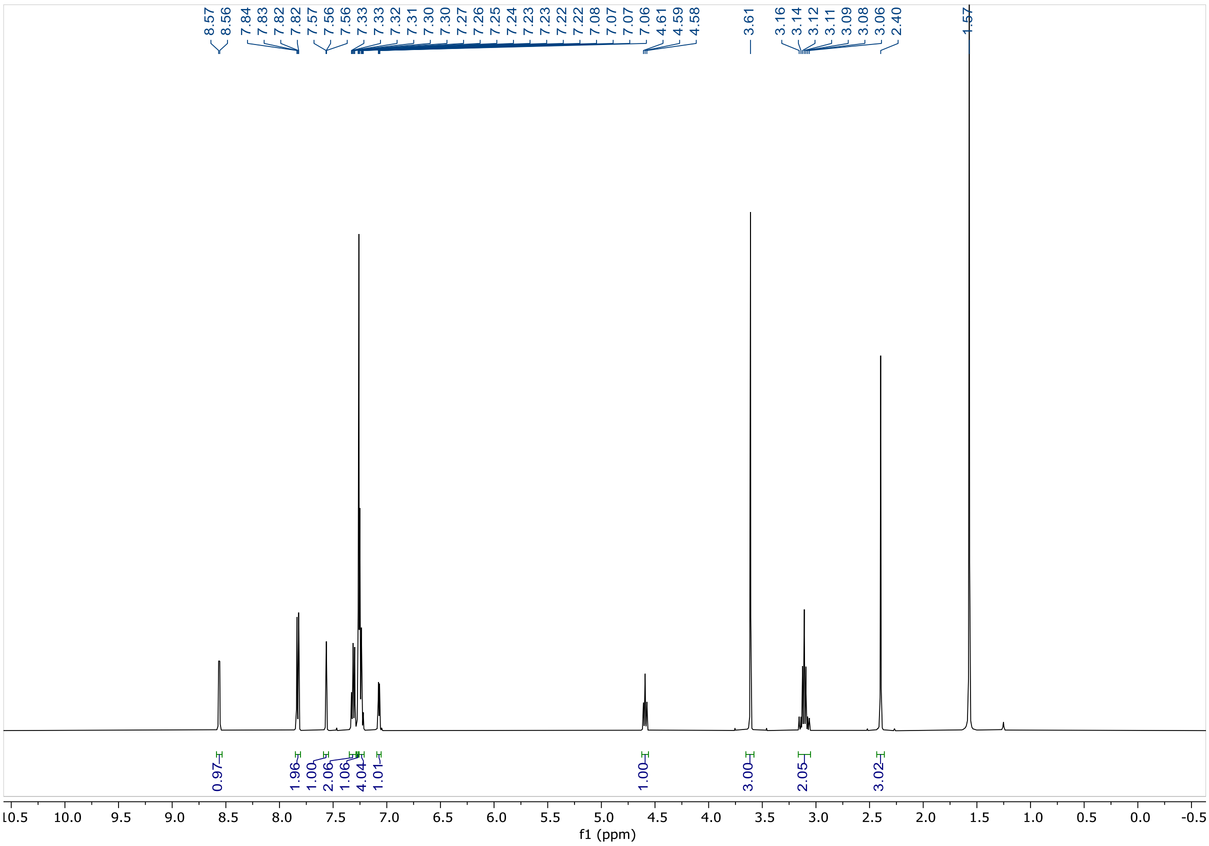

100 MHz, ^{13}C NMR in $CDCl_3$

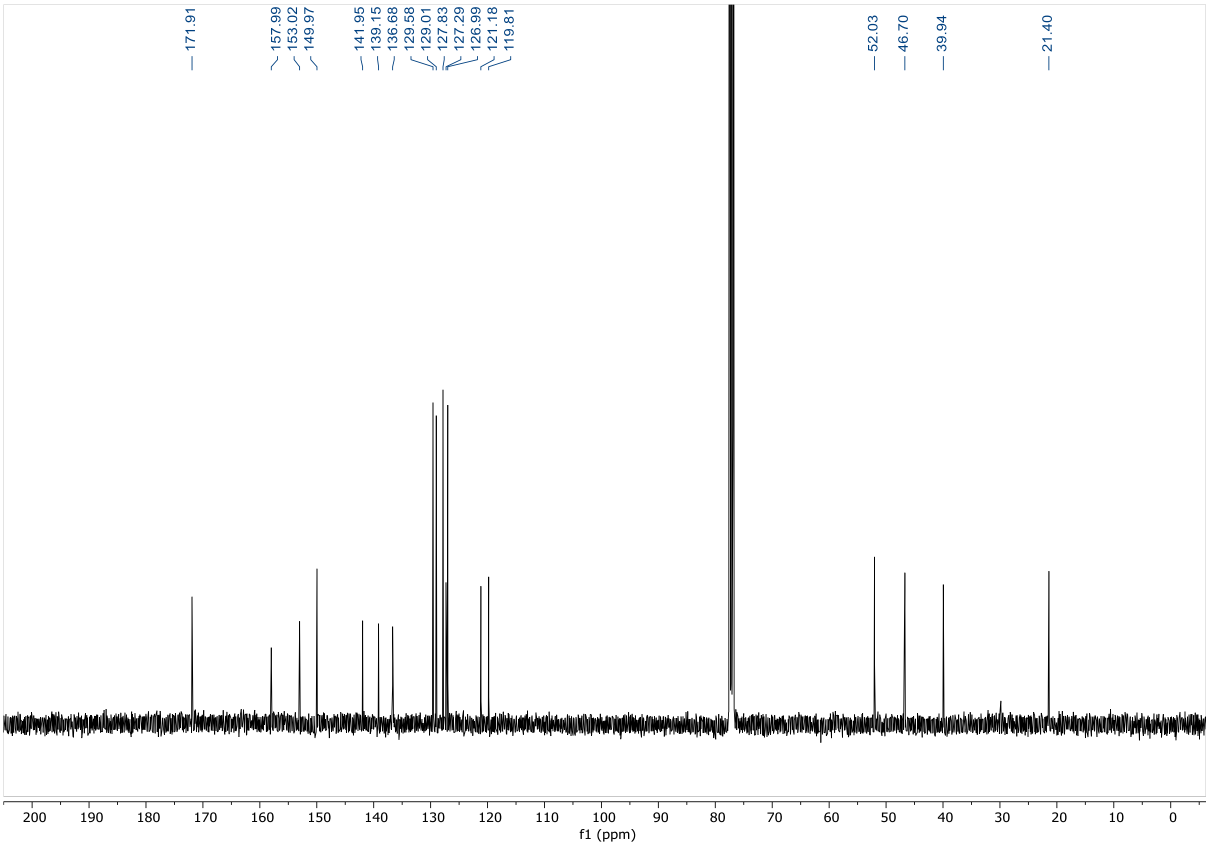

Supplementary Fig. 68. ^{1}H and ^{13}C NMR of **4d**.

Methyl (*R*)-3-(2-(4-methoxyphenyl)pyridin-4-yl)-3-phenylpropanoate (4e)

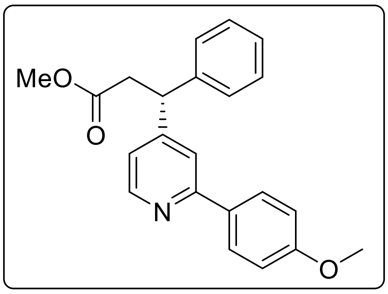

Prepared according to **GP**. Desired product **4e** was obtained as white solid (10.6 mg, 61% isolated yield). **^{1}H NMR** (500 MHz, $CDCl_3$) δ 8.54 (dd, *J* = 5.2, 0.8 Hz, 1H), 7.89 (d, *J* = 8.8 Hz, 2H), 7.53 (s, 1H), 7.34 – 7.29 (m, 2H), 7.26 – 7.21 (m, 3H), 7.04 (dd, *J* = 5.2, 1.6 Hz, 1H), 6.98 (d, *J* = 8.8 Hz, 2H), 4.58 (t, *J* = 7.9 Hz, 1H), 3.86 (s, 3H), 3.61 (s, 3H), 3.16 – 3.03 (m, 2H). **^{13}C NMR** (100 MHz, $CDCl_3$) δ 171.9, 160.6, 157.6, 153.0, 149.9, 142.0, 132.1, 129.0, 128.4, 127.8, 127.3, 120.8, 119.4, 114.2, 55.5, 52.0, 46.7, 39.9. **HRMS** (EI^+) m/z calcd. for $C_{22}H_{21}NO_3^+$ $[M]^+$: 347.1521, found: 347.1518. **Specific Rotation** $[\alpha]_D^{24}$ +12.41 (*c* 0.76, $CHCl_3$). **HPLC Analysis**. CHIRALCEL OD-H, 25 °C; *n*-hexane:*i*-PrOH = 75:25, 1.0 mL/min, 254 nm, t_{R1} (major) = 10.26 min, t_{R2} (minor) = 21.74 min, 94:6 er. The absolute stereochemistry was assigned by analogy to compound **3x** and **4k**.

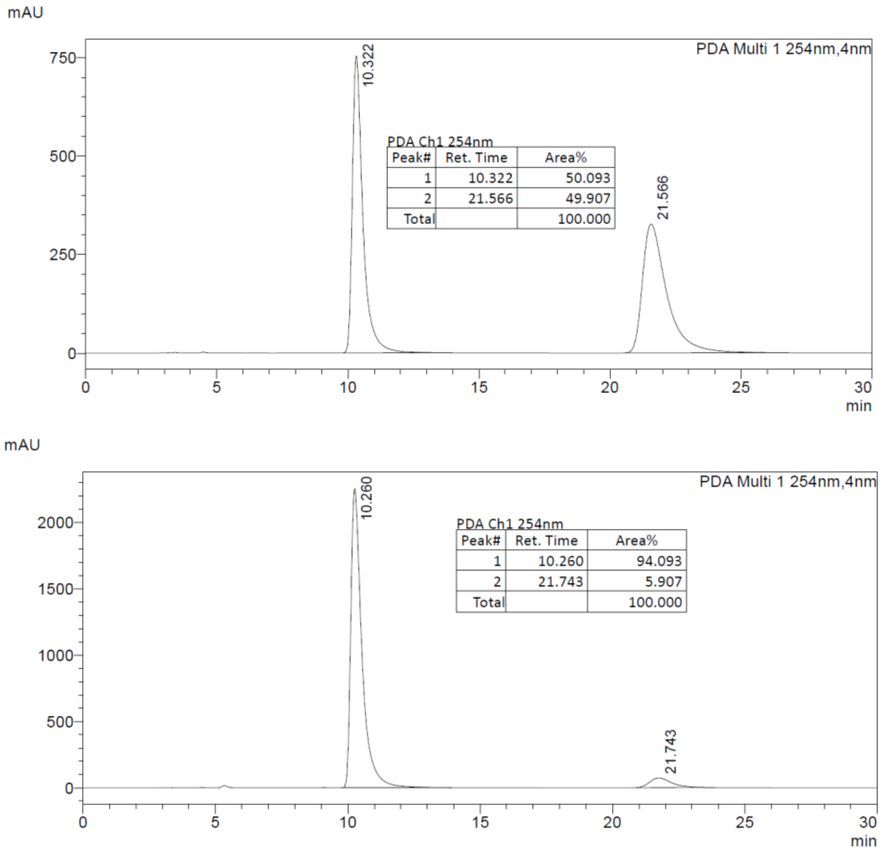

Supplementary Fig. 69. HPLC traces of *rac*-**4e** (top) and enantioenriched-**4e** (bottom).

500 MHz, ^{1}H NMR in CDCl$_3$

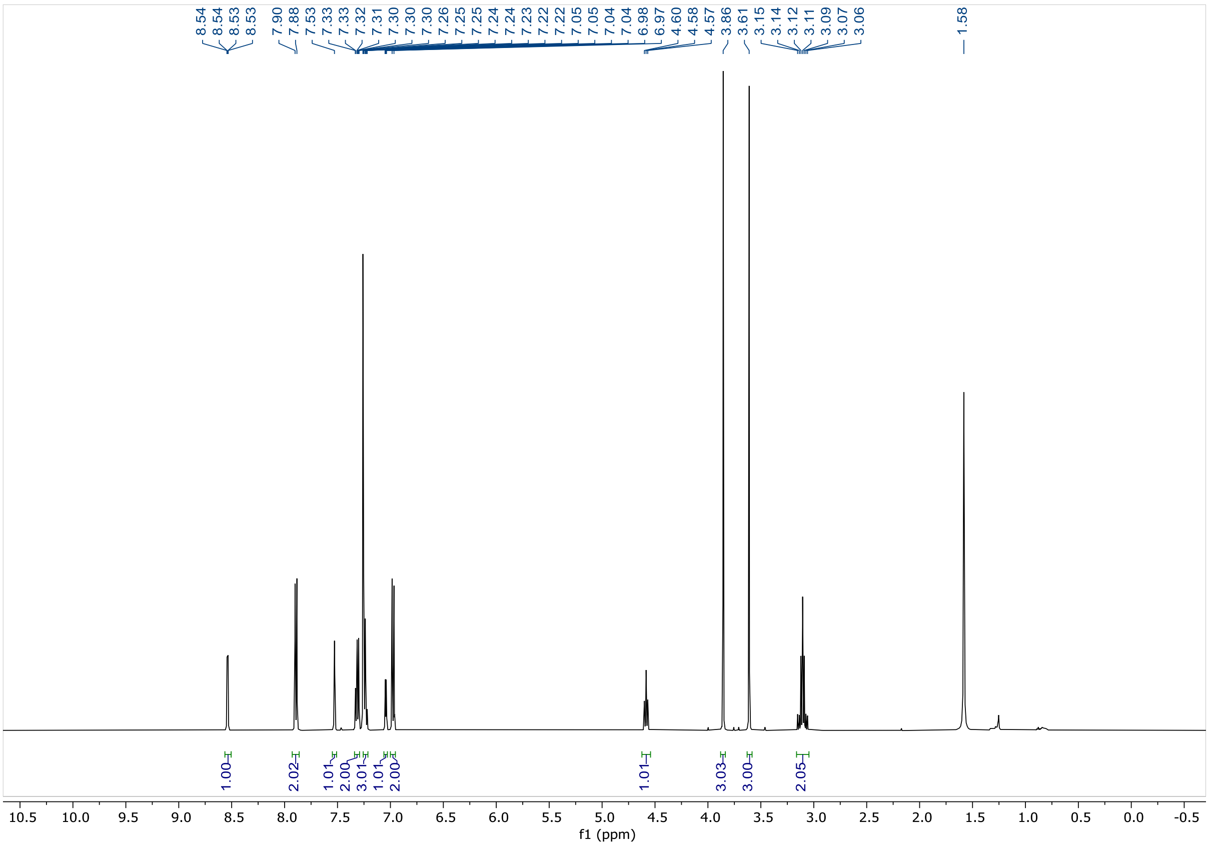

100 MHz, ^{13}C NMR in CDCl$_3$

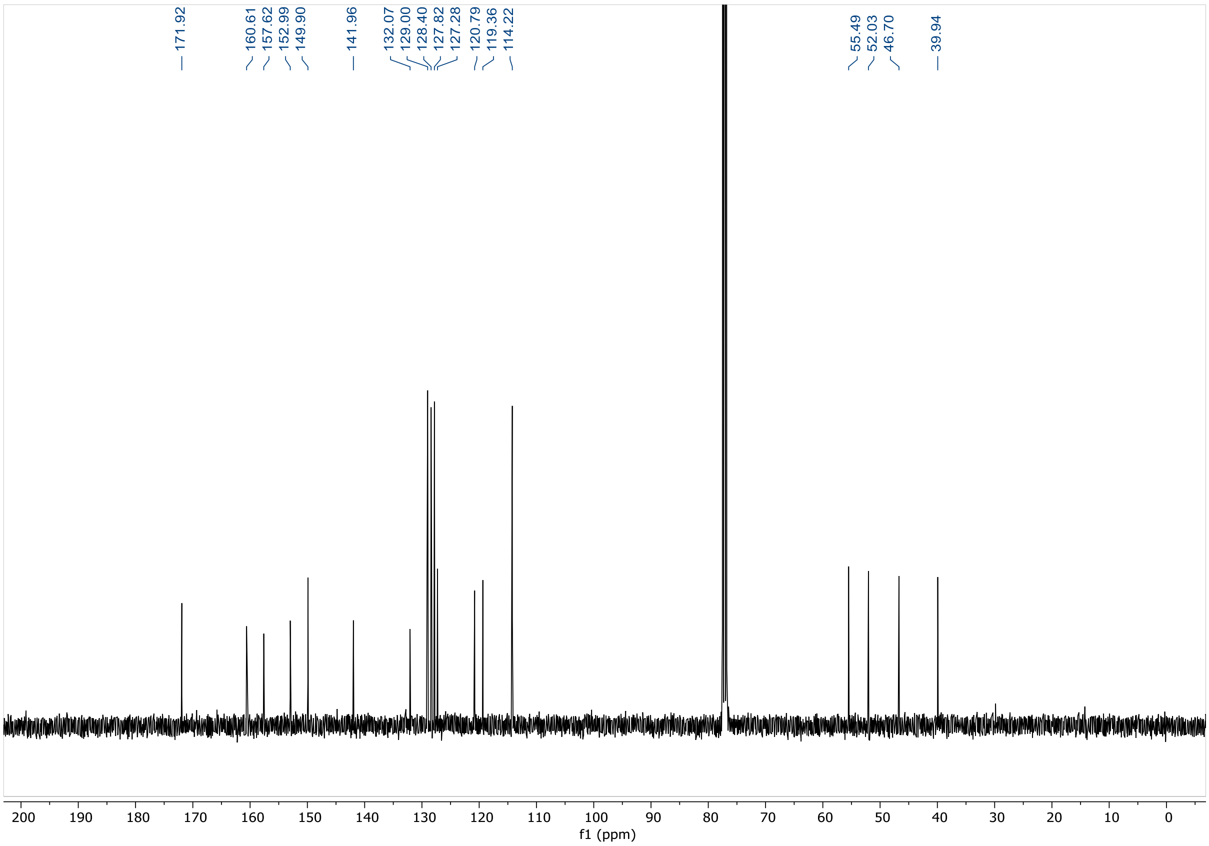

Supplementary Fig. 70. ^{1}H and ^{13}C NMR of **4e**.

Methyl (*R*)-3-phenyl-3-(2-(4-(trifluoromethyl)phenyl)pyridin-4-yl)propanoate (4f)

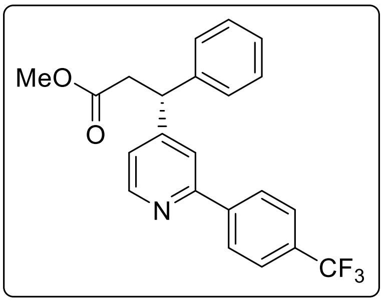

Prepared according to **GP**. Desired product **4f** was obtained as colorless gum (12.5 mg, 65% isolated yield). **^{1}H NMR** (500 MHz, $CDCl_3$) δ 8.62 (d, *J* = 5.1 Hz, 1H), 8.05 (d, *J* = 8.1 Hz, 2H), 7.71 (d, *J* = 8.2 Hz, 2H), 7.62 (d, *J* = 1.6 Hz, 1H), 7.36 – 7.29 (m, 2H), 7.26 – 7.23 (m, 3H), 7.17 (dd, *J* = 5.1, 1.6 Hz, 1H), 4.62 (t, *J* = 7.9 Hz, 1H), 3.62 (s, 3H), 3.32 – 2.99 (m, 2H). **^{13}C NMR** (100 MHz, $CDCl_3$) δ 171.8, 156.4, 153.5, 150.3, 142.7, 141.7, 131.0 (d, *J* = 32.3 Hz), 129.1, 127.8, 127.4, 127.4, 125.8 (q, *J* = 3.8 Hz), 124.3 (d, *J* = 271.9 Hz), 122.2, 120.5, 52.1, 46.7, 39.9. **^{19}F NMR** (376 MHz, $CDCl_3$) δ -62.6. **HRMS** (EI^+) m/z calcd. for $C_{22}H_{18}F_3NO_2^+$ $[M]^+$: 385.1290, found: 385.1289. **Specific Rotation** $[\alpha]_D^{25}$ +0.6 (*c* 1.0, $CHCl_3$). **HPLC Analysis**. CHIRALCEL OD-H, 25 ºC; *n*-hexane:*i*-PrOH = 75:25, 1.0 mL/min, 254 nm, t_{R1} (major) = 6.96 min, t_{R2} (minor) = 8.80 min, 96:4 er.

The absolute stereochemistry was assigned by analogy to compound **3x** and **4k**.

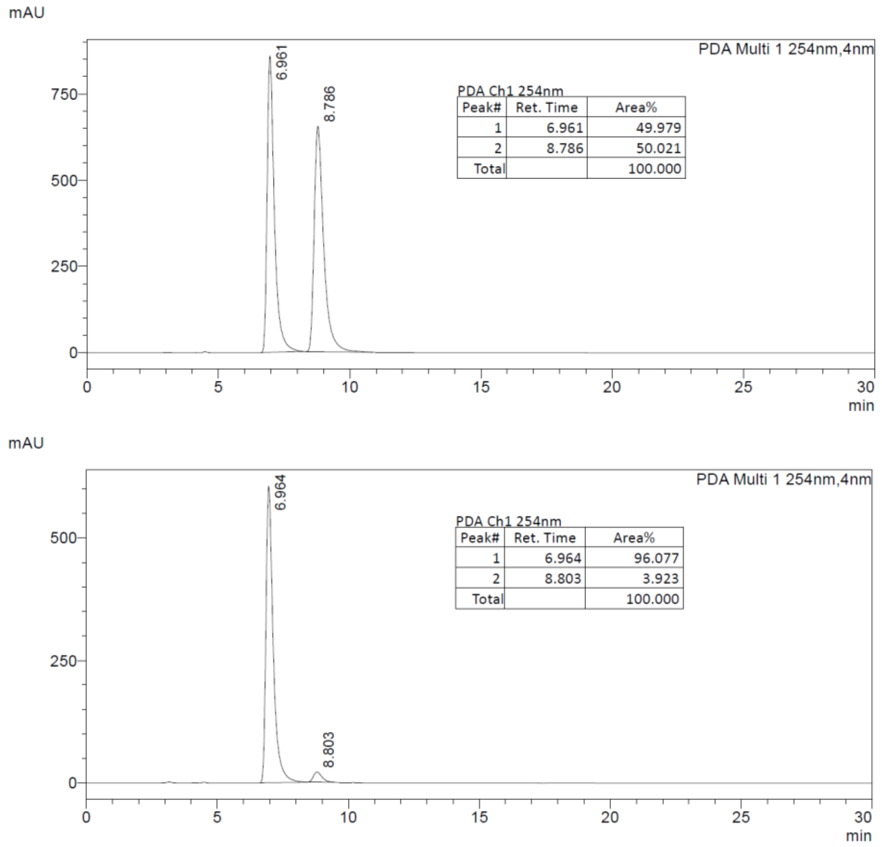

Supplementary Fig. 71. HPLC traces of *rac*-**4f** (top) and enantioenriched-**4f** (bottom).

500 MHz, ^{1}H NMR in $CDCl_3$

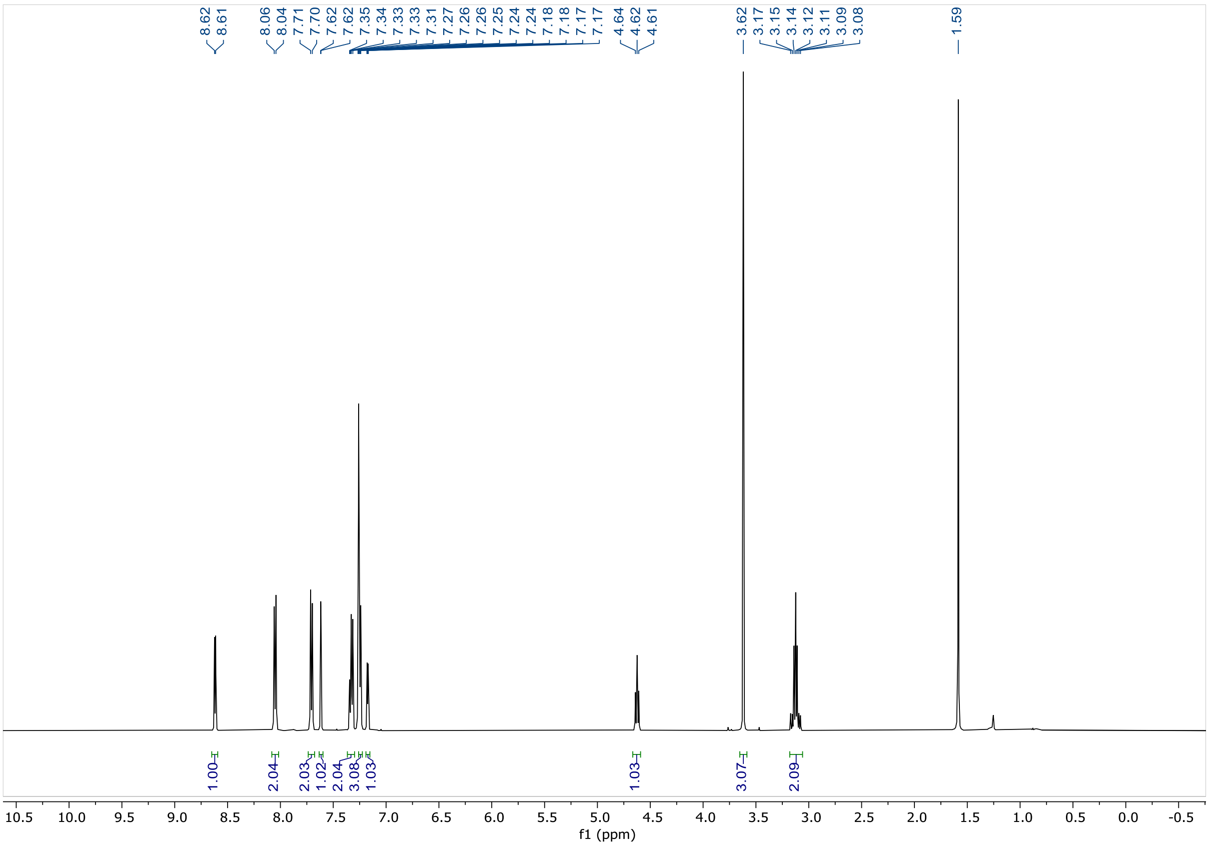

100 MHz, ^{13}C NMR in $CDCl_3$

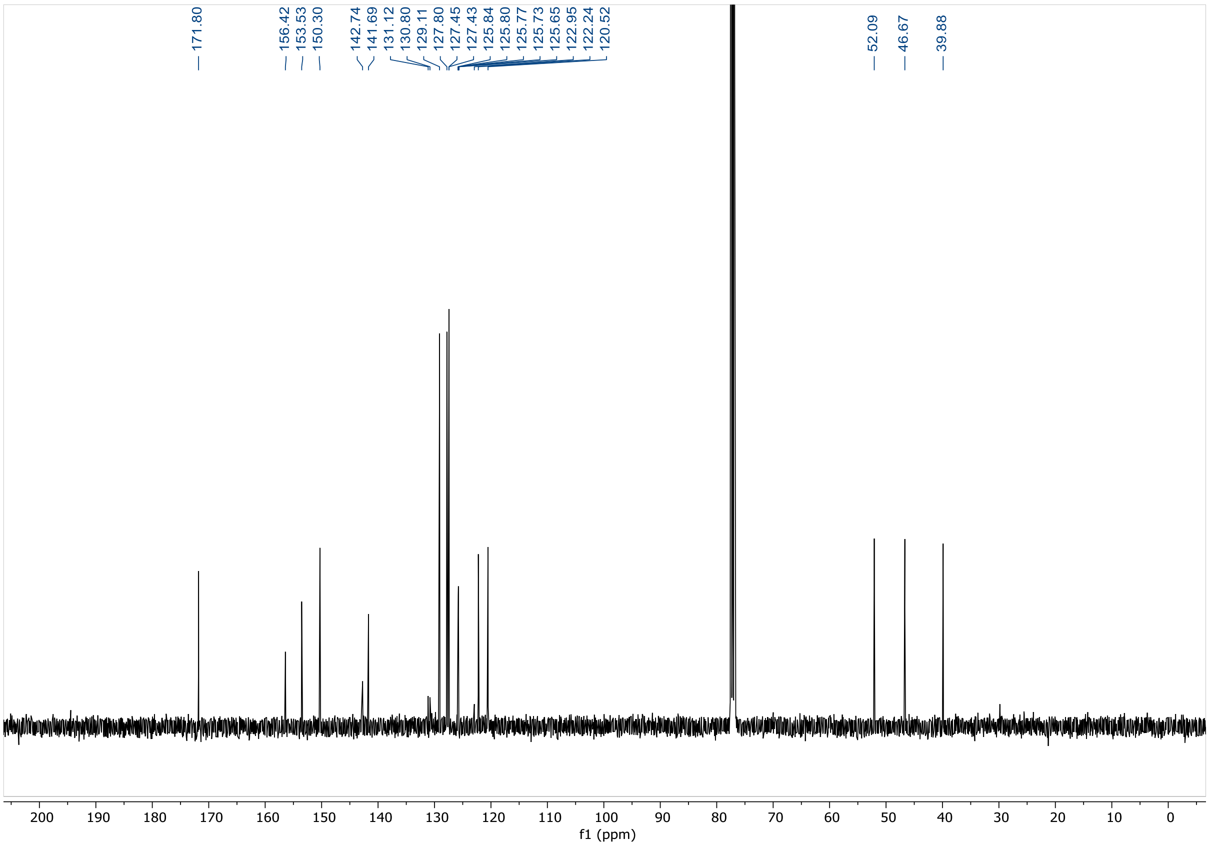

376 MHz, ^{19}F NMR in CDCl$_3$

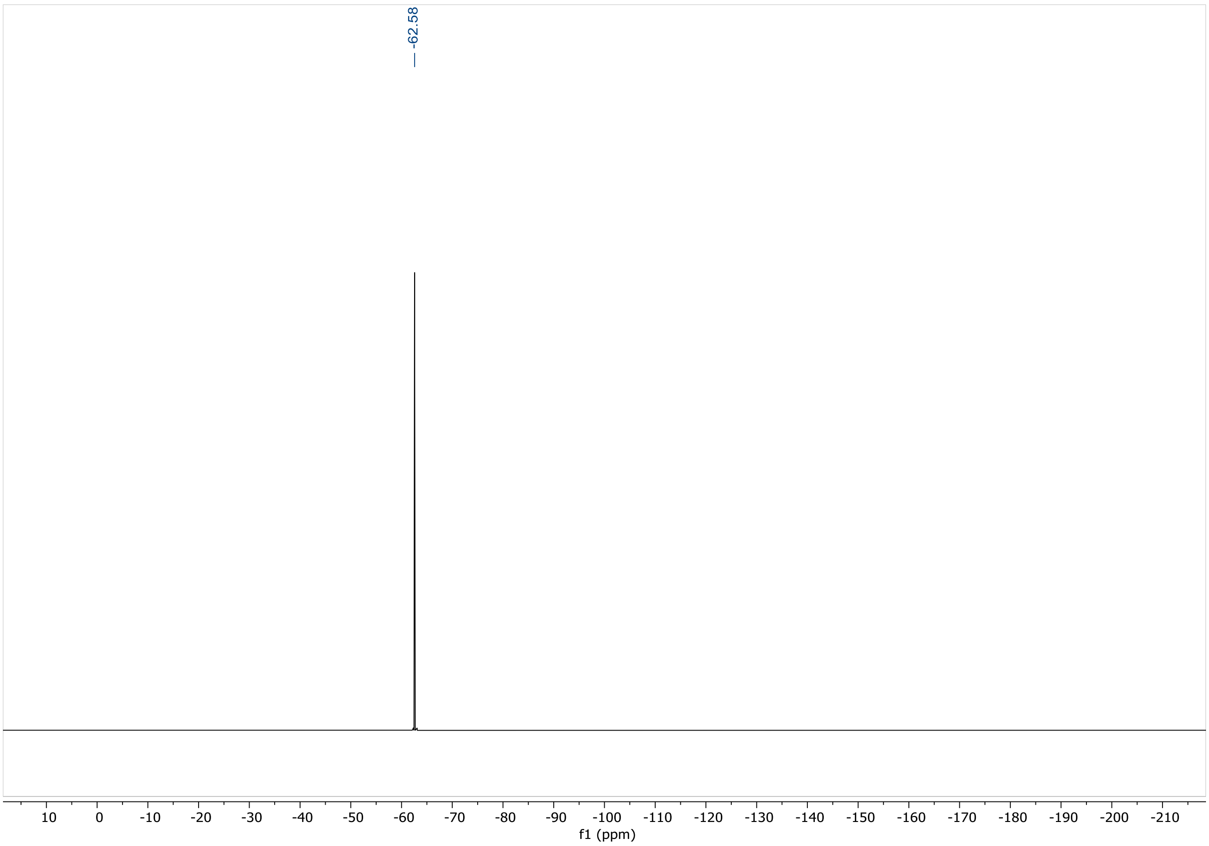

Supplementary Fig. 72. ^{1}H, ^{13}C, and ^{19}F NMR of **4f**.

Methyl (*R*)-3-phenyl-3-(2-(thiophen-2-yl)pyridin-4-yl)propanoate (4g)

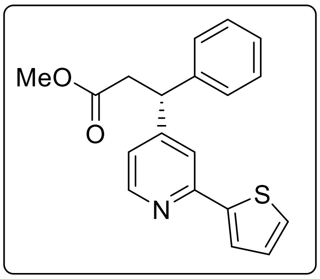

Prepared according to **GP**. Desired product **4g** was obtained as colorless gum (11.1 mg, 68% isolated yield). **^{1}H NMR** (500 MHz, $CDCl_3$) δ 8.46 (d, *J* = 5.1 Hz, 1H), 7.54 (dd, *J* = 3.7, 1.1 Hz, 1H), 7.52 (d, *J* = 1.1 Hz, 1H), 7.38 (dd, *J* = 5.0, 1.1 Hz, 1H), 7.32 (dd, *J* = 8.4, 7.0 Hz, 2H), 7.26 – 7.22 (m, 3H), 7.09 (dd, *J* = 5.0, 3.7 Hz, 1H), 7.01 (dd, *J* = 5.2, 1.7 Hz, 1H), 4.57 (t, *J* = 7.9 Hz, 1H), 3.61 (s, 3H), 3.15 – 3.04 (m, 2H). **^{13}C NMR** (100 MHz, $CDCl_3$) δ 171.8, 153.1, 153.0, 149.9, 144.8, 141.7, 129.0, 128.1, 127.8, 127.4, 124.7, 121.3, 118.2, 52.1, 46.6, 39.9. **HRMS** (EI^+) m/z calcd. for $C_{19}H_{17}NO_2S^+$ $[M]^+$: 323.0980, found: 323.0982. **Specific Rotation** $[\alpha]_D^{25}$ +0.41 (*c* 0.78, $CHCl_3$). **HPLC Analysis**. CHIRALCEL OD-H, 25 °C; *n*-hexane:*i*-PrOH = 75:25, 1.0 mL/min, 254 nm, t_{R1} (major) = 9.13 min, t_{R2} (minor) = 20.12 min, 96:4 er.

The absolute stereochemistry was assigned by analogy to compound **3x** and **4k**.

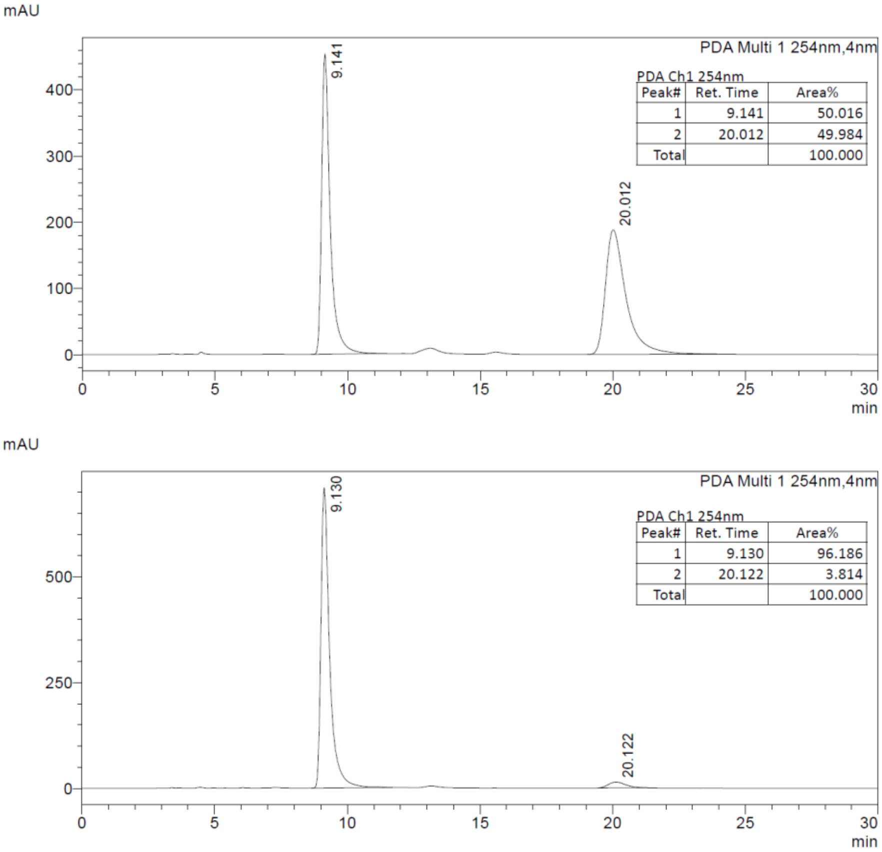

Supplementary Fig. 73. HPLC traces of *rac*-**4g** (top) and enantioenriched-**4g** (bottom).

500 MHz, ^{1}H NMR in $CDCl_3$

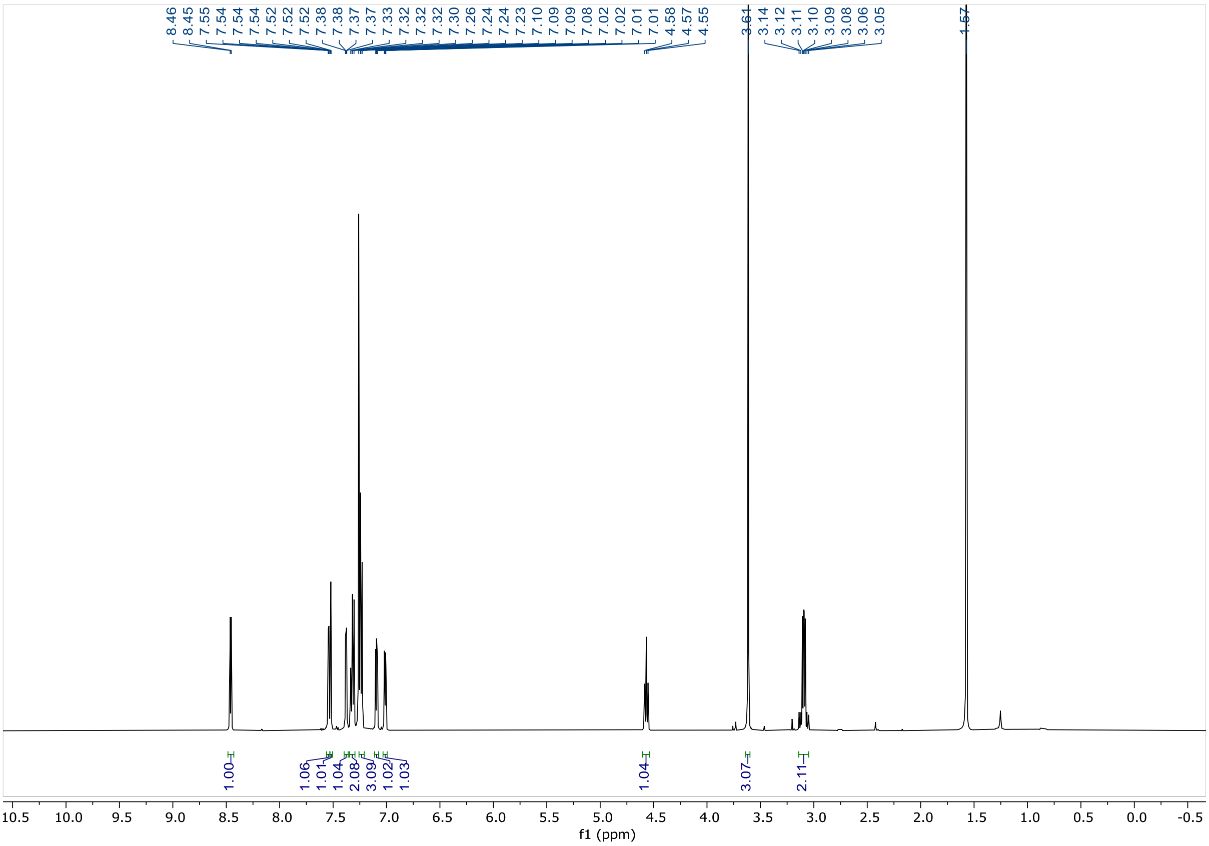

100 MHz, ^{13}C NMR in $CDCl_3$

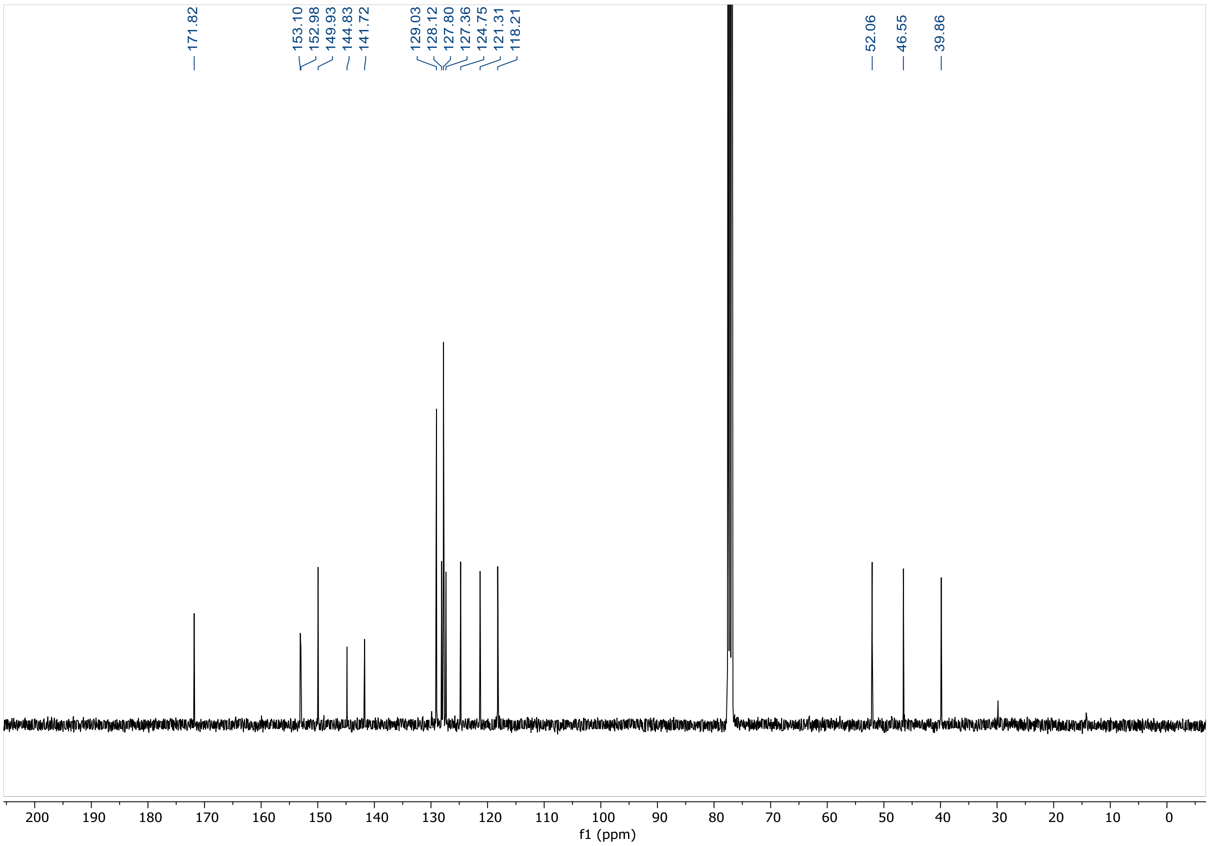

Supplementary Fig. 74. ^{1}H and ^{13}C NMR of **4g**.

Methyl (*R*)-3-([2,2'-bipyridin]-4-yl)-3-phenylpropanoate (4h)

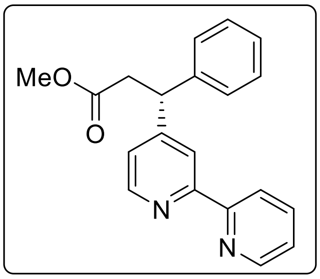

Prepared according to **GP**. Desired product **4h** was obtained as colorless gum (10.5 mg, 66% isolated yield). **^{1}H NMR** (400 MHz, $CDCl_3$) δ 8.67 (ddd, *J* = 4.9, 1.8, 0.9 Hz, 1H), 8.57 (d, *J* = 5.1 Hz, 1H), 8.39 – 8.30 (m, 2H), 7.80 (td, *J* = 7.7, 1.8 Hz, 1H), 7.33 – 7.27 (m, 5H), 7.25 – 7.19 (m, 1H), 7.17 (dd, *J* = 5.1, 1.8 Hz, 1H), 4.64 (t, *J* = 7.9 Hz, 1H), 3.60 (s, 3H), 3.24 – 3.07 (m, 2H). **^{13}C NMR** (100 MHz, $CDCl_3$) δ 171.9, 156.6, 156.2, 153.4, 149.6, 149.3, 142.0, 137.1, 129.0, 127.9, 127.2, 123.9, 123.2, 121.4, 120.3, 52.0, 46.8, 39.9. **HRMS** (EI^+) m/z calcd. for $C_{20}H_{18}N_2O_2^+$ $[M]^+$: 318.1368, found: 318.1366. **Specific Rotation** $[\alpha]_D^{25}$ -15.7 (*c* 0.73, $CHCl_3$). **HPLC Analysis**. CHIRALCEL OD-H, 25 °C; *n*-hexane:*i*-PrOH = 75:25, 1.0 mL/min, 254 nm, t_{R1} (major) = 9.92 min, t_{R2} (minor) = 16.49 min, 95:5 er.

The absolute stereochemistry was assigned by analogy to compound **3x** and **4k**.

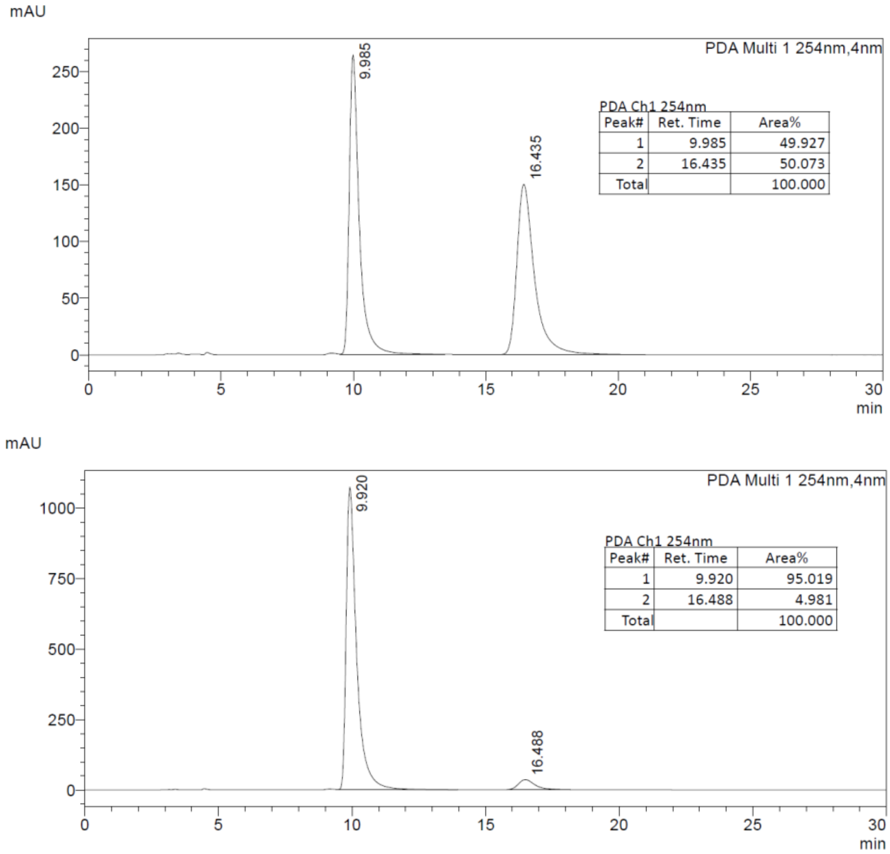

Supplementary Fig. 75. HPLC traces of *rac*-**4h** (top) and enantioenriched-**4h** (bottom).

400 MHz, ^{1}H NMR in $CDCl_3$

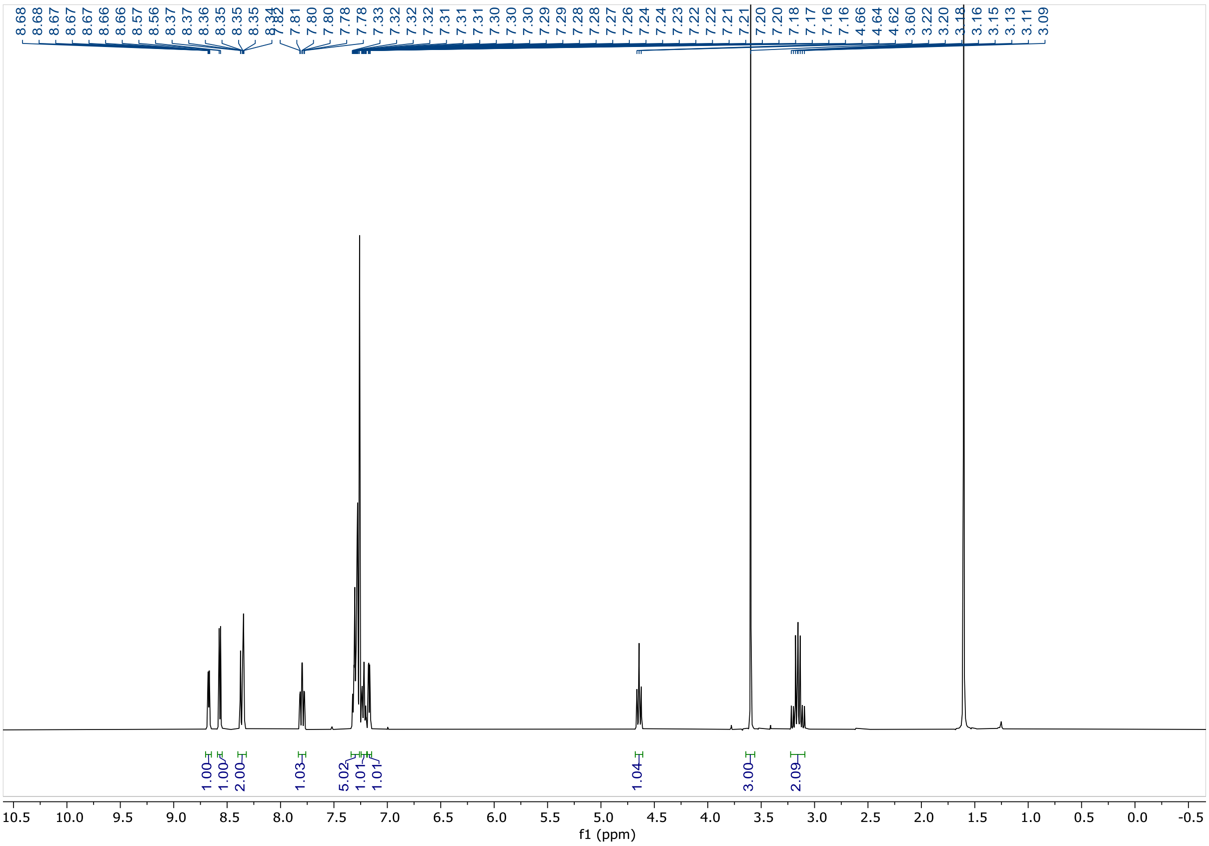

100 MHz, ^{13}C NMR in $CDCl_3$

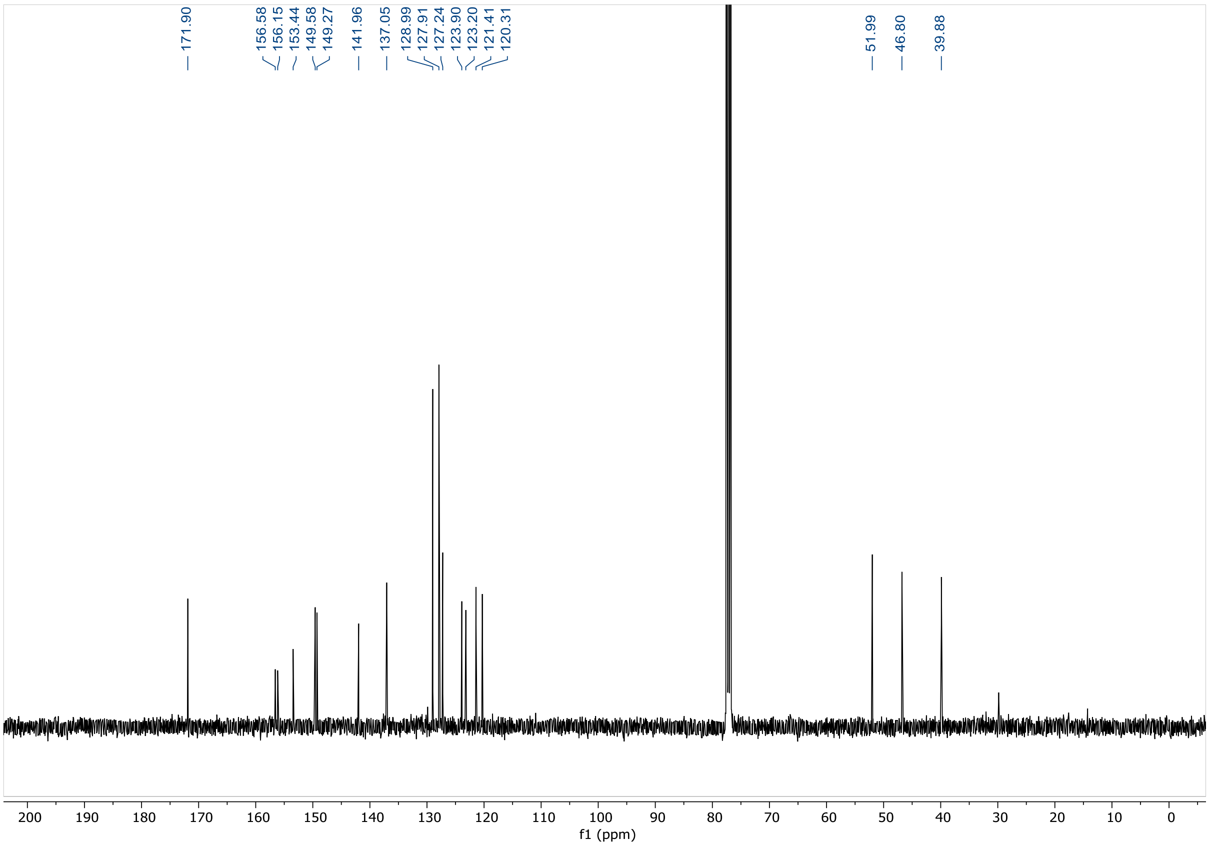

Supplementary Fig. 76. ^{1}H and ^{13}C NMR of **4h**.

Methyl (*R*)-3-(2-(3-bromophenyl)pyridin-4-yl)-3-phenylpropanoate (4i)

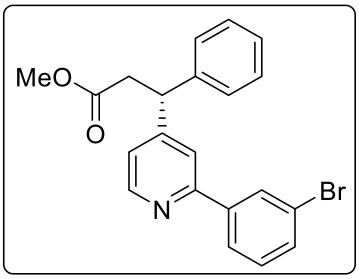

Prepared according to **GP**. Desired product **4i** was obtained as colorless gum (13.9 mg, 70% isolated yield). **^{1}H NMR** (500 MHz, $CDCl_3$) δ 8.59 (dd, *J* = 5.2, 0.8 Hz, 1H), 8.11 (t, *J* = 1.9 Hz, 1H), 7.85 (ddd, *J* = 7.9, 1.7, 1.0 Hz, 1H), 7.57 – 7.55 (m, 1H), 7.53 (ddd, *J* = 7.9, 2.0, 1.0 Hz, 1H), 7.36 – 7.30 (m, 3H), 7.26 – 7.22 (m, 3H), 7.14 (dd, *J* = 5.2, 1.7 Hz, 1H), 4.61 (t, *J* = 7.9 Hz, 1H), 3.62 (s, 3H), 3.17 – 3.06 (m, 2H). **^{13}C NMR** (100 MHz, $CDCl_3$) δ 171.8, 156.4, 153.4, 150.2, 141.8, 141.5, 132.1, 130.4, 130.2, 129.1, 127.8, 127.4, 125.7, 123.1, 122.0, 120.2, 52.1, 46.7, 39.9. **HRMS** (EI^+) m/z calcd. for $C_{21}H_{18}BrNO_2^+$ $[M]^+$: 395.0521, found: 395.0523. **Specific Rotation** $[\alpha]_D^{25}$ -7.6 (*c* 1.0, $CHCl_3$). **HPLC Analysis**. CHIRALCEL OD-H, 25 °C; *n*-hexane:*i*-PrOH = 75:25, 1.0 mL/min, 254 nm, t_{R1} (major) = 7.90 min, t_{R2} (minor) = 13.69 min, 96:4 er.

The absolute stereochemistry was assigned by analogy to compound **3x** and **4k**.

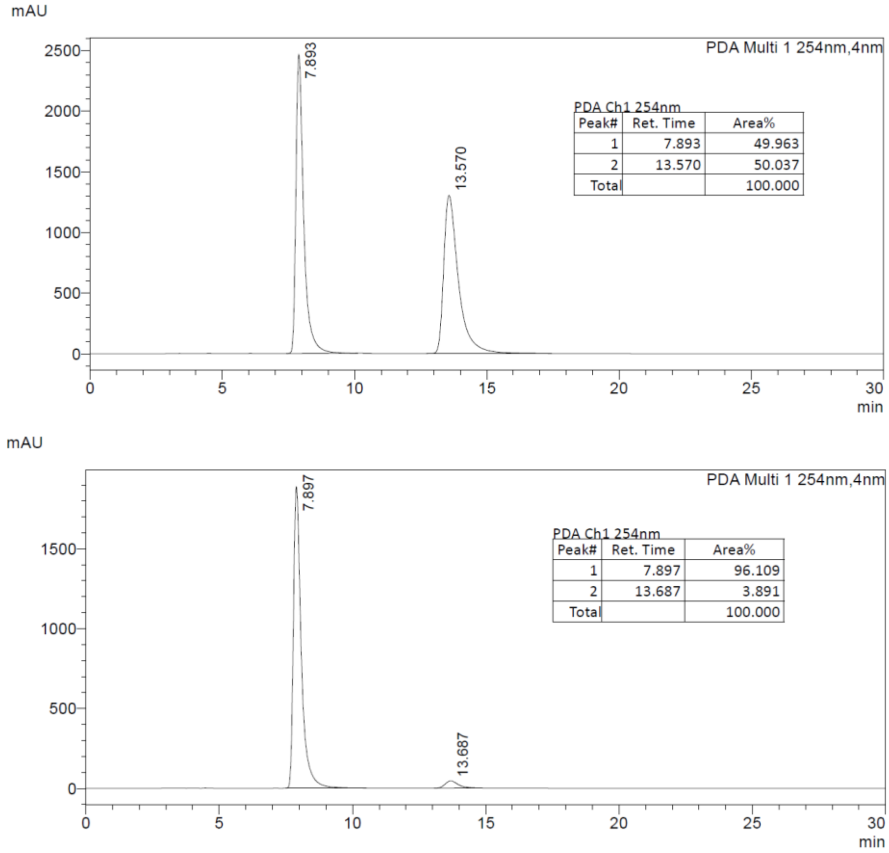

Supplementary Fig. 77. HPLC traces of *rac*-**4i** (top) and enantioenriched-**4i** (bottom).

500 MHz, ^{1}H NMR in $CDCl_3$

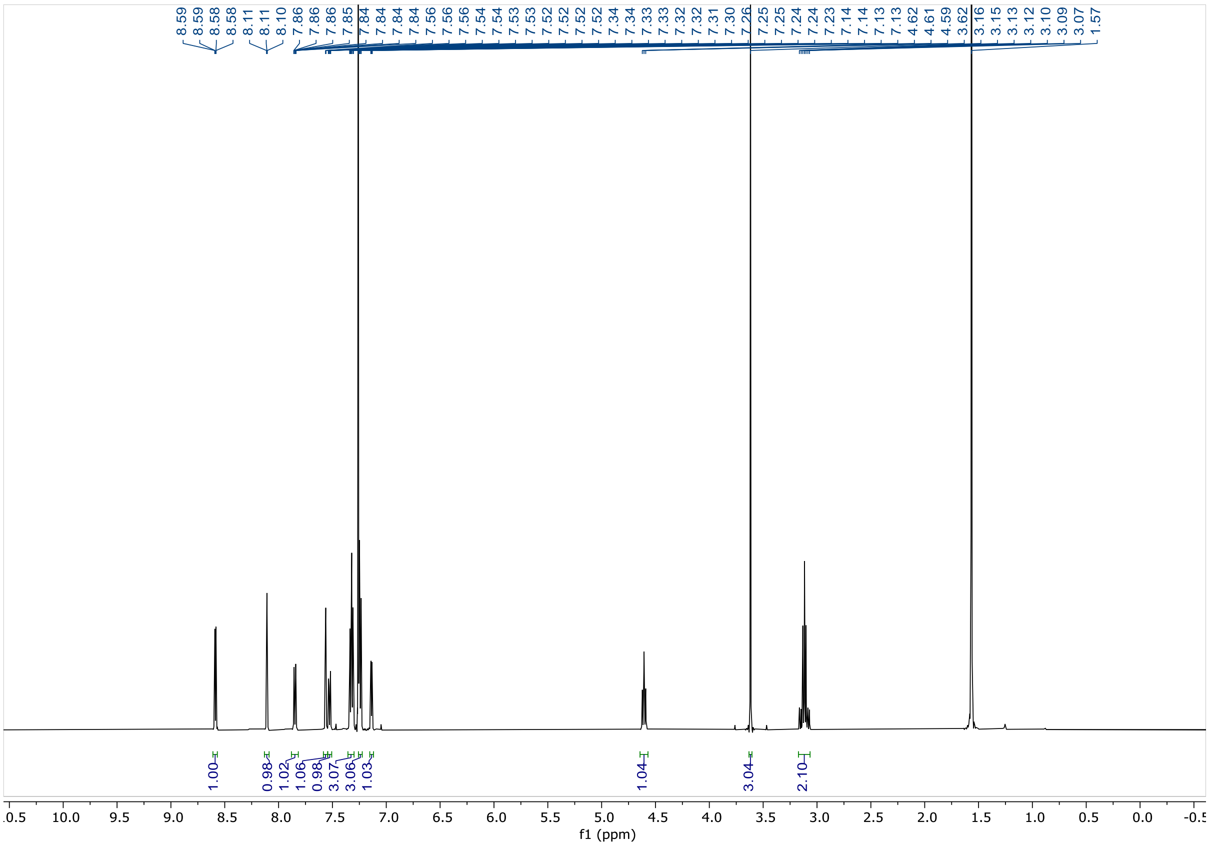

100 MHz, ^{13}C NMR in $CDCl_3$

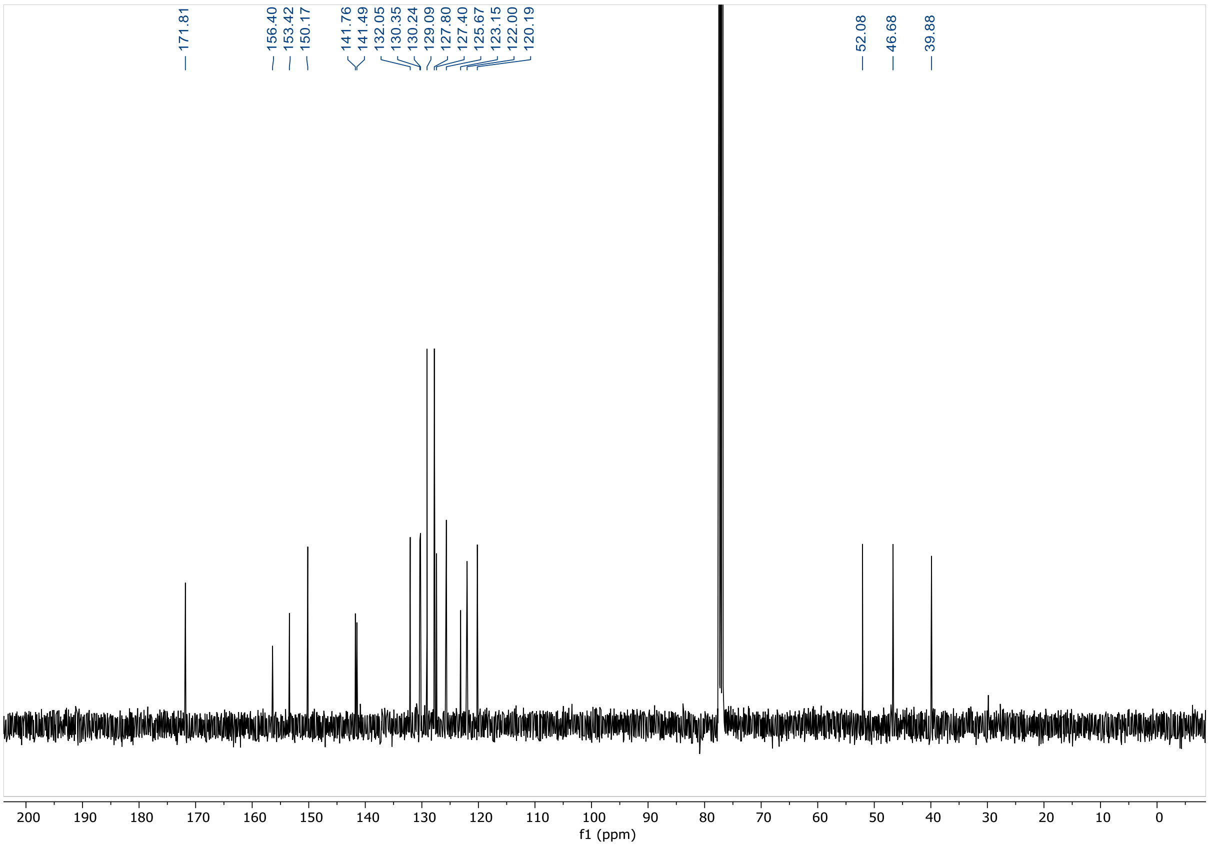

Supplementary Fig. 78. ^{1}H and ^{13}C NMR of **4i**.

Methyl (*R*)-3-(2-(4-bromophenyl)pyridin-4-yl)-3-phenylpropanoate (4j)

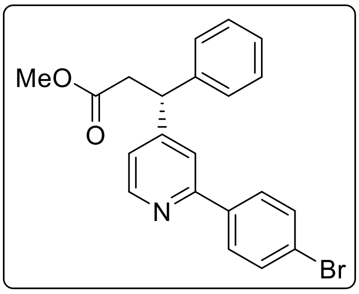

Prepared according to **GP**. Desired product **4j** was obtained as white solid (13.1 mg, 66% isolated yield). **^{1}H NMR** (500 MHz, $CDCl_3$) δ 8.57 (d, *J* = 5.2 Hz, 1H), 7.81 (d, *J* = 8.5 Hz, 2H), 7.58 (d, *J* = 8.6 Hz, 2H), 7.56 (d, *J* = 1.6 Hz, 1H), 7.36 – 7.30 (m, 2H), 7.26 – 7.22 (m, 3H), 7.12 (dd, *J* = 5.1, 1.7 Hz, 1H), 4.60 (t, *J* = 7.9 Hz, 1H), 3.61 (s, 3H), 3.22 – 2.99 (m, 2H). **^{13}C NMR** (100 MHz, $CDCl_3$) δ 171.8, 156.8, 153.4, 150.1, 141.8, 138.3, 132.0, 129.1, 128.7, 127.8, 127.4, 123.6, 121.7, 119.9, 52.1, 46.7, 39.9. **HRMS** (EI^+) m/z calcd. for $C_{21}H_{18}BrNO_2^+$ $[M]^+$: 395.0521, found: 395.0518. **Specific Rotation** $[\alpha]_D^{25}$ +4.8 (*c* 0.64, $CHCl_3$). **HPLC Analysis**. CHIRALCEL OD-H, 25 °C; *n*-hexane:*i*-PrOH = 75:25, 1.0 mL/min, 254 nm, t_{R1} (major) = 8.68 min, t_{R2} (minor) = 15.47 min, 95:5 er.

The absolute stereochemistry was assigned by analogy to compound **3x** and **4k**.

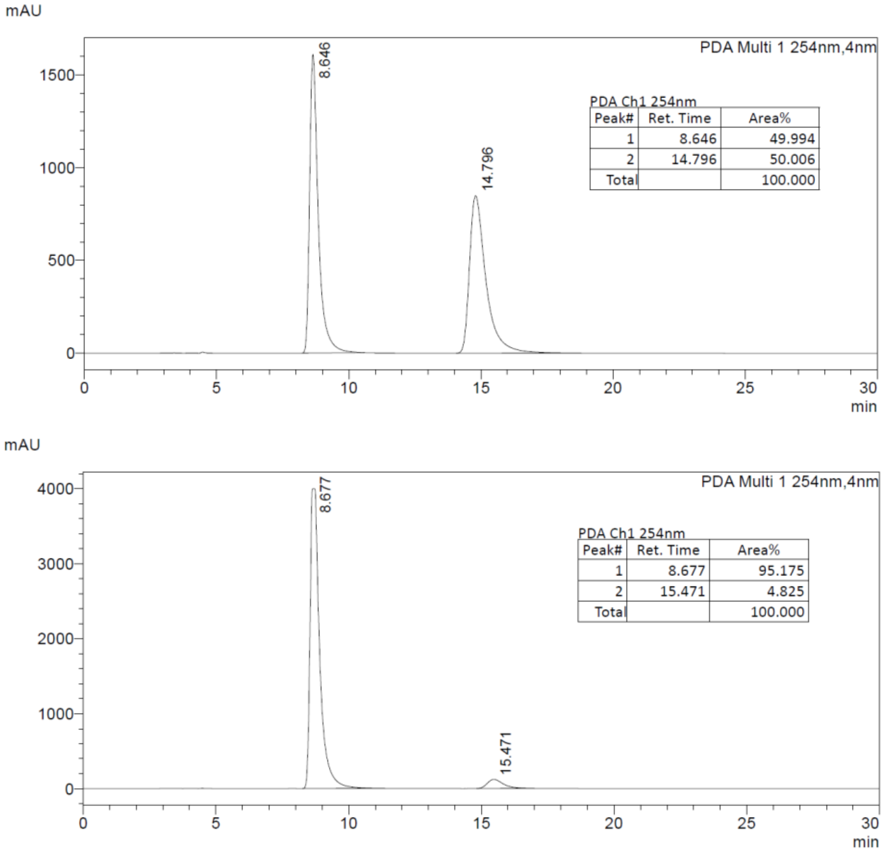

Supplementary Fig. 79. HPLC traces of *rac*-**4j** (top) and enantioenriched-**4j** (bottom).

500 MHz, ^{1}H NMR in $CDCl_3$

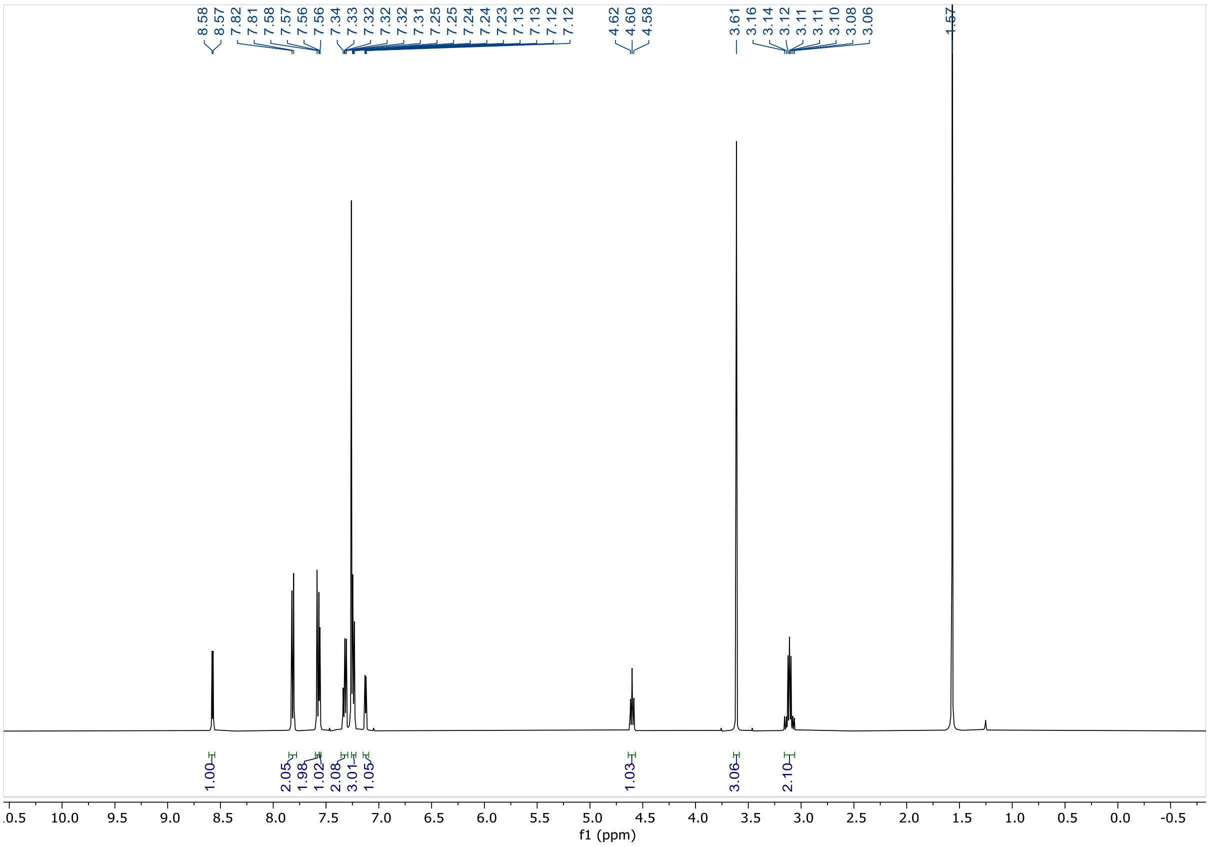

100 MHz, ^{13}C NMR in $CDCl_3$

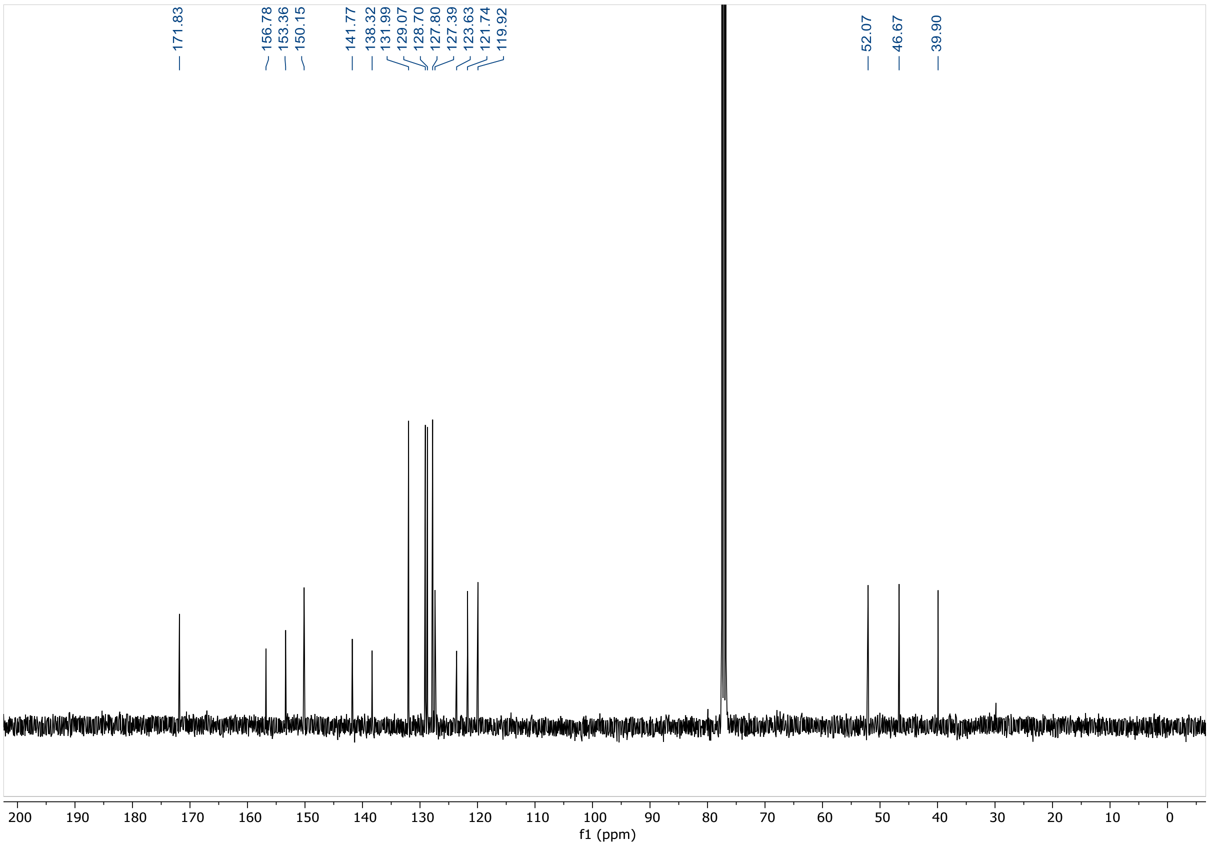

Supplementary Fig. 80. ^{1}H and ^{13}C NMR of **4j**.

Methyl (*R*)-3-(4-bromophenyl)-3-(2-(4-bromophenyl)pyridin-4-yl)propanoate (4k)

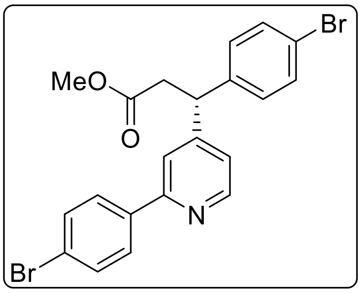

Prepared according to **GP**. Desired product **4k** was obtained as white solid (11.2 mg, 47% isolated yield). **^{1}H NMR** (400 MHz, $CDCl_3$) δ 8.58 (d, *J* = 5.1 Hz, 1H), 7.81 (d, *J* = 8.6 Hz, 2H), 7.58 (d, *J* = 8.6 Hz, 2H), 7.52 (s, 1H) 7.45 (d, *J* = 8.6 Hz, 2H), 7.16 – 7.05 (m, 3H), 4.60 – 4.52 (m, 1H), 3.62 (s, 3H), 3.15 – 2.99 (m, 2H). **^{13}C NMR** (100 MHz, $CDCl_3$) δ 171.5, 156.9, 152.7, 150.3, 140.8, 138.2, 132.2, 132.0, 129.6, 128.7, 123.8, 121.6, 121.4, 119.7, 52.2, 46.1, 39.7. **HRMS** (ESI^+) m/z calcd. For $C_{21}H_{18}Br_2NO_2^+$ $[M+H]^+$: 473.9704, found 473.9703. **Specific Rotation** $[\alpha]_D^{26}$ +11.0 (*c* 1.0, $CHCl_3$). **HPLC Analysis**. CHIRALCEL OD-H, 25 ºC; *n*-hexane:*i*-PrOH = 75:25, 1.0 mL/min, 254 nm, t_{R1} (major) = 10.58 min, t_{R2} (minor) = 19.89 min, 94:6 er.

The absolute stereochemistry was assigned by X-ray crystallography analysis.

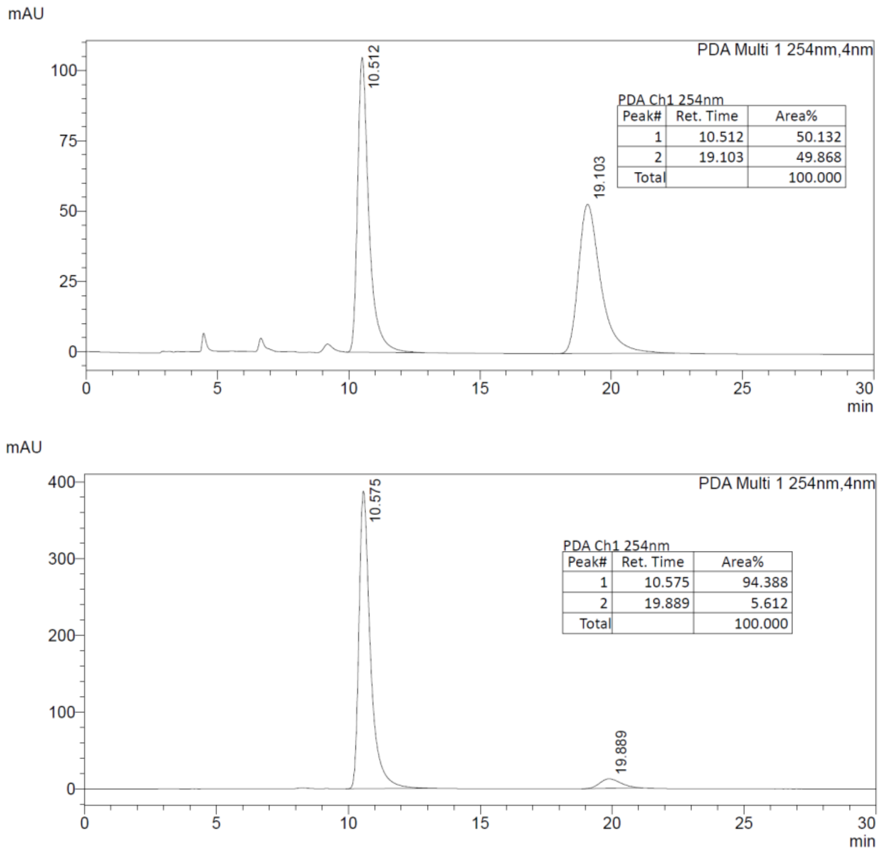

Supplementary Fig. 81. HPLC traces of *rac*-**4k** (top) and enantioenriched-**4k** (bottom).

400 MHz, ^{1}H NMR in $CDCl_3$.

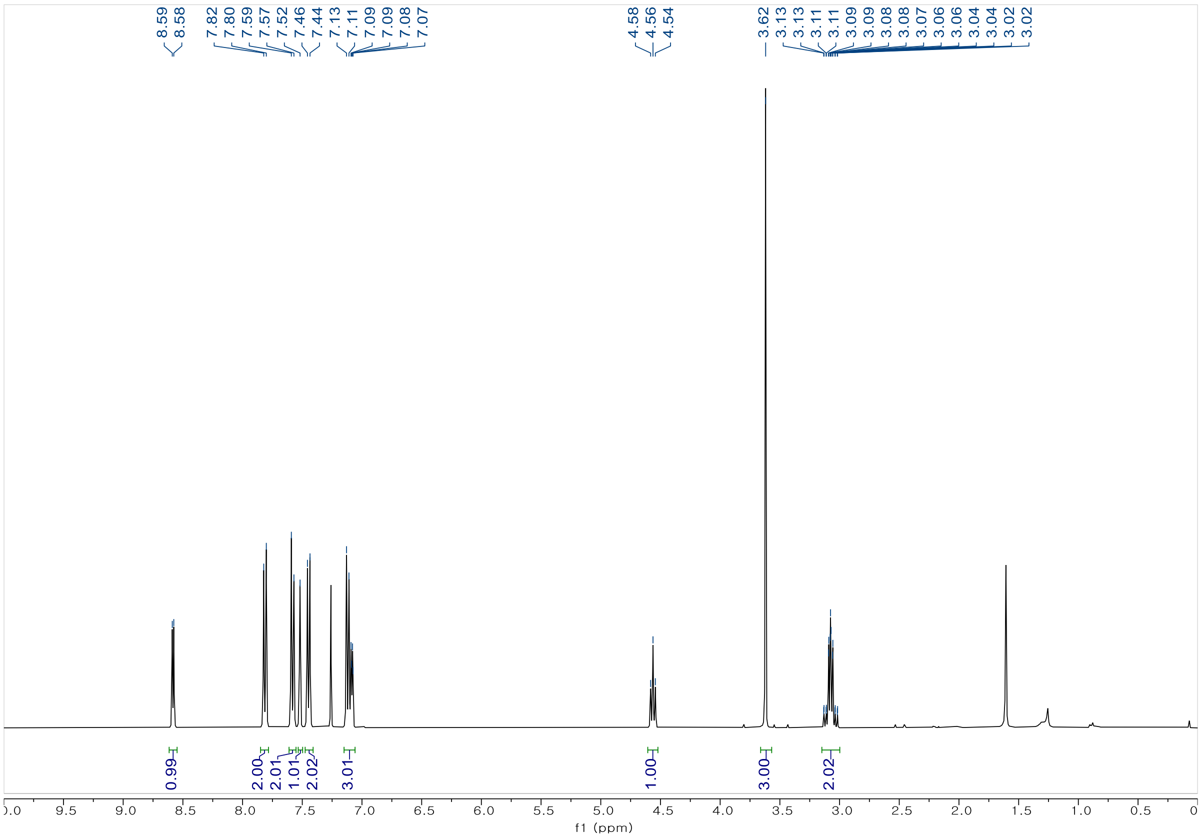

100 MHz, ^{13}C NMR in $CDCl_3$.

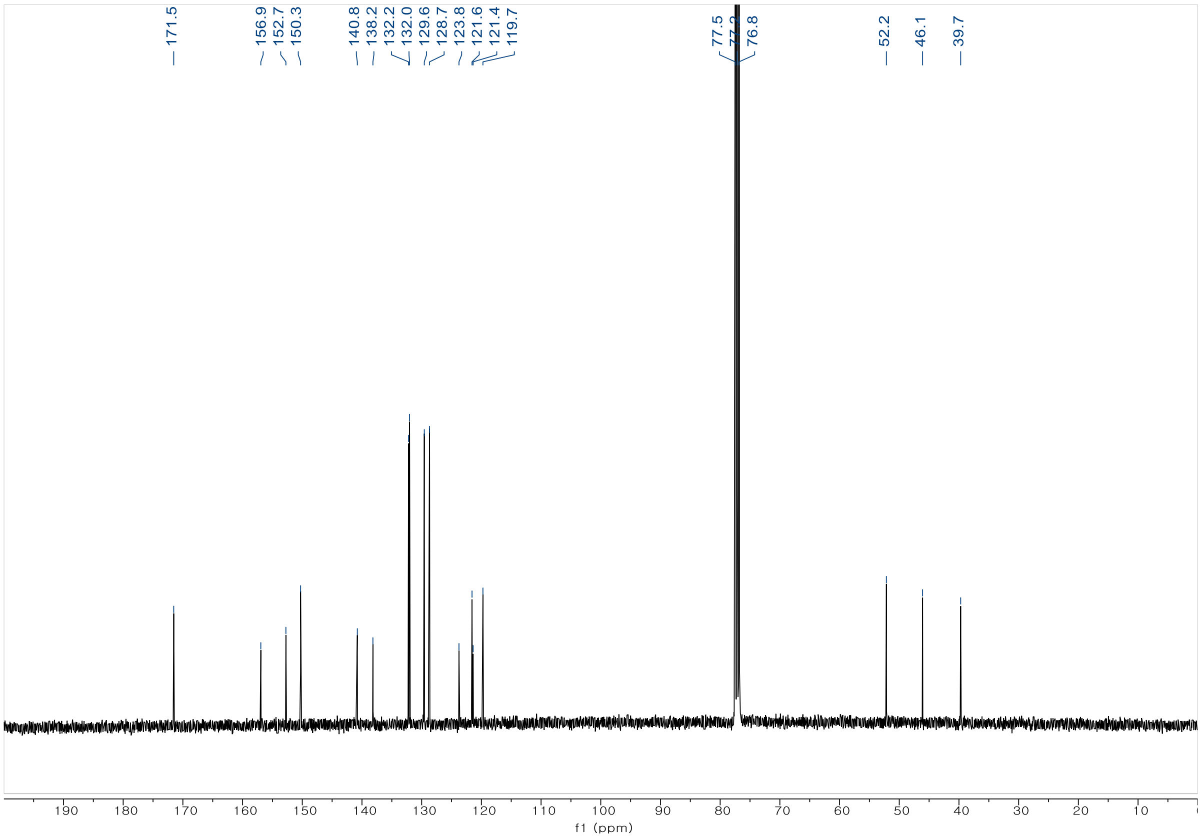

Supplementary Fig. 82. ^{1}H and ^{13}C NMR of **4k**.

Ethyl (*R*)-3-phenyl-3-(2-phenylpyridin-4-yl)propanoate (4l)

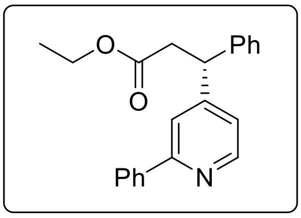

Prepared according to **GP**. Desired product **4l** was obtained as colorless oil (10.4 mg, 63% isolated yield). **^{1}H NMR** (400 MHz, $CDCl_3$) δ 8.59 (dd, *J* = 5.1, 0.8 Hz, 1H), 7.96 – 7.91 (m, 2H), 7.61 – 7.59 (m, 1H), 7.49 – 7.43 (m, 2H), 7.43 – 7.37 (m, 1H), 7.35 – 7.29 (m, 2H), 7.26 (s, 1H), 7.26 – 7.21 (m, 2H), 7.11 (dd, *J* = 5.1, 1.7 Hz, 1H), 4.60 (t, *J* = 8.0 Hz, 1H), 4.06 (q, *J* = 7.1 Hz, 2H), 3.18 – 3.02 (m, 2H), 1.13 (t, *J* = 7.1 Hz, 3H). **^{13}C NMR** (125 MHz, $CDCl_3$) δ 171.4, 158.0, 153.2, 150.0, 141.9, 139.5, 129.1, 129.0, 128.9, 127.9, 127.3, 127.1, 121.5, 120.2, 60.9, 46.8, 40.2, 14.2. **HRMS** (ESI^+) m/z calcd. For $C_{22}H_{22}NO_2^+$ $[M+H]^+$: 332.1651, found 332.1650. **Specific Rotation** $[\alpha]_D^{25}$ -4.2 (*c* 1.0, $CHCl_3$). **HPLC Analysis**. CHIRALCEL OD-H, 25 ºC; *n*-hexane:*i*-PrOH = 75:25, 1.0 mL/min, 254 nm, t_{R1} (major) = 6.65 min, t_{R2} (minor) = 10.45 min, 95:5 er.

The absolute stereochemistry was assigned by analogy to compound **3x** and **4k**.

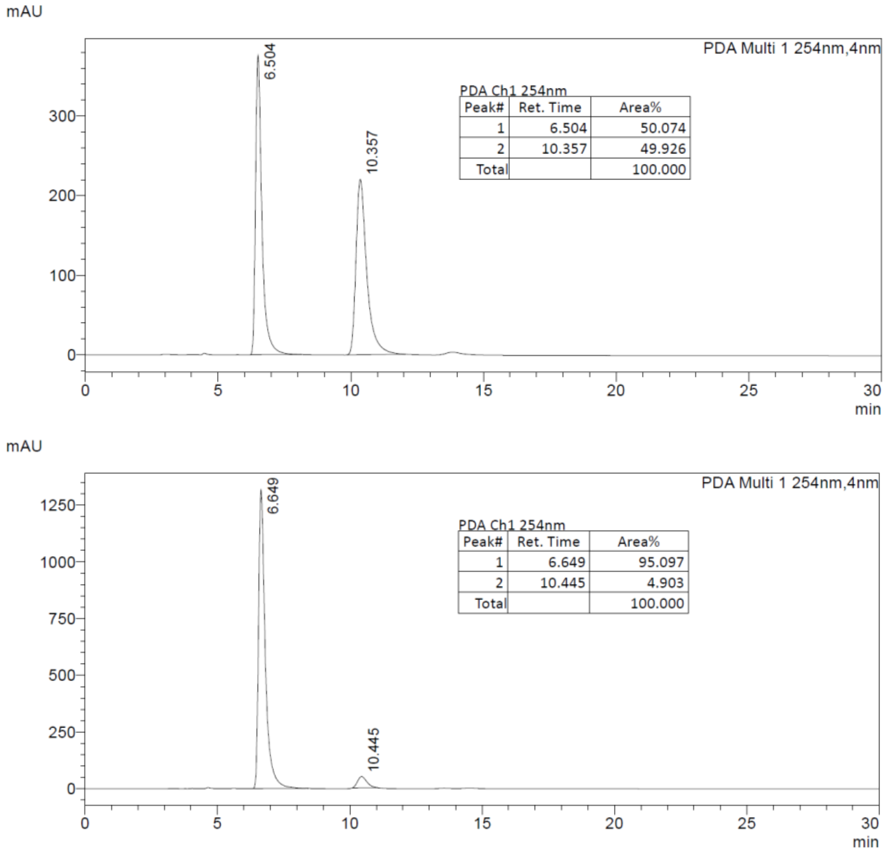

Supplementary Fig. 83. HPLC traces of *rac*-**4l** (top) and enantioenriched-**4l** (bottom).

400 MHz, ^{1}H NMR in $CDCl_3$

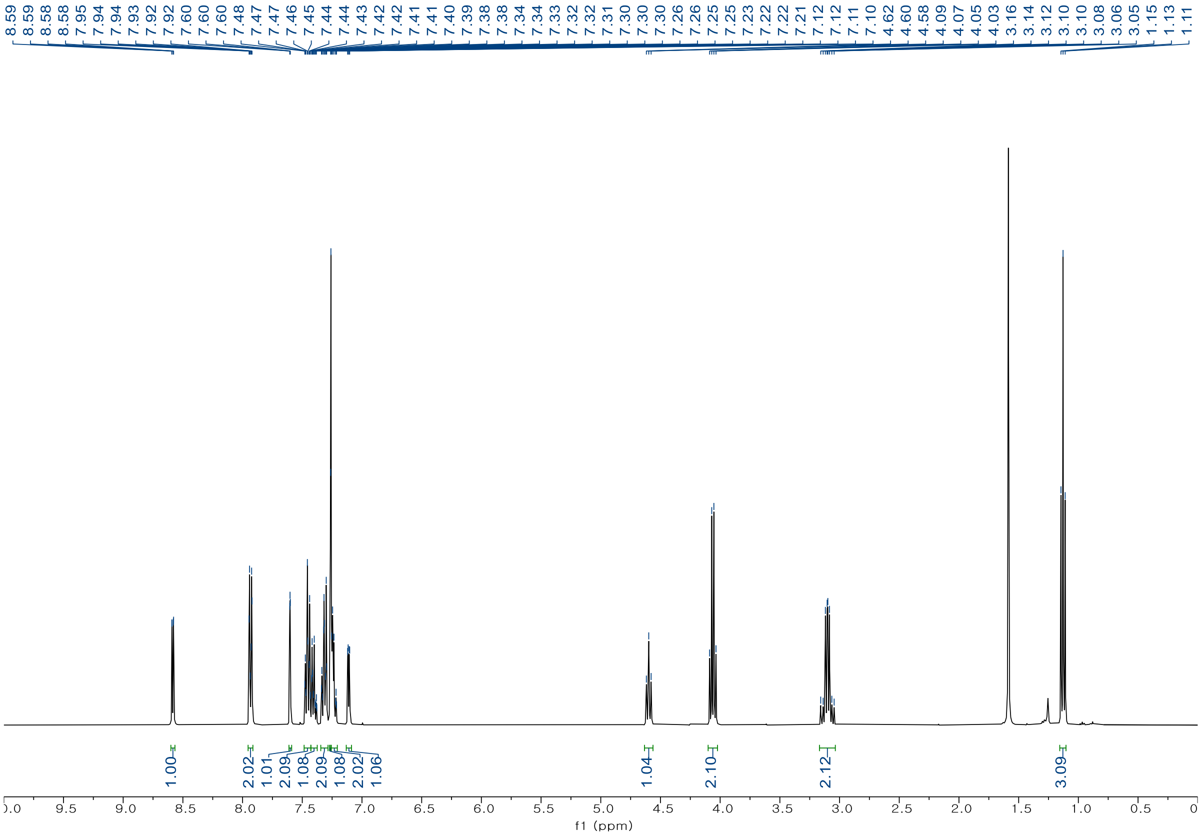

125 MHz, ^{13}C NMR in $CDCl_3$

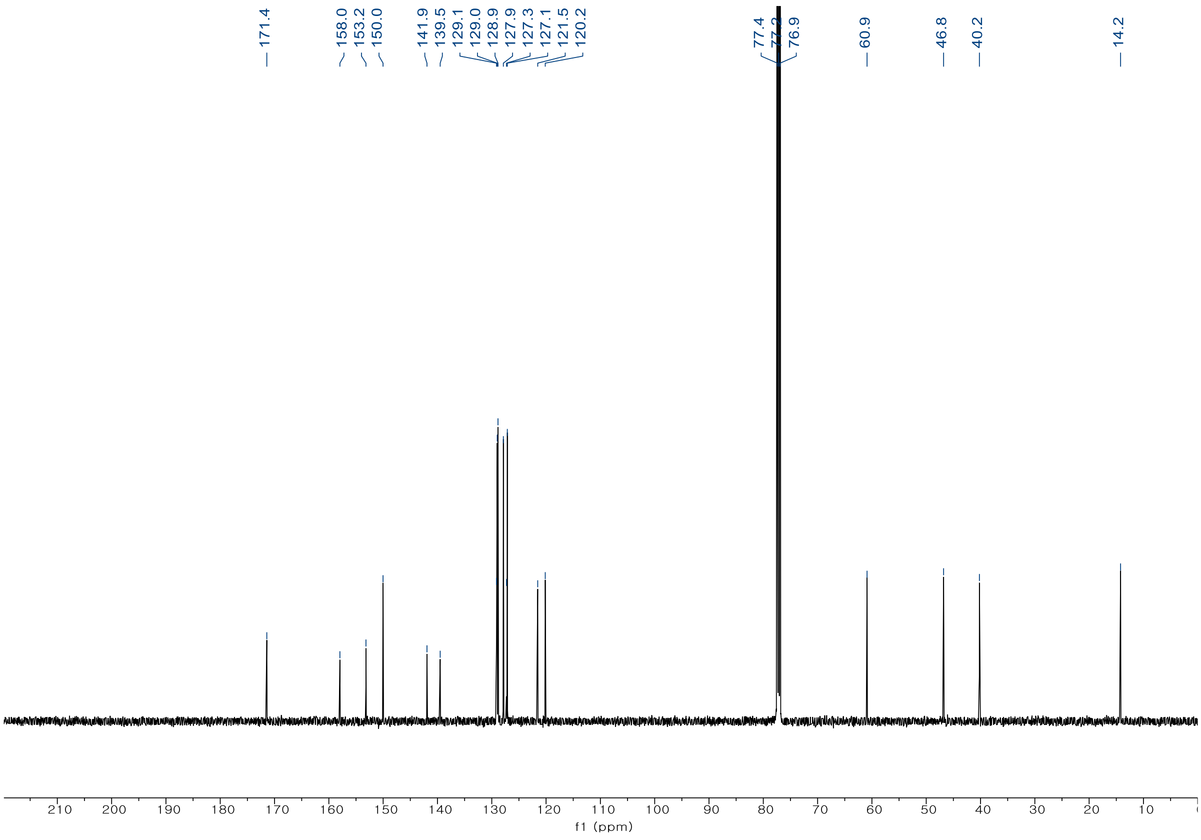

Supplementary Fig. 84. ^{1}H and ^{13}C NMR of **4l**.

Propyl (*R*)-3-phenyl-3-(2-phenylpyridin-4-yl)propanoate (4m)

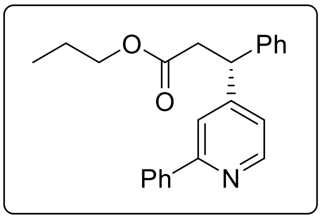

Prepared according to **GP**. Desired product **4m** was obtained as colorless oil (11.2 mg, 65% isolated yield). **^{1}H NMR** (400 MHz, $CDCl_3$) δ 8.58 (dd, *J* = 5.1, 0.7 Hz, 1H), 7.96 – 7.91 (m, 2H), 7.62 – 7.59 (m, 1H), 7.48 – 7.43 (m, 2H), 7.43 – 7.36 (m, 1H), 7.34 – 7.29 (m, 2H), 7.27 (d, *J* = 1.7 Hz, 1H), 7.25 – 7.20 (m, 2H), 7.11 (dd, *J* = 5.2, 1.7 Hz, 1H), 4.60 (t, *J* = 8.0 Hz, 1H), 3.96 (t, *J* = 6.7 Hz, 2H), 3.19 – 3.03 (m, 2H), 1.52 (h, *J* = 7.2 Hz, 2H), 0.81 (t, *J* = 7.4 Hz, 3H). **^{13}C NMR** (125 MHz, $CDCl_3$) δ 171.5, 158.0, 153.2, 150.0, 141.9, 139.5, 129.1, 129.0, 128.8, 127.8, 127.3, 127.1, 121.5, 120.2, 66.5, 46.8, 40.2, 22.0, 10.4. **HRMS** (ESI^+) m/z calcd. For $C_{23}H_{24}NO_2^+$ $[M+H]^+$: 346.1807, found 346.1806. **Specific Rotation** $[\alpha]_D^{25}$ -10.5 (*c* 0.56, $CHCl_3$). **HPLC Analysis**. CHIRALCEL OD-H, 25 °C; *n*-hexane:*i*-PrOH = 75:25, 1.0 mL/min, 254 nm, t_{R1} (major) = 6.11 min, t_{R2} (minor) = 8.62 min, 95:5 er.

The absolute stereochemistry was assigned by analogy to compound **3x** and **4k**.

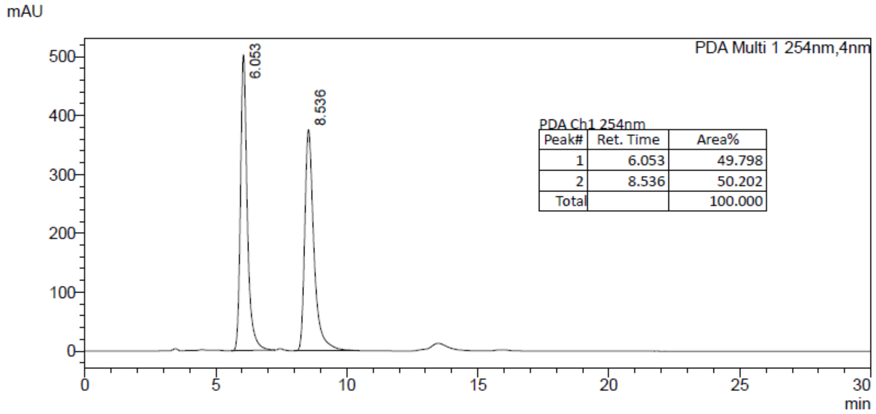

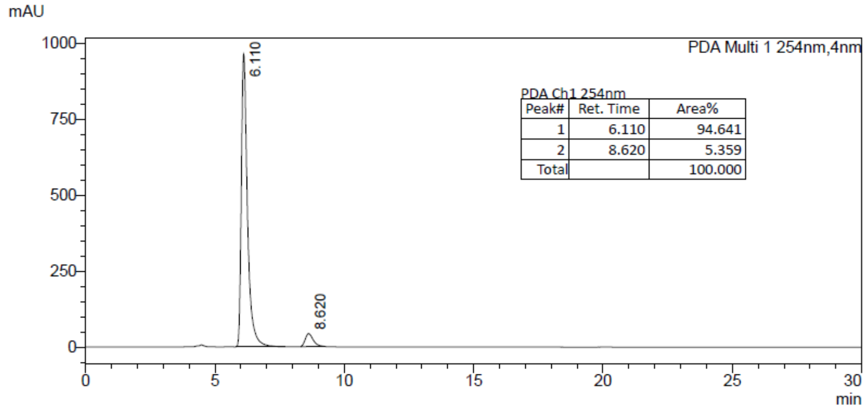

Supplementary Fig. 85. HPLC traces of *rac*-**4m** (top) and enantioenriched-**4m** (bottom).

400 MHz, ^{1}H NMR in $CDCl_3$

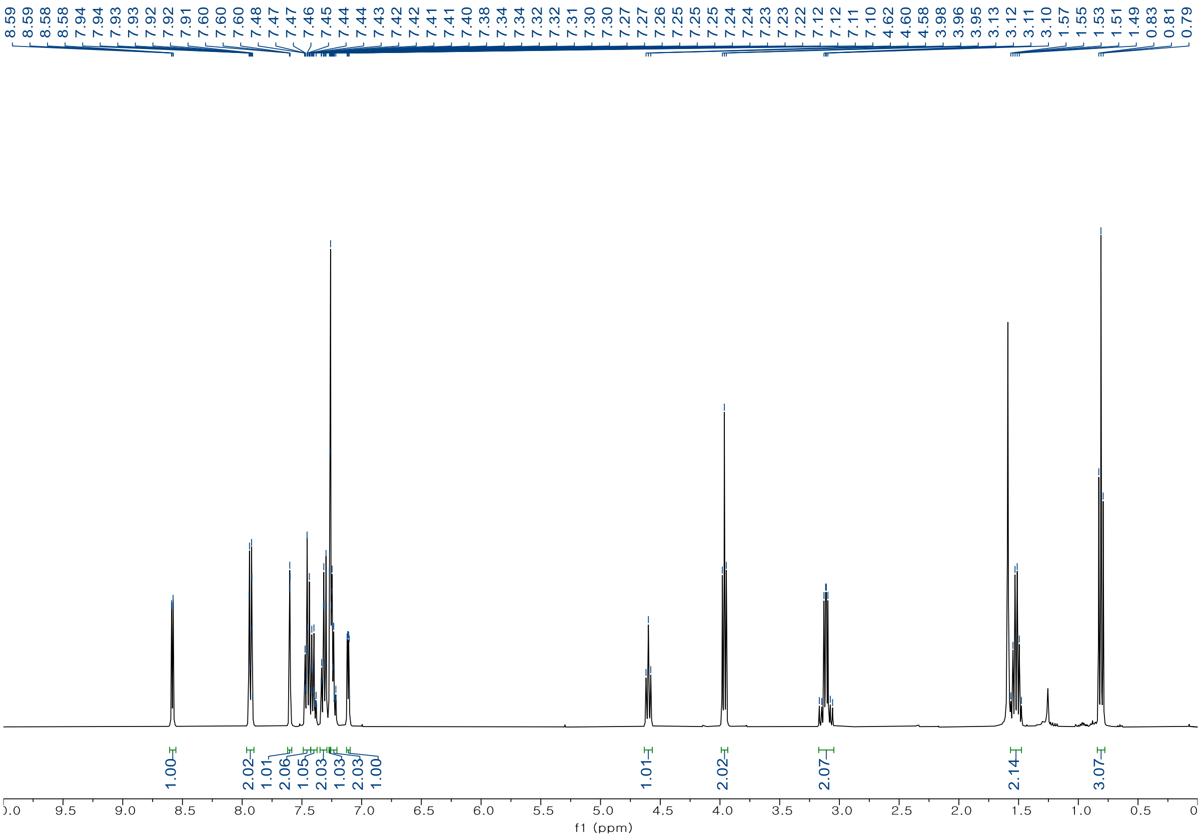

125 MHz, ^{13}C NMR in $CDCl_3$

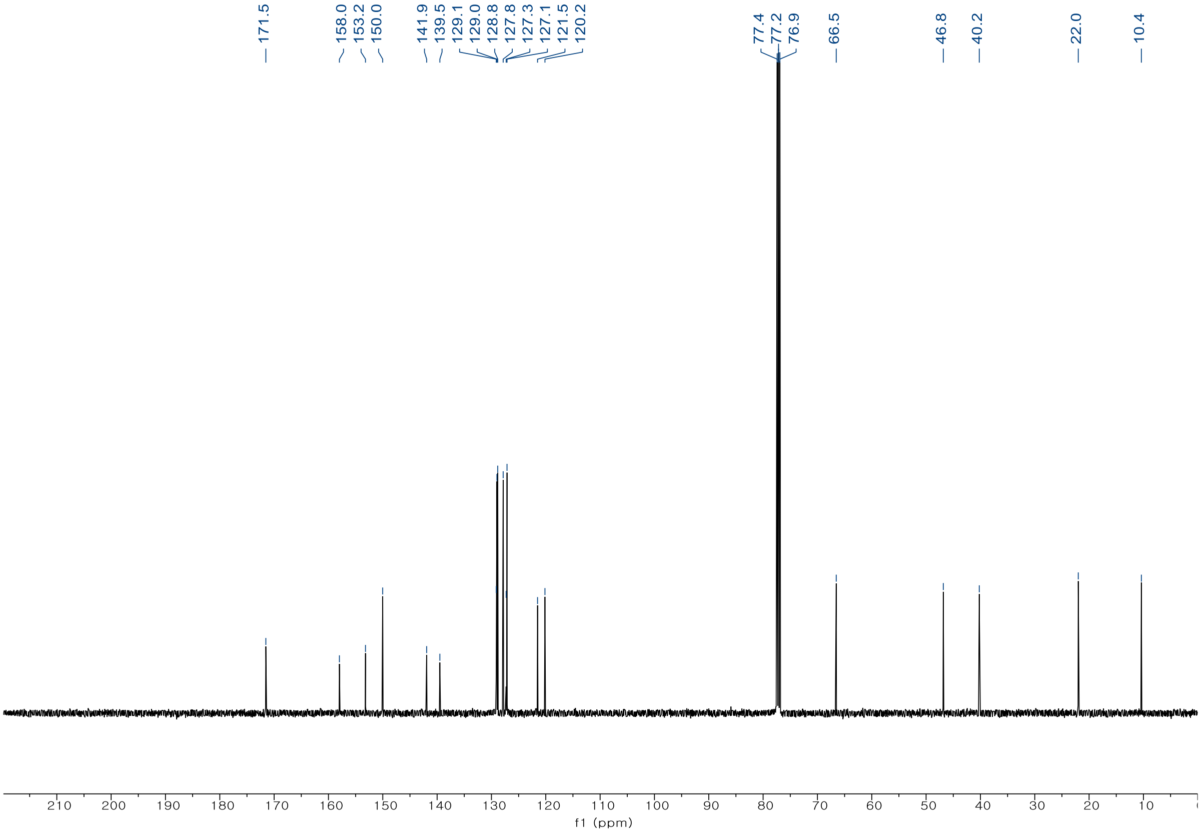

Supplementary Fig. 86. ^{1}H and ^{13}C NMR of **4m**.

Isobutyl (*R*)-3-phenyl-3-(2-phenylpyridin-4-yl)propanoate (4n)

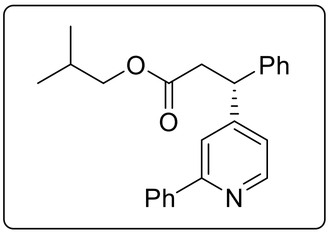

Prepared according to **GP**. Desired product **4n** was obtained as pale yellow oil (11.1 mg, 62% isolated yield). **^{1}H NMR** (400 MHz, $CDCl_3$) δ 8.61 (d, *J* = 5.1 Hz, 1H), 7.99 – 7.91 (m, 2H), 7.65 – 7.60 (m, 1H), 7.50 – 7.40 (m, 3H), 7.36 – 7.32 (m, 2H), 7.30 – 7.29 (m, 1H), 7.28 – 7.23 (m, 2H), 7.14 (dd, *J* = 5.1, 1.7 Hz, 1H), 4.62 (t, *J* = 8.0 Hz, 1H), 3.81 (d, *J* = 6.6 Hz, 2H), 3.23 – 3.07 (m, 2H), 1.82 (hept, *J* = 6.7 Hz, 1H), 0.83 (d, *J* = 6.7 Hz, 6H). **^{13}C NMR** (125 MHz, $CDCl_3$) δ 171.4, 157.9, 153.1, 149.9, 141.8, 139.4, 129.0, 128.9, 128.7, 127.7, 127.2, 127.0, 121.4, 120.0, 70.9, 46.7, 40.1, 27.6, 18.9. **HRMS** (ESI$^+$) m/z calcd. For $C_{24}H_{26}NO_2^+$ [M+H]$^+$: 360.1958, found 360.1967. **Specific Rotation** $[\alpha]_D^{25}$ +5.6 (*c* 0.54, $CHCl_3$). **HPLC Analysis**. CHIRALCEL OD-H, 25 ºC; *n*-hexane:*i*-PrOH = 75:25, 1.0 mL/min, 254 nm, t_{R1} (major) = 5.81 min, t_{R2} (minor) = 7.77 min, 94:6 er.

The absolute stereochemistry was assigned by analogy to compound **3x** and **4k**.

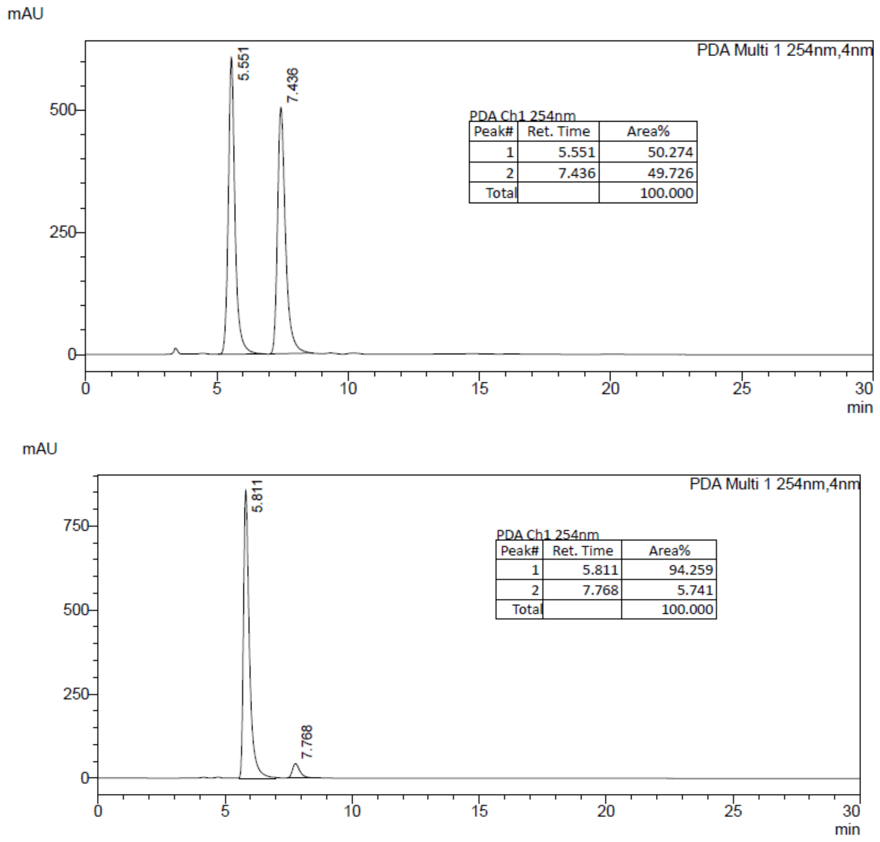

Supplementary Fig. 87. HPLC traces of *rac*-**4n** (top) and enantioenriched-**4n** (bottom).

400 MHz, ^{1}H NMR in $CDCl_3$

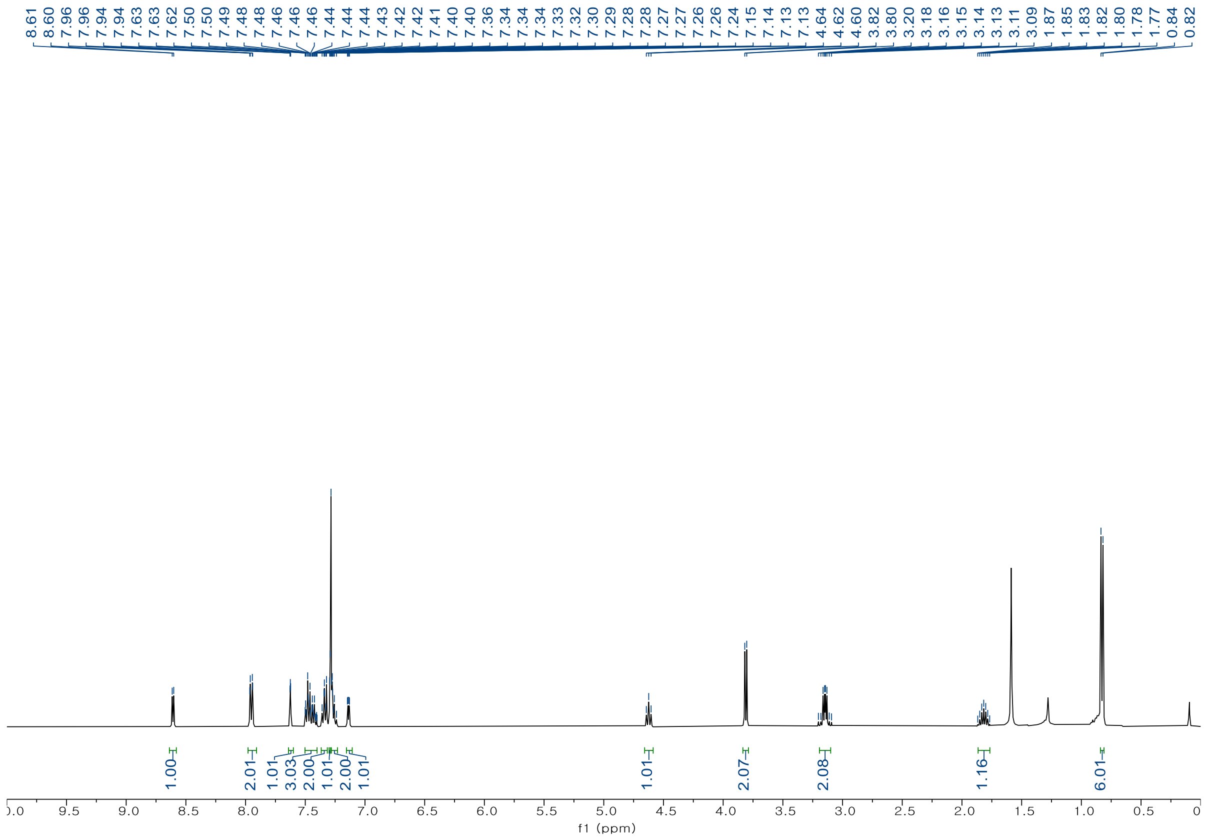

125 MHz, ^{13}C NMR in $CDCl_3$

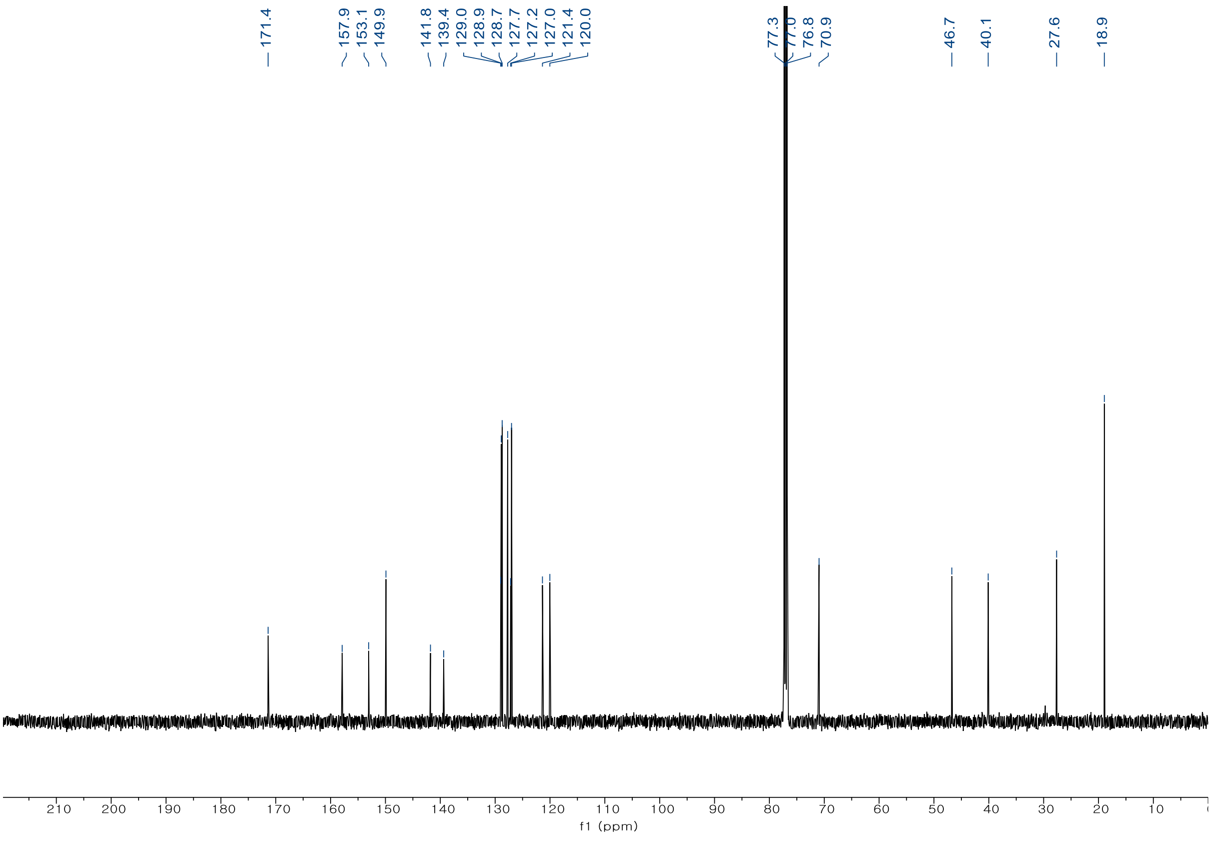

Supplementary Fig. 88. ^{1}H and ^{13}C NMR of **4n**.

Prop-2-yn-1-yl (*R*)-3-phenyl-3-(2-phenylpyridin-4-yl)propanoate (4o)

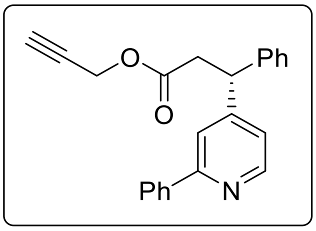

Prepared according to **GP**. Desired product **4o** was obtained as colorless oil (6.0 mg, 35% isolated yield). **^{1}H NMR** (400 MHz, CD_2Cl_2) δ 8.56 (dd, *J* = 5.1, 0.8 Hz, 1H), 8.00 – 7.92 (m, 2H), 7.64 (dt, *J* = 1.6, 0.7 Hz, 1H), 7.50 – 7.41 (m, 2H), 7.45 – 7.36 (m, 1H), 7.37 – 7.28 (m, 2H), 7.31 – 7.25 (m, 2H), 7.26 (s, 1H), 7.12 (dd, *J* = 5.2, 1.7 Hz, 1H), 4.68 – 4.54 (m, 3H), 3.26 – 3.10 (m, 2H), 2.43 (t, *J* = 2.5 Hz, 1H). **^{13}C NMR** (125 MHz, CD_2Cl_2) δ 170.4, 157.5, 152.8, 149.9, 141.8, 139.3, 128.9, 128.8, 128.6, 127.7, 127.1, 126.9, 121.4, 119.7, 77.4, 74.6, 52.1, 46.5, 39.4. **HRMS** (EI^+) m/z calcd. For $C_{23}H_{19}NO_2^+$ $[M]^+$: 341.1416, found 341.1412. **Specific Rotation** $[\alpha]_D^{25}$ +22.0 (*c* 0.1, $CHCl_3$). **HPLC Analysis**. CHIRALPAK IB, 25 °C; *n*-hexane:*i*-PrOH = 85:15, 1.0 mL/min, 254 nm, t_{R1} (major) = 13.02 min, t_{R2} (minor) = 24.34 min, 94:6 er.

The absolute stereochemistry was assigned by analogy to compound **3x** and **4k**.

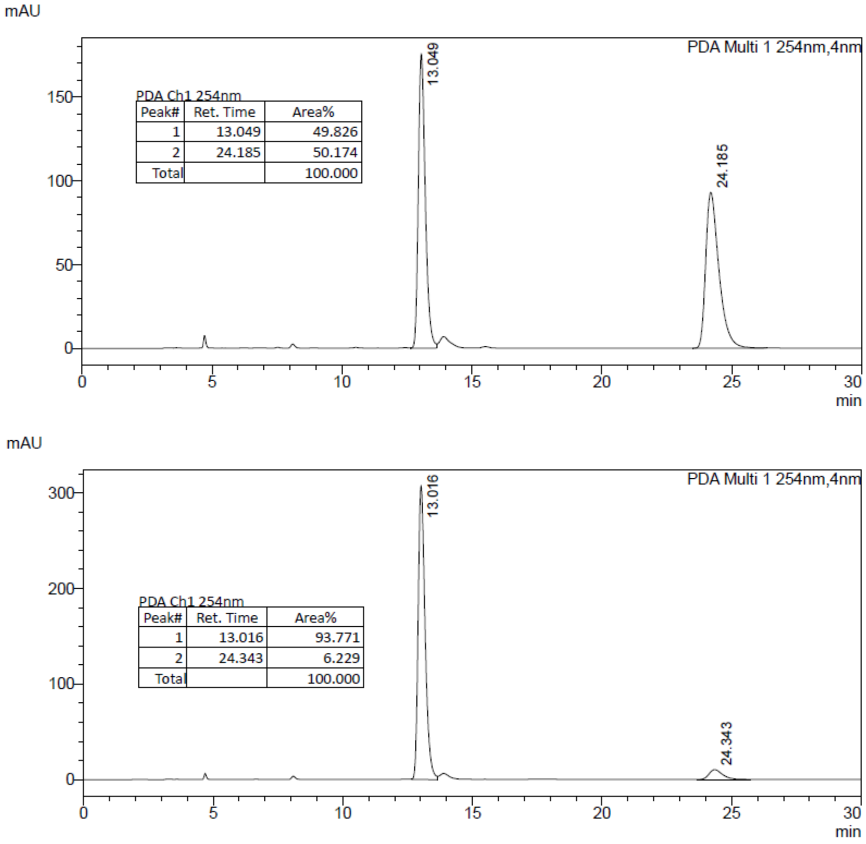

Supplementary Fig. 89. HPLC traces of *rac*-**4o** (top) and enantioenriched-**4o** (bottom).

400 MHz, ^{1}H NMR in CD_2Cl_2

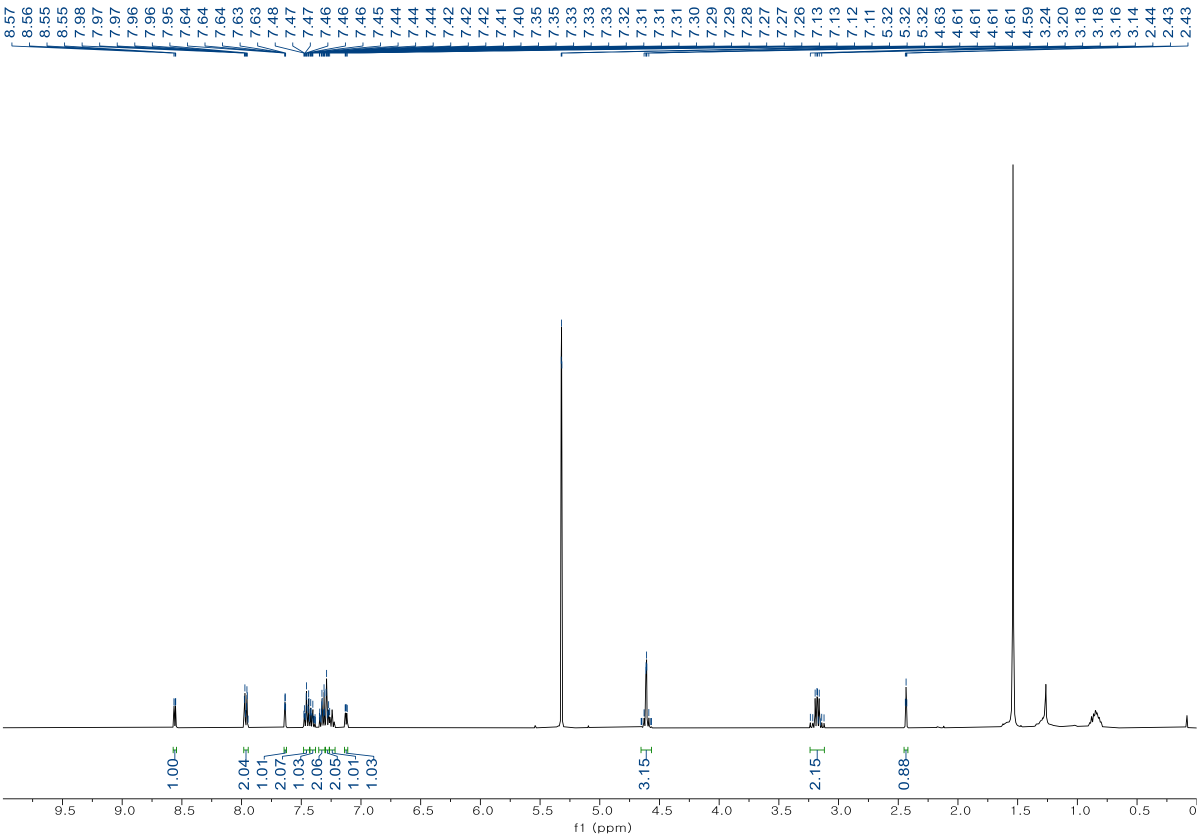

125 MHz, ^{13}C NMR in CD_2Cl_2

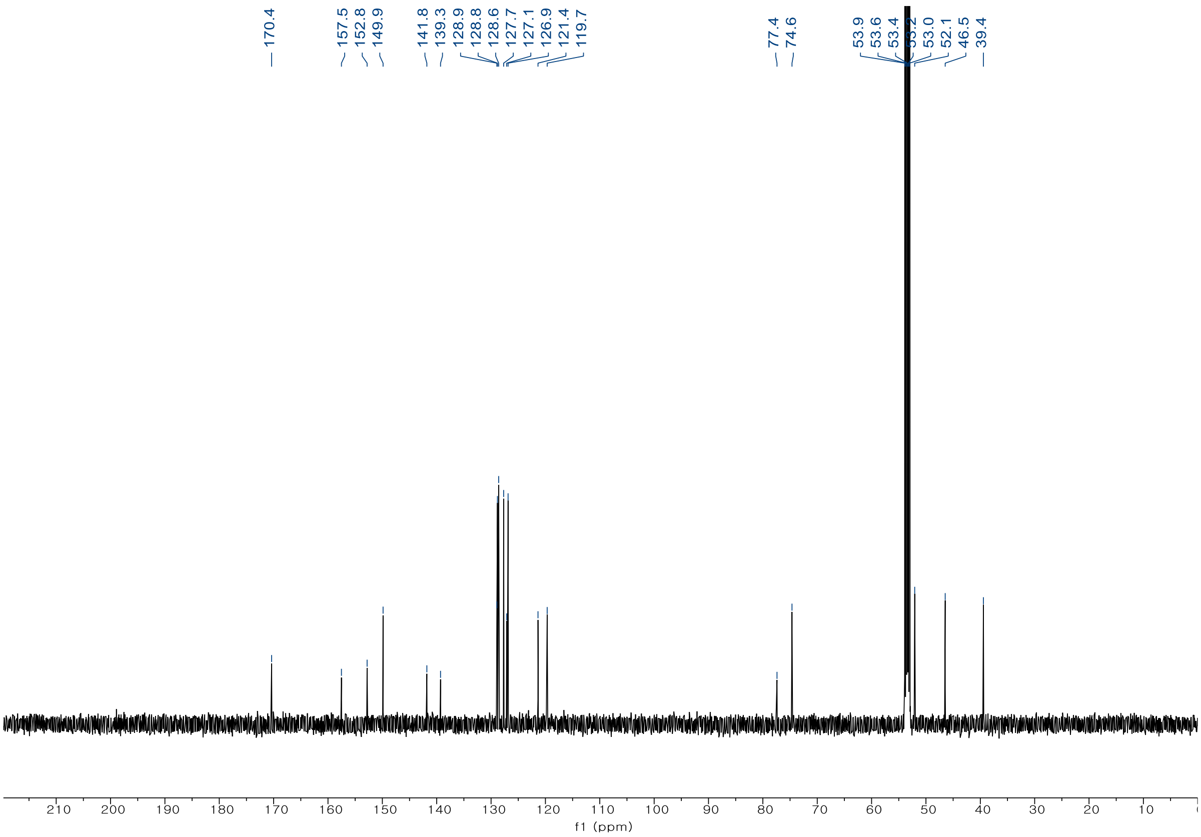

Supplementary Fig. 90. ^{1}H and ^{13}C NMR of **4o**.

Benzyl (*S*)-3-phenyl-3-(2-phenylpyridin-4-yl)propanoate (4p)

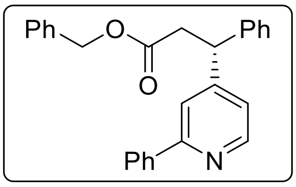

Prepared according to **GP**. Desired product **4p** was obtained as coloreless oil (10.1 mg, 61% isolated yield). **^{1}H NMR** (400 MHz, $CDCl_3$) δ 8.56 (dd, *J* = 5.1, 0.8 Hz, 1H), 7.95 – 7.87 (m, 2H), 7.58 (dt, *J* = 1.6, 0.7 Hz, 1H), 7.48 – 7.43 (m, 2H), 7.45 – 7.36 (m, 1H), 7.34 – 7.26 (m, 5H), 7.26 – 7.21 (m, 3H), 7.19 – 7.13 (m, 2H), 7.10 – 7.07 (m, 1H), 5.04 (s, 2H), 4.66 – 4.57 (m, 1H), 3.25 – 3.09 (m, 2H). **^{13}C NMR** (100 MHz, $CDCl_3$) δ 171.2, 158.0, 153.0, 150.0, 141.8, 139.4, 135.6, 129.1, 129.0, 128.8, 128.7, 128.4, 128.3, 127.8, 127.3, 127.1, 121.5, 120.1, 66.8, 46.8, 40.2. **HRMS** (ESI^+) m/z calcd. For $C_{27}H_{24}NO_2^+$ $[M+H]^+$: 394.1807, found 394.1805. **Specific Rotation** $[\alpha]_D^{26}$ –9.4 (*c* 1.0, $CHCl_3$). **HPLC Analysis**. CHIRALCEL OD-H, 25 °C; *n*-hexane:*i*-PrOH = 65:35, 1.0 mL/min, 254 nm, t_{R1} (major) = 9.28 min, t_{R2} (minor) = 38.64 min, 95:5 er.

The absolute stereochemistry was assigned by analogy to compound **3x** and **4k**.

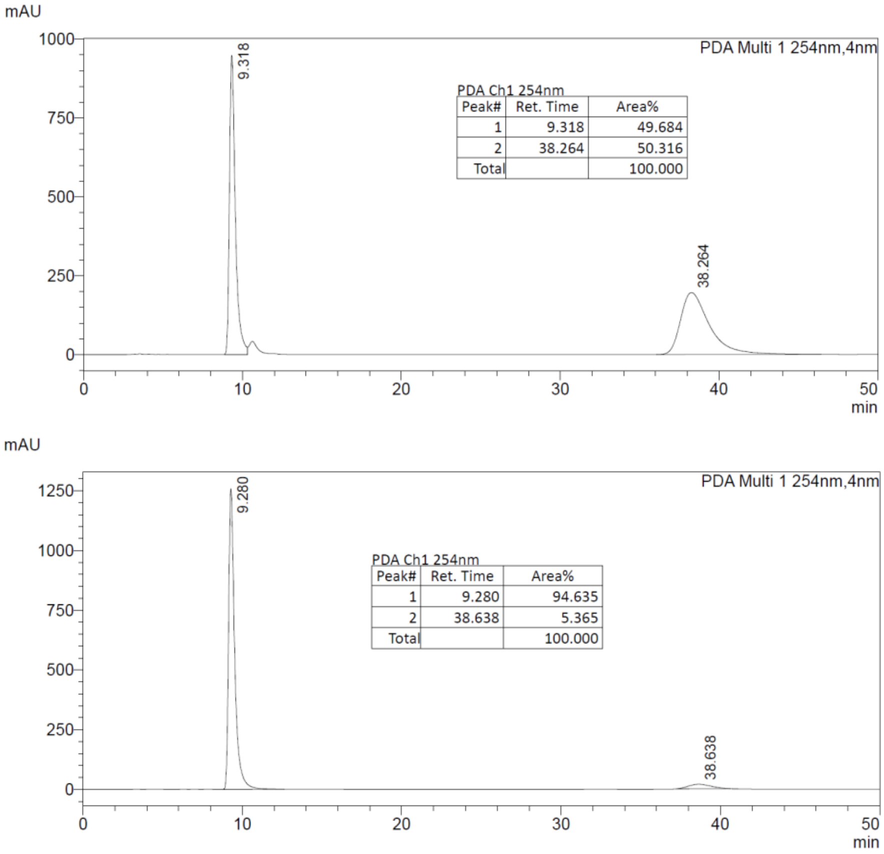

Supplementary Fig. 91. HPLC traces of *rac*-**4p** (top) and enantioenriched-**4p** (bottom).

400 MHz, ^{1}H NMR in $CDCl_3$.

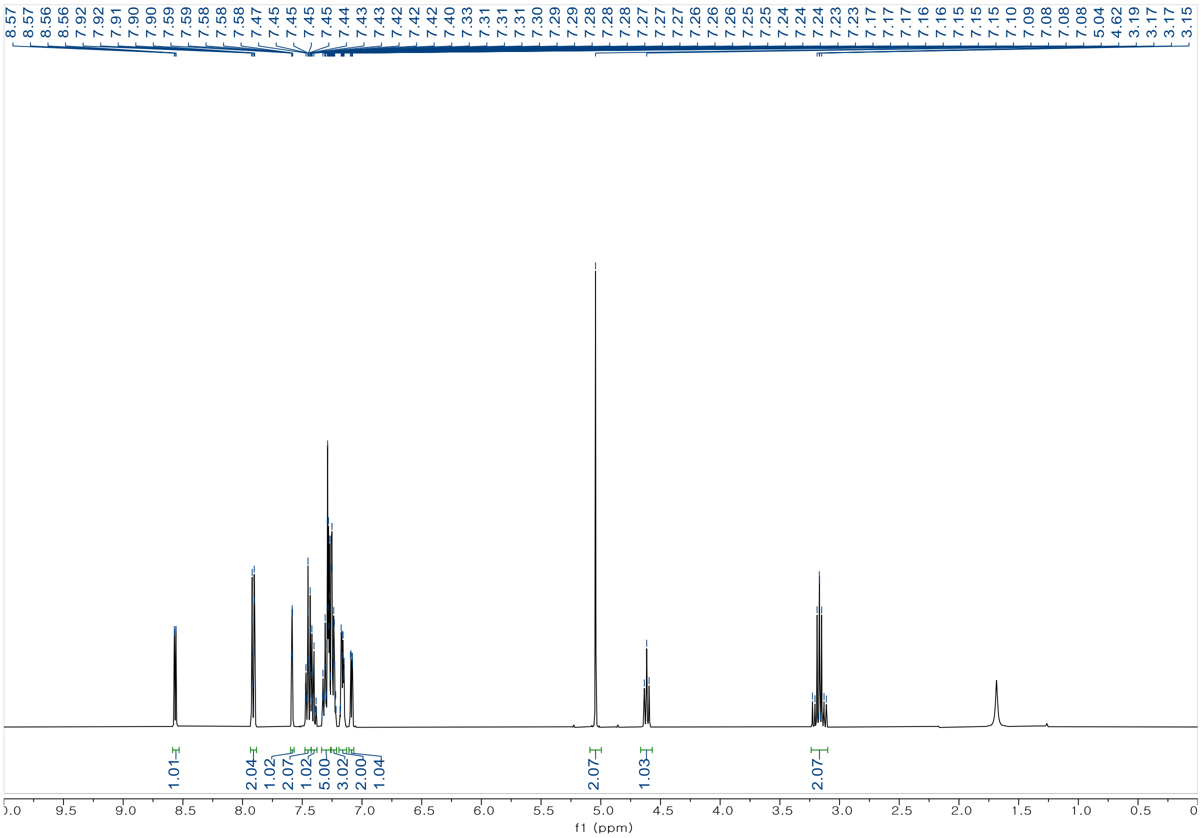

100 MHz, ^{13}C NMR in $CDCl_3$

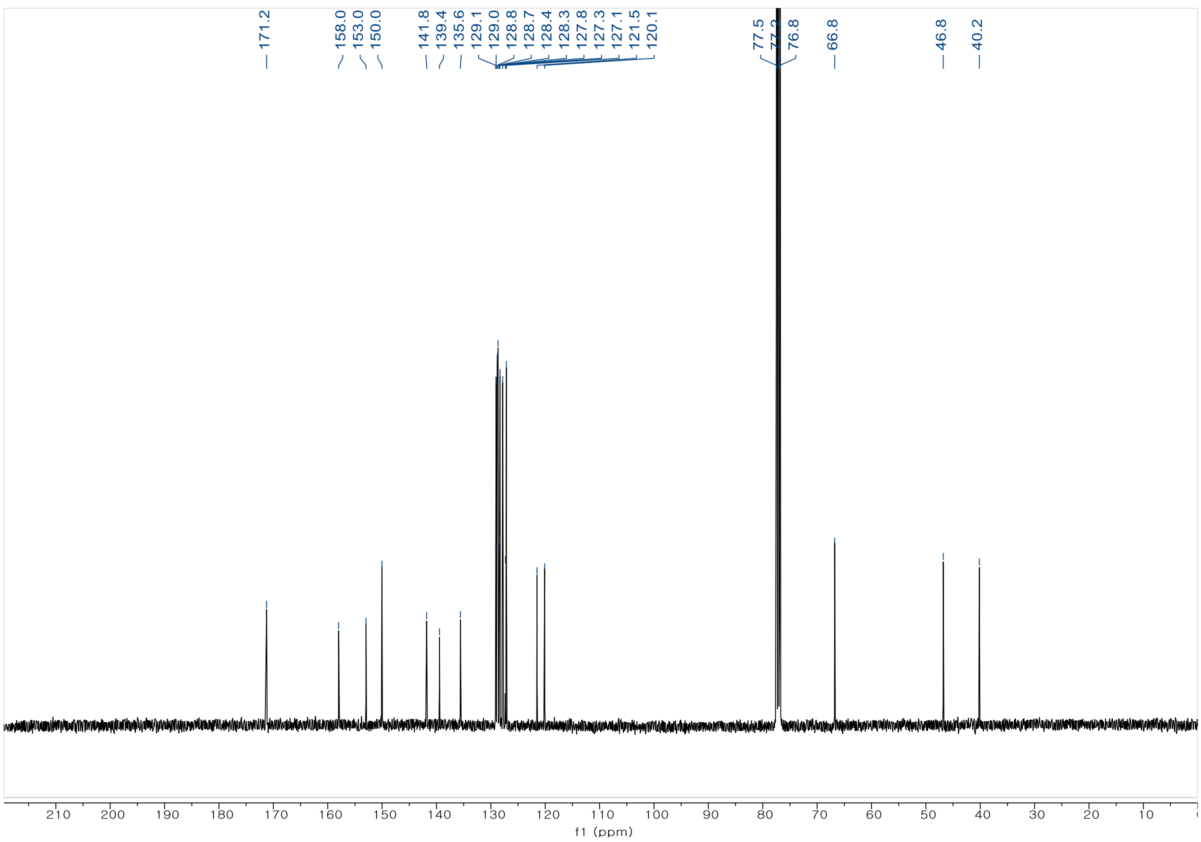

Supplementary Fig. 92. ^{1}H and ^{13}C NMR of **4p**.

Allyl (*R*)-3-phenyl-3-(2-phenylpyridin-4-yl)propanoate (4q)

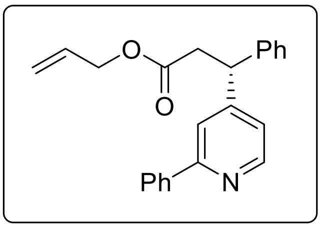

Prepared according to **GP**. Desired product **4q** was obtained as pale yellow oil (9.0 mg, 53% isolated yield). **^{1}H NMR** (400 MHz, $CDCl_3$) δ 8.59 (d, J = 5.1 Hz, 1H), 7.98 – 7.87 (m, 2H), 7.62 – 7.58 (m, 1H), 7.48 – 7.38 (m, 3H), 7.35 – 7.29 (m, 2H), 7.27 – 7.21 (m, 3H), 7.11 (dd, J = 5.2, 1.7 Hz, 1H), 5.77 (ddt, J = 17.3, 10.4, 5.8 Hz, 1H), 5.23 – 5.10 (m, 2H), 4.61 (t, J = 7.9 Hz, 1H), 4.51 (dt, J = 5.8, 1.4 Hz, 2H), 3.23 – 3.06 (m, 2H). **^{13}C NMR** (125 MHz, $CDCl_3$) δ 171.1, 158.0, 153.1, 150.1, 141.9, 139.5, 131.9, 129.1, 129.0, 128.9, 127.9, 127.3, 127.2, 121.5, 120.2, 118.7, 65.6, 46.8, 40.1. **HRMS** (ESI^+) m/z calcd. For $C_{23}H_{22}NO_2^+$ $[M+H]^+$: 344.1651, found 344.1651. **Specific Rotation** $[\alpha]_D^{25}$ -8.9 (c 0.61, $CHCl_3$). **HPLC Analysis**. CHIRALCEL OD-H, 25 ºC; n-hexane:i-PrOH = 75:25, 1.0 mL/min, 254 nm, t_{R1} (major) = 7.09 min, t_{R2} (minor) = 12.85 min, 94:6 er.

The absolute stereochemistry was assigned by analogy to compound **3x** and **4k**.

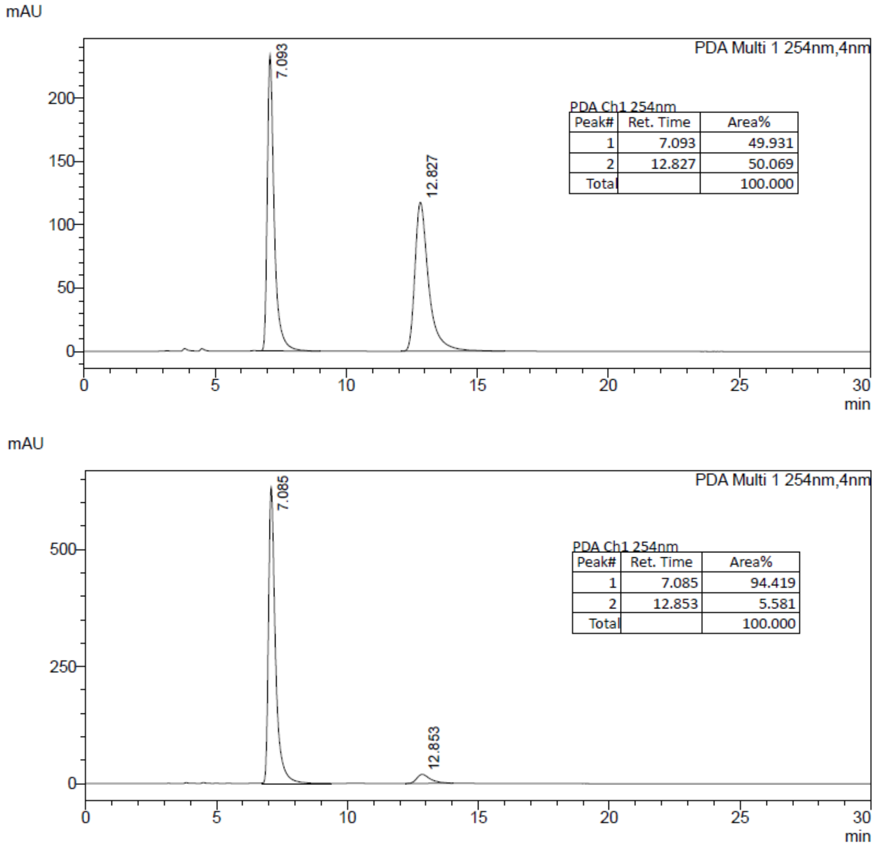

Supplementary Fig. 93. HPLC traces of *rac*-**4q** (top) and enantioenriched-**4q** (bottom).

400 MHz, ^{1}H NMR in $CDCl_3$

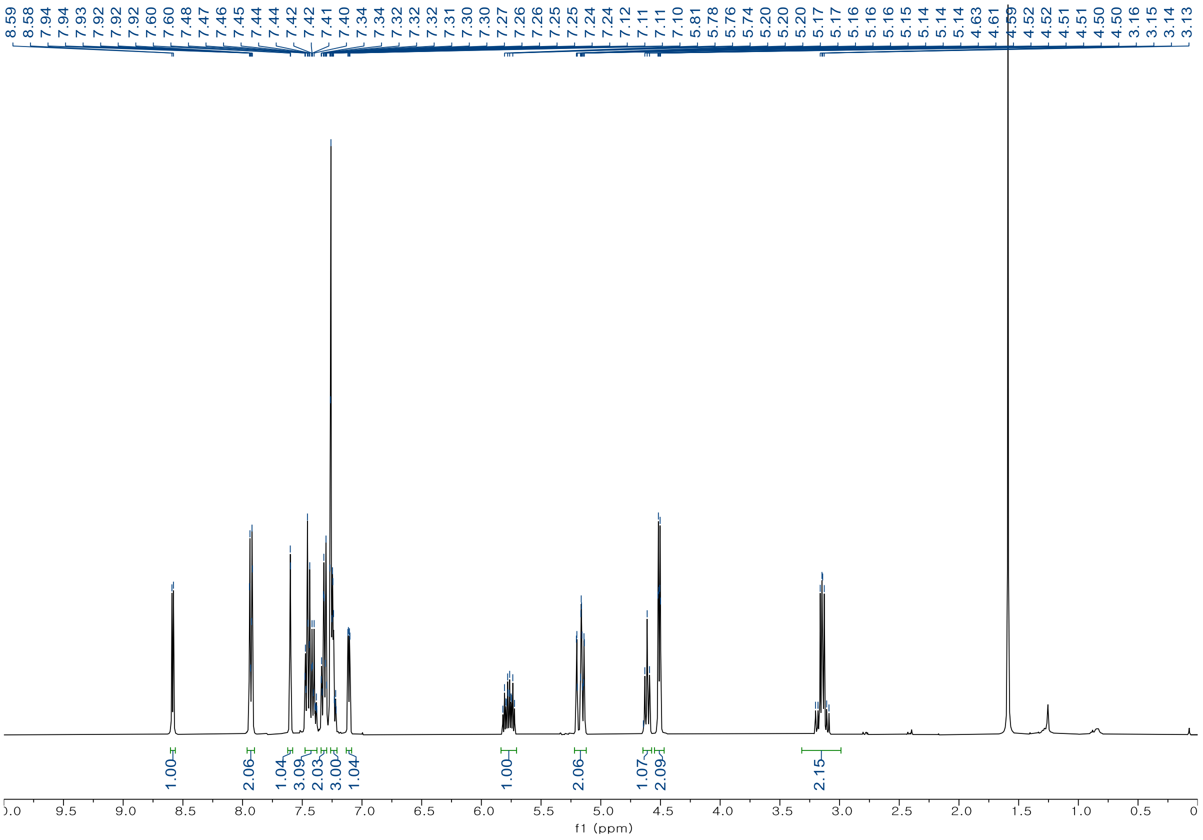

125 MHz, ^{13}C NMR in $CDCl_3$

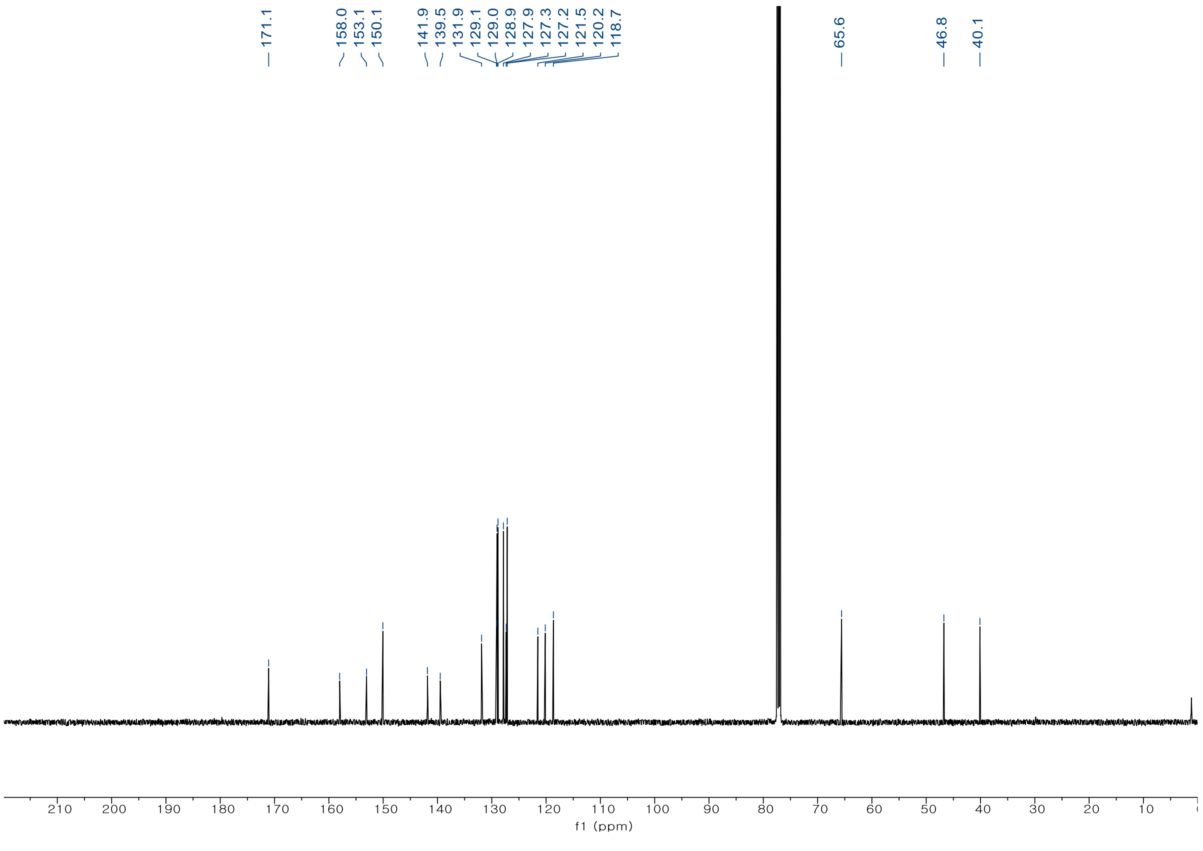

Supplementary Fig. 94. ^{1}H and ^{13}C NMR of **4q**.

Cyclopropylmethyl (*R*)-3-phenyl-3-(2-phenylpyridin-4-yl)propanoate (4r)

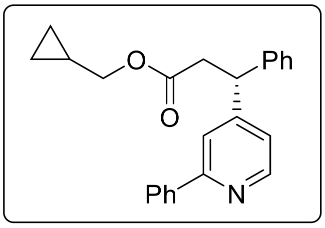

Prepared according to **GP**. Desired product **4r** was obtained as colorless oil (7.1 mg, 40% isolated yield). **^{1}H NMR** (500 MHz, $CDCl_3$) δ 8.58 (d, *J* = 5.1 Hz, 1H), 7.97 – 7.88 (m, 2H), 7.61 (d, *J* = 1.6 Hz, 1H), 7.49 – 7.43 (m, 2H), 7.42 – 7.37 (m, 1H), 7.34 – 7.29 (m, 2H), 7.28 – 7.27 (m, 1H), 7.26 – 7.21 (m, 2H), 7.12 (dd, *J* = 5.2, 1.7 Hz, 1H), 4.61 (t, *J* = 8.0 Hz, 1H), 3.83 (d, *J* = 7.3, 1.2 Hz, 2H), 3.20 – 3.06 (m, 2H), 1.04 – 0.92 (m, 1H), 0.49 – 0.40 (m, 2H), 0.18 – 0.11 (m, 2H). **^{13}C NMR** (100 MHz, $CDCl_3$) δ 171.6, 158.0, 153.1, 150.0, 142.0, 139.5, 129.1, 129.0, 128.9, 127.9, 127.3, 127.1, 121.6, 120.2, 69.7, 46.9, 40.3, 9.8, 3.3, 3.3. **HRMS** (EI^+) m/z calcd. For $C_{24}H_{23}NO_2^+$ $[M]^+$: 357.1729, found 357.1725. **Specific Rotation** $[\alpha]_D^{25}$ -6.8 (*c* 0.30, $CHCl_3$). **HPLC Analysis**. CHIRALCEL OD-H, 25 ºC; *n*-hexane:*i*-PrOH = 75:25, 1.0 mL/min, 254 nm, t_{R1} (major) = 6.57 min, t_{R2} (minor) = 11.44 min, 94:6 er. The absolute stereochemistry was assigned by analogy to compound **3x** and **4k**.

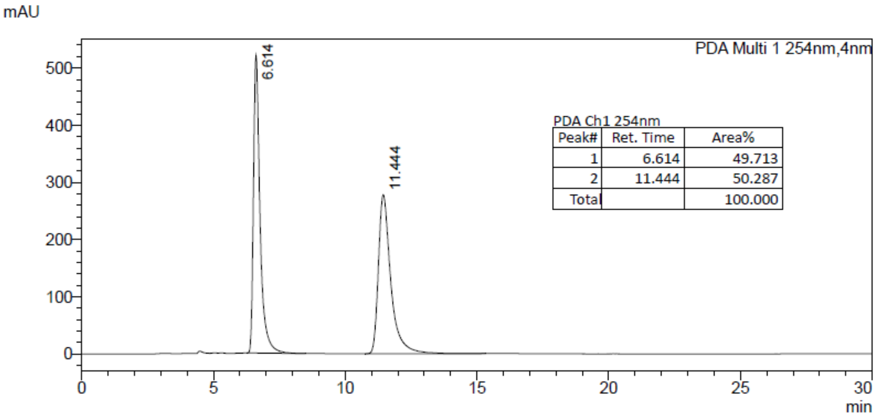

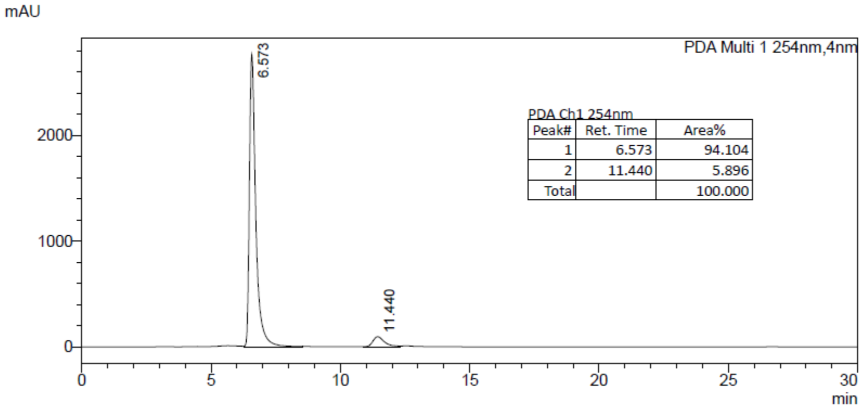

Supplementary Fig. 95. HPLC traces of *rac*-**4r** (top) and enantioenriched-**4r** (bottom).

500 MHz, ^{1}H NMR in $CDCl_3$

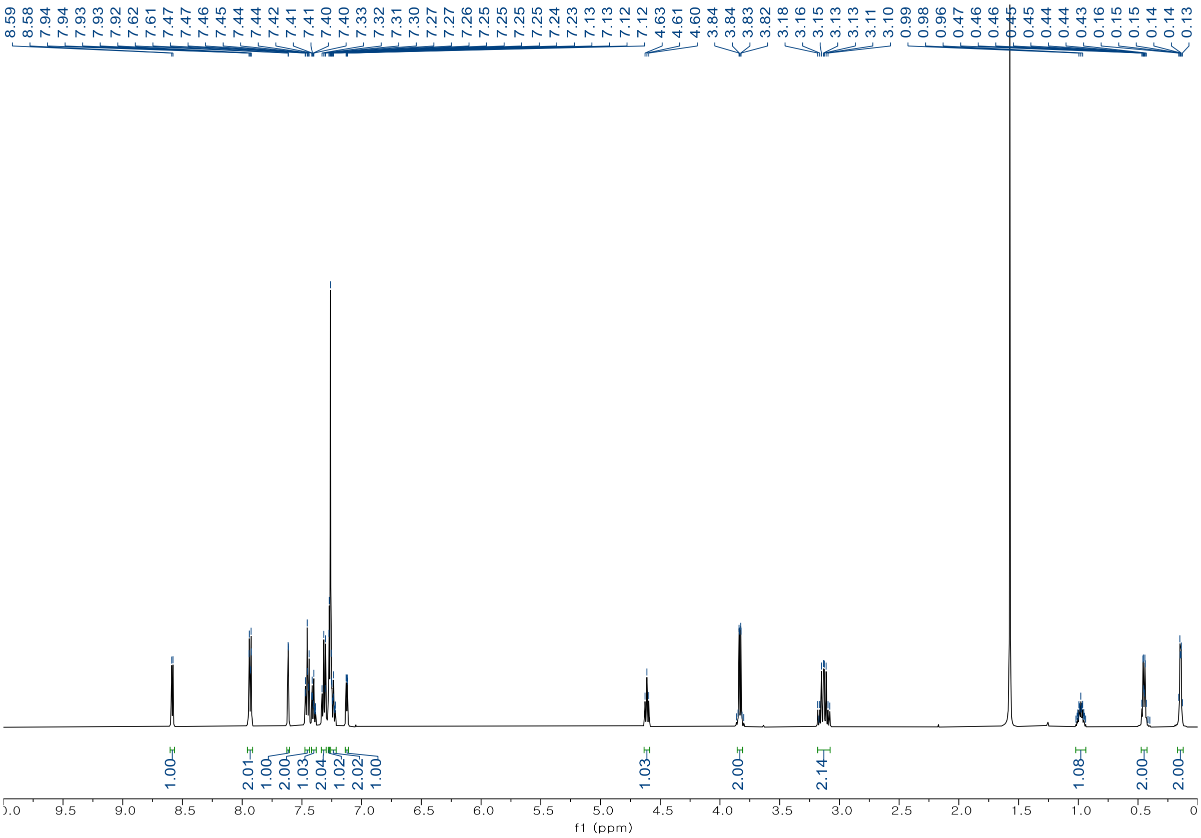

100 MHz, ^{13}C NMR in $CDCl_3$

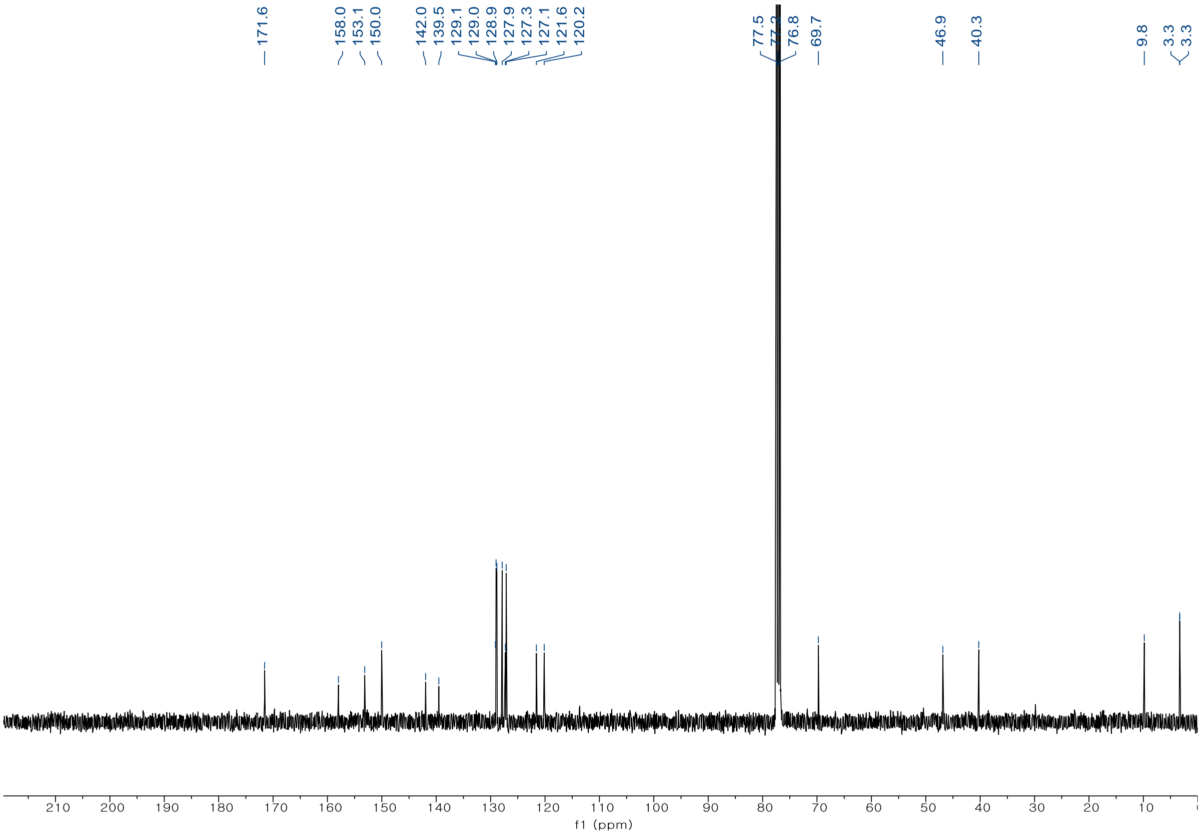

Supplementary Fig. 96. ^{1}H and ^{13}C NMR of **4r**.

Cyclohexylmethyl (*R*)-3-phenyl-3-(2-phenylpyridin-4-yl)propanoate (4s)

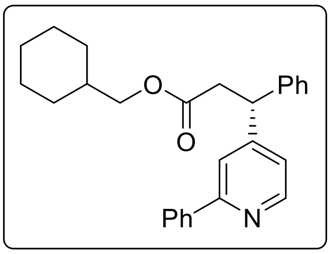

Prepared according to **GP**. Desired product **4s** was obtained as white solid (13.0 mg, 65% isolated yield). **^{1}H NMR** (400 MHz, $CDCl_3$) δ 8.58 (d, *J* = 5.1 Hz, 1H), 7.95 – 7.90 (m, 2H), 7.63 – 7.58 (m, 1H), 7.48 – 7.37 (m, 3H), 7.35 – 7.28 (m, 2H), 7.28 – 7.26 (m, 1H), 7.25 – 7.21 (m, 2H), 7.11 (dd, *J* = 5.2, 1.8 Hz, 1H), 4.59 (t, *J* = 8.0 Hz, 1H), 3.81 (d, *J* = 6.4 Hz, 2H), 3.19 – 3.03 (m, 2H), 1.68 – 1.59 (m, 1H), 1.56 – 1.40 (m, 3H), 1.21 – 0.75 (m, 7H). **^{13}C NMR** (100 MHz, $CDCl_3$) δ 171.5, 158.0, 153.1, 150.0, 141.9, 139.5, 129.1, 129.0, 128.8, 127.8, 127.3, 127.1, 121.5, 120.1, 70.1, 46.9, 40.3, 37.1, 29.6, 26.3, 25.7. **HRMS** (ESI^+) m/z calcd. For $C_{27}H_{30}NO_2^+$ $[M+H]^+$: 400.2277, found 400.2277. **Specific Rotation** $[\alpha]_D^{25}$ -10.5 (*c* 0.36, $CHCl_3$). **HPLC Analysis**. CHIRALCEL OD-H, 25 °C; *n*-hexane:*i*-PrOH = 75:25, 1.0 mL/min, 254 nm, t_{R1} (major) = 6.23 min, t_{R2} (minor) = 10.06 min, 93:7 er.

The absolute stereochemistry was assigned by analogy to compound **3x** and **4k**.

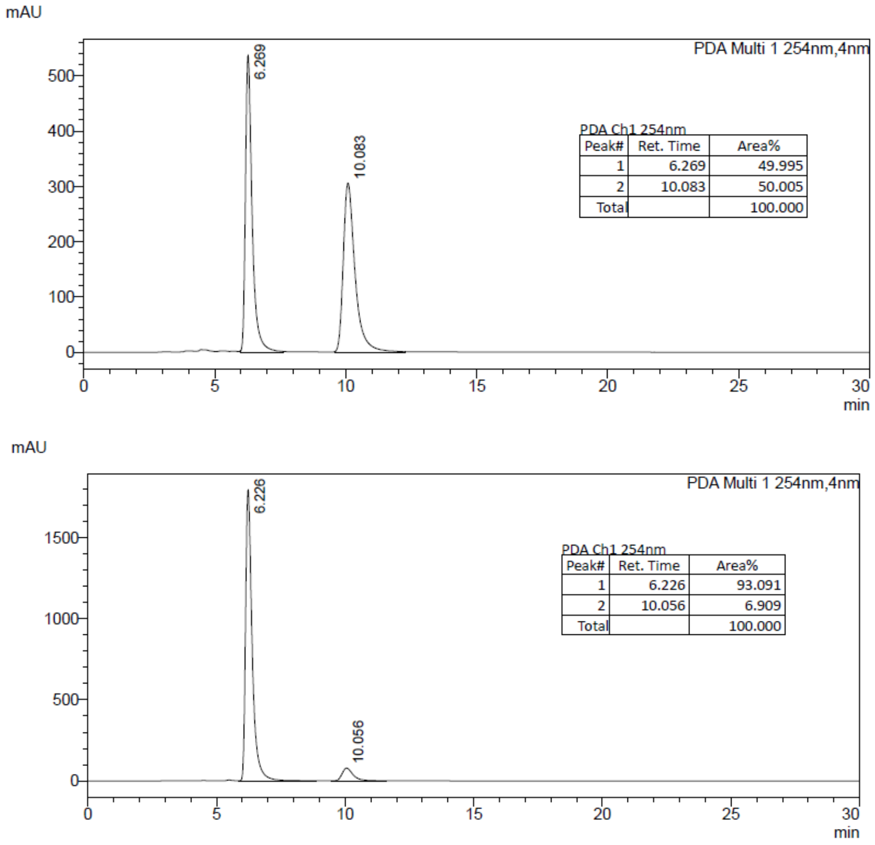

Supplementary Fig. 97. HPLC traces of *rac*-**4s** (top) and enantioenriched-**4s** (bottom).

400 MHz, ^{1}H NMR in $CDCl_3$

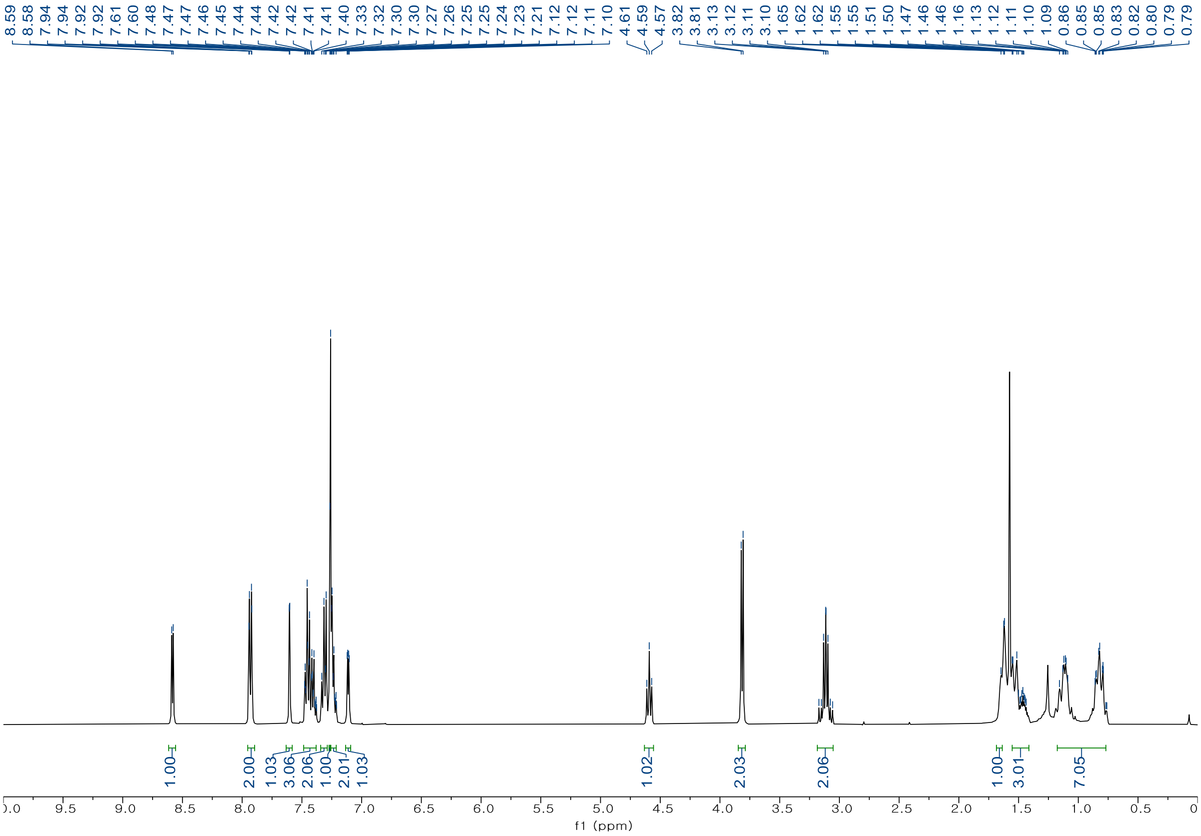

100 MHz, ^{13}C NMR in $CDCl_3$

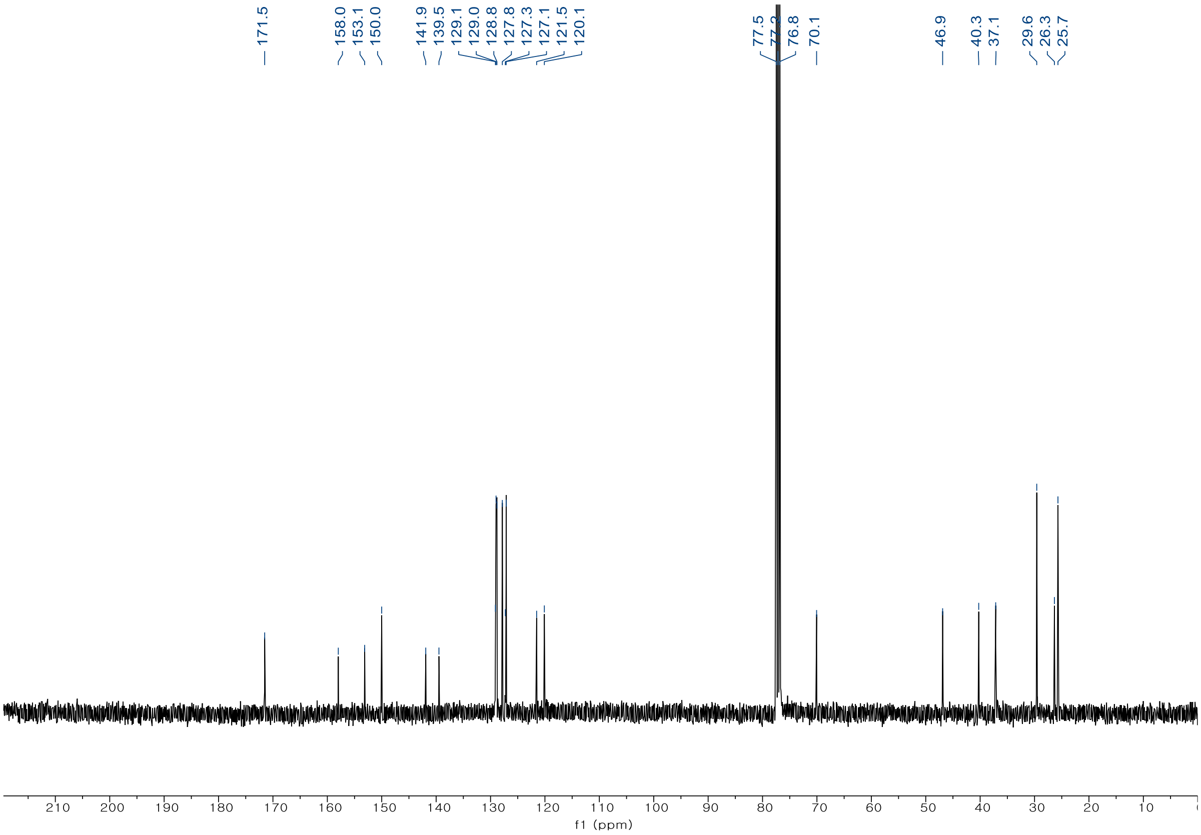

Supplementary Fig. 98. ^{1}H and ^{13}C NMR of **4s**.

(*R*)-3-Phenyl-3-(2-phenylpyridin-4-yl)-1-(1*H*-pyrazol-1-yl)propan-1-one (4t)

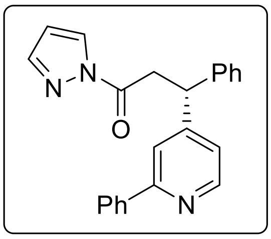

Prepared according to **GP**. Desired product **4t** was obtained as white solid (10.3 mg, 58% isolated yield). **^{1}H NMR** (500 MHz, $CDCl_3$) δ 8.60 (dd, *J* = 5.0, 0.8 Hz, 1H), 8.21 (dd, *J* = 2.8, 0.7 Hz, 1H), 7.97 – 7.91 (m, 2H), 7.78 – 7.74 (m, 1H), 7.71 – 7.67 (m, 1H), 7.49 – 7.45 (m, 2H), 7.45 – 7.39 (m, 1H), 7.37 – 7.32 (m, 4H), 7.28 – 7.23 (m, 1H), 7.20 (dd, *J* = 5.2, 1.8 Hz, 1H), 6.46 (dd, *J* = 2.9, 1.5 Hz, 1H), 4.87 (t, *J* = 7.7 Hz, 1H), 4.09 (dd, *J* = 17.1, 8.1 Hz, 1H), 3.95 (dd, *J* = 17.1, 7.3 Hz, 1H). **^{13}C NMR** (125 MHz, $CDCl_3$) δ 169.9, 158.0, 153.0, 150.1, 144.4, 141.9, 139.5, 129.1, 129.1, 128.8, 128.6, 128.0, 127.4, 127.2, 121.6, 120.2, 110.1, 46.1, 39.3. **HRMS** (ESI^+) m/z calcd. For $[C_{23}H_{20}N_3O]^+$: 354.1606, found 354.1606. **Specific Rotation** $[\alpha]_D^{25}$ +4.7 (*c* 0.72, $CHCl_3$). **HPLC Analysis**. CHIRALCEL OD-H, 25 °C; *n*-hexane:*i*-PrOH = 95:5, 1.0 mL/min, 254 nm, t_{R1} (major) = 23.57 min, t_{R2} (minor) = 28.09 min, 95:5 er. The absolute stereochemistry was assigned by analogy to compound **3x** and **4k**.

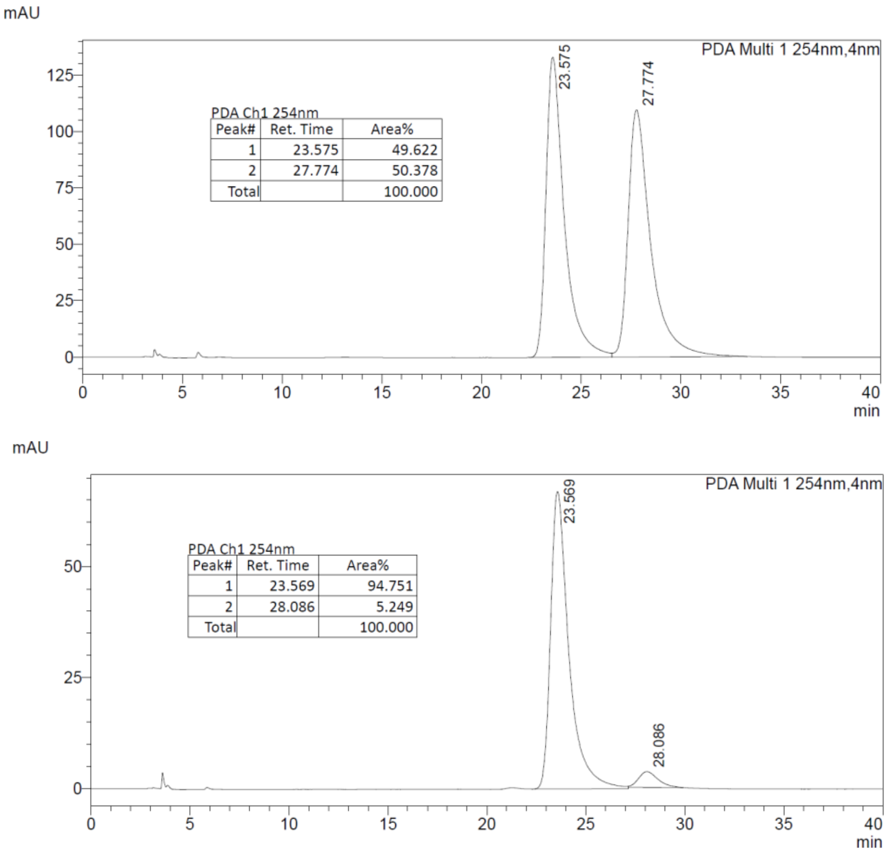

Supplementary Fig. 99. HPLC traces of *rac*-**4t** (top) and enantioenriched-**4t** (bottom).

500 MHz, ^{1}H NMR in $CDCl_3$.

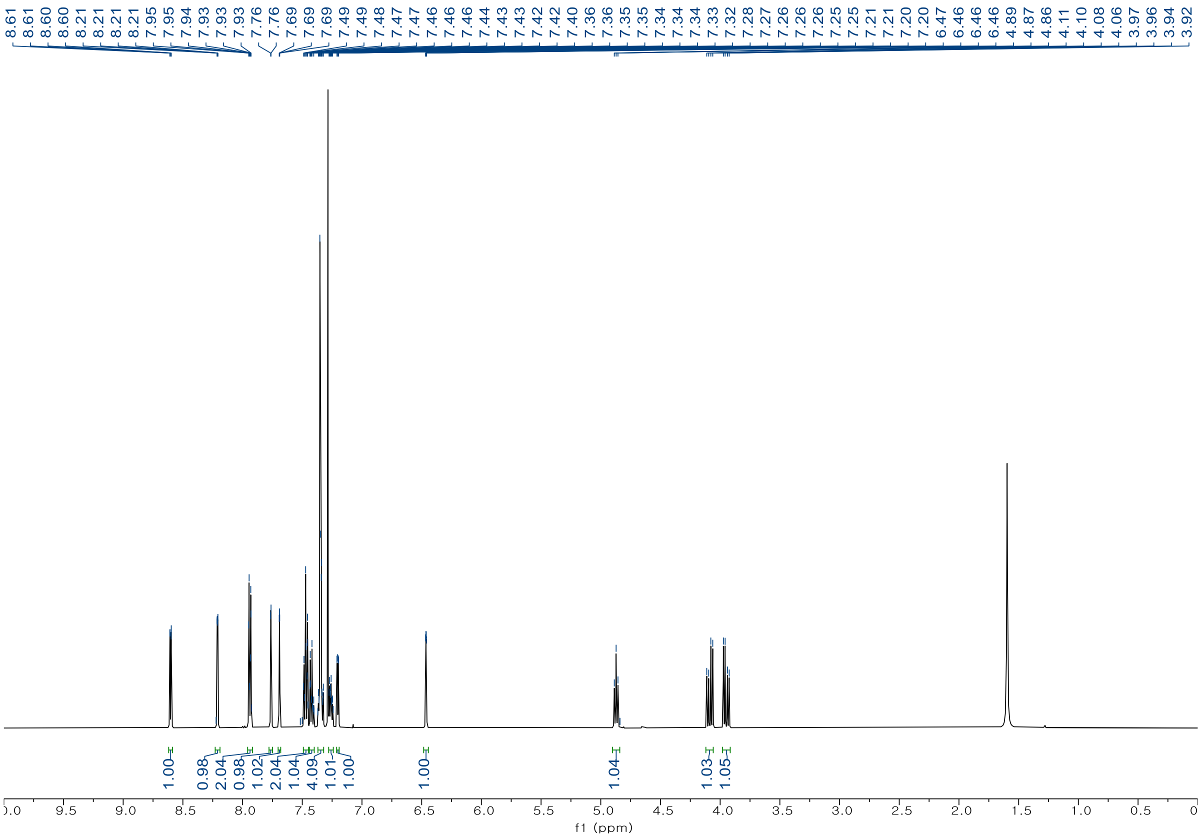

125 MHz, ^{13}C NMR in $CDCl_3$

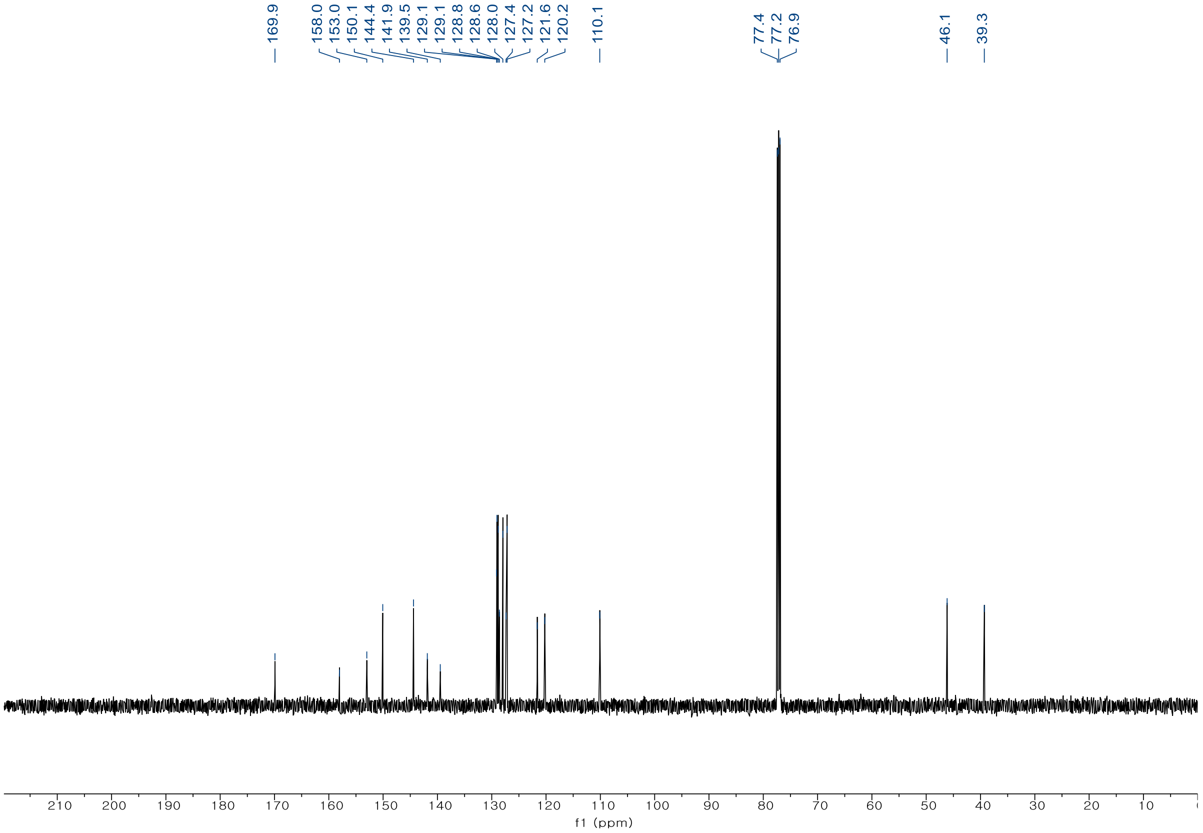

Supplementary Fig. 100. ^{1}H and ^{13}C NMR of **4t**.

Methyl (*R*)-3-((8*R*,9*S*,13*S*,14*S*)-13-methyl-17-oxo-7,8,9,11,12,13,14,15,16,17-decahydro-6*H*-cyclopenta[*a*]phenanthren-3-yl)-3-(2-phenylpyridin-4-yl)propanoate (6a)

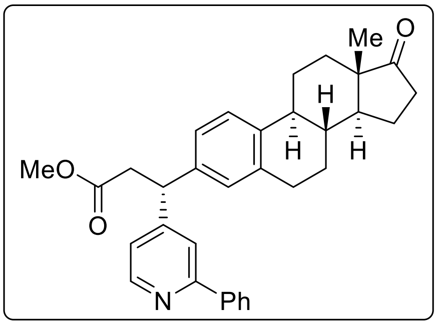

Prepared according to **GP**. Desired product **6a** was obtained as white solid (14.6 mg, 59% isolated yield). **^{1}H NMR** (400 MHz, $CDCl_3$) δ 8.62 – 8.55 (m, 1H), 7.98 – 7.90 (m, 2H), 7.63 – 7.57 (m, 1H), 7.49 – 7.43 (m, 2H), 7.43 – 7.37 (m, 1H), 7.25 – 7.20 (m, 1H), 7.12 (dd, J = 5.1, 1.7 Hz, 1H), 7.04 (dd, J = 8.1, 2.1 Hz, 1H), 6.96 – 6.92 (m, 1H), 4.58 – 4.49 (m, 1H), 3.62 (s, 3H), 3.18 – 3.02 (m, 2H), 2.86 (dd, J = 9.0, 4.2 Hz, 2H), 2.49 (dd, J = 18.8, 8.7 Hz, 1H), 2.43 – 2.35 (m, 1H), 2.26 (td, J = 10.7, 4.4 Hz, 1H), 2.19 – 1.90 (m, 4H), 1.62 – 1.36 (m, 6H), 0.89 (s, 3H). **^{13}C NMR** (100 MHz, $CDCl_3$) δ 221.0, 172.0, 157.9, 153.3, 150.0, 139.5, 139.4, 138.8, 137.2, 129.1, 128.8, 128.4, 127.1, 126.0, 125.0, 121.5, 120.2, 52.0, 50.6, 48.1, 46.3, 44.4, 39.9, 38.2, 36.0, 31.7, 29.6, 26.6, 25.7, 21.7, 14.0. **HRMS** (EI^+) m/z calcd. For $C_{33}H_{35}NO_3^+$ $[M]^+$: 493.2617, found 493.2615. **Specific Rotation** $[\alpha]_D^{26}$ +82.8 (c 1.0, $CHCl_3$). **HPLC Analysis**. CHIRALCEL OD-H, 25 ºC; *n*-hexane:*i*-PrOH = 75:25, 1.0 mL/min, 254 nm, t_{R1} (major diastereomer) = 17.98 min, t_{R2} (minor diastereomer) = 41.86 min, 98:2 dr.

The absolute stereochemistry was assigned by analogy to compound **3x** and **4k**.

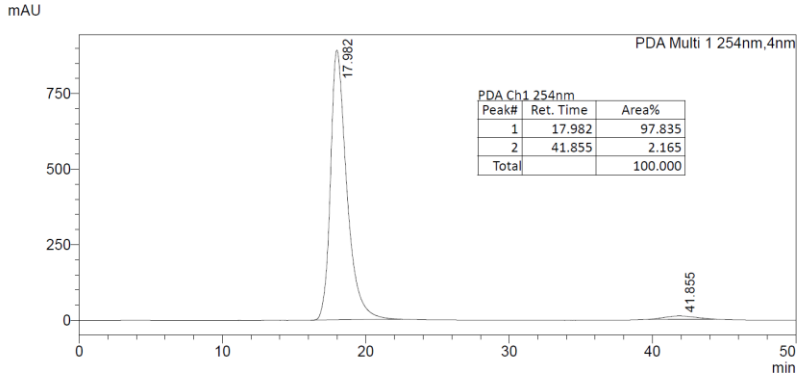

Supplementary Fig. 101. HPLC traces of diastereomeric mixture of **6a** using NHC **5f**.

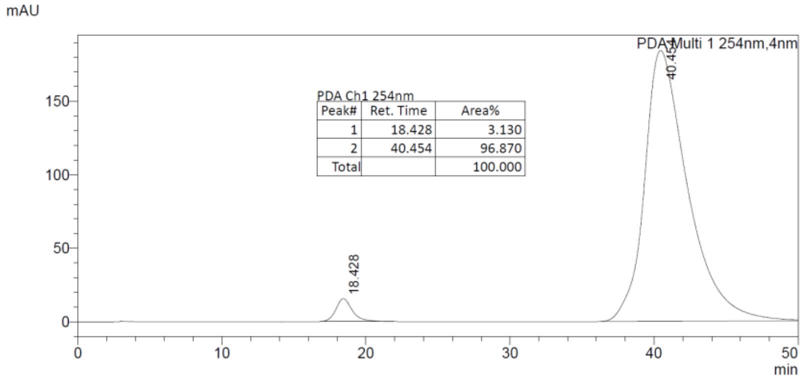

Supplementary Fig. 102. HPLC traces of diastereomeric mixture of **6a** using NHC **5f*** (3:97 dr).

400 MHz, ^{1}H NMR in $CDCl_3$.

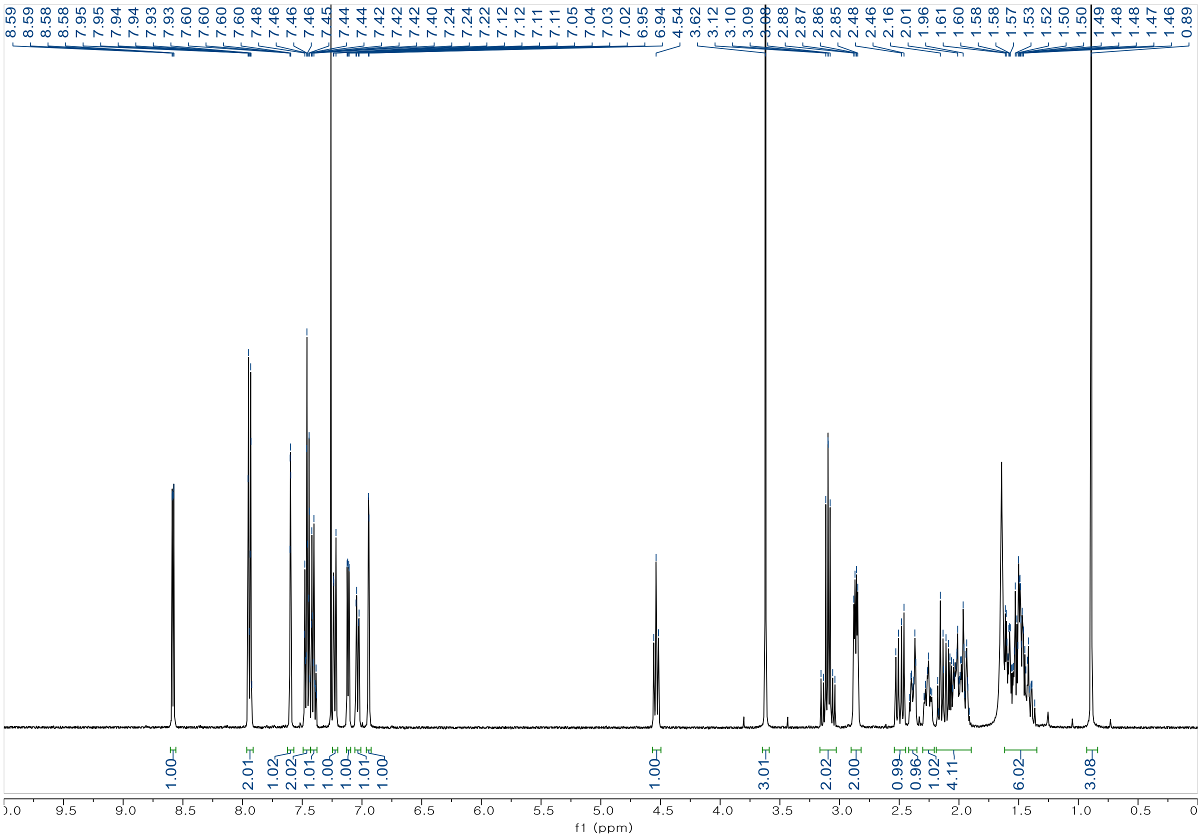

100 MHz, ^{13}C NMR in $CDCl_3$.

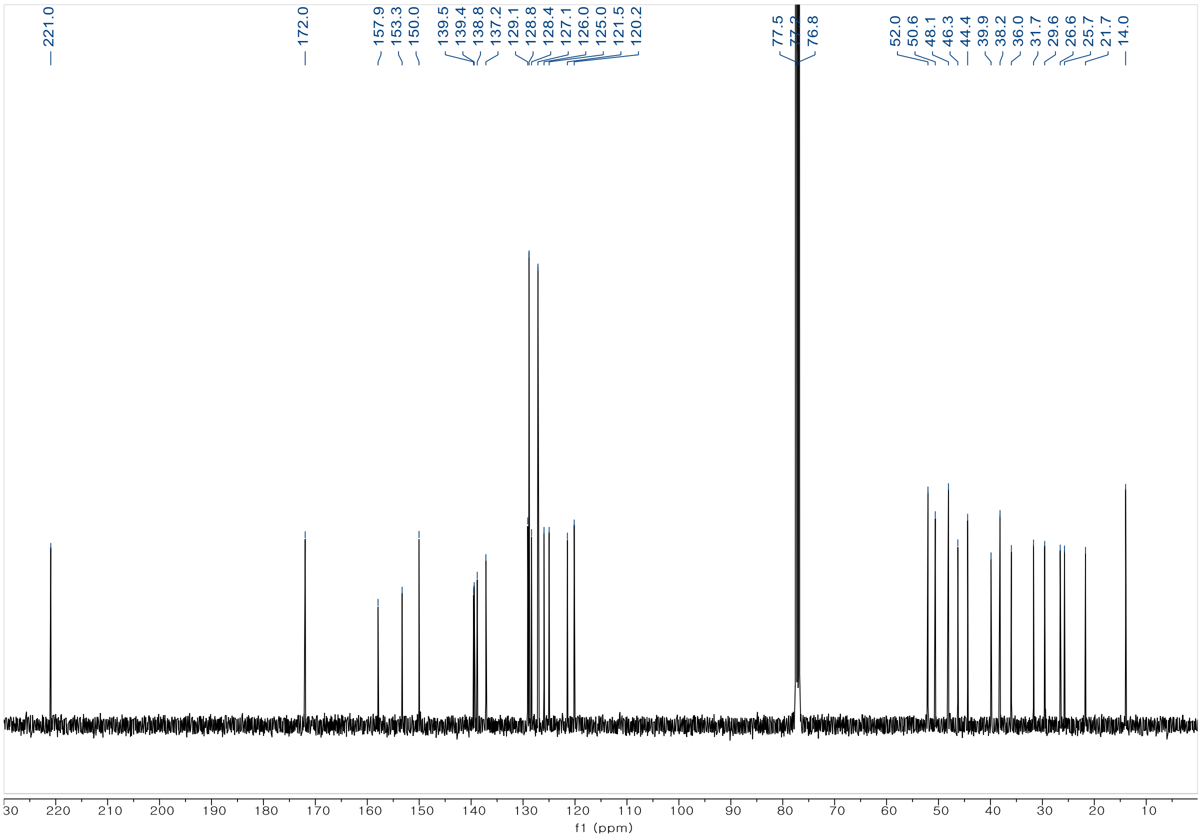

Supplementary Fig. 103. ^{1}H and ^{13}C NMR of **6a**.

Methyl (*R*)-2-((tert-butoxycarbonyl)amino)-3-(4-((*S*)-3-methoxy-3-oxo-1-(2-phenylpyridin-4-yl) propyl)phenyl)propanoate (6b)

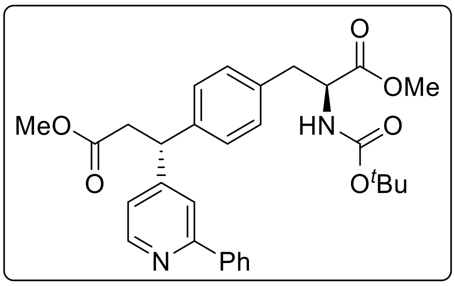

Prepared according to **GP**. Desired product **6b** was obtained as white solid (15.6 mg, 60% isolated yield). **^{1}H NMR** (400 MHz, $CDCl_3$) δ 8.61 – 8.56 (m, 1H), 7.96 – 7.90 (m, 2H), 7.59 – 7.55 (m, 1H), 7.49 – 7.42 (m, 2H), 7.42 – 7.37 (m, 1H), 7.17 (d, *J* = 8.0 Hz, 2H), 7.12 – 7.05 (m, 3H), 4.97 (d, *J* = 8.4 Hz, 1H), 4.56 (d, *J* = 7.9 Hz, 2H), 3.67 (s, 3H), 3.61 (s, 3H), 3.17 – 2.93 (m, 4H), 1.37 (s, 9H). **^{13}C NMR** (100 MHz, $CDCl_3$) δ 172.4, 171.8, 158.0, 155.1, 153.0, 150.0, 140.6, 139.4, 135.2, 129.9, 129.1, 128.8, 127.9, 127.1, 121.4, 120.1, 80.1, 54.4, 52.3, 52.0, 46.3, 39.9, 38.1, 28.4. **HRMS** (ESI^+) m/z calcd. For $C_{30}H_{35}N_2O_6^+$ $[M+H]^+$: 519.2495, found 519.2496. **Specific Rotation** $[\alpha]_D^{26}$ +37.1 (*c* 1.0, $CHCl_3$). **HPLC Analysis** CHIRALCEL OD-H, 25 ºC; *n*-hexane:*i*-PrOH = 75:25, 1.0 mL/min, 254 nm, t_{R1} (minor enantiomer, major diastereomer) = 10.94, t_{R2} (major enantiomer, major diastereomer) = 13.34, t_{R3} (minor enantiomer, minor diastereomer) = 20.56, t_{R4} (major enantiomer, minor diastereomer) = 29.46, 95:5 dr, 4.3:1 er.

The absolute stereochemistry was assigned by analogy to compound **3x** and **4k**.

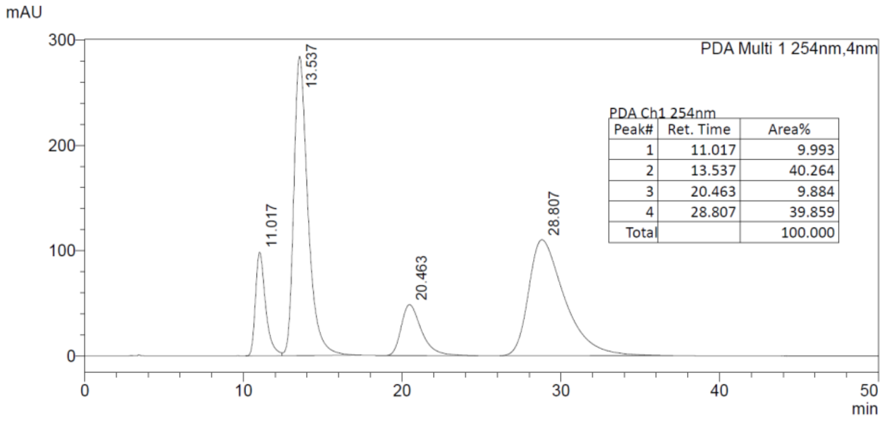

Supplementary Fig. 104. HPLC traces of **6b** using NHC **5a** (4.0:1 dr, 1:1 er).

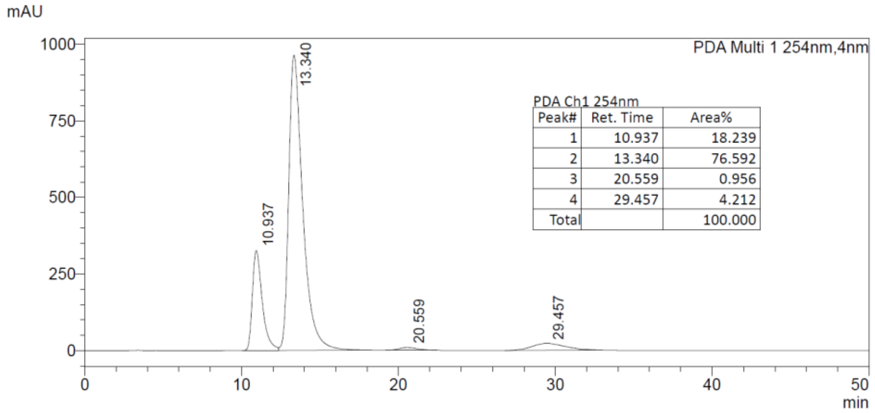

Supplementary Fig. 105. HPLC traces of **6b** using NHC **5f**.

400 MHz, ^{1}H NMR in CDCl3.

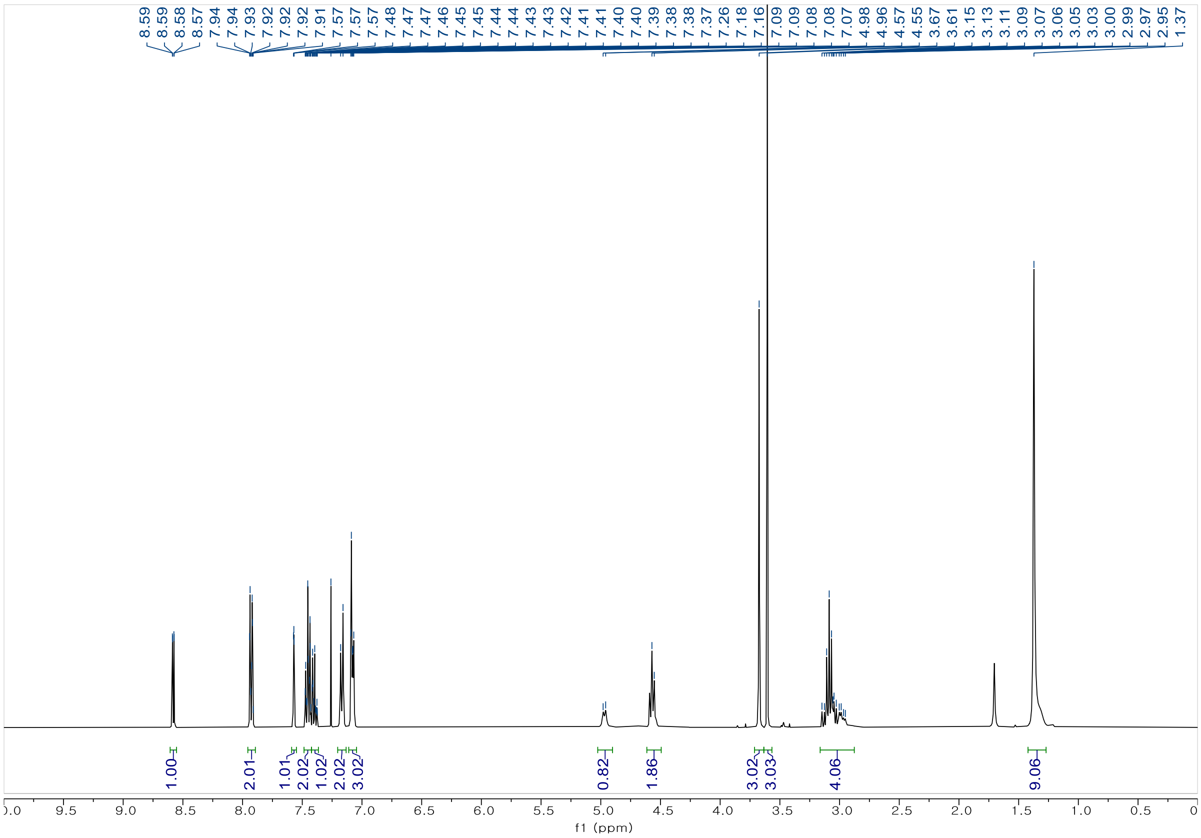

100 MHz, ^{13}C NMR in CDCl3.

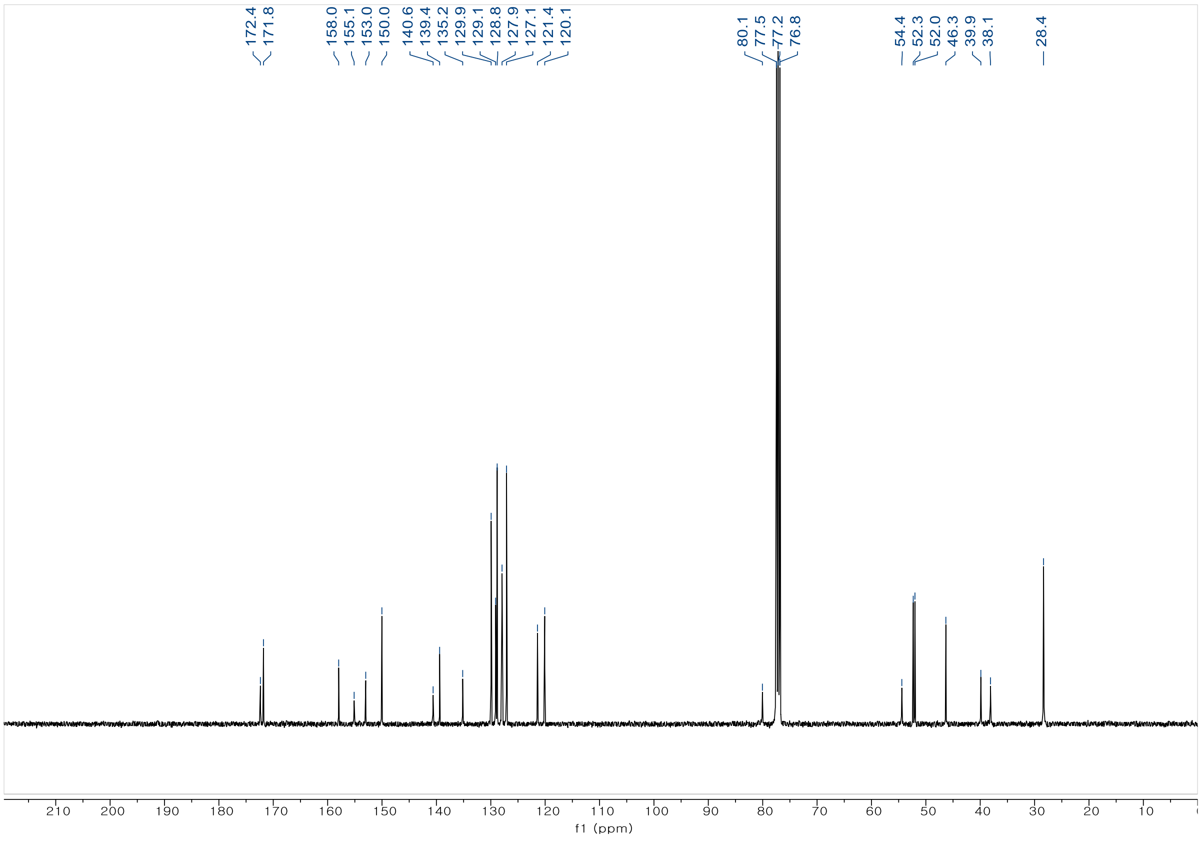

Supplementary Fig. 106. ^{1}H and ^{13}C NMR of **6b**.

Methyl (*S*)-3-(4,5-diphenyloxazol-2-yl)-3-(2-phenylpyridin-4-yl)propanoate (6c)

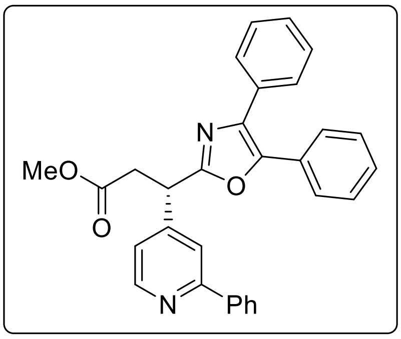

Prepared according to **GP**. Desired product **6c** was obtained as colorless gum (15.2 mg, 62% isolated yield). **^{1}H NMR** (500 MHz, $CDCl_3$) δ 8.67 (dd, *J* = 5.2, 0.8 Hz, 1H), 8.01 – 7.97 (m, 2H), 7.76 (s, 1H), 7.68 – 7.64 (m, 2H), 7.54 – 7.51 (m, 2H), 7.50 – 7.45 (m, 2H), 7.44 – 7.40 (m, 1H), 7.40 – 7.36 (m, 2H), 7.36 – 7.30 (m, 4H), 7.27 (dd, *J* = 5.1, 1.7 Hz, 1H), 4.81 (t, *J* = 7.7 Hz, 1H), 3.69 (s, 3H), 3.52 (dd, *J* = 16.7, 7.8 Hz, 1H), 3.11 (dd, *J* = 16.7, 7.4 Hz, 1H). **^{13}C NMR** (100 MHz, $CDCl_3$) δ 171.3, 161.8, 158.3, 150.4, 148.8, 146.3, 139.2, 135.4, 132.3, 129.3, 128.9, 128.8, 128.8, 128.8, 128.7, 128.4, 128.1, 127.2, 126.7, 121.5, 120.2, 52.2, 41.1, 38.5. **HRMS** (ESI^+) m/z calcd. For $C_{30}H_{25}N_2O_3^+$ $[M+H]^+$: 461.1865, found 461.1864. **Specific Rotation** $[\alpha]_D^{20}$ +40.6 (*c* 1.0, $CHCl_3$). **HPLC Analysis**. CHIRALCEL OD-H, 25 °C; *n*-hexane:*i*-PrOH = 65:35, 1.0 mL/min, 254 nm, t_{R1} (major) = 7.45 min, t_{R2} (minor) = 10.22 min, 86:14 er.

The absolute stereochemistry was assigned by analogy to compound **3x** and **4k**.

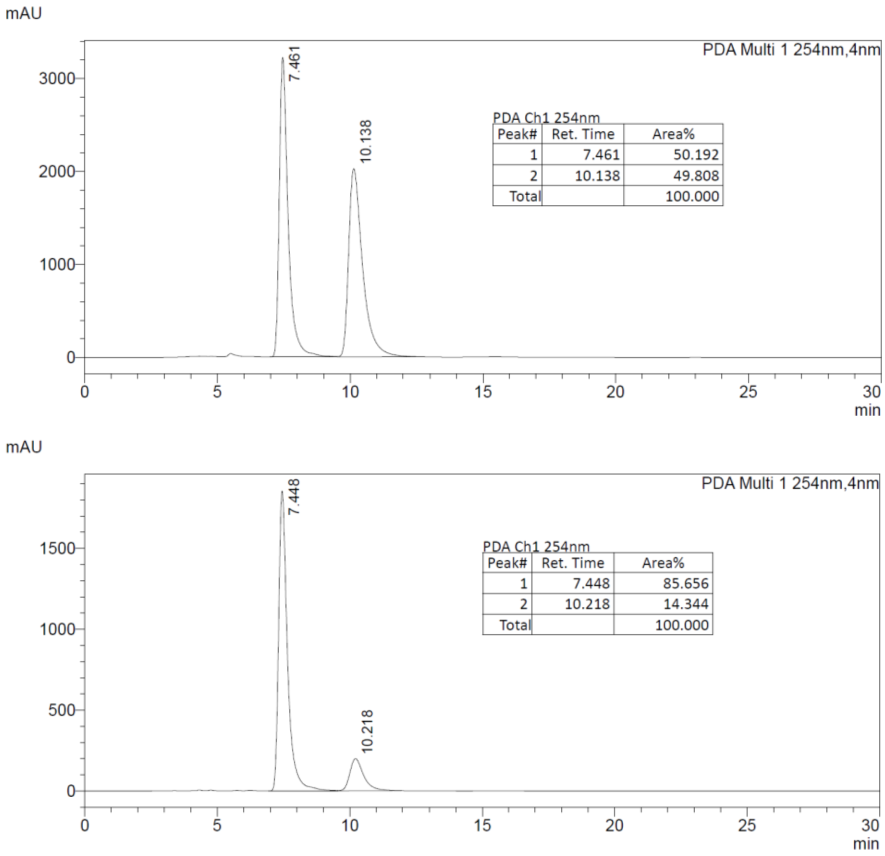

Supplementary Fig. 107. HPLC traces of *rac*-**6c** (top) and enantioenriched-**6c** (bottom).

500 MHz, ^{1}H NMR in $CDCl_3$

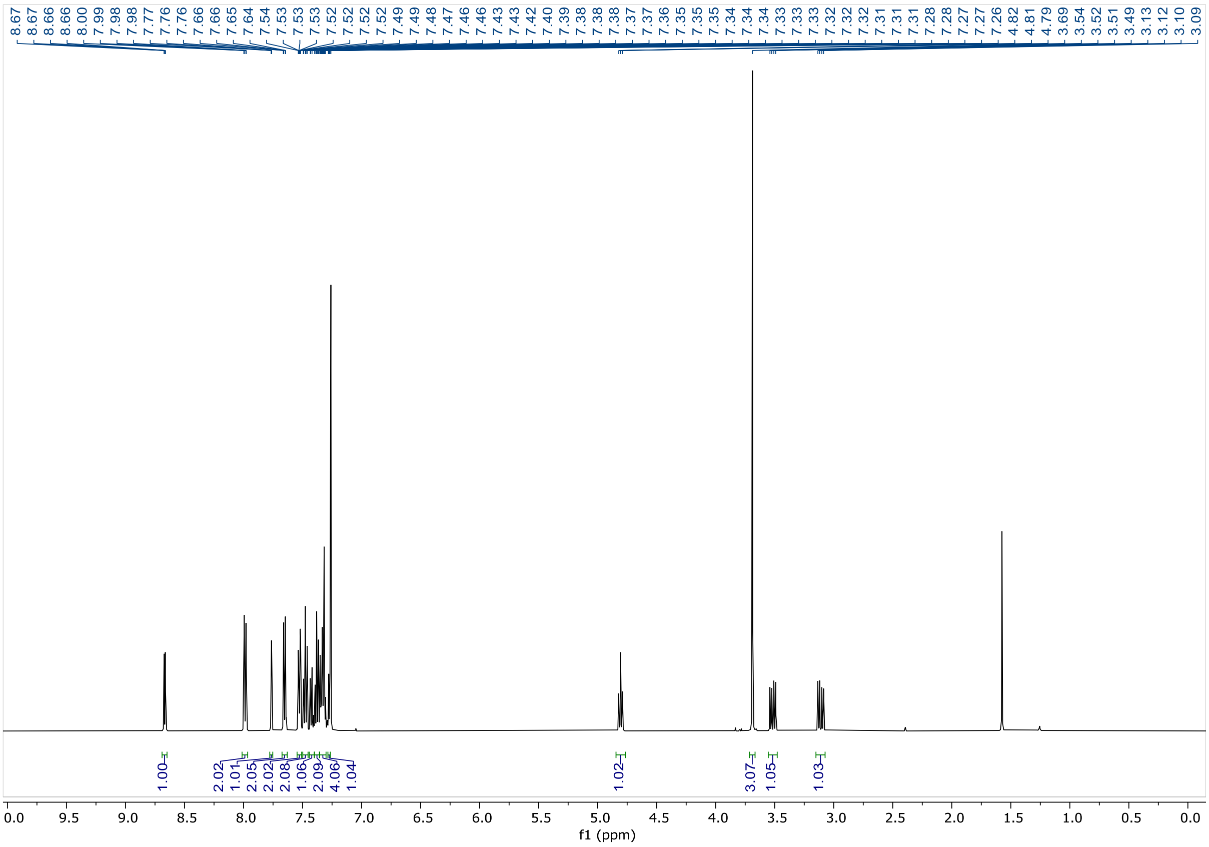

100 MHz, ^{13}C NMR in $CDCl_3$

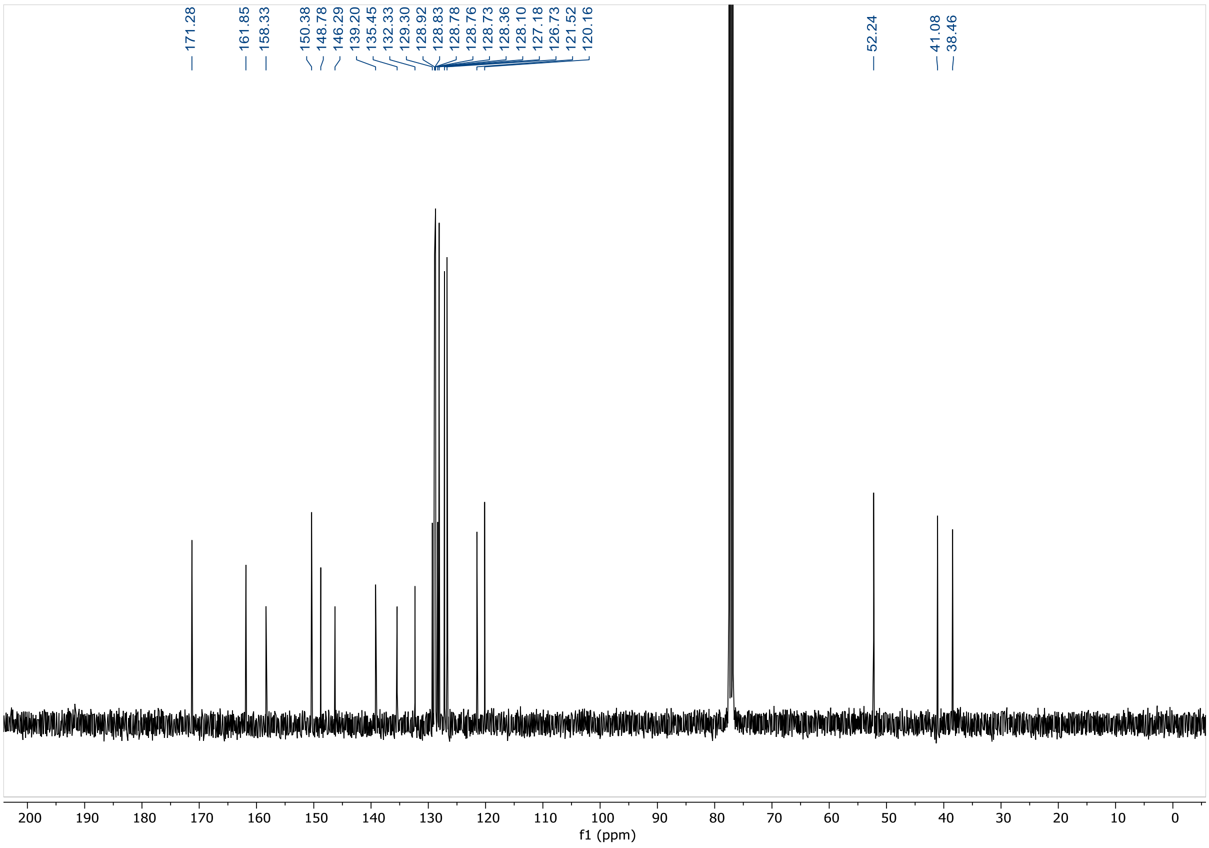

Supplementary Fig. 108. ^{1}H and ^{13}C NMR of **6c**.

Ethyl (*S*)-2-(4-isobutoxy-3-(3-methoxy-3-oxo-1-(2-phenylpyridin-4-yl)propyl)phenyl)-4-methylt-hiazole-5-carboxylate (6d)

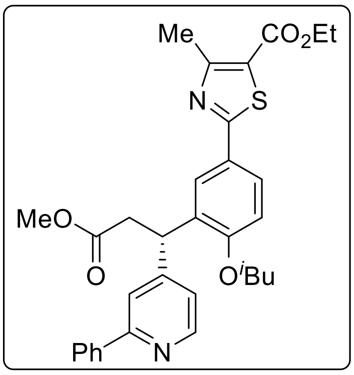

Prepared according to **GP**. Desired product **6d** was obtained as colorless oil (16.2 mg, 50% isolated yield). **^{1}H NMR** (400 MHz, $CDCl_3$) δ 8.56 (d, *J* = 5.1 Hz, 1H), 7.95 – 7.87 (m, 3H), 7.83 (dd, *J* = 8.5, 2.3 Hz, 1H), 7.64 – 7.59 (m, 1H), 7.49 – 7.35 (m, 3H), 7.12 (dd, *J* = 5.2, 1.7 Hz, 1H), 6.87 (d, *J* = 8.6 Hz, 1H), 5.00 – 4.92 (m, 1H), 4.35 (q, *J* = 7.1 Hz, 2H), 3.77 – 3.67 (m, 2H), 3.63 (s, 3H), 3.20 – 3.11 (m, 2H), 2.77 (s, 3H), 2.01 (dq, *J* = 13.3, 6.6 Hz, 1H), 1.38 (t, *J* = 7.1 Hz, 3H), 0.96 (d, *J* = 6.7 Hz, 3H), 0.93 (d, *J* = 6.7 Hz, 3H). **^{13}C NMR** (100 MHz, $CDCl_3$) δ 172.0, 169.8, 162.5, 161.2, 158.9, 157.7, 152.8, 149.8, 139.6, 130.9, 129.0, 128.8, 127.6, 127.1, 126.6, 125.7, 121.6, 121.1, 120.3, 111.9, 75.0, 61.3, 52.1, 40.9, 38.8, 28.4, 19.4, 19.3, 17.7, 14.5. **HRMS** (ESI^+) m/z calcd. for $C_{32}H_{35}N_2O_5S^+$ $[M+H]^+$: 559.2267, found: 559.2266. **Specific Rotation** $[\alpha]_D^{25}$ +116.4 (*c* 1.0, $CHCl_3$). **HPLC Analysis** CHIRALCEL OD-H, 25 °C; *n*-hexane:*i*-PrOH = 85:15, 1.0 mL/min, 254 nm, t_{R1} (major) = 9.77 min, t_{R2} (minor) = 12.01 min, 94:6 er.

The absolute stereochemistry was assigned by analogy to compound **3x** and **4k**.

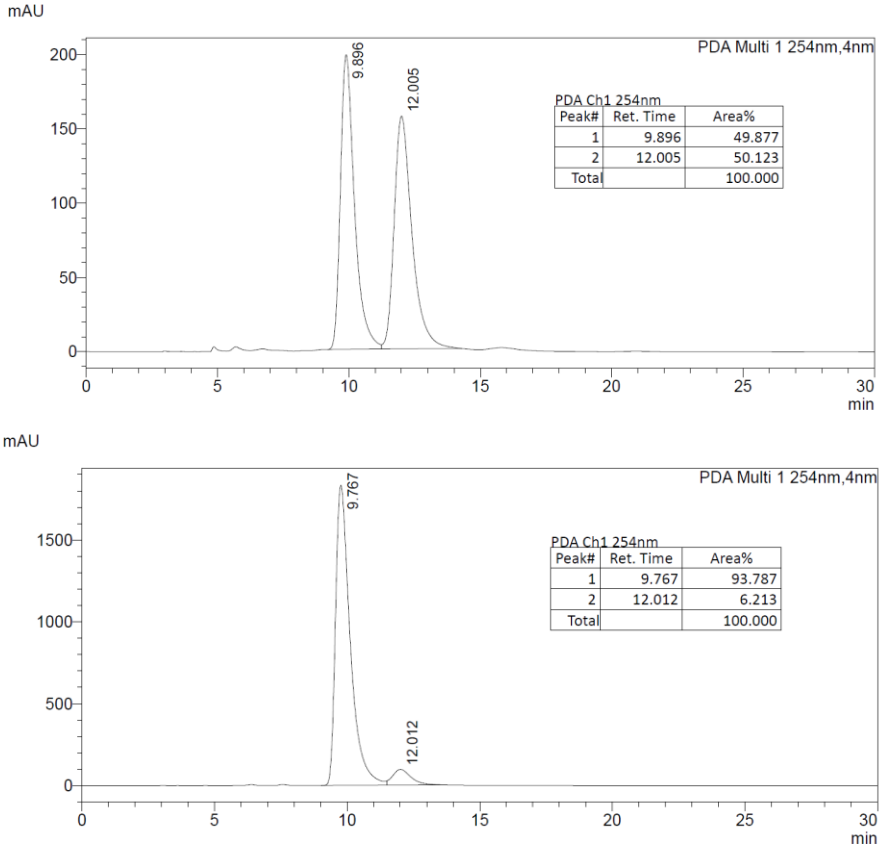

Supplementary Fig. 109. HPLC traces of *rac*-**6d** (top) and enantioenriched-**6d** (bottom).

400 MHz, ^{1}H NMR in $CDCl_3$.

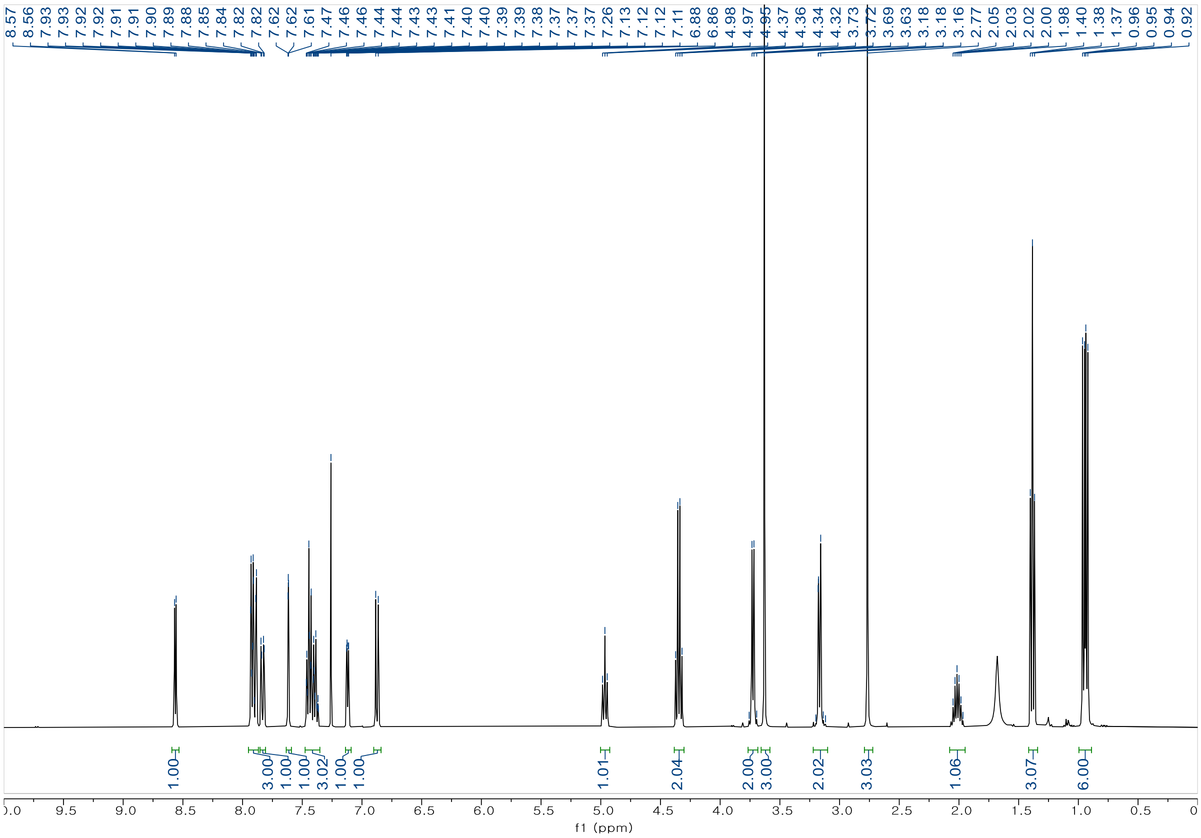

100 MHz, ^{13}C NMR in $CDCl_3$.

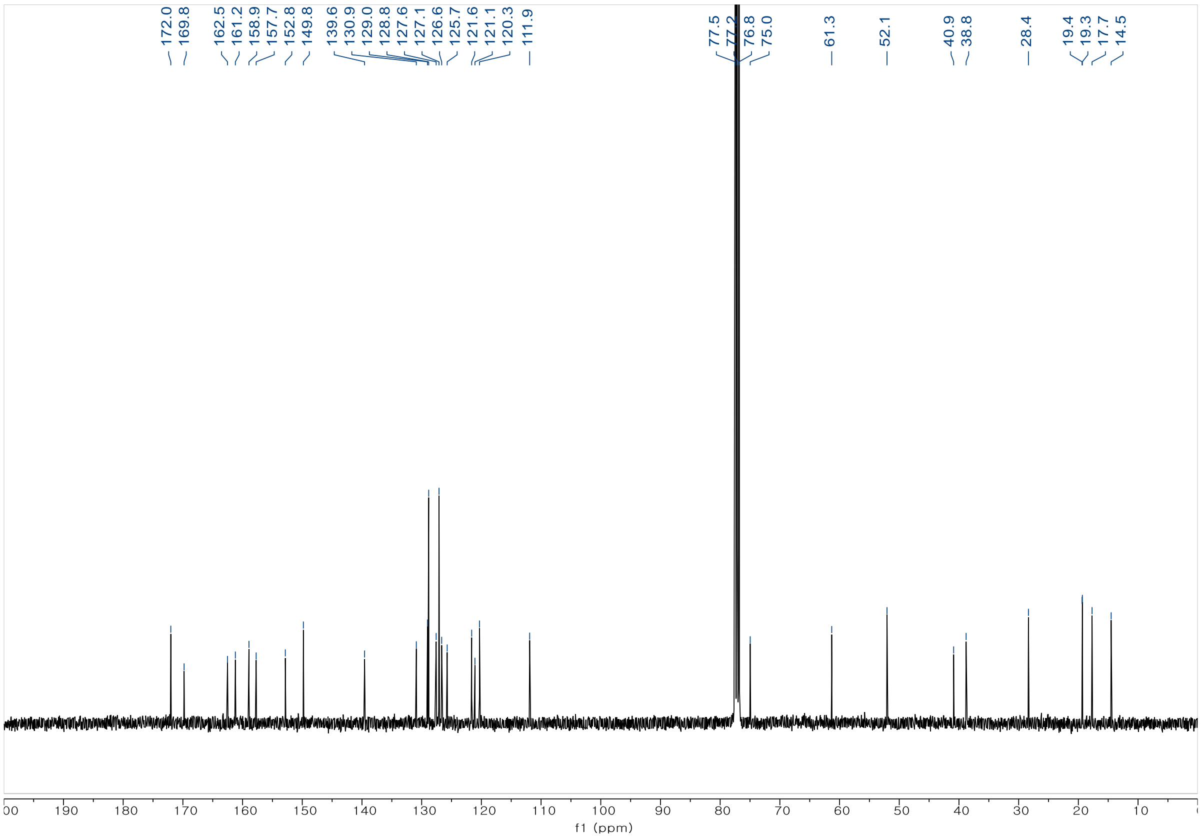

Supplementary Fig. 110. ^{1}H and ^{13}C NMR of **6d**.

X-Ray Crystallographic Data

Crystallographic data for 3x (CCDC 2116151)

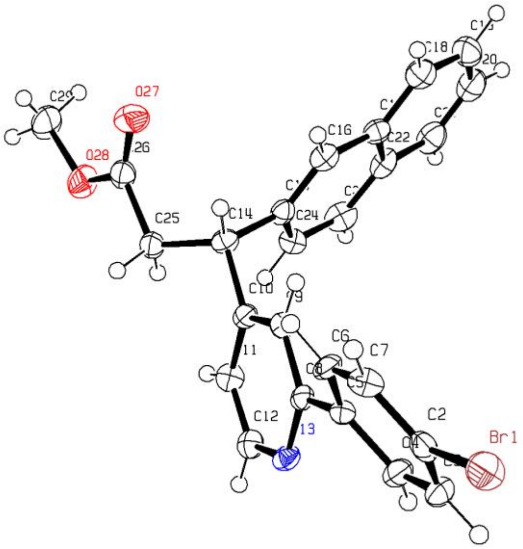

Supplementary Table 6. Crystal data and structure refinement for **3x**.

Empirical formula	$C_{25}H_{20}BrNO_2$	
Formula weight	446.33	
Temperature	173(2) K	
Wavelength	0.71073 Å	
Crystal system	Monoclinic	
Space group	$P2_1$	
Unit cell dimensions	a = 10.3624(6) Å	α = 90°
	b = 6.1965(4) Å	β = 95.3572(19)°
	c = 15.8980(10) Å	γ = 90°
Volume	1016.36(11) $Å^3$	
Z	2	
Density (calculated)	1.458 Mg/m^3	
Absorption coefficient	2.043 mm^{-1}	
F(000)	456	
Crystal size	0.171 x 0.092 x 0.039 mm^3	
Theta range for data collection	3.094 to 25.995°.	
Index ranges	–12<=h<=12, –7<=k<=7, –19<=l<=19	
Reflections collected	14020	
Independent reflections	3889 [R(int) = 0.0463]	
Completeness to theta = 25.242°	98.2 %	
Absorption correction	Semi-empirical from equivalents	
Max. and min. transmission	0.7456 and 0.6076	
Refinement method	Full-matrix least-squares on F^2	
Data / restraints / parameters	3889 / 1 / 265	
Goodness-of-fit on F^2	1.151	
Final R indices [I>2sigma(I)]	R1 = 0.0427, wR2 = 0.1037	
R indices (all data)	R1 = 0.0470, wR2 = 0.1078	
Absolute structure parameter	0.057	
Largest diff. peak and hole	0.993 and –0.403 e·$Å^{-3}$	

Crystallographic data for 4k (CCDC 2116149)

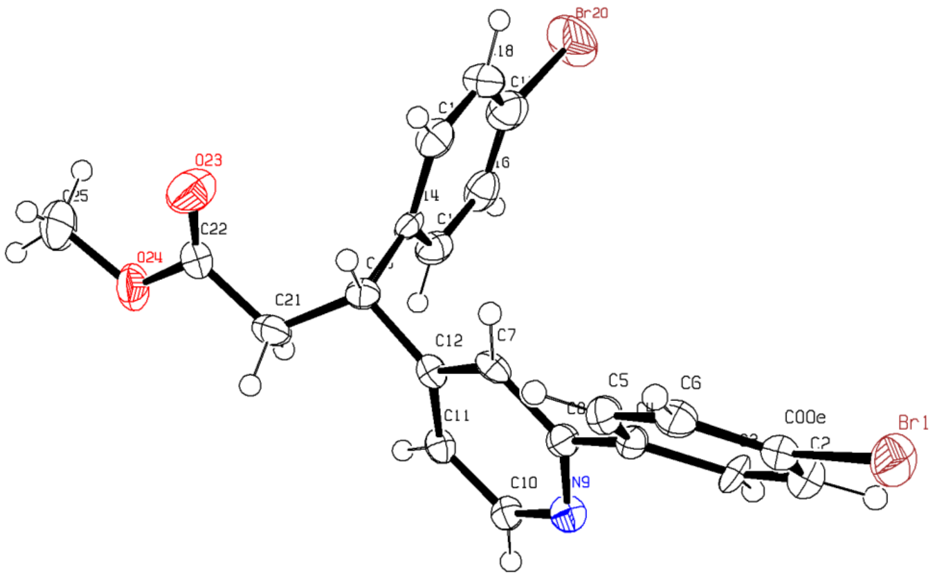

Supplementary Table 7. Crystal data and structure refinement for **4k**.

Empirical formula	$C_{21}H_{17}Br_2NO_2$	
Formula weight	475.18	
Temperature	173(2) K	
Wavelength	0.71073 Å	
Crystal system	Monoclinic	
Space group	*C*2	
Unit cell dimensions	a = 31.1342(18) Å	α = 90°
	b = 5.9956(4) Å	β = 96.507(2)°
	c = 10.6196(6) Å	γ = 90°
Volume	1969.6(2) $Å^3$	
Z	4	
Density (calculated)	1.602 Mg/m^3	
Absorption coefficient	4.130 mm^{-1}	
F(000)	944	
Crystal size	0.131 x 0.061 x 0.018 mm^3	
Theta range for data collection	2.457 to 25.488°.	
Index ranges	–37<=h<=37, –7<=k<=7, –12<=l<=11	
Reflections collected	6728	
Independent reflections	3362 [R(int) = 0.0525]	
Completeness to theta = 25.242°	96.3 %	
Absorption correction	Semi-empirical from equivalents	
Max. and min. transmission	0.7454 and 0.5986	
Refinement method	Full-matrix least-squares on F^2	
Data / restraints / parameters	3362 / 325 / 237	
Goodness-of-fit on F^2	1.099	
Final R indices [I>2sigma(I)]	R1 = 0.0677, wR2 = 0.1097	
R indices (all data)	R1 = 0.0857, wR2 = 0.1142	
Absolute structure parameter	0.025	
Largest diff. peak and hole	1.546 and –0.854 e·$Å^{-3}$	

Crystallographic data for *rac*-3d (CCDC 2116154)

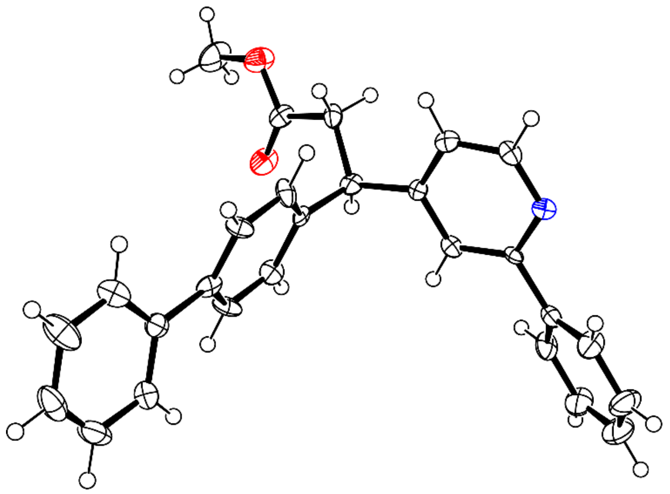

Supplementary Table 8. Crystal data and structure refinement for *rac*-**3d**.

Empirical formula	$C_{27}H_{23}NO_2$	
Formula weight	393.46	
Temperature	173(2) K	
Wavelength	0.71073 Å	
Crystal system	Orthorhombic	
Space group	$P2_12_12_1$	
Unit cell dimensions	a = 21.927(2) Å	α = 90°
	b = 6.0950(7) Å	β = 90°
	c = 15.6811(16) Å	γ = 90°
Volume	2095.7(4) Å^3	
Z	4	
Density (calculated)	1.247 Mg/m^3	
Absorption coefficient	0.078 mm^{-1}	
F(000)	832	
Crystal size	0.254 x 0.034 x 0.027 mm^3	
Theta range for data collection	2.598 to 25.499°.	
Index ranges	–26<=h<=26, –7<=k<=6, –14<=l<=18	
Reflections collected	13131	
Independent reflections	3836 [R(int) = 0.1244]	
Completeness to theta = 25.242°	98.4 %	
Absorption correction	Semi-empirical from equivalents	
Max. and min. transmission	0.7455 and 0.3882	
Refinement method	Full-matrix least-squares on F^2	
Data / restraints / parameters	3836 / 0 / 263	
Goodness-of-fit on F^2	1.097	
Final R indices [I>2sigma(I)]	R1 = 0.0949, wR2 = 0.2047	
R indices (all data)	R1 = 0.1203, wR2 = 0.2170	
Largest diff. peak and hole	0.310 and –0.285 e·Å^{-3}	

Crystallographic data for *rac*-3e (CCDC 2116152)

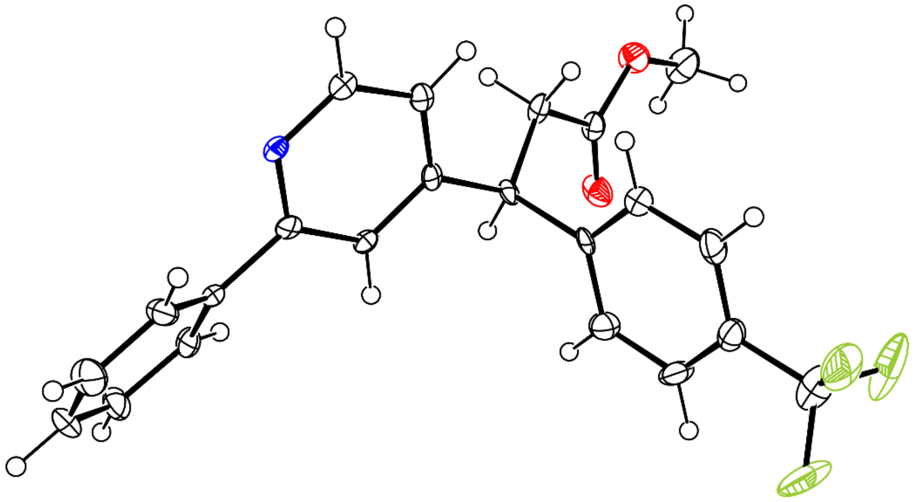

Supplementary Table 9. Crystal data and structure refinement for *rac*-**3e**.

Empirical formula	$C_{22}H_{18}F_3NO_2$	
Formula weight	385.37	
Temperature	173(2) K	
Wavelength	0.71073 Å	
Crystal system	Orthorhombic	
Space group	$P2_12_12_1$	
Unit cell dimensions	a = 5.9980(5) Å	α = 90°
	b = 13.7113(14) Å	β = 90°
	c = 22.423(2) Å	γ = 90°
Volume	1844.1(3) $Å^3$	
Z	4	
Density (calculated)	1.388 Mg/m^3	
Absorption coefficient	0.109 mm^{-1}	
F(000)	800	
Crystal size	0.137 x 0.043 x 0.035 mm^3	
Theta range for data collection	2.971 to 25.998°.	
Index ranges	–7<=h<=7, –16<=k<=16, –27<=l<=27	
Reflections collected	26423	
Independent reflections	3630 [R(int) = 0.1025]	
Completeness to theta = 25.242°	99.5 %	
Absorption correction	Semi-empirical from equivalents	
Max. and min. transmission	0.7455 and 0.5558	
Refinement method	Full-matrix least-squares on F^2	
Data / restraints / parameters	3630 / 168 / 291	
Goodness-of-fit on F^2	1.194	
Final R indices [I>2sigma(I)]	R1 = 0.0804, wR2 = 0.1328	
R indices (all data)	R1 = 0.0962, wR2 = 0.1379	
Largest diff. peak and hole	0.249 and –0.256 e·$Å^{-3}$	

Crystallographic data for *rac*-4d (CCDC 2116153)

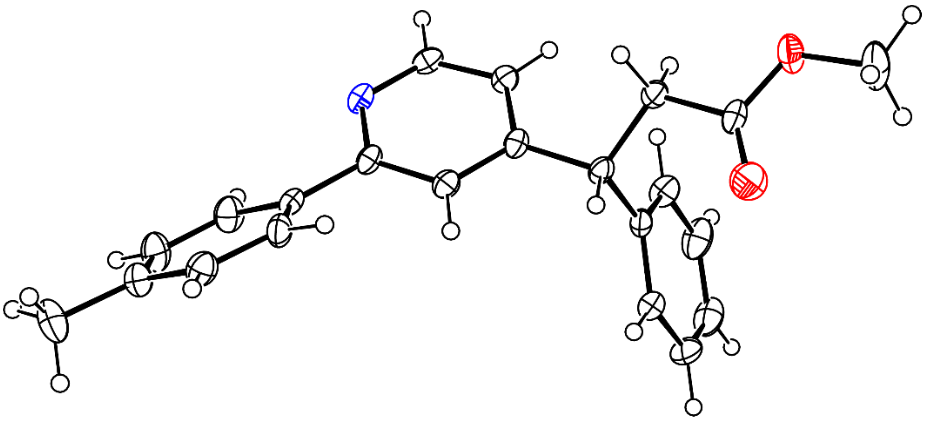

Supplementary Table 10. Crystal data and structure refinement for *rac*-**4d**.

Empirical formula	$C_{22}H_{21}NO_2$	
Formula weight	331.40	
Temperature	173(2) K	
Wavelength	0.71073 Å	
Crystal system	Monoclinic	
Space group	$P2_1$	
Unit cell dimensions	a = 11.4488(8) Å	α = 90°
	b = 6.2146(4) Å	β = 113.957(2)°
	c = 13.6220(10) Å	γ = 90°
Volume	885.70(11) $Å^3$	
Z	2	
Density (calculated)	1.243 Mg/m^3	
Absorption coefficient	0.079 mm^{-1}	
F(000)	352	
Crystal size	0.171 x 0.026 x 0.024 mm^3	
Theta range for data collection	3.009 to 27.499°.	
Index ranges	–14<=h<=14, –8<=k<=8, -17<=l<=17	
Reflections collected	13913	
Independent reflections	3969 [R(int) = 0.1086]	
Completeness to theta = 25.242°	98.6 %	
Absorption correction	Semi-empirical from equivalents	
Max. and min. transmission	0.7456 and 0.3341	
Refinement method	Full-matrix least-squares on F^2	
Data / restraints / parameters	3969 / 1 / 231	
Goodness-of-fit on F^2	1.081	
Final R indices [I>2sigma(I)]	R1 = 0.0789, wR2 = 0.1676	
R indices (all data)	R1 = 0.0957, wR2 = 0.1756	
Largest diff. peak and hole	0.321 and –0.262 e·$Å^{-3}$	

Supplementary References

[1] Klinger, D.; Nilles, K.; Theato, P. Synthesis of Polymeric 1-Iminopyridinium Ylides as Photoreactive Polymers. *J. Polym. Sci.: Part A: Polymer Chemistry* **2010**, *48*, 832–844.

[2] Johnston, K. A.; Allcock, R. W.; Jiang, Z.; Collier, I. D.; Blakli, H.; Rosair, G. M.; Bailey, P. D.; Morgan, K. M.; Kohno, Y.; Adams, D. R. Concise Routes to Pyrazolo[1,5-*a*]pyridin-3-yl Pyridazin-3-ones. *Org. Biomol. Chem.* **2008**, *6*, 175–186.

[3] Grayson, E. J.; Bernardes, G. J. L.; Chalker, J. M.; Boutureira, O.; Koeppe, J. R.; Davis, B. G. A. Coordinated Synthesis and Conjugation Strategy for the Preparation of Homogeneous Glycoconjugate Vaccine Candidates. *Angew. Chem., Int. Ed.* **2011**, *50*, 4127–4132.

[4] Battistuzzi, G.; Cacchi, S.; Fabrizi, G. An Efficient Palladium-Catalyzed Synthesis of Cinnamaldehydes from Acrolein Diethyl Acetal and Aryl Iodides and Bromides. *Org. Lett.* **2003**, *5*, 777–780.

[5] Lee, Y. H.; Morandi, B. Metathesis-active ligands enable a catalytic functional group metathesis between aroyl chlorides and aryl iodides. *Nature Chemistry* **2018**, *10*, 1016–1022.

[6] Evans, D. A.; Nagorny, P.; Reynolds, D. J.; McRae, K. J. Enantioselective Synthesis of Oasomycin A, Part II: Synthesis of theC29–C46 Subunit. *Angew. Chem., Int. Ed.* **2007**, *46*, 541−544.

[7] (a) Lee, W.; Jeon, H. J.; Jung, H.; Kim, D.; Seo, S.; Chang, S. Controlled Relay Process to Access N-Centered Radicals for Catalyst-free Amidation of Aldehydes under Visible Light. *Chem* **2021**, *7*, 495–508. (b) Yang, Q.; Wang, Y.; Luo, S.; Wang, J. Kinetic Resolution and Dynamic Kinetic Resolution of Chromene by Rhodium-Catalyzed Asymmetric Hydroarylation. *Angew. Chem., Int. Ed.* **2019**, *131*, 5397–5401.

[8] Dhayalan, V.; Gadekar, S. C.; Alassad, Z.; Milo, A. Unravelling mechanistic features of organocatalysis with in situ modifications at the secondary sphere. *Nat. Chem.* **2019**, *11*, 543.

[9] Kerr, M. S.; Read de Alaniz, J.; Rovis, T. An Efficient Synthesis of Achiral and Chiral 1,2,4-Triazolium Salts: Bench Stable Precursors for N-Heterocyclic Carbenes. *J. Org. Chem.* **2005,** *70*, 5725–5728.

[10] (a) Liu, Q.; Perreault, S.; Rovis, T. Catalytic Asymmetric Intermolecular Stetter Reaction of Glyoxamides with Alkylidenemalonates. J. Am. Chem. Soc. 2008, 130, 14066–14067. (b) Huang, X.-L.; He, L.; Shao, P.-L.; Ye, S. [4+2] Cycloaddition of Ketenes with N-Benzoyldiazenes Catalyzed by N-Heterocyclic Carbene. *Angew. Chem., Int. Ed.* **2009**, *48*, 192−195. (c) Rovis, T.; Vora, H. U. Triazolium carbene catalysts and stereoselective bond forming reactions thereof. U.S. Patent US 20130274470, Octover 17, 2013.

[11] Guin, J.; De Sarkar, S.; Grimme, S.; Studer, A. Biomimetic Carbene-Catalyzed Oxidations of Aldehydes Using TEMPO. *Angew. Chem., Int. Ed.* **2008**, *47*, 8727−8730.

[12] Zhang, Y., Du, Y., Huang, Z., Xu, J., Wu, X., Wang, Y., Wang, M., Yang, S., Webster, R. D.; Chi, Y. R. N-Heterocyclic Carbene-Catalyzed Radical Reactions for Highly Enantioselective β-Hydroxylation of Enals. *J. Am. Chem. Soc.* **2015**, 137, 2416−2419.
